# Recurrent *GNAQ* mutation encoding T96S in natural killer/T cell lymphoma

**DOI:** 10.1038/s41467-019-12032-9

**Published:** 2019-09-16

**Authors:** Zhaoming Li, Xudong Zhang, Weili Xue, Yanjie Zhang, Chaoping Li, Yue Song, Mei Mei, Lisha Lu, Yingjun Wang, Zhiyuan Zhou, Mengyuan Jin, Yangyang Bian, Lei Zhang, Xinhua Wang, Ling Li, Xin Li, Xiaorui Fu, Zhenchang Sun, Jingjing Wu, Feifei Nan, Yu Chang, Jiaqin Yan, Hui Yu, Xiaoyan Feng, Guannan Wang, Dandan Zhang, Xuefei Fu, Yuan Zhang, Ken H. Young, Wencai Li, Mingzhi Zhang

**Affiliations:** 1 Department of Oncology, The First Affiliated Hospital of Zhengzhou University, 450052 Zhengzhou, China; 2 Lymphoma Diagnosis and Treatment Center of Henan Province, 450000 Zhengzhou, China; 3 Institute of Clinical Medicine, The First Affiliated Hospital of Zhengzhou University, 450052 Zhengzhou, China; 4 Medical Research Centre, The First Affiliated Hospital of Zhengzhou University, 450052 Zhengzhou, China; 5 Department of Pathology, The First Affiliated Hospital of Zhengzhou University, 450052 Zhengzhou, China; 6 Novogene Bioinformatics Technology Co, Ltd, 38 Xueqing Road, 100083 Beijing, China; 7 The Academy of Medical Science of Zhengzhou University, 450052 Zhengzhou, China; 8 Department of Hematopathology, The University of Texas MD Anderson Cancer Center, Houston, TX 77030 USA

**Keywords:** Cell death, Cell growth, Cancer genetics, Cancer genomics, Genomics

## Abstract

Natural killer/T cell lymphoma (NKTCL) is a rare and aggressive malignancy with a higher prevalence in Asia and South America. However, the molecular genetic mechanisms underlying NKTCL remain unclear. Here, we identify somatic mutations of *GNAQ* (encoding the T96S alteration of Gαq protein) in 8.7% (11/127) of NKTCL patients, through whole-exome/targeted deep sequencing. Using conditional knockout mice (*Ncr1-Cre-Gnaq*^*fl/fl*^), we demonstrate that Gαq deficiency leads to enhanced NK cell survival. We also find that Gαq suppresses tumor growth of NKTCL via inhibition of the AKT and MAPK signaling pathways. Moreover, the Gαq T96S mutant may act in a dominant negative manner to promote tumor growth in NKTCL. Clinically, patients with *GNAQ* T96S mutations have inferior survival. Taken together, we identify recurrent somatic *GNAQ* T96S mutations that may contribute to the pathogenesis of NKTCL. Our work thus has implications for refining our understanding of the genetic mechanisms of NKTCL and for the development of therapies.

## Abstract (web summary)

Natural killer/T cell lymphoma (NKTCL) is a rare and aggressive disease. Here, the authors identify recurrent somatic mutations of *GNAQ* in NKTCL, and model how this mutation contributes to NKTCL pathogenesis.

## Introduction

Natural killer/T cell lymphoma (NKTCL) is an aggressive subtype of non-Hodgkin’s lymphoma that is rare overall but shows a predilection for Asian and South American populations^1–5^. NKTCLs are almost exclusively extranodal involving the nasal and/or paranasal area, with a strong association with Epstein–Barr virus (EBV) infection^3,4,6–8^. Despite a multimodality chemotherapy and radiotherapy treatment approach, the survival of patients with NKTCL is poor^9–12^.

The molecular pathogenesis of NKTCL currently remains elusive^13,14^. In the past decade, however, several oncogenic pathways, including Janus kinase (JAK)—signal transducer and activator of transcription (STAT), mitogen-activated protein kinase (MAPK), AKT, and NF-κB signaling pathways, have been identified in the development of NKTCL by gene expression profiling^15–20^. Recently, *DDX3X*, *STAT3*, *JAK3*, *BCOR*, and *TP53* have been revealed as novel genes mutated in NKTCL by high-throughput sequencing studies^21–28^.

In this study, we sought to identify additional oncogenic drivers and altered pathways that contribute to NKTCL tumorigenesis in 127 patients with NKTCL through whole-exome/targeted deep sequencing. In addition to frequently mutated genes reported previously, somatic mutations of *GNAQ* (encoding the T96S alteration of Gαq protein) were identified in 8.7% (11/127) of the patients with NKTCL. Experiments using conditional knockout mice demonstrated that Gαq deficiency enhanced the survival of natural killer (NK) cells. We also found that Gαq suppressed NKTCL tumor growth via inhibition of the AKT and MAPK signaling pathways. Furthermore, the Gαq T96S mutant might act in a dominant negative manner to promote tumor growth in NKTCL. In addition, we observed that patients with *GNAQ* T96S mutations had inferior survival.

To our knowledge, the present study includes one of the largest series of NKTCL patients ever described and defines in detail the genetic landscape of mutations. In particular, recurrent *GNAQ* T96S mutations were detected in our NKTCL patients.

## Results

### Whole-exome sequencing of NKTCL

**Fig. 1:**
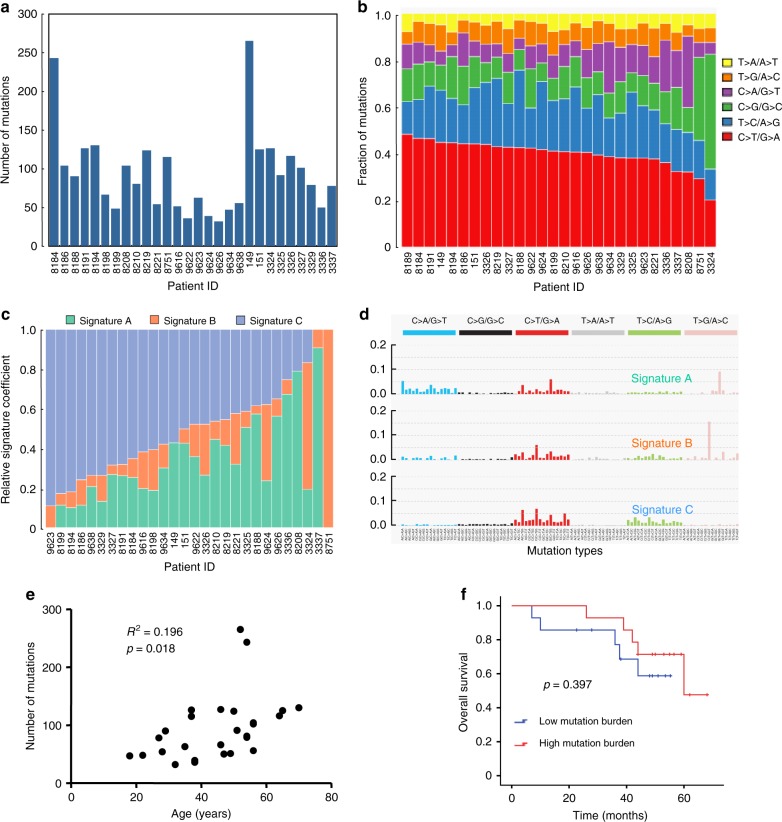
Whole-exome sequencing in 28 patients with NKTCL. **a** The number and type of nonsilent somatic mutations identified by whole-exome sequencing. **b** The spectrum of mutations in NKTCL. **c**, **d** Three dominant signatures identified by combined nonnegative matrix factorization clustering and correlation in NKTCL, with 30 curated mutational signatures defined by the COSMIC database. **e** The correlation analysis of nonsilent somatic mutations and the age of the NKTCL patients (*n* = 28; *R*^2^ = 0.196, *P* = 0.018). **f** The association of the somatic nonsilent mutation burden with overall survival in NKTCL (log-rank *P* = 0.397)

Whole-exome sequencing was performed on paired normal and tumor DNA from 28 patients with NKTCL (Supplementary Fig. 1). The demographics and clinical features of the patients are summarized in Supplementary Table 1. The mean sequencing depth was 84.67×, and a mean of 91.34% of the target sequence was covered to a depth of at least 20× (Supplementary Table 2). A total of 2642 nonsilent mutations, including 2374 missense, 114 nonsense, 105 splice site, 2 nonstop, and 47 insertion or deletion mutations, were identified (Supplementary Table 3). The somatic nonsilent mutation load per subject varied significantly in NKTCL (mean 94, range 32–265, Fig. 1a). Sanger sequencing yielded a 92.11% (70/76) validation rate (Supplementary Table 4). Next, we analyzed the mutation spectrum of NKTCL to determine whether mutagenic processes are operative in NKTCL. The predominant type of substitution was a C→T transition at NpCpG sites in NKTCL (Fig. 1b). Combining the nonnegative matrix factorization clustering and correlation with the 30 curated mutational signatures defined by the catalog of somatic mutations in cancer (COSMIC) database^29^ revealed three predominant signatures in NKTCL (Fig. 1c, d). The predominantly matched signature was Signature 1 (cosine similarity, 0.84), which was found in all tumor types and is thought to result from age-related accumulation of 5-methylcytosine deamination events.

Furthermore, we evaluated the relationship between somatic nonsilent mutation burden and the clinical characteristics of NKTCL. The results showed that somatic nonsilent mutation burden was correlated with patient age (*R*^2^ = 0.196, *P* = 0.018; Fig. 1e), and a significant correlation remained (*R*^2^ = 0.304, *P* = 0.0035; Supplementary Fig. 2) after excluding two patients with more than 200 mutations. However, no association was found between nonsilent mutation burden and other clinical features, such as gender, stage, and Eastern Cooperative Oncology group (ECOG) score. Moreover, no relationship between somatic nonsilent mutation burden and overall survival (OS) was observed in NKTCL patients revealed according to survival analysis (*P* = 0.397; Fig. 1f).

### Recurrent *GNAQ* mutations in NKTCL

Through whole-exome sequencing, frequent mutations in *GNAQ*, *TET2*, and *USP8*, as well as *DDX3X* and *STAT3* genes previously reported, were identified in our cohort of patients with NKTCL. Prompted by this discovery, we performed targeted deep sequencing in an extended validation group of 73 NKTCL cases. A total of 221 genes, including recurrently mutated genes detected by our exome sequencing and other genes previously reported to be mutated in NKTCL, were sequenced (Supplementary Table 5). The mean average coverage of the target genes was 1408× (a minimum of 1011×), and a mean of 99.38% of the target sequence was covered to a depth of at least 100× (Supplementary Tables 6 and 7).

**Fig. 2:**
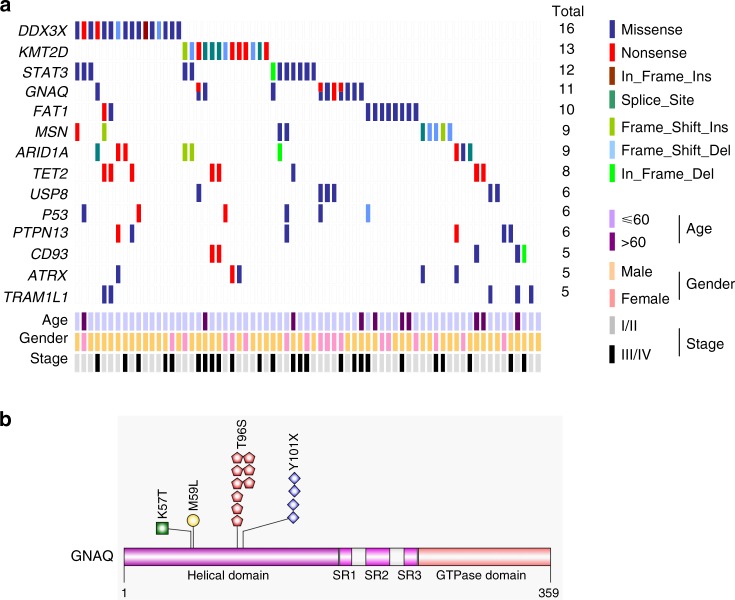
Frequently mutated genes. **a** Fourteen frequently mutated genes are ranked according to the frequency of mutations. Samples are displayed in columns. Gene mutations are shown in different colors according to the type of alteration. The total number of patients with mutations in each gene is listed on the right. **b** Mapping of *GNAQ* mutation sites in the total NKTCL patient cohort. Functional domains of the altered proteins are based on the UniProt database

To identify the frequently mutated genes in NKTCL, we combined the sequencing data of the discovery cohort and the validation cohort. After excluding implausible genes, such as the genes encoding olfactory receptors and extremely large proteins, and genes with very long introns, we identified the following 14 genes: *DDX3X* (16/101), *KMT2D* (also known as *MLL2*, 13/101), *STAT3* (12/101), *GNAQ* (11/101), *FAT1* (10/101), *MSN* (9/101), *ARID1A* (9/101), *TET2* (8/101), *USP8* (6/101), *TP53* (6/101), *PTPN13* (6/101), *CD93* (5/101), *ATRX* (5/101), and *TRAM1L1* (5/101). Each of these gene mutations was present in at least five NKTCL samples (Fig. 2a).

Notably, 15 point mutations in *GNAQ* were identified in 11 NKTCL samples: 9 mutations encoding T96S, 4 mutations encoding Y101X, 1 mutation encoding p.K57T, and 1 mutation encoding p.M59L. Mutations in T96S cooccurred with Y101X and p.M59L in three cases and one case, respectively (Fig. 2b and Supplementary Fig. 3). The frequently mutated genes were further validated in another independent cohort of NKTCL patients without matched normal tissue by performing whole-exome sequencing (*n* = 26, Supplementary Table 8). Interestingly, *GNAQ* T96S mutations were found in 2 of the 26 examined cases, and both were subsequently validated by Sanger sequencing (Supplementary Fig. 4). In this study, *GNAQ* T96S mutations were identified in 8.7% (11/127) of the NKTCL cases.

### Gαq deficiency leads to enhanced NK cell survival

**Fig. 3:**
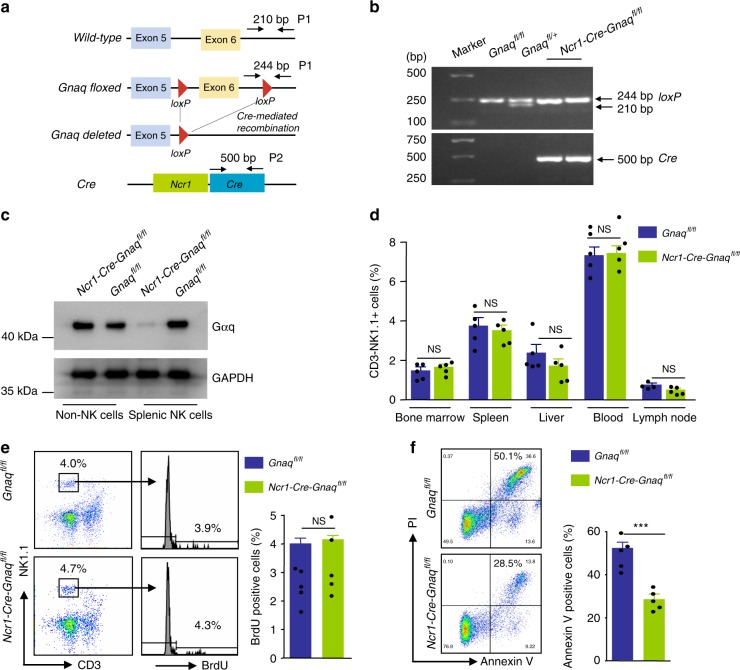
Gαq deficiency leads to enhanced NK cell survival. **a** In *Gnaq*^*fl/fl*^ mice, exon 6 of the *Gnaq* gene is flanked with *loxP* sites. Crossing *Gnaq*^*fl/fl*^ mice with *Ncr1-Cre* mice leads to *Cre*-mediated recombination, resulting in exon 6 deletion. The diagram is not to scale. **b** Genotyping of mouse tail DNA for the presence of *Cre* recombinase transgene and a floxed transgene. Genomic PCR was performed on DNA derived from different mice. The presence of the *Cre* recombinase gene is detected by the amplification of a 500-bp product. The floxed products expected (244 bp) are shown. **c** Gαq protein levels were measured by western blot in splentic NK and non-NK cells from *Ncr1-Cre-Gnaq*^*fl/fl*^ and *Gnaq*^*fl/f*^ mice. **d** The frequencies of NK cells in the bone marrow, spleen, peripheral blood, liver, and lymph node in *Ncr1-Cre-Gnaq*^*fl/fl*^ and *Gnaq*^*fl/f*^ mice (*n* = 5 per genotype). Bar graphs show the percentage of NK cells (CD3^−^NK1.1^+^). Data are representative of two independent experiments. **e** In vivo proliferation of splenic NK cells. Mice were injected intraperitoneally with BrdU (2 mg), and after 12 h, the incorporation of BrdU in splenic NK cells was analyzed. Numbers adjacent to outlined areas in the dot plot indicate the percentage of CD3^−^NK1.1^+^ cells. Histograms show the percentage of BrdU-positive cells (*n* = 5 per genotype). Data are representative of two independent experiments. **f** Purified splenic NK cells were cultured in media without IL-2 for 6 h, and viability was monitored by Annexin V staining. Histograms show the percentage of Annexin V-positive cells (*n* = 5 per genotype). Data are representative of two independent experiments. All data are expressed as the mean ± s.e.m.; NS, not significant. *P < 0.05, **P < 0.01, ***P < 0.001, unpaired two-tailed Student’s *t*-test. Source data are provided as a Source Data file

NKTCL is a mature NK cell neoplasm^30,31^. However, the function of *GNAQ* in normal NK cells is unclear. To address this question, we generated *Ncr1-Cre-Gnaq*^*fl/fl*^ mice that express Cre recombinase under control of the *Ncr1* promoter, which allowed NK cell-specific ablation of *Gnaq* (Fig. 3a, b). *Ncr1-Cre-Gnaq*^*fl/fl*^ mice were born at the expected Mendelian ratio without any visible alterations in organ morphology or overt pathology.

First, we verified Gαq expression in sorted NK cells isolated from *Ncr1-Cre-Gnaq*^*fl/fl*^ mice and their littermate controls that harbored *loxP*-flanked *Gnaq* alleles but lacked *Ncr1-Cre* (*Gnaq*^*fl/fl*^ mice). As expected, splenic NK cells from *Ncr1-Cre-Gnaq*^*fl/fl*^ mice showed significantly reduced Gαq expression compared with NK cells from control mice; however, there was no difference in Gαq expression on non-NK cells between the two groups (Fig. 3c). Therefore, *Gnaq* was deleted exclusively in NK cells.

Next, we determined whether *Gnaq* influences the distribution of NK cells within different compartments. However, no significant differences in NK cell frequency were detected among tissue types (bone marrow, spleen, peripheral blood, liver, and lymphoma node) in *Ncr1-Cre-Gnaq*^*fl/fl*^ mice and their littermate controls (Fig. 3d).

Finally, we evaluated whether *Gnaq* is involved in the regulation of NK cell expansion or survival. The expansion of splenic NK cells was assessed by an in vivo BrdU incorporation assay. No differences in DNA replication were observed in splenic NK cells between *Ncr1-Cre-Gnaq*^*fl/fl*^ mice and their littermate controls (Fig. 3e). We further investigated whether *Gnaq* regulates NK cell survival. Purified splenic NK cells were cultured in media without IL-2 for 6 h, and cell viability was monitored by Annexin V staining. Surprisingly, NK cells from *Ncr1-Cre-Gnaq*^*fl/fl*^ mice exhibited a significant survival advantage compared with NK cells from their corresponding littermate controls (Fig. 3f). Collectively, these data suggested that NK cells from mice lacking *Gnaq* have an intrinsic survival advantage over normal NK cells.

### Tumor-suppressive role of Gαq in NKTCL

**Fig. 4:**
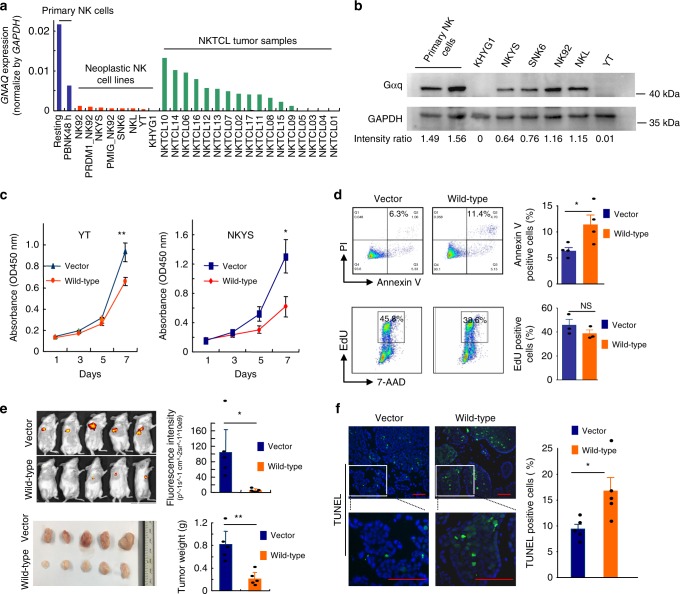
Tumor-suppressive role of Gαq in NKTCL. **a** *GNAQ* mRNA expression in normal NK cells, neoplastic NK cells, and tumor samples. The *GNAQ* expression values were obtained from previously published data and our RNA sequencing data and normalized to *GAPDH*. Resting NK: >95% CD56^+^CD3^−^ NK cells isolated from peripheral blood lymphocytes. PBNK48h: 48-h IL-2-activated NK cells. PMIG_NK92 and PRDM1_NK92: NK92 cells transduced with PMIG and PRDM1, respectively. **b** GNAQ protein expression in normal and neoplastic NK cells. **c** Forced expression of Gαq suppressed cell viability in YT (left) and NKYS (right) cells. YT and NKYS cells were stably transfected with vector control or wild-type *GNAQ*, and cell viability was assessed using a CCK-8 assay. Cell viability is presented by the absorbance value at OD 450 nm, which was measured with a Multiskan FC microplate reader (Thermo Scientific, Waltham, MA, USA). The value is directly proportional to the number of viable cells in the culture medium. Data are representative of three independent experiments. **d** Overexpression of Gαq significantly enhanced cell apoptosis but had little effect on NK cell proliferation. Cell apoptosis and cell proliferation were assessed by Annexin V staining (upper panel) and EdU incorporation assay (lower panel), respectively. Data are representative of at least three independent experiments. **e** NOD/SCID mice were inoculated subcutaneously with YT cells stably transfected with vector control or wild-type Gαq (*n* = 5 in each group). The tumor burden was monitored by utilizing the IVIS Spectrum system (Perkin Elmer, Beaconsfield, UK) after 6 weeks. Representative images and quantitative data for each group are shown in the upper panel. Representative images of xenograft tumors and tumor weights for each group are shown in the lower panel. **f** Representative images (left) and quantitative data (right) (*n* = 5 in each group) for TUNEL staining of the xenograft tumor tissues. Scale bars, 50 μm. All data are expressed as the mean ± s.e.m.; NS, not significant. *P < 0.05, **P < 0.01, ***P < 0.001, unpaired two-tailed Student’s *t*-test. Source data are provided as a Source Data file

We next sought to determine the functional role of Gαq in NKTCL. First, we examined the expression of *GNAQ* in NK cells isolated from normal and neoplastic tissues. A pooled analysis of previously published datasets^15^ and our own RNA sequencing data showed that *GNAQ* expression was significantly lower in neoplastic NK cell lines and tumor samples compared with primary human NK cells (Fig. 4a). This result was further confirmed by western blot analysis of Gαq protein expression in primary human NK cells and different neoplastic NK cell lines. In particular, loss of Gαq expression was observed in a novel NK leukemia cell line (KHYG1) (Fig. 4b). Chromosome 9q deletion has been reported in KHYG1, which may account for the loss of Gαq in this cell line^32,33^. Additionally, somatic copy number alteration analysis was performed in the 28 exome cases, and somatic copy number loss of *GNAQ* was found in one case (Supplementary Fig. 5).

Second, we generated stable cell lines (YT and NKYS) expressing vector control or *GNAQ*. Both cell lines carried wild-type *GNAQ* according to the RNA sequencing data^15^. As shown in Fig. 4c, forced expression of Gαq significantly suppressed tumor cell viability in comparison with the vector control in both cell lines. We further examined the effect of Gαq on cell apoptosis and proliferation by Annexin V staining and EdU incorporation assay, respectively. In vitro studies demonstrated that overexpression of Gαq significantly enhanced cell apoptosis but had little effect on NK cell proliferation (Fig. 4d).

Finally, YT cells stably expressing Gαq or vector control cells were injected subcutaneously into two groups of NOD/SCID mice (*n* = 5 each). The tumor size was measured every 2 days with calipers, and tumor burden was monitored 6 weeks after cell implantation. As expected, in vivo fluorescence imaging of xenograft mice showed that the fluorescence intensity (p^-1sec^-1cm^-2sr^-1) of tumors derived from the Gαq group was significantly lower than those from the control group (*P* = 0.011; Fig. 4e). Consistently, the mean tumor weight of the Gαq group at the end of the experiment was significantly lower (0.81 ± 0.24 g) than that of the control group (0.21 ± 0.12 g, *P* = 0.002; Fig. 4e). We further addressed the question of whether Gαq affected cell survival in vivo. The number of apoptotic cells was significantly higher in the Gαq group than in the vector control group, as determined by the TDT-mediated dUTP nick end labeling (TUNEL) assay (*P* = 0.031; Fig. 4f). In addition, the YT cells expressing Gαq were significantly less proliferative based on Ki67 staining and analysis of tumor sections (Supplementary Fig. 6). This finding indicated that overexpression of Gαq affects the survival and/or proliferative capacity of YT cells in the tumor microenvironment, which appears to be the mechanism by which Gαq can suppress tumor growth in vivo.

### Gαq T96S mutants act in a dominant negative manner in NKTCL

**Fig. 5:**
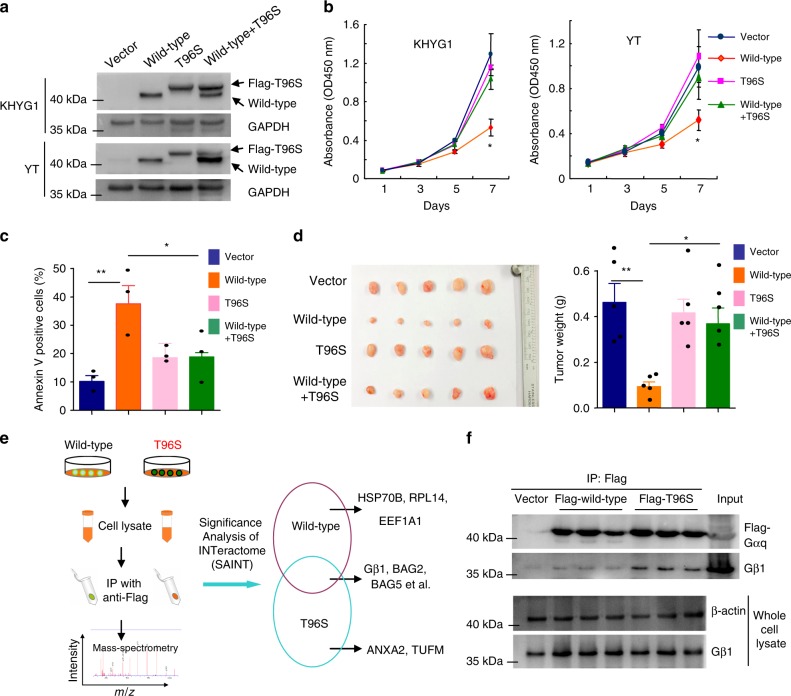
Gαq T96S mutant acts in a dominant negative manner to promote tumor growth of NKTCL. **a** Western blot shows that tetracycline-inducible cell lines (KHYG1 and YT) express equal amounts of wild-type and mutant Gαq simultaneously. **b** Viability of YT and KHYG1 cells with inducible expression of vector, wild-type, T96S or wild-type plus T96S. Cell viability is presented as the absorbance value at OD 450 nm. Data are representative of three independent experiments. **c** Apoptosis was induced after starving KHYG1 cells of IL-2 for 12 h and was assessed by Annexin V staining assay. Data are representative of three independent experiments. **d** Representative images of xenograft tumors (left) and tumor weights (right) for YT cells with inducible expression of vector, wild-type, T96S or wild-type plus T96S (*n* = 5 in each group). **e** Schematic diagram of the approach used to identify Gαq-interacting partners using immunoprecipitation in combination with mass spectrometry. Significance analysis of INTeractome (SAINT) expression was utilized to calculate the probability of protein–protein interactions from background, nonspecific interactions. The right panel shows representative binding proteins identified by mass spectrometry. **f** Confirmation of the interaction of wild-type Gαq or Gαq T96S mutant with Gβ1 by immunoprecipitation assay. The Gαq T96S mutant bound Gβ1 more tightly than wild-type Gαq in YT cells. All data are expressed as the mean ± s.e.m.; NS, not significant. *P < 0.05, **P < 0.01, ***P < 0.001, unpaired two-tailed Student’s *t*-test. Source data are provided as a Source Data file

We then attempted to characterize the functional role of the Gαq T96S mutant in NKTCL. Because all Gαq mutation-positive cases of NKTCL were heterozygous for this mutation, we established tetracycline-inducible cell lines (KHYG1 and YT) that could simultaneously express equal levels of wild-type and mutant Gαq. The expression levels of wild-type and Gαq T96S mutant were verified by western blot (Fig. 5a).

Expression of wild-type Gαq led to a significant decrease in the viability of NK cells. In contrast, the expression of the Gαq T96S mutant could compromise the ability of wild-type Gαq to impair NK cell viability (Fig. 5b). However, no significant difference between the Gαq T96S mutant and control groups was present, possibly due to the absent or extremely low expression of endogenous Gαq in these two NK cell lines. Similarly, forced expression of wild-type Gαq enhanced cell apoptosis of NK cells, and this proapoptotic effect of wild-type Gαq could be attenuated by the Gαq T96S mutant (Fig. 5c), suggesting a dominant negative nature of Gαq T96S. Moreover, we examined the effect of the Gαq T96S mutant on NKTCL tumorigenesis in vivo. Consistent with the above findings, wild-type Gαq significantly reduced the tumor-formation ability compared with the vector control, whereas the Gαq T96S mutant impaired the tumor-suppressor activity of wild-type Gαq. The mean weights of the tumors in the vector control, wild-type, Gαq T96S, and wild-type + Gαq T96S groups were (0.46 ± 0.08), (0.09 ± 0.02), (0.41 ± 0.06), and (0.37 ± 0.07) g, respectively (wild-type versus vector, *P* = 0.002; Gαq T96S versus vector, not significant; Fig. 5d).

Furthermore, we identified protein binding partners of wild-type or Gαq T96S mutants using immunoprecipitation coupled to mass spectrometry. Significance analysis of INTeractome (SAINT) expression was utilized to calculate the probability of protein–protein interaction from background, nonspecific interactions^34–37^. Using a SAINT probability threshold of ≥0.8, we identified 17 high-confidence interacting partners of Gαq (Fig. 5e and Supplementary Table 9). Gβ1 (GNB1), a known direct binding partner of Gαq, appeared to have higher affinity for the Gαq T96S mutant than its wild-type. Furthermore, immunoprecipitation assay confirmed that the Gαq T96S mutant bound Gβ1 more tightly than wild-type Gαq in YT cells (Fig. 5f). It is well known that the Gα mutant can exert a dominant negative effect in the heterotrimeric complex by sequestration of the Gβγ subunits^38^. It is therefore suggested that sequestering the Gβ subunits may be a possible mechanism by which the Gαq T96S mutant exerts its dominant negative effect on wild-type Gαq in NK cells, which should be further investigated. Altogether, these data suggested that the Gαq T96S mutant might contribute to the pathogenesis of NKTCL through the inhibition of wild-type Gαq in a dominant negative manner.

### Gαq suppresses AKT and MAPK signaling pathways in NK cells

**Fig. 6:**
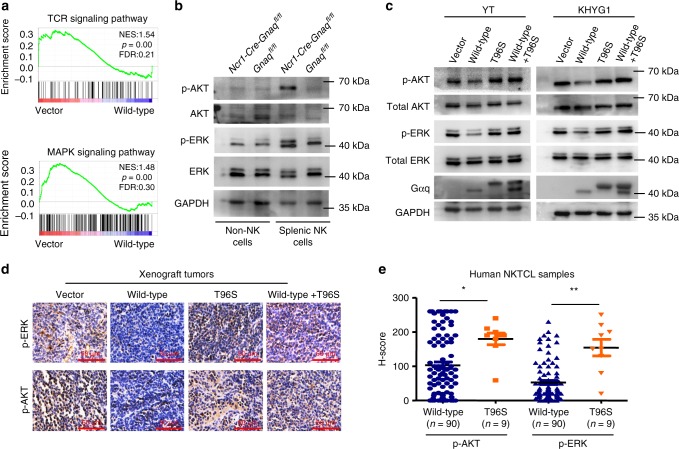
Gαq suppresses AKT and MAPK signaling pathways in NK cells. **a** RNA sequencing and subsequent gene set enrichment analysis (GSEA) were performed on NKYS cells stably expressing vector control or wild-type Gαq. The *P* value of GSEA was computed by a 1000-gene-set two-sided permutation test. **b** p-ERK and p-AKT expression in NK cells from *Ncr1-Cre-Gnaq*^*fl/fl*^ and *Gnaq*^*fl/fl*^ mice. **c** The effect of wild-type Gαq or T96S mutant on the activation of ERK and AKT in YT (left) and KHYG1 (right) cells. GAPDH was used as a loading control. **d** Representative immunostaining of p-ERK and p-AKT in the xenograft tumor tissues; scale bars, 50 μm. **e** Immunohistochemical study of p-ERK and p-AKT in human NKTCL samples with wild-type Gαq (*n* = 90) and T96S mutant (*n* = 9). The expression levels of p-ERK and p-AKT were scored using the semiquantitative *H*-score (histochemical score) visual approach, taking into consideration the intensity of staining and the percentage of stained cells. All data are expressed as the mean ± s.e.m.; NS, not significant. *P < 0.05, **P < 0.01, ***P < 0.001, unpaired two-tailed Student’s *t*-test. Source data are provided as a Source Data file

To examine the effect of Gαq on gene expression in NK cells, we performed RNA sequencing analysis using RNA prepared from NKYS cells stably expressing vector control or wild-type Gαq. Gene set enrichment analysis (GSEA) demonstrated that both MAPK and T cell receptor (TCR) signaling pathways were significantly enriched in cells expressing the vector control compared with wild-type Gαq cells (Fig. 6a and Supplementary Table 10). Consistently, NK cells from *Ncr1-Cre-GNAQ*^*fl/fl*^ mice showed significantly higher expression of phospho-ERK (p-ERK) and phospho-AKT (p-AKT) than their control littermates (Fig. 6b). These results indicated that Gαq could suppress AKT and MAPK signaling pathways in NK cells, consistent with previous studies reporting that these two pathways could be suppressed by Gαq in mice B and T cells^39,40^.

These findings were further validated in NKTCL cells. As shown in Fig. 6c, compared with the vector control group, the phosphorylation of ERK and AKT was suppressed in the presence of wild-type Gαq. However, unlike wild-type Gαq, the Gαq T96S mutant was unable to reduce the phosphorylation of ERK and AKT. In contrast, it could affect the suppressive function of wild-type Gαq on the two signaling pathways, underlining a dominant negative nature of Gαq T96S. Similar results were obtained from immunohistochemistry analysis of mouse xenograft tumors (Fig. 6d). Moreover, phospho-ERK and phospho-AKT were detected in human NKTCL samples, with significantly higher levels in patients with the mutant *GNAQ* T96S genotype than in patients with the wild-type Gαq genotype using two immunohistochemistry scoring methods (Fig. 6e and Supplementary Fig. 7).

### *GNAQ* T96S mutations predict a worse prognosis

We investigated the relationship between *GNAQ* T96S mutations and clinical characteristics. In this analysis, only patients with matched tumor/normal sample pairs were included, two individuals with non-T96S *GNAQ* mutations (Y101X in #8188 and K57T in #36) were excluded from the survival analysis. We found that *GNAQ* T96S mutations were significantly associated with advanced disease stage (*P* = 0.025) and a higher fraction of Ki67-positive cells (>50%) in the tumor samples (*P* = 0.035; Supplementary Table 11).

**Fig. 7:**
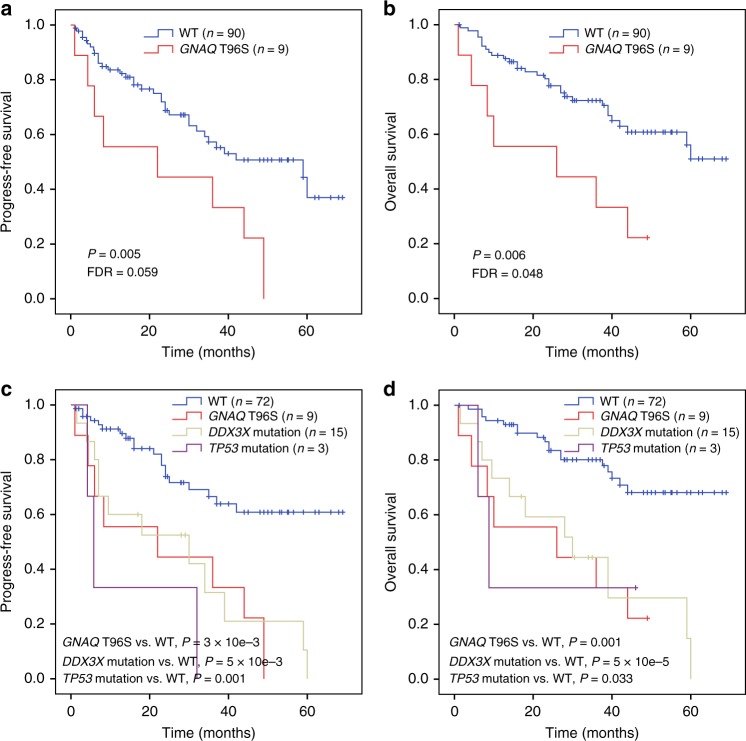
*GNAQ* T96S mutations predict a worse prognosis in NKTCL. **a**, **b** Progression-free survival (**a**) and overall survival (**b**) of NKTCL patients with *GNAQ* T96S mutations (*n* = 9) or wild-type (WT, *n* = 90). **c**, **d** Progression-free survival (**c**) and overall survival (**d**) of patients according to the mutation status of *DDX3X*, *GNAQ*, and *TP53*. Patients were divided into four groups: those with *GNAQ* T96S mutations (*n* = 9), *DDX3X* mutations (*n* = 15), or *TP53* mutations (*n* = 3) and those without mutations in any of these three genes (WT, *n* = 72). Two individuals with both *DDX3X* and *TP53* mutations were grouped into the *DDX3X* cohort. One individual with both *GNAQ* T96S and *TP53* mutations was grouped into the *GNAQ* T96S cohort. Survival curves were estimated with the Kaplan–Meier method and compared using a two-sided log-rank test

Univariate analysis suggested that patients with *GNAQ* T96S mutations (*n* = 9) had a worse prognosis than individuals with wild-type *GNAQ* (*n* = 90). The estimated 3-year OS rates of patients with *GNAQ* T96S mutations and wild-type *GNAQ* were 33.3 ± 15.7% and 72.3 ± 5.0%, respectively (*P* = 0.006, FDR = 0.048; Fig. 7a). The estimated 3-year progression-free survival (PFS) rates for subjects from the *GNAQ* T96S and wild-type *GNAQ* groups were 22.2 ± 13.9% and 57.3 ± 6.2%, respectively (*P* = 0.005, FDR = 0.059; Fig. 7b). Moreover, when a Cox multivariate regression model was applied, the T96S mutation status of *GNAQ* remained an independent prognostic marker for poor outcomes after adjusting for B symptoms, the International Prognostic Index (IPI), the Ki67 index (>50%), and the primary site of the tumor (OS: HR = 3.31, CI = 1.33–8.24, *P* *=* 0.010; and PFS: HR = 2.85, CI = 1.27–6.37, *P* = 0.011; Supplementary Table 12). Altogether, these results suggested that *GNAQ* T96S mutations were positively correlated with advanced tumor stage and poor clinical outcomes in NKTCL.

In addition, mutations in *DDX3X* or *TP53* were associated with a worse prognosis of patients with NKTCL, consistent with previous studies^41^. In contrast, patients without mutations in *DDX3X*, *TP53*, or *GNAQ* (T96S) showed a more favorable prognosis than those harboring the mutations (Fig. 7c, d). These observations indicated that the mutational status of *DDX3X*, *TP53* and *GNAQ* genes might represent a promising marker for therapeutic stratification of patients with NKTCL, which needs to be validated in future large, prospective studies.

## Discussion

NKTCL is a rare, malignant neoplasm characterized by a highly aggressive clinical course^13,42–45^. Although considerable advances in our understanding of the mechanisms involved in NKTCL have been made in recent years, the rare nature of NKTCL and its heterogeneity limit the ability to standardize therapy^13,42–46^. Somatic mutation analysis represents a useful tool in selecting personalized therapy^14,22,47^. In this study, we undertook large-scale screening in a cohort of NKTCL patients and identified recurrent somatic *GNAQ* mutations encoding a p. T96S alteration in 8.7% (11/127) of NKTCL samples. In addition to *GNAQ*, mutations in multiple genes involved in G protein-coupled receptor (GPCR) signaling pathways, including *GPR98*, *GPR125*, *GNAS*, *GNA12*, *GNAI2*, and *PLCB3*, were also detected in our study. These mutations appeared to be mutually exclusive and affected 24.8% (25/101) of total NKTCL cases (Supplementary Fig. 8), suggesting that deregulated GPCR signaling plays a major role in the tumorigenesis of NKTCL.

Gαq is a member of the q class of Gα subunits that mediates signals between GPCRs and downstream effectors^48,49^. Although the oncogenic potential of Gαq has been widely studied in human tumors, its roles in NK cells remain unclear^49^. Using newly generated conditional knockout mice (*Ncr1-Cre-GNAQ*^*fl/fl*^), we found that NK cells lacking *GNAQ* have an intrinsic survival advantage over normal counterparts, which is consistent with previous studies in B and T lymphocytes^39,40^. Further functional studies in neoplastic NK cells revealed a tumor-suppressive role of Gαq in NKTCL. Gαq suppressed the activation of AKT and ERK in the same way it did in B and T lymphocytes^39,40^. Therefore, the dual function of Gαq in different types of tumors suggests that it may potentially act either as a tumor suppressor or as an oncogene depending on the cellular context. This type of functional duality based on cellular context has been observed for Gα13 (encoded by *GNA13*), which is another member of the Gα protein family^49,50^. Gα13 has been previously linked to cellular transformation and characterized as growth promoting in epithelial cancer models^51,52^. However, recent studies have identified a Gα13-dependent pathway that exerts dual actions in suppressing growth and blocking dissemination of germinal center B cells, which are frequently disrupted in germinal center B-cell-derived lymphoma^53–55^.

According to the COSMIC database^29^, there are four mutational hotspots in *GNAQ*: Q209, R183, T96 and Y101. The frequency of these mutation hotspots within *GNAQ* appears to exhibit significant differences between solid and hematopoietic tumors. Q209 mutations comprise the most frequently mutated hotspot in solid tumors, followed by R183 mutations. In striking contrast, T96 is the predominantly mutated hotspot in hematopoietic tumors, followed by Y101 (Supplementary Fig. 9). This pattern was also verified in NKTCL patients in the present study. This work thus suggests the intriguing possibility that different *GNAQ* mutations may have different functional consequences, and the properties crucial for their oncogenic functions vary depending on the tissue of origin.

However, this study has some limitations that must be noted. First, the Y101X mutation harbors a premature termination codon. In contrast to T96S, cell line transduction experiments showed that the Y101X mutation could disrupt truncated Gαq protein expression (Supplementary Fig. 10). We speculated that instability of truncated Gαq protein or nonsense-mediated mRNA decay might account for the disruption of truncated Gαq protein expression, which needs to be further investigated. We did not conduct functional analysis of the non-hot spot mutants (K57T and M59L) in the present study, given that these two mutants occurred in only 1% of the NKTCL cases. However, the functions of the two mutants need to be addressed in future studies. Second, germline DNA was unavailable in 26 specimens among the 127 NKTCL cases. Although a strict filter was applied for variant calling, the somatic nature of these mutations identified in this cohort could not be confirmed. We therefore excluded these cases in the subsequent survival analysis. Third, further studies are warranted to clarify the molecular mechanisms mediated by Gαq in suppressing AKT and MAPK signaling in NK cells.

Taken together, we identified recurrent somatic GNAQ T96S mutations, which may contribute to the pathogenesis of NKTCL. These findings have implications for refining our understanding of the genetic mechanisms of NKTCL and for treatment development.

## Methods

### Patients and samples

The demographics and clinical features of the patient cohort are summarized in Supplementary Table 1. In total, 127 formalin-fixed, paraffin-embedded (FFPE) tumor tissues from NKTCL patients were obtained at the time of diagnosis at the First Affiliated Hospital of Zhengzhou University. All cases were reviewed and interpreted independently by three experienced pathologists, and diagnoses were made according to the current World Health Organization classification criteria. The experimental design is depicted in Supplementary Fig. 1. The study was conducted in accordance with the Declaration of Helsinki and with approval from the Institutional Review Board of the First Affiliated Hospital of Zhengzhou University. Signed informed consent was obtained from these patients.

### DNA extraction

Genomic DNA from whole blood and FFPE samples was extracted using a Blood & Cell Culture DNA Kit (Qiagen, Hilden, Germany) and a QIAamp DNA FFPE Tissue Kit (Qiagen), respectively. The quality and yield of purified DNA were assessed by fluorimetry (Qubit, Invitrogen), Nanodrop 1000 spectrophotometry (Thermo Scientific, Wilmington, DE, USA), and gel electrophoresis.

### Whole-exome sequencing and bioinformatics analysis

To identify somatic genomic variants associated with NKTCL, we performed WES on 28 tumor samples and their matched whole-blood samples. Genomic DNA (1–1.5 µg) was fragmented with a Covaris ultrasonicator targeting peak sizes ranging from 180 to 280 bp. The fragment ends were blunted and 5′-phosphorylated with T4 polynucleotide kinase, T4 DNA polymerase, and Klenow Large Fragment (New England BioLabs). The 3′ ends were A-tailed using Klenow Exo-Minus (New England BioLabs), and the fragments were ligated to Illumina paired-end adapters. Ligation products were purified with Agencourt AMPure XP beads and enriched by PCR using the Illumina PCR primers InPE1.0 and InPE2.0 and PCR primer indices. Pooled, indexed libraries were captured using the Agilent SureSelect Human All Exon 50 Mb kit (Agilent Technologies) according to the manufacturer’s protocol and sequenced on an Illumina HiSeq 2500 instrument.

Sequencing reads were aligned to the human reference genome (hg19, downloaded from the UCSC Genome Browser http://genome.ucsc.edu/) using the Burrows-Wheeler Aligner (BWA)^56^ version 0.5.8 with default parameter settings. SAMtools was used to convert the SAM files into BAM files and to pile up sequences after local alignment^57^. The Picard command (http://sourceforge.net/projects/picard/files/picard-tools/) was used to remove PCR duplications. Next, insertion or deletion (InDel) realignment and base quality score recalibration were performed with the Genome Analysis Toolkit (GATK) version 2.6.5^58^. For tumor–normal tissue sample pairs, somatic single-nucleotide variations (SNVs) and somatic InDels were called using MuTect^59^ and Strelka^60^, respectively. Control-FREEC was used to detect copy number alterations in tumors compared with normal cells^61^. Somatic copy number alteration (Supplementary Table 14) and loss of heterozygosity (LOH, Supplementary Table 15) analyses were performed in the 28 exome cases. For the 26 non-paired tumor samples with no matched germline DNA, probable somatic variants were detected by SomVarIUS with the default settings^62^. Only variants from the 26 non-paired tumor samples were further filtered to remove those that were present in dbSNP v135 (http://www.ncbi.nlm.nih.gov/SNP/), the 1000 Genomes Project (http://browser.1000genomes.org), or an in-house database containing germline variants identified in ~500 Chinese exomes. Variants were also visually inspected with the Integrative Genomics Viewer (IGV, https://www.broadinstitute.org/igv/) to exclude probable sequencing artifacts. Gene mutation annotation of the identified variants was carried out using ANNOVAR^63^. The impact of the SNVs on protein function was predicted by PolyPhen-2, SIFT, or MutationTaster. Whole-exome sequencing data were deposited into the NCBI Sequence Read Archive under accession code SRP107053.

### Targeted deep sequencing

We designed a custom panel of 221 genes (Supplementary Table 5) using the SureDesign Tool (Agilent Technologies). Sequencing libraries were prepared from DNA extracted from 73 paired tumor and normal samples using the SureSelect XT2 Target Enrichment System for the Illumina Multiplexed Sequencing Platform (Illumina) according to the manufacturer’s instructions. Target-enriched libraries were then sequenced on an Illumina HiSeq 2500 sequencing platform. A bioinformatics analysis was performed as described for the exome sequencing analysis. All candidate variants were manually inspected in the IGV to exclude false positives.

### Sanger sequencing

Selected SNVs detected by whole-exome sequencing were validated by Sanger sequencing. Primers specific to the regions of interest were designed and synthesized by Sangon Biotech (Shanghai, China, Supplementary Table 4). PCR was performed using standard procedures followed by direct sequencing on an ABI 3730xl automatic sequencer (PE Applied Biosystems, Foster City, CA, USA).

### Mice and genotyping

*Ncr1-Cre-Gnaq*^*fl/fl*^ mice (C57BL/6) were generated by crossing *Gnaq*^*fl/fl*^ mice with *Ncr1-Cre* mice. *Gnaq*^*fl/fl*^ mice were generated by Viewsolid Biotech (Beijing, China). Briefly, *loxP* sites flanking exon 6 of *Gnaq* were introduced by CRISPR/Cas9. This locus of *Gnaq* has been previously successfully used to generate conditional *Gnaq*-deficient mice^64^. The in vitro-synthesized sgRNA, Cas9 mRNA, and donor vectors were injected into mouse zygotes and then transferred into pseudopregnant mice. Neonatal mutant mice were identified by genotyping and sequencing. Genomic DNA samples were prepared from tail biopsies of 5-day-old mice. PCR genotyping was performed. The primer pairs used to identify correct insertion of LoxP flanking exon 6 of *Gnaq* and *Cre* are provided in Supplementary Table 13. The relevant Animal Ethics and Experimentation Committees approved animal experiments according to the guidelines of the First Affiliated Hospital of Zhengzhou University. Both female and male mice aged between 8 and 15 weeks of age were used in this study. Age and sex matching was performed for each independent experiment (*n* ≥ 4 mice per genotype). All procedures were conducted in accordance with the Animal Care and Use Committee guidelines of Zhengzhou University.

To isolate cells from the spleen, spleens were minced and treated with 2 mg/ml collagenase D in Hanks balanced salt solution with CaCl_2_/MgCl_2_ for 30 min at room temperature followed by filtering through 70-μm nylon cell strainers. The livers of mice were perfused with PBS to remove blood for the isolation of liver-resident cells, and single-cell suspensions were subsequently generated as described for the spleen. Single-cell suspensions from lymph nodes were generated by meshing the organs through 70-μm nylon cell strainers. Contaminating erythrocytes in cell suspensions were lysed using ACK lysis buffer (Thermo Scientific, Waltham, MA, USA) or removed by density-gradient centrifugation through Ficoll/Hypaque (Sigma-Aldrich, St Louis, MO, USA), and the remaining cells were washed and subjected to subsequent analysis and functional assays. Purified NK cells were obtained by negative selection using NK Cell Isolation kits (Miltenyi Biotec). The purity as assessed by flow cytometry was 90–95%.

For in vivo bromodeoxyuridine (BrdU) incorporation assays, mice were intraperitoneally injected with 2 mg of BrdU (in 200 μl). After 12 h, splenocytes were isolated, stained, fixed, permeabilized, and treated with DNase. Analysis of BrdU incorporation was performed using a BrdU Flow Kit (BD Pharmingen). Survival was assessed by culturing freshly isolated splenic NK cells for 6 h at a density of 3 × 10^4^ cells per well in 100 μl of RPMI 1640 medium plus FBS (without IL-2) in 96-well plates. Live and dead cells were discriminated by staining with propidium iodide and Annexin V–FITC (BD Pharmingen). Stained samples were analyzed using FACSCantoII and FACSDiva software Version 6.1.2 (BD Biosciences).

### Cell culture

Three NK cell lines (YT^65^, KHYG1^32^, and NKYS^66^) were obtained from Dr. Wing C. Chan (City of Hope Medical Center). The NK92^67^ cell line was purchased from the American Type Culture Collection (ATCC), and the NKL^68^ cell line was purchased from the cell bank of the Bena Culture Collection (Beijing, China). The SNK6^69^ cell line was kindly provided by Dr. Norio Shimizu and Yu Zhang of Chiba University. Cells were cultured in RPMI 1640 medium (Invitrogen, California, USA) supplemented with 10% heat-inactivated FBS (Sigma-Aldrich, St Louis, MO, USA), 2 mM glutamine, 100 U/ml penicillin, and 100 µg/ml streptomycin (Invitrogen, California, USA) in 5% CO_2_ atmosphere at 37 °C. In addition, all cell lines except the YT line were interleukin-2 (150 IU/ml, PeproTech, Rocky Hill, NJ, USA)-dependent. The identity of these cell lines was confirmed by short tandem repeat (STR) profiling or high-throughput sequencing, and they both tested negative for mycoplasma contamination using a Lonza MycoAlert Mycoplasma Detection Kit.

### Mutagenesis and constructs

Full-length human *GNAQ* cDNA was purchased from Sino Biological Inc. (Beijing, China). Mutagenesis to create constructs encoding the T96S or Y101X mutants was carried out using a Quick-Change Site-Directed Mutagenesis Kit (Agilent Technologies, Santa Clara, CA) according to the manufacturer’s instructions. These constructs encoding wild-type and mutant *GNAQ* were subcloned into the lentivirus-based expression vector pCDH-CMV-MCS-EF1-copGFP (System Biosciences, #CD511B-1). In addition, 3×Flag-tagged *GNAQ* T96S was subcloned into the tetracycline-inducible pLVX-TetOne-Puro vector (Clontech, Takara, #631849). All cDNA sequences were confirmed by Sanger sequencing.

### Lentivirus production and generation of stable cell lines

For overexpression of wild-type GNAQ and mutants, 12 µg of purified plasmids pCDH-CMV-MCS-EF1-copGFP-vector, pCDH-CMV-MCS-EF1-copGFP-wild-type, pCDH-CMV-MCS-EF1-copGFP-T96S, or pCDH-CMV-MCS-EF1-copGFP-Y101X was cotransfected with 8 µg of psPAX2 packaging plasmid and 4 µg of pMD2.G envelope plasmid into HEK293T cells using Lipofectamine 2000 (Invitrogen) according to the manufacturer’s protocol. The viruses were harvested 72 h posttransfection, and cells were infected with these viruses in the presence of 8 mg/ml polybrene. Infected cells expressing GFP were sorted using a flow cytometer (FACSCantoII, BD Bioscience, San Jose, CA, USA), and the purity of the sorted cell fractions consistently exceeded 95%.

To establish cell lines stably expressing equal amounts of wild-type and T96S simultaneously, KHYG1 and YT cells were infected with pCDH-CMV-MCS-EF1-copGFP-vector or pCDH-CMV-MCS-EF1-copGFP-wild-type lentivirus. The stably transfected clones were selected by GFP. These cells were subsequently infected with pLVX-TetOne-Puro-vector or pLVX-TetOne-Puro-3×Flag-T96S lentivirus and selected with 4 µg/ml puromycin (Sigma Chemical Co., St Louis, MO, USA) to establish tetracycline-inducible cell lines. The tetracycline-inducible cell lines were maintained in RPMI 1640 medium with 0.25 µg/ml puromycin, and the cells were incubated with 20 ng/ml doxycycline (Sigma-Aldrich, Shanghai, China) for up to 48 h to induce T96S mutant expression.

### Cell viability assay

Cells were seeded in 96-well plates in triplicate at a density of 5 × 10^3^ cells/well in 100 μl of cell culture medium. Cell viability was evaluated using a Cell Counting Kit-8 (CCK-8, Dojindo, Tokyo, Japan) at the indicated days according to the manufacturer’s instructions. In brief, 10 µl of CCK-8 reagent was added to each well. The cells were incubated at 37 °C in an atmosphere containing 5% CO_2_ for 2 h. Cell viability was presented by the absorbance value at OD 450 nm, which was measured with a Multiskan FC microplate reader (Thermo Scientific, Waltham, MA, USA). The value is directly proportional to the number of viable cells in the culture medium. Independent experiments were repeated at least three times.

### Flow cytometric analysis

Cell proliferation was assessed by BrdU incorporation assays with a BrdU Flow Kit (BD Pharmingen) or an EdU Click Proliferation Kit (BD Pharmingen). Cell apoptosis was analyzed with a PE-Annexin V Kit (BD Pharmingen). The following antibodies were purchased from BD Biosciences: CD122-PE (TM-Bta1), CD19-FITC (1D3), CD335/NKp46-PE (29A1.4), CD3e-FITC (145-2C11), CD3e-PerCP-Cy5.5 (145-2C11), CD4-FITC (RM4-5), CD49b (DX5) Pan-NK Cells-BV421, CD8a-PE (53-6.7), Erythroid Cells-FITC (TER-119), Ly-6G/Ly-6C-FITC (RB6-8C5), NK-1.1-APC (PK136), NK-1.1-APC-Cy7 (PK136), and TCR-Bta Chain-BV421 (H57-597). Stained samples were analyzed using FACSCantoII and FACSDiva software Version 6.1.2 (BD Biosciences).

### Xenograft tumor assay

Female NOD/SCID (4–5 weeks old, No. 11400700201639) mice were purchased from Vital River Laboratories (Beijing, China). The mice were housed five/cage in microisolator units under humidity- and temperature-controlled conditions with 12-h light–dark cycles. The animals were fed a standard sterile laboratory diet (Zhengzhou University, Zhengzhou, China) beginning at least 3 days before use. Animal health was examined prior to tumor implantation and randomization to ensure that only animals without any symptoms of disease were selected to begin experimental procedures. During the experiments, animals were monitored twice daily regarding tumor burden, general conditions, and food and water supply. The mice were randomly distributed into four groups and subcutaneously injected with 5.0 × 10^6^ cells in 0.1 ml of PBS/Matrigel (BD Biosciences, CA, USA) mixture (1:1 volume) into the flank regions. These four groups were tetracycline-inducible YT cells stably transfected with vector control, wild-type, T96S, and wild-type + T96S (*n* = 5 per group). When the needle was inserted under the skin, it was held parallel to the animal's body to avoid puncturing underlying structures, and the syringe was aspirated to ensure that the needle tip had not penetrated a blood vessel. Doxycycline was administered as a 2 mg/ml concentration in drinking water supplemented with 5% sucrose (Sigma-Aldrich, Shanghai, China). Because doxycycline has limited stability in water, the supply was changed every other day. The tumor size was measured every 2 days with calipers, and the tumor burden was monitored after 6 weeks of implantation by utilizing the IVIS Spectrum system (Perkin Elmer, Beaconsfield, UK). The mice were then euthanized by asphyxiation in a CO_2_ chamber, and the tumors were excised using standard forceps, scissors, and surgical blades. For histological analysis of xenograft tumors, mouse samples were blinded during sample processing and just identified by their mouse ID numbers. All procedures were conducted in accordance with the Animal Care and Use Committee guidelines of Zhengzhou University.

### TUNEL assay

Xenograft tumors were fixed in 10% PBS-buffered formalin and embedded in paraffin. The apoptotic cells were determined by TUNEL staining using a One Step TUNEL Apoptosis Assay Kit (Beyotime, Jiangsu, China), and the nuclei were stained with DAPI. Images were captured using a fluorescence microscope (DMI400B, Leica Company, Germany).

### RNA sequencing and analysis

RNA isolation, library construction, and sequencing were performed by the Novogene Bioinformatics Institute (Beijing, China). Total RNAs were extracted using the RNeasy Mini kit (Qiagen) according to the manufacturer’s protocol. The quality and yield of the RNA were assessed by fluorimetry (Qubit, Invitrogen), Nanodrop 1000 spectrophotometry (Thermo Scientific, Wilmington, DE, USA), and gel electrophoresis. RNA integrity was assessed using the RNA Nano 6000 Assay kit with the Bioanalyzer 2100 system (Agilent Technologies, CA, USA). A total of 3 μg of RNA from each sample was used as input material for RNA sample preparation. First, ribosomal RNA was removed using an rRNA Removal kit (Epicentre, WI, USA). Second, sequencing libraries were generated using the Illumina TruSeq RNA Sample Preparation kit (Illumina, CA, USA) according to the manufacturer’s recommendations. To select cDNA fragments between 150 and 200 bp in length, the library fragments were purified with the AMPure XP system (Beckman Coulter, Beverly, USA). Fragments were then amplified by ten cycles of PCR using Phusion DNA polymerase, and libraries were validated with the Bioanalyzer 2100 system (Agilent Technologies, CA, USA). Lastly, the libraries were applied to an Illumina flow cell using the Illumina Cluster Station. After cluster generation, the libraries were sequenced on an Illumina Hiseq 2000 platform, and 100-bp paired-end reads were generated. Bowtie (v2.2.3) was used to build the reference genome index, and TopHat (v2.0.12) was used to align the clean paired-end reads to the reference genome. The read numbers mapped to each gene were counted by HTSeq (v0.6.1), and the expected number of fragments per kilobase of transcript sequence per million base pairs sequenced (FPKM) was calculated. GSEA (version 2.2.4) and gene set collection of KEGG pathways were used for enrichment analysis. The statistical significance of signature enrichment was assessed using 1000-gene-set permutations. The raw sequencing data are available from the NCBI and are archived under the accession number SRP180943.

### Immunoprecipitation

The immunoprecipitation assay was performed using the Pierce™ Classic Magnetic IP/Co-IP kit (Thermo Scientific, Waltham, MA, USA) according to the manufacturer’s protocol. Briefly, cells (1.0 × 10^7^) were lysed in cold Pierce IP Lysis buffer and Halt™ Protease and Phosphatase Inhibitor cocktail (Thermo Scientific, Waltham, MA, USA) for 15 min on ice. Cell debris was removed by centrifugation at 13,000×*g* for 10 min. Cell lysate (1000 µg) was combined with 10 μg of Flag antibody (Thermo Scientific, Waltham, MA, USA) and incubated overnight at 4 °C with rotation. The antigen sample/antibody mixture was added to the tube containing prewashed magnetic beads (50 µl) and incubated at room temperature for 1 h with mixing. The beads were collected with a magnetic stand and washed twice with IP lysis/wash buffer and once with purified water; the antigen/antibody complex was then eluted.

### Mass spectrometry analysis

The eluted proteins were digested according to the SISPROT protocol, as described in a previous study^70^. The desalted peptides were lyophilized under vacuum and kept at –20 °C until mass spectrometry analysis.

The desalted sample was dissolved in solvent A and analyzed via reverse-phase high-pressure liquid chromatography electrospray ionization tandem mass spectrometry (RP-HPLC-ESI-MS/MS) using a TripleTOF 5600+ mass spectrometer (SCIEX, Concord, Ontario, Canada) coupled to a NanoSpray III ion source and a nanoLC Eksigent 415 system (SCIEX, Concord, Ontario, Canada). Nano-scale reverse-phase liquid chromatography (RPLC) was performed with a trap-and-elution configuration using a Nano cHiPLC Trap column (200 μm × 0.5 mm ChromXP C18-CL 3 μm 120 Å) and a nano-scale analytical column (75 μm × 15 cm ChromXP C18-CL 3 μm 120 Å). Solvents A and B were 2% and 98% acetonitrile in water supplemented with 0.1% formic acid. The sample was loaded in the trap column at a flow rate of 3 μl/min for 7 min using 100% solvent A. For each sample, a stepwise gradient of 60 min (0–0.5 min, 95–92% A; 0.5–38 min, 92–75% A; 38–44 min, 75–65% A; 44–45 min, 65–20% A; 45–51 min, 20% A; 51–52 min, 20–95% A; 52–60 min, 95% A) or 90 min (0–0.5 min, 95–93% A; 0.5–55 min, 93–75% A; 55–70 min, 75–65% A; 70–75 min, 65–50% A; 75–76 min, 50–20% A; 76–81 min, 20% A; 81–82 min, 20–95% A; 82–90 min, 95% A) was separately performed using a flow rate of 300 μl/min.

The following parameters were used with the mass spectrometer: MS survey scan range, 350–1500 *m*/*z*; MS survey, 0.25 s; MS/MS scan range, 100–1500 *m*/*z*. The precursor ions were fragmented in the collision cell using rolling collision energy, and the collision energy spread (CES) was set to 10. Up to 30 precursor ions were selected for subsequent MS/MS experiments with an accumulation time of 0.07 s per MS/MS scan and a total cycle time of 2.4 s. The selection criteria for precursor ions included an intensity greater than 200 cps and a charge state ranging from +2 to +5. The precursor ions were fragmented in the collision cell using rolling collision energy, and the CES was set to 5. The following parameters were used: dynamic excluded time, 18 s; ignore peaks within 6 Da; exclude after occurrences, 1; mass tolerance, 50 ppm.

The original .wiff files were converted to .mgf files using PeakView software (SCIEX, Concord, Ontario, Canada), and the resulting files were subjected to database searches using Mascot 2.3 (Matrix Science, London, United Kingdom). The following parameters were used for database search: trypsin, KR/P; max cleavage sites, 2; fixed modification, carboxamidomethylation (C); variable modification, oxidation (M), deamidated (NQ), acetyl (N-term); mass tolerance, 0.05 Da for the precursor ion and 0.1 Da for the fragment ion. The searches were conducted in a UniProt Swiss-Prot database (downloaded in May 2017, with 20201 reviewed and canonical protein sequence entries) containing whole *Homo sapiens* proteins and the same number of reversed protein sequences. Three independent IP-Mass experiments were performed. SAINT express (v3.6.1) was the statistical tool utilized to calculate the probability of protein–protein interaction from background, nonspecific interactions with a SAINT probability threshold of ≥0.8 (ref. ^34–37^).

### Western blot analysis

Cells were lysed in cold RIPA lysis buffer and Halt™ Protease and Phosphatase Inhibitor cocktail (Thermo Scientific, Waltham, MA, USA) for 20 min on ice. The cell lysates were clarified by centrifugation at 10,000×*g* for 20 min. Proteins (10–25 μg) were resolved by SDS-PAGE and transferred onto nitrocellulose membranes (Amersham Biosciences, Piscataway, NJ, USA). The membranes were blocked in TBS-T buffer (20 mM Tris-HCl, pH 7.5, 150 mM NaCl, and 0.05% Tween-20) containing 5% (w/v) non-fat milk at room temperature for 1 h and then probed at 4 °C overnight with antibodies to detect AKT (pan) (clone C67E7, 1:1000), phospho-AKT (Ser473) (clone D9E, 1:1000), phospho-p44/42 MAPK (ERK1/2) (Thr202/Tyr204) (clone D13.14.4E, 1:1000), p44/42 MAPK (ERK1/2) (clone 137F5, 1:2000) from Cell Signaling Technology (Boston, MA, USA); Gαq (clone ab75825 and ab190082, 1:1000) from Abcam (Cambridge, USA); DYKDDDDK Tag Monoclonal Antibody (FG4R, MA1-91878, 1:1000) from Thermo Scientific (Waltham, MA, USA), and GAPDH (clone 60004-1-Ig, 1:2000) from ProteinTech (Chicago, IL, USA). Detection was carried out with the SuperSignal West Femto Maximum Sensitivity Substrate Trial kit (Pierce, Rockford, IL, USA). The band images were digitally captured and quantified with a ChemiDoc™ XRS+ system (Bio-Rad Laboratories, Hercules, CA, USA).

### Immunohistochemistry

Immunohistochemistry was performed as described previously^71^. The FFPE sections were immunostained using the Dako EnVision™ Flex+ System (K8012; Dako, Glostrup, Denmark). Deparaffinization and epitope unmasking were carried out in a PT Link instrument using an EnVision™ Flex target retrieval solution (Dako, Carpinteria, CA, USA). The sections were treated with 0.3% hydrogen peroxide (H_2_O_2_) for 5 min to block endogenous peroxidase. Sections were incubated overnight at 4 °C with the following antibodies: phospho-p44/42 MAPK (ERK1/2) (Thr202/Tyr204) (clone D13.14.4E; diluted 1:100; Cell Signaling Technology, Beverly, MA), phospho-AKT (Ser473) (clone D9E; diluted 1:100; Cell Signaling Technology, Beverly, MA), Gαq (clone ab75825; diluted 1:50; Abcam), and Ki67 (clone MIB-1; diluted 1:50; Dako). The specimens were subsequently treated with EnVision™ Flex mouse or rabbit linker (15 min), EnVision™ Flex/HRP enzyme (30 min), and 3′3-diaminobenzidine tetrahydrochloride (10 min). The samples were counterstained with hematoxylin, dehydrated, and mounted with Richard-Allan Scientific Cyto seal XYL mounting medium (Thermo Scientific, Waltham, MA, USA). The sample series included appropriate positive and negative controls. All cases were scored by the pathologist without prior knowledge of the patients’ mutational status and outcomes. For histological analysis of xenograft tumors, mouse samples were blinded during sample processing and just identified by their mouse ID numbers. Expression levels of p-ERK and p-AKT were scored using the semi-quantitative *H*-score (Histochemical score) visual approach, taking into consideration the intensity of staining and the percentage of stained cells^72^. Staining intensity was scored as 0, 1, 2, or 3 for negative, weak, moderate, and strong, respectively. The percentage of positive cells for each intensity was subjectively estimated. *H*-score ranged from 0 (no staining) to 300 (maximum staining) and was calculated by application of the following formula: *H* score = 1 × (% light staining) + 2 × (% moderate staining) + 3 × (% strong staining). Alternatively, expression levels of p-ERK and p-AKT were scored semiquantitatively according to the percentage of IHC-positive cell: +, 0; ++, 1–10%; +++, 11–50%; ++++, >50%. All cases were scored by the pathologist without prior knowledge of the patients’ mutational status of Gαq. The relationship between the expression of p-ERK (or p-AKT) and Gαq mutational status were evaluated by Fisher’s exact test, 2 × 4 table (www.vassarstats.net).

### Statistical analysis

All data are expressed as the mean ± s.e.m. Comparisons between and among groups were performed with unpaired two-tailed Student’s *t*-test and analysis of variance (ANOVA), respectively. The association of *GNAQ* mutations and clinicopathological characteristics was analyzed by the *χ*^2^ test or Fisher’s two-tailed exact test. Analyses were conducted to evaluate the relationship between gene mutations and prognosis, and only those frequently mutated genes (mutated in at least 5% of patients) were included. OS rates and PFS were calculated using the Kaplan–Meier method, and significance was assessed by the log-rank test. Derived *P* values were adjusted for false discovery rate (FDR) using the Benjamini–Hochberg method^73^, and an FDR threshold of 0.1 was set for significance. Statistical analysis was carried out using IBM SPSS Statistics 19 software (IBM Corp., Armonk, NY, USA) and the online statistics calculator VassarStats (www.vassarstats.net). *P* < 0.05 was considered significant.

## Supplementary information

Supplementary Information

Source Data

## Data Availability

The data supporting the main findings of this study are available within the article and its Supplementary Information and Supplementary Data files. Whole-exome sequencing data were deposited into the NCBI Sequence Read Archive under accession code SRP107053 (https://www.ncbi.nlm.nih.gov/sra/). The raw RNA sequencing data are available from NCBI and are archived under accession number SRP180943 (https://www.ncbi.nlm.nih.gov/sra/). The raw LC/MS data have been deposited to the ProteomeXchange Consortium^74^ via the PRIDE^75^ partner repository with the dataset identifier PXD013285 (www.ebi.ac.uk/pride). The source data underlying Figs. 3b–f, 4b–f, 5a–d, f, and 6b, c, e and Supplementary Fig. 6 are provided as a Source Data file. Any other data are available from the authors upon reasonable request.
