# Supplementary material for: Recurrent GNAQ mutation encoding T96S in natural killer/T cell lymphoma
Source: Nat Commun. 2019 Sep 16;10:4209. doi: 10.1038/s41467-019-12032-9 (PMC6746819; doi:10.1038/s41467-019-12032-9)
Supplement: Supplementary file 1 — Supplementary Information [file 41467_2019_12032_MOESM1_ESM.pdf]

Supplementary information

Recurrent *GNAQ* mutation encoding T96S in natural killer/T cell lymphoma

Li et al

Table of Contents

1. Supplementary Figures

Supplementary Fig. 1……………………………………………………………..3

Supplementary Fig. 2……………………………………………………………..4

Supplementary Fig. 3……………………………………………………………..5

Supplementary Fig. 4……………………………………………………………..6

Supplementary Fig. 5……………………………………………………………..7

Supplementary Fig. 6……………………………………………………………..8

Supplementary Fig. 7……………………………………………………………..9

Supplementary Fig. 8……………………………………………………………..12

Supplementary Fig. 9……………………………………………………………..13

Supplementary Fig. 10………..…………………………………………………..14

2. Supplementary Tables

Supplementary Table 1………..…………………………………………….……..15

Supplementary Table 2………..…………………………………………….……..19

Supplementary Table 3………..…………………………………………….……..21

Supplementary Table 4………..…………………………………………….……..55

Supplementary Table 5………..…………………………………………….……..58

Supplementary Table 6………..…………………………………………….……..60

Supplementary Table 7………..…………………………………………….……..64

Supplementary Table 8………..…………………………………………….……..70

Supplementary Table 9………..…………………………………………….……..105

Supplementary Table 10…..…..…………………………………………….……..106

Supplementary Table 11………..……………..………………………….……..107

Supplementary Table 12………..……………..………………………….……..109

Supplementary Table 13………..……………..………………………….……..110

Supplementary Table 14………..……………..………………………….……..111

Supplementary Table 15………..……………..………………………….……..115

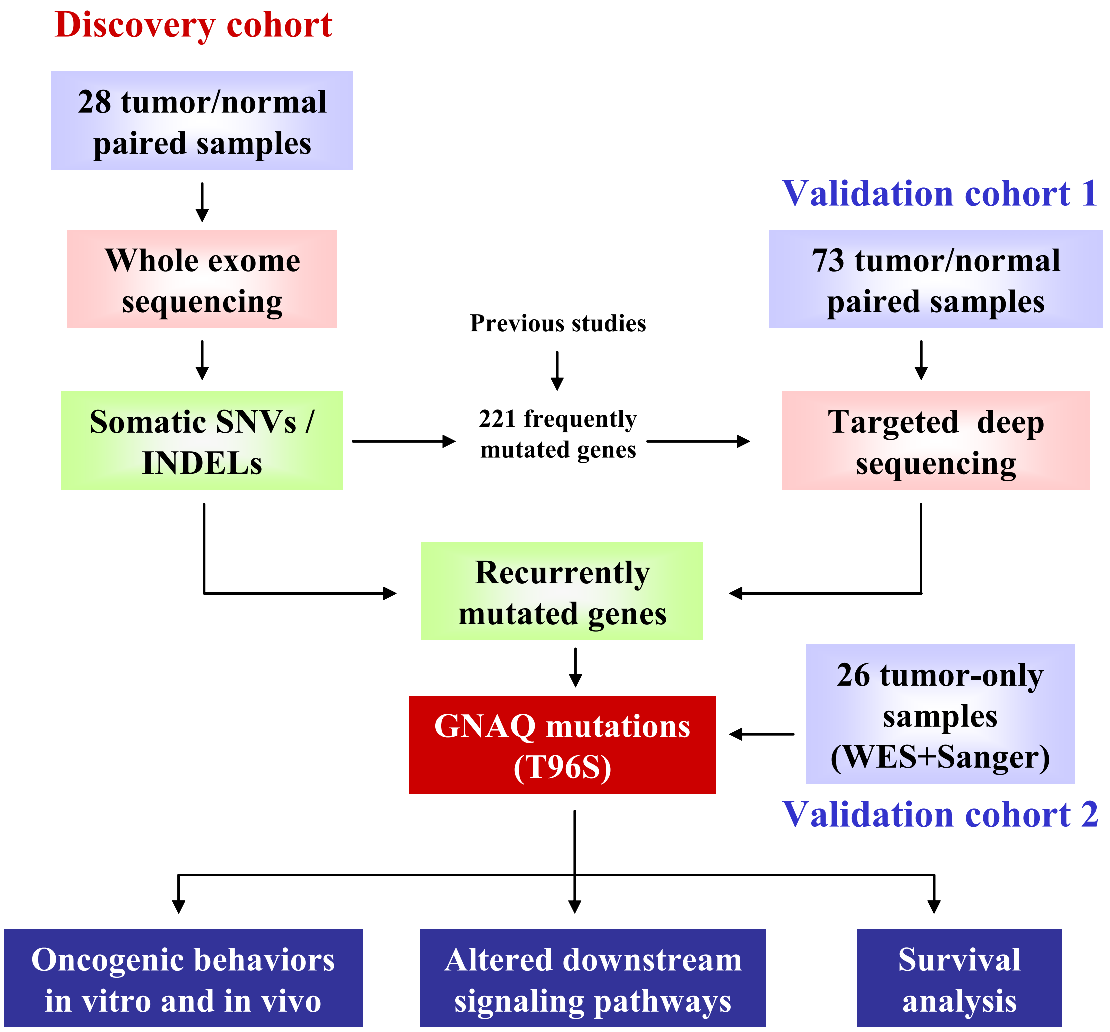

Supplementary Fig. 1 Experimental design. A combination of whole-exome sequencing (n=28) and targeted deep sequencing (n=73) was performed on a total of 101 NKTCL pairs. The recurrently mutated *GNAQ* T96S mutations were identified, and these mutations were further validated in a cohort of 26 NKTCL samples without matched normal tissue. Functional studies of *GNAQ* (wild-type and mutants) were performed, and the altered downstream signaling pathways were examined. Moreover the associations between *GNAQ* T96S mutations and prognosis were assessed.

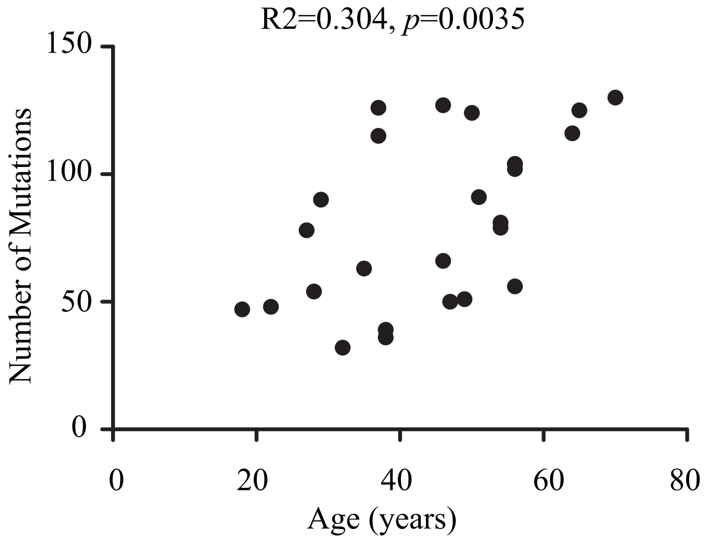

Supplementary Fig. 2 The correlation of somatic nonsilent mutation burden and age in NKTCL patients (R^2=0.295, *p*=0.004) after excluding the two patients with more than 200 mutations.

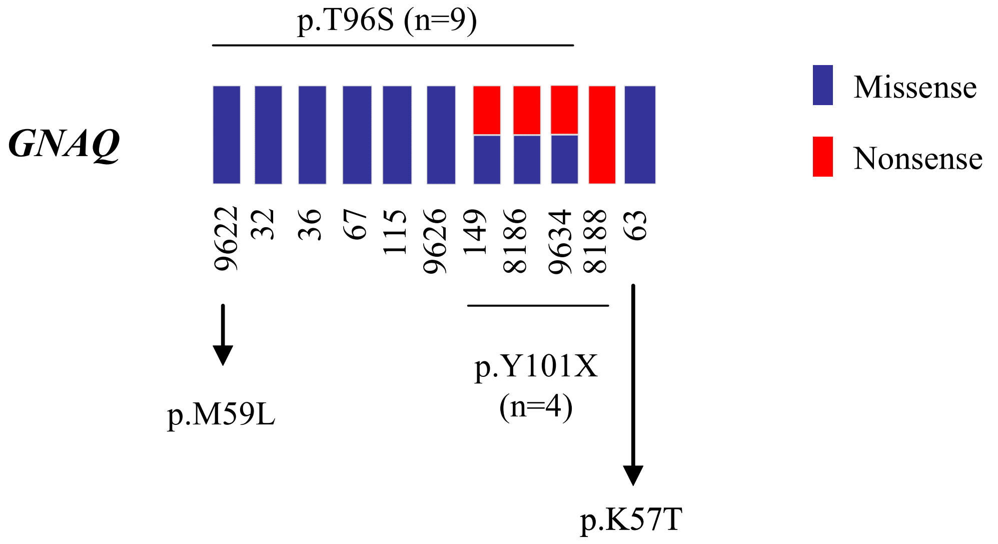

Supplementary Fig. 3 Fifteen point mutations in *GNAQ* were identified in 11 NKTCL samples: 9 mutations encoding T96S, 4 mutations encoding Y101X, and one mutation encoding p.K57T, and one mutation encoding p.M59L. Mutations in T96S cooccurred with Y101X and p.M59L in 3 cases and one case, respectively.

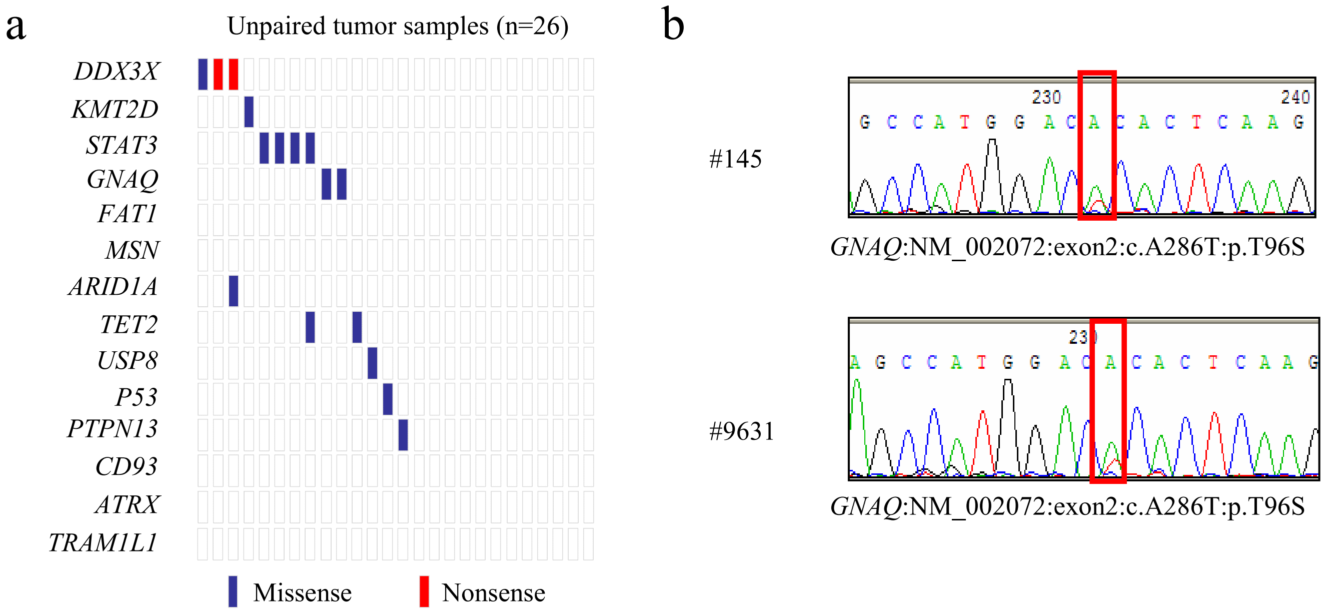

Supplementary Fig. 4 Validation of the frequently mutated genes in an independent cohort of NKTCL (n=26) with no matched normal tissue. **a** Mutation status of the 14 frequently mutated genes in this cohort. **b** Validation of *GNAQ* T96S mutations in two NKTCL patients (#145 and #9631) by Sanger sequencing.

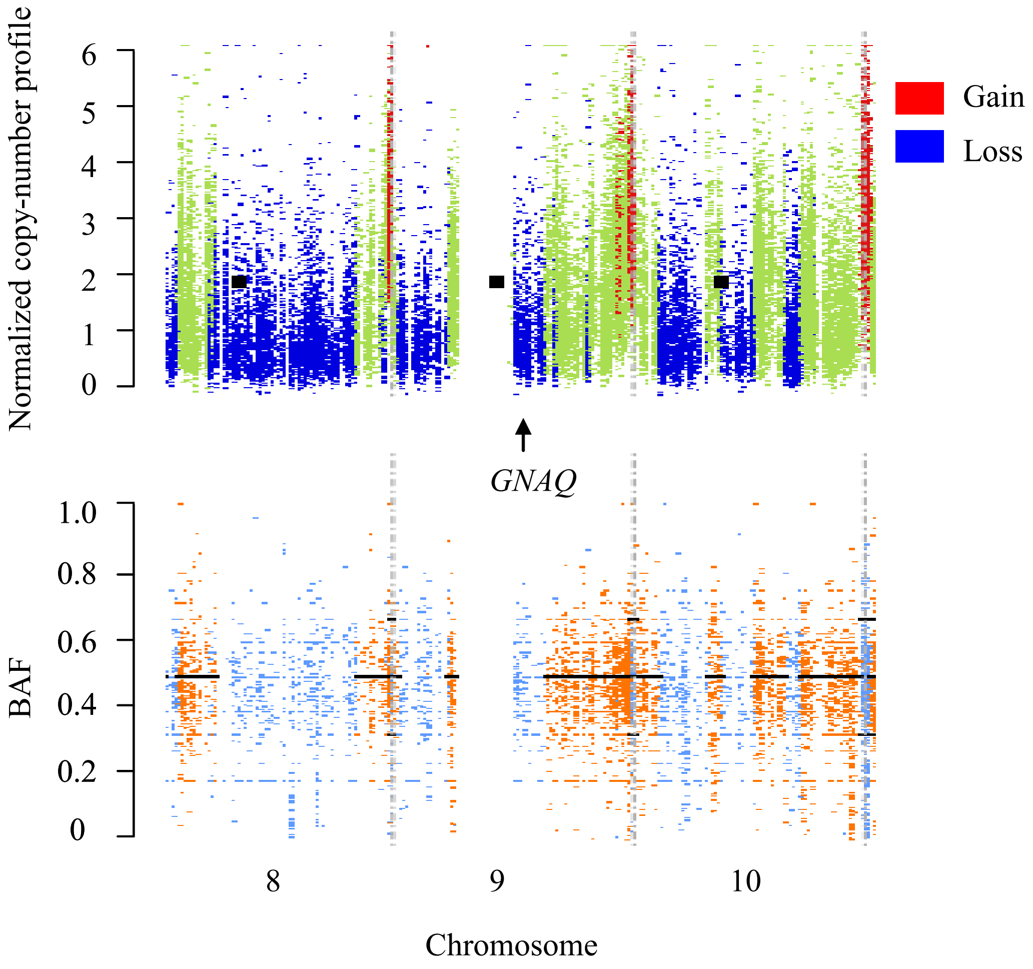

Supplementary Fig. 5 Control-FREEC calculates copy number and BAF profiles and detects regions of copy number gain/loss.

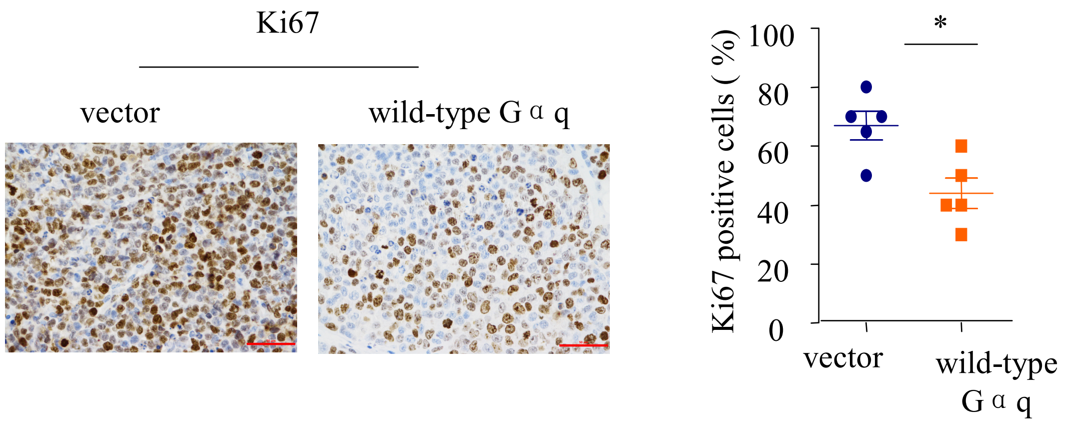

Supplementary Fig. 6 Representative images and quantitative data for Ki67 staining of xenograft tumor tissues. Data are expressed as the mean±s.e.m.; scale bars, 50 μm. *<0.05. Source data are provided as a Source Data file.

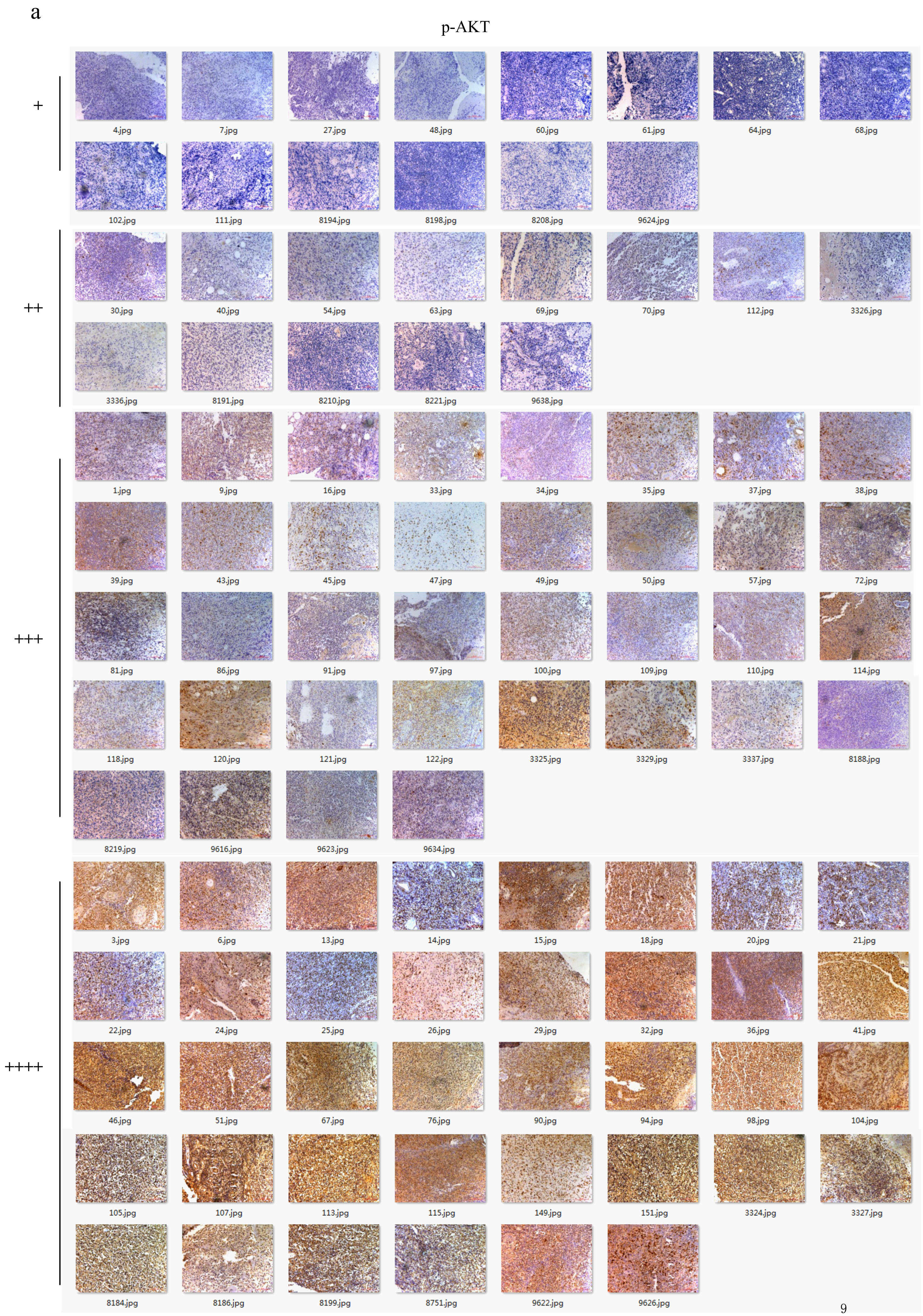
a
p-AKT
+
4.jpg 7.jpg 27.jpg 48.jpg 60.jpg 61.jpg 64.jpg 68.jpg
102.jpg 111.jpg 8194.jpg 8198.jpg 8208.jpg 9624.jpg
++
30.jpg 40.jpg 54.jpg 63.jpg 69.jpg 70.jpg 112.jpg 3326.jpg
3336.jpg 8191.jpg 8210.jpg 8221.jpg 9638.jpg
+++
1.jpg 9.jpg 16.jpg 33.jpg 34.jpg 35.jpg 37.jpg 38.jpg
39.jpg 43.jpg 45.jpg 47.jpg 49.jpg 50.jpg 57.jpg 72.jpg
81.jpg 86.jpg 91.jpg 97.jpg 100.jpg 109.jpg 110.jpg 114.jpg
118.jpg 120.jpg 121.jpg 122.jpg 3325.jpg 3329.jpg 3337.jpg 8188.jpg
8219.jpg 9616.jpg 9623.jpg 9634.jpg
++++
3.jpg 6.jpg 13.jpg 14.jpg 15.jpg 18.jpg 20.jpg 21.jpg
22.jpg 24.jpg 25.jpg 26.jpg 29.jpg 32.jpg 36.jpg 41.jpg
46.jpg 51.jpg 67.jpg 76.jpg 90.jpg 94.jpg 98.jpg 104.jpg
105.jpg 107.jpg 113.jpg 115.jpg 149.jpg 151.jpg 3324.jpg 3327.jpg
8184.jpg 8186.jpg 8199.jpg 8751.jpg 9622.jpg 9626.jpg

b

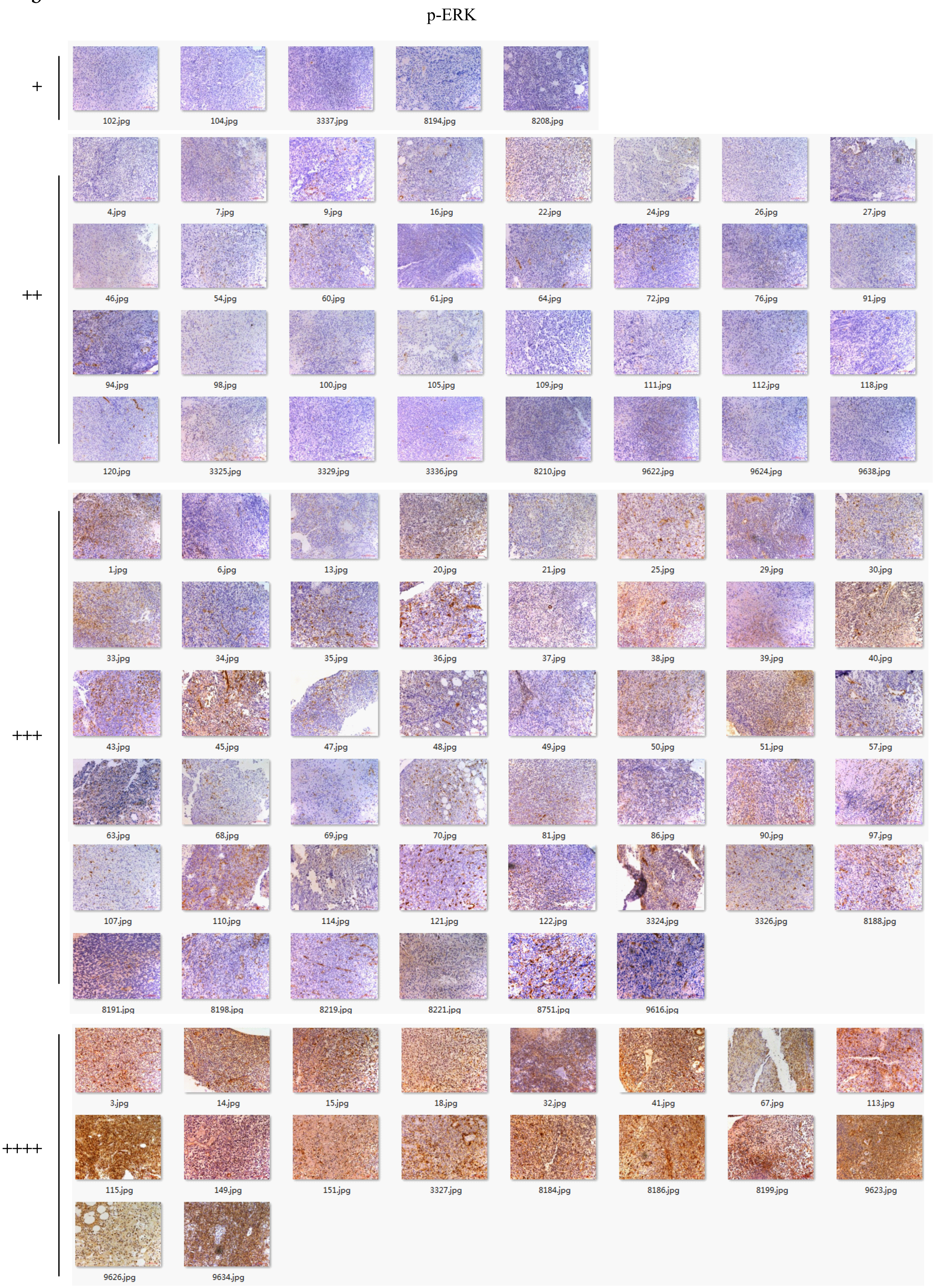

p-ERK
+
102.jpg
104.jpg
3337.jpg
8194.jpg
8208.jpg
++
4.jpg
7.jpg
9.jpg
16.jpg
22.jpg
24.jpg
26.jpg
27.jpg
46.jpg
54.jpg
60.jpg
61.jpg
64.jpg
72.jpg
76.jpg
91.jpg
94.jpg
98.jpg
100.jpg
105.jpg
109.jpg
111.jpg
112.jpg
118.jpg
120.jpg
3325.jpg
3329.jpg
3336.jpg
8210.jpg
9622.jpg
9624.jpg
9638.jpg
+++
1.jpg
6.jpg
13.jpg
20.jpg
21.jpg
25.jpg
29.jpg
30.jpg
33.jpg
34.jpg
35.jpg
36.jpg
37.jpg
38.jpg
39.jpg
40.jpg
43.jpg
45.jpg
47.jpg
48.jpg
49.jpg
50.jpg
51.jpg
57.jpg
63.jpg
68.jpg
69.jpg
70.jpg
81.jpg
86.jpg
90.jpg
97.jpg
107.jpg
110.jpg
114.jpg
121.jpg
122.jpg
3324.jpg
3326.jpg
8188.jpg
8191.jpg
8198.jpg
8219.jpg
8221.jpg
8751.jpg
9616.jpg
++++
3.jpg
14.jpg
15.jpg
18.jpg
32.jpg
41.jpg
67.jpg
113.jpg
115.jpg
149.jpg
151.jpg
3327.jpg
8184.jpg
8186.jpg
8199.jpg
9623.jpg
9626.jpg
9634.jpg

c

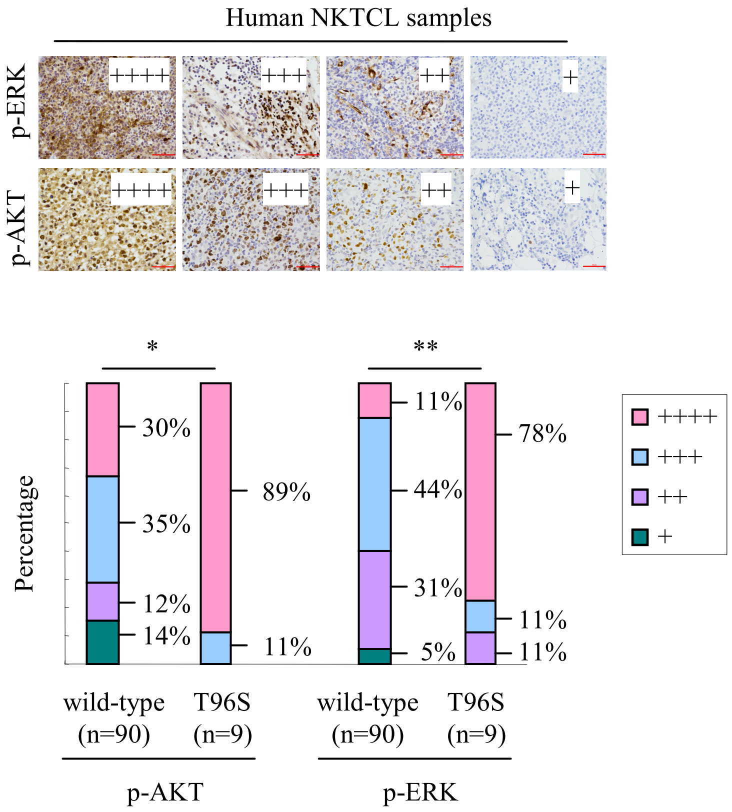

Supplementary Fig. 7 Immunohistochemical staining results of phospho-AKT (**a**) and phospho-ERK (**b**) in human NKTCL samples with wild-type (n=90) and T96S mutant (n=9). The expression levels of phospho-ERK and phospho-AKT were scored semiquantitatively according to the percentage of IHC-positive cells: +, 0%; ++, 1–10%; +++, 11–50%; or ++++, >50%. *p* values were calculated using a two-sided Fisher's exact test (**c**). Scale bars, 50 μm. *p<0.05, ** p<0.01.

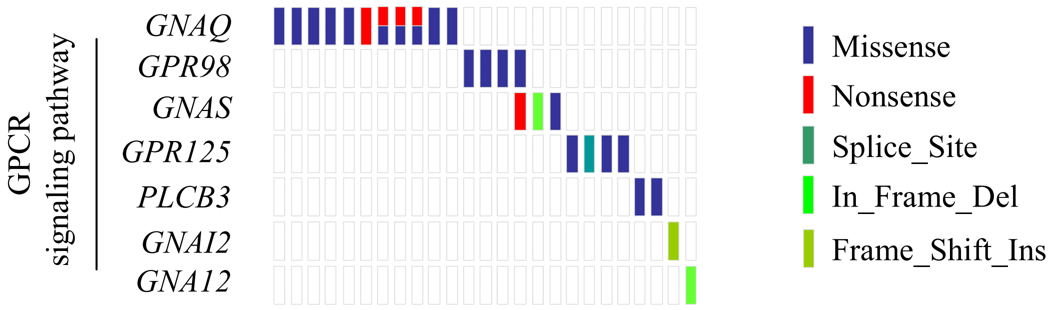

Supplementary Fig. 8 Heat map of genes in the commonly mutated GPCR signaling pathway in NKTCL. Each row represents a mutated gene. Each column represents a patient sample. Blocks are color coded by the functional type of mutation.

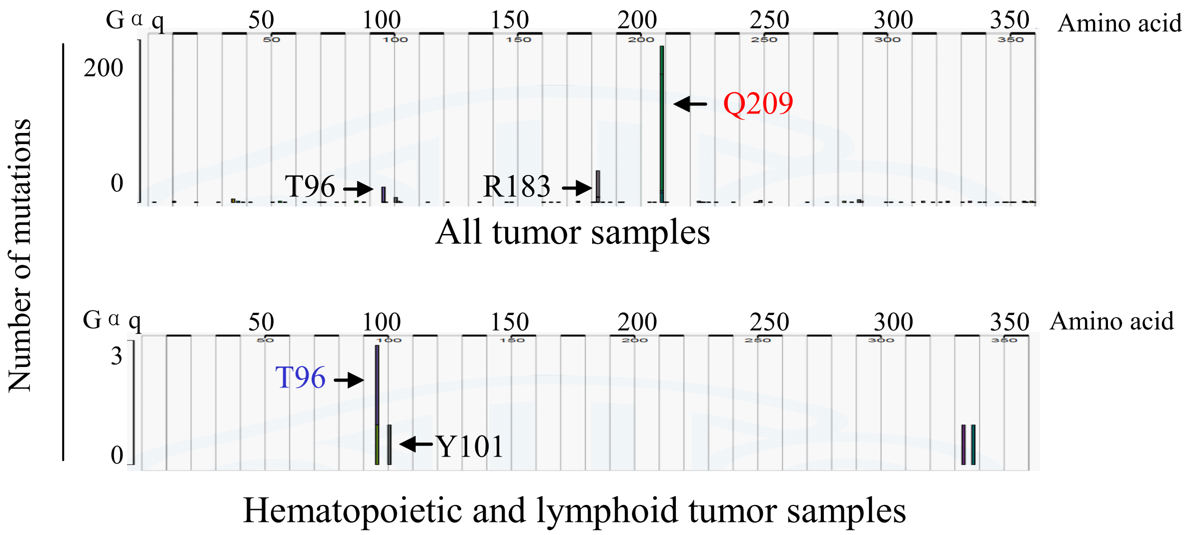

Supplementary Fig. 9 The most common sites of *GNAQ* mutations identified in different tumor tissues in the COSMIC database.

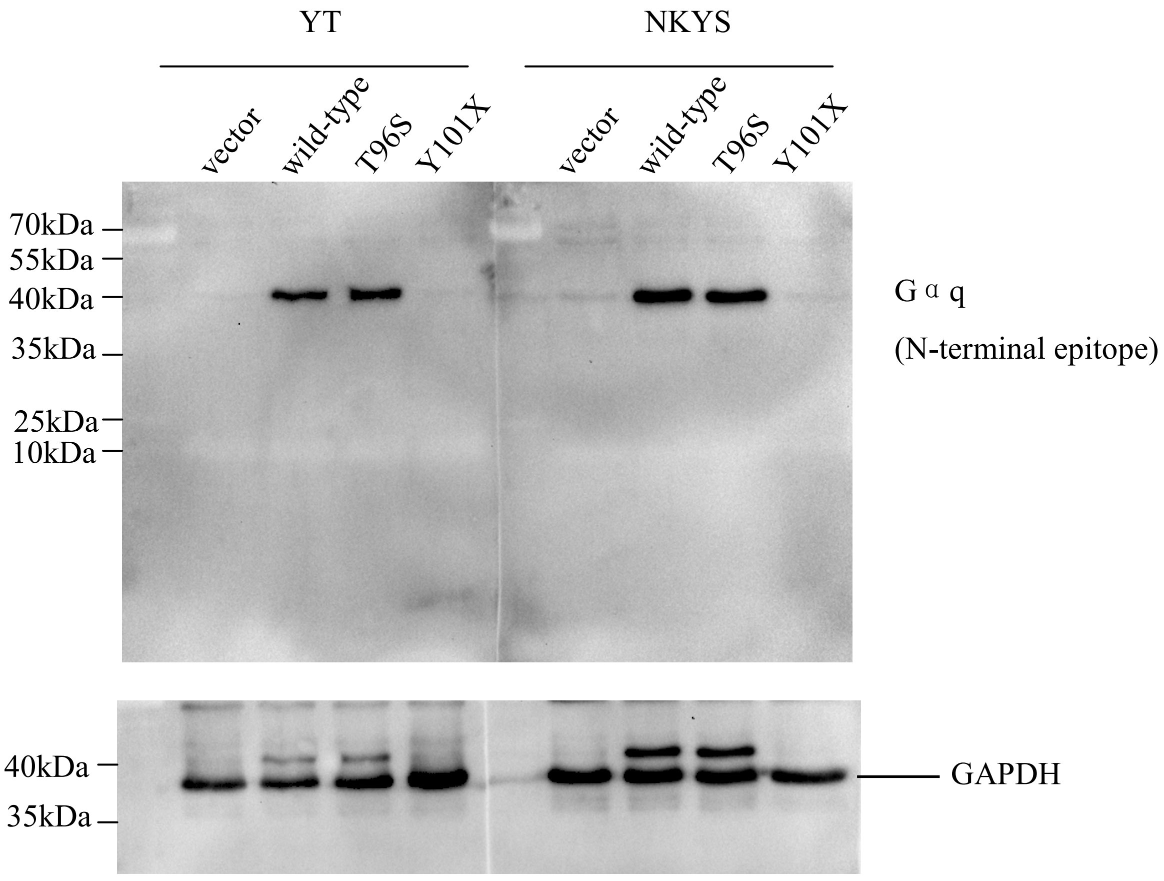

Supplementary Fig. 10 Western blot analysis of Gαq Y101X mutant expression in YT and NKYS cells.

Supplementary Table 1. Demographics and clinical features of 127 subjects with NKTCL.

NO.	Patient ID	Age (year)	Gender	ECOG PS	Ann Arbor stage	LDH	B symptoms	IPI	Primary site	Ki67	EBV DNA	TCR rearrangement	PFS (month)	PFS event	OS (month)	OS event	Therapy	Sequencing	CD56	CD3ε	EBER	TIA1	Granzyme B	CD2	CD4	CD8	βF1	TCRγ
1	9624	38	Male	1	II	Normal	Absence	0	Nasal	70%	Normal	-	8.0	0.0	22.6	0	CT+RT	WES	+	+	+	+	+	+	-	-	-	-
2	3329	54	Male	1	II	Normal	Presence	0	Nasal	50%	Normal	-	27.9	0.0	27.9	0	CT+RT	WES	+	+	+	-	+	+	-	-	-	-
3	8191	46	Male	0	I	Normal	Absence	0	Nasal	20%	Normal	-	59.0	0.0	59.0	0	CT+RT	WES	+	+	+	+	+	+	-	-	-	-
4	8194	70	Male	0	II	Normal	Absence	1	Nasal	70%	Normal	-	56.5	0.0	56.5	0	CT+RT	WES	+	+	+	+	+	+	-	-	-	-
5	8186	56	Female	0	II	Normal	Absence	0	Nasal	70%	Elevated	-	22.0	1.0	26.0	1	CT+RT	WES	+	+	+	+	+	+	-	-	-	-
6	8184	54	Male	0	III	Normal	Absence	1	Nasal	70%	Elevated	-	62.0	0.0	62.0	0	CT	WES	+	+	+	+	+	+	-	-	-	-
7	3324	37	Male	0	II	Normal	Absence	0	Nasal	80%	Normal	-	39.0	1.0	39.0	1	CT+RT	WES	+	+	+	+	+	-	-	-	-	-
8	9638	56	Male	0	IV	Normal	Absence	2	Nasal	40%	Normal	-	7.0	1.0	7.0	1	CT+RT	WES	+	+	+	+	+	+	-	-	-	-
9	9634	18	Female	1	IV	Normal	Presence	2	Nasal	60%	Elevated	-	36.0	1.0	36.0	1	CT	WES	+	+	+	+	+	+	-	-	-	-
10	8221	28	Female	2	II	Elevated	Absence	2	Nasal	60%	Normal	-	53.5	0.0	53.5	0	CT+RT	WES	+	+	+	+	+	+	-	-	-	-
11	8219	50	Male	1	II	Normal	Absence	0	Nasal	60%	Normal	-	53.0	0.0	53.0	0	CT+RT	WES	+	+	+	+	+	-	-	-	-	-
12	9623	35	Male	1	I	Normal	Absence	0	Nasal	60%	NA	-	48.0	0.0	48.0	0	CT+RT	WES	+	+	+	+	+	+	-	-	-	-
13	8199	22	Male	0	II	Normal	Presence	0	Nasal	30%	Normal	-	55.3	0.0	55.3	0	CT+RT	WES	+	+	+	+	+	+	-	-	-	-
14	3325	51	Female	0	I	Normal	Presence	0	Nasal	90%	Normal	-	42.0	1.0	42.0	1	CT+RT	WES	+	+	+	+	+	+	-	-	-	-
15	8188	29	Female	0	I	Normal	Presence	0	Nasal	70%	Normal	-	60.0	1.0	60.0	1	CT	WES	+	+	+	+	+	+	-	-	-	-
16	8210	54	Male	1	I	Normal	Presence	0	Nasal	40%	Normal	+	51.0	0.0	51.0	0	CT+RT	WES	+	+	+	+	+	+	-	-	-	-
17	8751	37	Female	1	II	Normal	Absence	0	Nasal	50%	Normal	-	55.0	0.0	55.0	0	CT+RT	WES	+	+	+	+	+	+	-	-	-	-
18	9626	32	Female	1	II	Normal	Absence	0	Nasal	60%	Normal	-	49.0	1.0	49.0	0	CT	WES	+	+	+	+	+	+	-	-	-	-
19	149	52	Male	2	III	Normal	Presence	2	Nasal	60%	Elevated	-	44.0	1.0	44.0	1	CT+RT	WES	+	+	+	+	+	+	-	-	-	-
20	3336	47	Male	1	II	Normal	Absence	0	Nasal	50%	Normal	-	5.0	1.0	37.6	1	CT+RT	WES	+	+	+	+	+	+	-	-	-	-
21	3337	27	Female	1	IV	Elevated	Presence	3	Nasal	30%	NA	-	38.0	0.0	38.0	0	CT	WES	+	+	+	+	+	+	-	-	-	-
22	9622	38	Male	1	IV	Normal	Presence	2	Nasal	70%	Elevated	+	6.0	1.0	10.0	1	CT	WES	+	+	+	-	+	+	-	-	-	-
23	8208	56	Male	0	II	Elevated	Presence	1	Nasal	40%	Normal	-	50.0	0.0	50.0	0	CT+RT	WES	+	+	+	+	+	+	-	-	-	-
24	8198	46	Male	1	II	Normal	Absence	0	Nasal	60%	Normal	-	48.0	0.0	48.0	0	CT+RT	WES	+	+	+	+	+	+	-	-	-	-
25	9616	49	Male	3	IV	Normal	Presence	3	Nasal	60%	Elevated	-	1.0	1.0	44.0	1	CT	WES	+	+	+	+	+	+	-	-	-	-
26	3326	64	Male	2	I	Elevated	Presence	3	Nasal	80%	NA	+	44.0	0.0	44.0	0	CT	WES	+	+	+	+	+	+	-	-	-	-
27	151	65	Male	0	I	Normal	Presence	2	Nasal	40%	Elevated	-	68.0	0.0	68.0	0	RT	WES	+	+	+	+	+	+	-	-	-	-
28	3327	56	Male	1	I	Elevated	Absence	1	Nasal	40%	Elevated	-	49.0	0.0	49.0	0	CT	WES	+	+	+	+	+	+	-	-	-	-
29	100	15	Male	1	II	Elevated	Presence	1	Nasal	61%	Normal	-	9.5	1.0	9.5	1	CT	TS	+	+	+	+	+	+	-	-	-	-
30	102	33	Male	0	IV	Normal	Absence	2	Nasal	80%	Normal	-	3.0	0.0	11.0	0	CT	TS	+	+	+	+	+	+	-	-	-	-
31	104	28	Male	1	I	Normal	Presence	0	Nasal	50%	Normal	+	14.0	0.0	14.0	0	CT	TS	+	+	+	+	+	+	-	-	-	-
32	105	70	Male	1	IV	Elevated	Absence	4	Nasal	80%	Elevated	-	16.0	1.0	16.0	1	CT	TS	+	+	+	+	+	+	-	-	-	-
33	107	45	Male	3	IV	Normal	Absence	3	Nasal	90%	Elevated	-	5.8	1.0	8.8	1	CT	TS	+	+	+	+	+	+	-	-	-	-
34	109	43	Male	0	II	Normal	Absence	0	Nasal	30%	Elevated	-	24.0	1.0	24.0	1	CT	TS	+	+	+	+	+	+	-	-	-	-

35	110	59	Female	1	IV	Elevated	Absence	3	Adrenal gland	90%	Elevated	-	3.0	1.0	7.0	1	CT	TS	+	+	+	+	+	+	-	-	-	-
36	111	35	Female	3	IV	Elevated	Presence	4	Intestine	70%	Elevated	-	3.0	1.0	3.5	1	CT	TS	+	+	+	+	+	+	-	-	-	-
37	112	50	Male	0	I	Normal	Absence	0	Nasal	70%	Normal	-	16.0	0.0	16.0	0	RT	TS	+	+	+	+	+	+	-	-	-	-
38	113	49	Male	1	IV	Elevated	Presence	3	Intestine	40%	NA	-	14.5	0.0	14.5	0	CT	TS	+	+	+	+	+	+	-	-	-	-
39	114	70	Male	0	I	Elevated	Absence	2	Nasal	80%	Elevated	+	69.0	0.0	69.0	0	CT	TS	+	+	+	+	+	+	-	-	-	+
40	115	60	Male	1	II	Normal	Absence	0	Nasal	40%	Normal	-	49.0	1.0	49.0	0	CT+RT	TS	+	+	+	+	+	+	-	-	-	-
41	118	47	Male	2	III	Elevated	Presence	3	Nasal	80%	Elevated	-	16.0	1.0	16.0	1	CT	TS	+	+	+	+	+	+	-	-	-	-
42	120	74	Male	1	IV	Normal	Absence	3	Penis	40%	Elevated	-	14.0	0.0	14.0	0	CT	TS	+	+	+	+	+	-	-	-	-	-
43	121	8	Male	0	IV	Elevated	Absence	3	Nasal	60%	Normal	-	7.0	1.0	14.0	1	CT	TS	+	+	+	+	+	+	-	-	-	-
44	122	32	Male	1	III	Normal	Absence	1	Nasal	60%	Normal	-	15.0	0.0	15.0	0	CT	TS	+	+	+	+	+	+	-	-	-	-
45	32	61	Male	2	III	Elevated	Presence	4	Nasal	70%	NA	-	1.0	1.0	1.0	1	CT	TS	+	+	+	+	+	+	-	-	-	-
46	33	51	Male	0	III	Normal	Absence	1	Nasal	40%	Normal	-	20.0	0.0	34.0	0	CT	TS	+	+	+	+	+	+	-	-	-	-
47	34	34	Male	1	III	Elevated	Absence	2	Nasal	50%	Normal	-	30.0	1.0	30.0	1	CT	TS	+	+	+	+	+	+	-	-	-	-
48	35	43	Female	0	II	Normal	Absence	0	Nasal	60%	NA	-	2.0	0.0	42.0	0	CT+RT	TS	+	+	+	+	+	+	-	-	-	-
49	36	15	Male	1	IV	Elevated	Presence	3	Nasal	60%	Normal	-	4.3	1.0	4.3	1	CT	TS	+	+	+	+	+	+	-	-	-	-
50	37	59	Male	1	I	Normal	Absence	0	Nasal	40%	NA	-	24.5	0.0	24.5	0	RT	TS	+	+	+	+	+	+	-	-	-	-
51	38	51	Male	1	I	Normal	Absence	1	Intestine	70%	NA	-	34.0	1.0	34.0	0	CT	TS	+	+	+	+	+	+	-	-	-	-
52	39	74	Male	0	I	Normal	Absence	1	Nasal	81%	Normal	-	37.0	0.0	37.0	0	CT+RT	TS	+	+	+	+	+	+	-	-	-	-
53	40	35	Male	1	II	Normal	Presence	0	Nasal	70%	Elevated	-	4.0	1.0	30.5	0	CT+RT	TS	+	+	+	+	+	+	-	-	-	-
54	41	66	Female	1	I	Normal	Presence	2	Intestine	90%	Normal	-	6.0	1.0	6.0	1	CT	TS	+	+	+	+	+	+	-	-	-	-
55	43	35	Female	1	IV	Elevated	Absence	3	Nasal	10%	Elevated	-	12.5	1.0	12.5	1	CT+RT	TS	+	+	+	+	+	+	-	-	-	-
56	45	57	Male	1	I	Normal	Absence	0	Nasal	10%	Normal	-	28.0	0.0	28.0	0	CT+RT	TS	+	+	+	+	+	+	-	-	-	-
57	46	53	Male	1	I	Elevated	Absence	1	Nasal	70%	Normal	-	28.0	0.0	28.0	0	CT+RT	TS	+	+	+	+	+	+	-	-	-	-
58	47	36	Female	0	III	Normal	Absence	1	Nasal	60%	Normal	-	35.0	0.0	35.0	0	CT	TS	+	+	+	+	+	+	-	-	-	-
59	48	44	Female	1	I	Normal	Presence	0	Nasal	50%	Normal	-	24.0	1.0	24.0	1	CT+RT	TS	+	+	+	+	+	+	-	-	-	-
60	49	58	Male	1	III	Elevated	Presence	2	Nasal	30%	NA	+	4.0	0.0	40.0	0	CT	TS	+	+	+	+	+	+	-	+/-	-	+
61	50	28	Male	1	II	Elevated	Absence	1	Nasal	70%	Normal	-	28.6	0.0	28.6	0	CT+RT	TS	+	+	+	+	+	+	-	-	-	-
62	51	50	Female	1	II	Normal	Absence	0	Nasal	60%	NA	-	32.0	1.0	46.0	0	CT+RT	TS	+	+	+	+	+	+	-	-	-	-
63	54	76	Female	1	II	Normal	Presence	1	Nasal	40%	NA	-	3.0	0.0	30.0	0	CT	TS	+	+	+	+	+	+	-	-	-	-
64	57	48	Male	1	IV	Normal	Presence	2	Nasal	90%	Normal	-	21.0	1.0	21.0	1	CT	TS	+	+	+	+	+	+	-	-	-	-
65	60	34	Female	0	II	Normal	Absence	0	Nasal	40%	Normal	-	6.0	0.0	62.0	0	CT	TS	+	+	+	+	+	+	-	-	-	-
66	61	55	Male	1	III	Elevated	Presence	2	Nasal	80%	NA	-	8.0	0.0	63.0	0	CT	TS	+	+	+	+	+	+	-	-	-	-
67	63	33	Male	1	IV	Elevated	Presence	3	Nasal	60%	Elevated	-	20.0	1.0	20.0	1	CT	TS	+	+	+	+	+	+	-	-	-	-
68	64	50	Male	1	III	Normal	Presence	1	Nasal	70%	Elevated	-	24.0	0.0	24.0	0	CT	TS	+	+	+	+	+	+	-	-	-	-
69	67	61	Male	1	III	Normal	Presence	2	Nasal	80%	Elevated	-	8.3	1.0	8.3	1	CT	TS	+	+	+	+	+	+	-	-	-	-
70	68	42	Male	1	II	Normal	Absence	0	Nasal	60%	NA	-	29.0	0.0	35.0	0	CT+RT	TS	+	+	+	+	+	+	-	-	-	-
71	69	23	Female	1	I	Elevated	Absence	1	Nasal	70%	NA	-	25.0	1.0	40.0	1	CT	TS	+	+	+	+	+	+	-	-	-	-
72	70	40	Female	1	I	Normal	Absence	0	Nasal	20%	NA	-	40.0	0.0	40.0	0	CT+RT	TS	+	+	+	+	+	+	-	-	-	-

73	72	41	Male	1	II	Elevated	Absence	2	Lung	20%	Normal	-	10.0	0.0	31.0	0	CT+RT	TS	+	+	+	+	+	+	-	-	-	-
74	76	45	Male	1	I	Normal	Presence	0	Nasal	40%	Normal	-	28.0	0.0	28.0	0	CT	TS	+	+	+	+	+	+	-	-	-	-
75	81	44	Female	1	IV	Elevated	Presence	3	Nasal	60%	Elevated	-	1.5	1.0	1.5	1	CT	TS	+	+	+	+	+	+	-	-	-	-
76	86	42	Male	1	I	Normal	Absence	0	Nasal	40%	Elevated	+	7.0	1.0	7.0	1	CT+RT	TS	+	+	+	+	+	+	-	-	-	+
77	90	54	Female	1	I	Normal	Absence	0	Nasal	30%	Normal	-	30.0	1.0	48.0	0	RT	TS	+	+	+	+	+	+	-	-	-	-
78	91	20	Male	1	II	Normal	Absence	0	Nasal	90%	Normal	+	18.0	1.0	18.0	1	RT	TS	+	+	+	+	+	+	-	-	-	+
79	97	22	Female	1	II	Normal	Presence	1	Lymph node	80%	Elevated	-	4.2	1.0	6.0	1	CT	TS	+	+	+	+	+	+	-	-	-	-
80	98	29	Male	1	II	Elevated	Presence	2	Skin	30%	Elevated	-	14.0	1.0	27.0	1	CT+RT	TS	+	+	+	+	+	+	-	-	-	-
81	94	17	Female	1	I	Elevated	Presence	1	Nasal	40%	NA	-	48.0	0.0	63.0	0	CT+RT	TS	+	+	+	+	+	+	-	-	-	-
82	25	23	Female	0	II	Normal	Presence	0	Nasal	40%	Normal	-	9.0	0.0	63.0	0	CT	TS	+	+	+	+	+	+	-	-	-	-
83	24	39	Male	1	II	Normal	Presence	0	Nasal	40%	Normal	-	37.0	1.0	39.0	1	CT+RT	TS	+	+	+	+	+	+	-	-	-	-
84	22	46	Male	0	II	Normal	Absence	0	Nasal	30%	Normal	-	66.0	0.0	66.0	0	CT	TS	+	+	+	+	+	+	-	-	-	-
85	21	51	Male	1	II	Normal	Absence	0	Nasal	60%	Normal	-	17.0	0.0	17.0	0	CT+RT	TS	+	+	+	+	+	+	-	-	-	-
86	20	58	Male	0	II	Normal	Absence	0	Nasal	30%	Normal	-	45.0	0.0	60.0	0	CT+RT	TS	+	+	+	+	+	+	-	-	-	-
87	9	38	Male	1	II	Elevated	Absence	1	Nasal	20%	Normal	-	12.0	0.0	45.0	0	CT+RT	TS	+	+	+	+	+	+	-	-	-	-
88	7	52	Male	1	II	Elevated	Presence	1	Nasal	20%	Normal	-	8.0	1.0	8.0	1	CT+RT	TS	+	+	+	+	+	+	-	-	-	-
89	6	44	Male	0	I	Normal	Absence	0	Nasal	30%	Normal	-	16.0	0.0	36.0	0	RT	TS	+	+	+	+	+	+	-	-	-	-
90	4	74	Male	1	II	Elevated	Absence	2	Nasal	80%	Normal	-	10.0	0.0	38.0	0	CT+RT	TS	+	+	+	+	+	+	-	-	-	-
91	30	55	Female	1	II	Normal	Absence	0	Nasal	30%	NA	-	63.0	0.0	63.0	0	CT	TS	+	+	+	+	+	+	-	-	-	-
92	3	52	Male	0	III	Normal	Absence	2	Esophagus	90%	Normal	-	13.0	0.0	13.0	0	CT+RT	TS	+	+	+	+	+	+	-	-	-	-
93	29	37	Male	0	I	Normal	Absence	0	Nasal	40%	NA	-	59.0	1.0	59.0	1	CT	TS	+	+	+	+	+	+	-	-	-	-
94	27	56	Male	1	I	Normal	Presence	0	Nasal	60%	NA	-	60.0	1.0	60.0	1	CT	TS	+	+	+	+	+	+	-	-	-	-
95	26	18	Female	1	IV	Normal	Absence	2	Nasal	30%	NA	-	23.0	1.0	23.0	1	CT+RT	TS	+	+	+	+	+	+	-	-	-	-
96	18	62	Female	1	I	Normal	Presence	1	Nasal	40%	Normal	-	23.0	1.0	27.0	1	CT	TS	+	+	+	+	+	+	-	-	-	-
97	16	51	Male	1	II	Normal	Absence	0	Nasal	50%	Normal	-	18.0	0.0	40.0	0	RT	TS	+	+	+	+	+	+	-	-	-	-
98	15	36	Male	0	I	Elevated	Presence	1	Nasal	70%	Normal	-	1.2	0.0	1.2	0	CT	TS	+	+	+	+	+	+	-	-	-	-
99	14	24	Male	0	I	Normal	Absence	0	Nasal	60%	Normal	-	28.0	0.0	28.0	1	CT	TS	+	+	+	+	+	+	-	-	-	-
100	13	28	Female	1	I	Normal	Absence	0	Nasal	40%	Normal	-	50.0	0.0	50.0	0	CT	TS	+	+	+	+	+	+	-	-	-	-
101	1	36	Female	1	II	Normal	Absence	0	Nasal	40%	Normal	-	35.0	1.0	42.0	0	CT	TS	+	+	+	+	+	+	-	-	-	-
102	8170 *	22	Female	0	II	Normal	Absence	0	Nasal	60%	NA	-	NA	NA	NA	NA	CT+RT	WES	+	+	+	+	+	+	+/-	-	-	-
103	8173 *	42	Female	1	II	Normal	Presence	0	Nasal	70%	Normal	-	NA	NA	NA	NA	CT+RT	WES	+	+	+	+	+	+	-	-	-	-
104	8178 *	40	Male	2	I	Normal	Presence	1	Nasal	50%	NA	+	NA	NA	NA	NA	CT+RT	WES	+	+	+	+	+	+	-	-	-	+
105	8181 *	40	Female	1	II	Normal	Absence	0	Nasal	50%	Normal	-	36	0	36	0	CT+RT	WES	+	+	+	+	+	+	-	-	-	-
106	8185 *	38	Female	0	II	Normal	Absence	1	Nasal	90%	NA	-	NA	NA	NA	NA	RT	WES	+	+	+	+	+	+	-	+/-	-	-
107	8192 *	27	Male	1	II	Normal	Absence	1	Nasal	70%	Normal	-	NA	NA	NA	NA	CT	WES	+	+	+	+	+	+	-	-	-	-
108	8195 *	64	Female	1	III	Normal	Presence	3	Nasal	70%	Normal	-	20	1	24	1	CT+RT	WES	+	+	+	+	+	+	-	-	-	-
109	8203 *	55	Female	0	III	Elevated	Presence	3	Nasal	80%	Elevated	-	NA	NA	NA	NA	CT+RT	WES	+	+	+	+	+	+	-	-	-	-
110	8204 *	63	Female	0	II	Elevated	Presence	3	Nasal	70%	Normal	-	NA	NA	NA	NA	RT	WES	+	+	+	+	+	+	-	-	-	-
111	8206 *	66	Female	0	II	Elevated	Presence	2	Nasal	60%	Elevated	-	NA	NA	NA	NA	CT	WES	+	+	+	+	+	+	-	-	-	-

112	8207 *	47	Male	1	I	Normal	Absence	1	Nasal	20%	Normal	-	42	0	42	0	CT+RT	WES	+	+	+	+	+	+	-	+/-	-	-
113	8212 *	45	Male	1	I	Elevated	Absence	2	Liver	80%	NA	-	NA	NA	NA	NA	CT+RT	WES	+	+	+	+	+	+	-	+	-	+
114	8213 *	59	Male	1	II	Normal	Absence	1	Nasal	60%	Normal	-	NA	NA	NA	NA	CT+RT	WES	+	+	+	+	+	+	-	-	-	-
115	8215 *	60	Male	0	II	Normal	Absence	2	Nasal	70%	Normal	-	NA	NA	NA	NA	CT+RT	WES	+	+	+	+	+	+	-	-	-	-
116	8216 *	55	Male	1	I	Elevated	Presence	2	Nasal	70%	Elevated	-	NA	NA	NA	NA	CT	WES	+	+	+	+	+	+	-	-	-	-
117	8218 *	60	Female	1	II	Normal	Absence	1	Nasal	20%	Elevated	-	NA	NA	NA	NA	CT+RT	WES	+	+	+	+	+	+	-	-	-	+
118	8222 *	61	Male	0	I	Normal	Absence	1	Lymph node	80%	NA	-	48	0	48	0	CT	WES	+	+	+	+	+	+	+/-	-	-	-
119	8223 *	45	Female	2	II	Normal	Presence	1	Nasal	70%	Normal	-	NA	NA	NA	NA	CT	WES	+	+	+	+	+	+	-	-	-	-
120	8224 *	29	Male	0	IV	Elevated	Absence	3	skin	40%	NA	-	12	1	12	1	CT+RT	WES	+	+	+	+	+	-	-	-	-	+
121	8225 *	43	Male	0	II	Normal	Absence	0	Nasal	80%	NA	+	NA	NA	NA	NA	CT	WES	+	+	+	+	+	+	-	-	-	+
122	9629 *	57	Male	1	II	Normal	Presence	1	Nasal	40%	Normal	-	NA	NA	NA	NA	CT	WES	+	+	+	+	+	+	-	-	-	-
123	9612 *	58	Male	0	I	Normal	Absence	1	Nasal	70%	Normal	-	55	0	55	0	CT	WES	+	+	+	+	+	+	-	+	-	-
124	9631 *	52	Male	1	I	Normal	Absence	0	Nasal	80%	Normal	-	NA	NA	NA	NA	RT	WES	+	+	+	+	-	-	-	-	-	-
125	143 *	24	Male	1	II	Normal	Absence	0	Nasal	70%	Elevated	-	NA	NA	NA	NA	CT+RT	WES	+	+	+	+	+	+	-	+/-	-	+
126	145 *	62	Female	2	IV	Elevated	Absence	4	Nasal	90%	Normal	-	NA	NA	NA	NA	CT	WES	+	+	+	+	+	+	+	-	-	-
127	3334 *	33	Male	1	I	Normal	Absence	0	Nasal	70%	Normal	-	NA	NA	NA	NA	RT	WES	+	+	+	+	+	+	-	-	-	-

Abbreviations: Serum LDH, Serum lactate dehydrogenase; ECOG PS, Eastern Cooperative Oncology Group performance status; IPI, International Prognostic Index;
CT, Chemotherapy; RT, Radiotherapy;WES, Whole exome sequencing; TS, Target sequencing. ＊, Samples without matched normal tissue;
EBER, Epstein-Barr virus (EBV)-encoded small RNAs.

Supplementary Table 2. Summary of whole-exome sequencing data of the 28 cases of NKTCL.

Patient ID*	Average sequencing depth on target	Coverage of target region**	Fraction of target covered with at least 20×	Fraction of target covered with at least 10×
149	104.09	98.70%	94.00%	96.70%
151	81.89	98.80%	94.10%	97.10%
3324	64.94	99.40%	92.70%	97.40%
3325	67.47	99.30%	93.70%	97.60%
3326	43.42	99.50%	80.00%	94.60%
3327	45.1	99.20%	83.40%	94.80%
3329	64.29	99.50%	91.20%	97.20%
3336	69.78	99.60%	92.50%	97.70%
3337	93.78	96.90%	64.10%	76.40%
8184	132.07	99.70%	94.30%	97.90%
8186	111.53	99.60%	95.40%	98.20%
8188	126.94	99.70%	96.00%	98.70%
8191	110.72	99.60%	94.10%	97.70%
8194	75.18	99.60%	92.10%	97.30%
8198	70.54	99.60%	90.00%	96.70%
8199	69.89	99.70%	92.40%	97.70%
8208	124.09	99.70%	95.70%	98.50%
8210	97.68	99.60%	95.10%	98.10%
8219	195.53	99.80%	96.70%	99.00%
8221	148.05	99.60%	97.20%	98.70%
8751	90.78	99.50%	91.10%	96.70%
9616	80.13	99.60%	90.70%	96.80%
9622	62.49	99.40%	88.80%	96.30%
9623	69.33	99.30%	90.70%	96.60%
9624	92.56	99.60%	92.80%	97.60%
9626	95.26	99.40%	93.10%	97.30%
9634	153.9	99.60%	95.20%	98.30%
9638	101.66	99.50%	87.10%	95.30%
N149	76.18	99.80%	91.50%	96.90%
N151	77.64	99.70%	91.00%	96.50%
N3325	70.92	99.60%	90.00%	96.10%
N3326	66.53	99.70%	88.70%	95.70%
N3329	76.78	99.70%	90.60%	96.40%
N3336	76.31	99.70%	91.00%	96.60%
N3337	78.44	99.60%	91.20%	96.40%
N9634	80.13	99.50%	95.40%	98.20%
N9638	68.16	99.50%	93.40%	97.80%
N9622	71.45	99.40%	94.40%	97.80%
N8208	84.8	99.60%	95.20%	98.20%
N8198	82.77	99.50%	95.00%	98.20%
N3324	66.75	99.50%	93.00%	97.70%
N3327	77.15	99.40%	94.70%	97.90%
N8186	67.06	99.30%	93.50%	97.60%
N9616	59.33	99.80%	80.40%	93.60%
N8184	75.45	99.70%	90.00%	96.00%
N8188	76.86	99.70%	91.40%	96.70%
N8191	77.31	99.80%	91.40%	96.80%
N8194	80.11	99.80%	91.70%	96.80%
N8199	75.4	99.70%	90.40%	96.30%
N8210	75.72	99.80%	91.10%	96.70%

N8219	74.7	99.80%	90.90%	96.50%
N8221	77.4	99.70%	91.50%	96.70%
N8751	76.41	99.70%	91.20%	96.60%
N9623	78.1	99.70%	90.70%	96.30%
N9624	74.75	99.70%	90.30%	96.20%
N9626	75.65	99.60%	91.10%	96.60%

*N, normal.
**Based on NCBI human reference genome GRC Build 37 (hg19).

Supplementary Table 3. List of somatic non-silent mutations identified by whole exome sequencing of 28 cases of NKTCL.

Patient ID	Hugo Symbol	Entrez Gene Id	Chromosome	Position*	Variant Classification	Reference Allele	Tumor Allele	Variant allele frequency	Protein Change
3337	*ABCF2*	10061	7	150919617	Nonsense	C	A	0.2	p.E270X
3337	*ACTR8*	93973	3	53902893	Splice_Site	C	A	0.143	c.1732-4G>T
3337	*ADCK2*	90956	7	140373434	Missense	C	T	0.267	p.R102C
3337	*AGAP5*	729092	10	75434752	Missense	C	T	0.4	p.V556I
3337	*AHNAK2*	113146	14	105414800	Missense	C	A	0.333	p.V2330L
3337	*ANAPC7*	51434	12	110841410	Missense	C	G	0.246	p.R42P
3337	*B4GALT6*	9331	18	29205630	Missense	T	C	0.263	p.T366A
3337	*BMP2K*	55589	4	79793795	Missense	C	A	0.25	p.Q546K
3337	*BOD1L1*	259282	4	13588088	Missense	C	A	0.4	p.D2789Y
3337	*CACNA1C*	775	12	2224512	Missense	G	A	0.208	p.A58T
3337	*CCL23*	6368	17	34340828	Missense	C	A	0.105	p.E69D
3337	*CHD2*	1106	15	93489428	Missense	C	G	0.333	p.I453M
3337	*CMYA5*	202333	5	79025711	Missense	G	A	0.25	p.E375K
3337	*CNTROB*	116840	17	7836585	Missense	C	T	0.07	p.P63L
3337	*COL5A1*	1289	9	137582762	Missense	G	T	0.097	p.Q38H
3337	*CSTF2T*	23283	10	53457663	In_Frame_Del	ATACCTCC	C	0.056	p.544_549del
3337	*CTSG*	1511	14	25042898	Missense	A	C	0.298	p.I238R
3337	*CTTNBP2*	83992	7	117450848	Missense	G	T	0.182	p.L129M
3337	*CTU2*	348180	16	88779247	Missense	C	A	0.049	p.A224D
3337	*DDX60L*	91351	4	169353603	Splice_Site	C	A	0.4	c.1516+3G>T
3337	*DMC1*	11144	22	38917651	Frame_Shift_Del	CT	C	0.129	p.K305fs
3337	*DOCK2*	1794	5	169412829	Splice_Site	C	A	0.167	c.2899-3C>A
3337	*DPYS*	1807	8	105456535	Missense	T	C	0.088	p.Y245C
3337	*E2F7*	144455	12	77423695	Missense	C	A	0.278	p.R600S
3337	*EP300*	2033	22	41546191	Missense	C	A	0.154	p.P936T
3337	*FAM186B*	84070	12	49994360	Nonsense	C	A	0.068	p.E355X
3337	*FAM209A*	200232	20	55100949	Missense	C	A	0.111	p.F113L
3337	*FAT4*	79633	4	126370253	Missense	C	A	0.182	p.D2694E
3337	*FCN3*	8547	1	27697158	Missense	G	A	0.234	p.A196V
3337	*FOXA1*	3169	14	38060721	Missense	G	A	0.264	p.A423V
3337	*GNAS*	2778	20	57429244	Missense	G	A	0.138	p.R246H
3337	*GPAT2*	150763	2	96688961	Missense	G	A	0.121	p.S681L
3337	*HGC6.3*	100128124	6	168377136	Missense	T	G	0.176	p.Q66P
3337	*HGC6.3*	100128124	6	168377262	Missense	T	G	0.091	p.Q24P
3337	*HTR1B*	3351	6	78172216	Missense	T	A	0.212	p.K302M
3337	*IL17C*	27189	16	88706430	Missense	T	G	0.216	p.F182V
3337	*KCNH7*	90134	2	163280034	Missense	C	A	0.154	p.A656S
3337	*L2HGDH*	79944	14	50735942	Missense	C	A	0.286	p.R282L
3337	*LHFPL2*	10184	5	77784941	Missense	C	A	0.133	p.A156S
3337	*MAN2B2*	23324	4	6599022	Missense	G	A	0.182	p.V414I
3337	*MKLN1*	4289	7	131113791	Splice_Site	G	T	0.333	c.848-1G>T
3337	*MMEL1*	79258	1	2528092	Missense	G	A	0.203	p.R437C
3337	*MPL*	4352	1	43805690	Missense	G	A	0.133	p.G249D
3337	*MUC21*	394263	6	30955179	Missense	G	C	0.2	p.E409D
3337	*MUC4*	4585	3	195501161	Missense	G	A	0.226	p.P4320L
3337	*MXRA7*	439921	17	74681213	Missense	C	A	0.077	p.K147N
3337	*MYH4*	4622	17	10363600	Missense	C	A	0.133	p.A396S
3337	*NKRF*	55922	X	118723880	Missense	G	A	0.231	p.A518V
3337	*NLRP14*	338323	11	7065044	Missense	G	T	0.182	p.S596I
3337	*NLRP3*	114548	1	247588293	Missense	G	T	0.224	p.K516N
3337	*NOS3*	4846	7	150698413	Missense	C	A	0.2	p.A443E
3337	*NRXN1*	9378	2	50847258	Missense	G	T	0.154	p.L448M
3337	*OR52M1*	119772	11	4566917	Missense	G	A	0.212	p.R166H
3337	*PDE4D*	5144	5	59189315	Missense	C	A	0.137	p.Q45H
3337	*PDZRN3*	23024	3	73432601	Missense	G	A	0.16	p.T1039M
3337	*POTEE*	445582	2	132021629	Missense	G	T	0.222	p.E867D
3337	*PRB4*	5545	12	11461769	Missense	G	T	0.114	p.P50T
3337	*PROB1*	389333	5	138727979	Missense	G	A	0.064	p.T931I
3337	*PROC*	5624	2	128184745	Missense	T	G	0.308	p.V248G
3337	*PTH1R*	5745	3	46940276	Missense	C	A	0.319	p.R255S
3337	*PUS10*	150962	2	61180157	Missense	T	C	0.5	p.D428G
3337	*RNF125*	54941	18	29617143	Missense	G	T	0.167	p.A77S
3337	*RNU6-28P*	100873756	15	43733730	Missense	A	G	0.25	p.V1026A
3337	*RSBN1L*	222194	7	77407690	Missense	G	T	0.286	p.C610F
3337	*SEPT5-GP1BB*	100526833	22	19711652	Missense	C	A	0.107	p.R96S
3337	*SIGLEC8*	27181	19	51961440	Missense	G	A	0.229	p.R68W
3337	*SLIT3*	6586	5	168093655	Missense	C	T	0.157	p.R1459H
3337	*SRRM5*	100170229	19	44117884	Missense	A	C	0.164	p.R537S
3337	*ST5*	6764	11	8752362	Missense	C	T	0.167	p.A159T
3337	*STAM*	8027	10	17756762	Nonsense	C	T	0.245	p.Q536X
3337	*TCFL5*	10732	20	61492781	Missense	A	G	0.13	p.L81P
3337	*TCN1*	6947	11	59620714	Missense	T	C	0.222	p.Y401C
3337	*TDG*	6996	12	104376700	Missense	A	C	0.15	p.K201T
3337	*TIGD2*	166815	4	90034606	Nonsense	G	T	0.333	p.E161X

3337	*TMEM14B*	81853	6	10756728	Missense	C	T	0.222	p.R108C
3337	*USP44*	84101	12	95927762	Missense	T	C	0.167	p.T91A
3337	*ZBTB7C*	201501	18	45567049	Missense	C	T	0.174	p.D144N
3337	*ZFHX2*	85446	14	23996919	Missense	C	T	0.239	p.R1010K
3336	*ANKRD22*	118932	10	90591731	Missense	A	C	0.55	p.V25G
3336	*ARID3A*	1820	19	965056	Missense	A	C	0.444	p.T392P
3336	*ASB18*	401036	2	237149941	Missense	C	T	0.052	p.A104T
3336	*BCAS1*	8537	20	52645166	Missense	A	G	0.043	p.V163A
3336	*CADM2*	253559	3	85961603	Missense	A	G	0.328	p.S87G
3336	*CASP8*	841	2	202134279	Nonsense	C	T	0.29	p.Q118X
3336	*CES2*	8824	16	66969362	Missense	C	T	0.05	p.R6C
3336	*CLIC6*	54102	21	36042964	Missense	G	C	0.328	p.S426T
3336	*COPS3*	8533	17	17179481	Splice_Site	G	A	0.077	c.56-3C>T
3336	*CYP4A11*	1579	1	47395874	Missense	A	C	0.136	p.I491M
3336	*DENND4C*	55667	9	19346689	Nonsense	G	T	0.134	p.E1259X
3336	*DHRS4L1*	728635	14	24520160	Missense	A	C	0.474	p.T146P
3336	*E2F7*	144455	12	77436967	Missense	C	T	0.067	p.R334H
3336	*EMC2*	9694	8	109488176	Missense	G	A	0.154	p.C192Y
3336	*FCGBP*	8857	19	40373950	Missense	G	T	0.154	p.T4043N
3336	*FRY*	10129	13	32709038	Splice_Site	C	A	0.25	c.886-3C>A
3336	*FSIP2*	401024	2	186667513	Frame_Shift_Ins	C	CA	0.208	p.Q4583fs
3336	*HLA-B*	3106	6	31323238	Missense	C	T	0.348	p.D251N
3336	*HMCN1*	83872	1	185964204	Missense	A	C	0.24	p.T1255P
3336	*HRNR*	388697	1	152192396	Missense	C	T	0.5	p.S570N
3336	*LPCAT2*	54947	16	55562466	Missense	G	A	0.06	p.M163I
3336	*MUC4*	4585	3	195497174	Missense	C	G	0.057	p.M4437I
3336	*MUC7*	4589	4	71347240	Missense	T	C	0.177	p.V260A
3336	*MYOM2*	9172	8	2092748	Missense	G	A	0.269	p.G1414D
3336	*NAALADL2*	254827	3	174814755	Missense	C	A	0.262	p.N73K
3336	*NEO1*	4756	15	73590698	Missense	G	A	0.165	p.R1304Q
3336	*NOTCH1*	4851	9	139409115	Missense	T	G	0.071	p.N685T
3336	*OR4A15*	81328	11	55135435	Missense	C	T	0.055	p.P26S
3336	*PCNXL4*	64430	14	60582053	Missense	G	A	0.043	p.V177I
3336	*PDGFRB*	5159	5	149500554	Missense	G	A	0.355	p.A828V
3336	*PKHD1*	5314	6	51889653	Missense	T	C	0.299	p.Y1652C
3336	*PLEKHN1*	84069	1	907758	Missense	A	G	0.214	p.E319G
3336	*PRG4*	10216	1	186276052	Missense	A	C	0.333	p.T401P
3336	*QRICH1*	54870	3	49084547	Missense	C	A	0.182	p.A491S
3336	*RRP7A*	27341	22	42910199	Missense	G	A	0.152	p.R224W
3336	*RSPO4*	343637	20	941023	Missense	G	A	0.341	p.R228C
3336	*SLC30A1*	7779	1	211751948	Missense	A	C	0.281	p.C3G
3336	*SLU7*	10569	5	159835658	Missense	A	G	0.083	p.M229T
3336	*SORL1*	6653	11	121458827	Missense	G	A	0.237	p.G1305R
3336	*SPTA1*	6708	1	158585142	Missense	C	T	0.141	p.D2218N
3336	*STAT3*	6774	17	40486030	Missense	G	T	0.357	p.Q279K
3336	*SYNJ2*	8871	6	158497754	Missense	A	C	0.48	p.T797P
3336	*TIGD2*	166815	4	90035549	Missense	A	G	0.061	p.H475R
3336	*TTN-AS1*	100506866	2	179456054	Nonsense	G	C	0.07	p.S20133X
3336	*VAX1*	11023	10	118897494	Missense	G	C	0.333	p.A25G
3336	*WWC1*	23286	5	167798475	Missense	C	T	0.221	p.P56S
3336	*ZBTB42*	100128927	14	105267770	Missense	T	A	0.313	p.L79Q
3336	*ZNF444*	55311	19	56669869	Missense	G	A	0.188	p.A102T
3336	*ZNF596*	169270	8	196274	Missense	T	G	0.091	p.V476G
3336	*ZSCAN1*	284312	19	58565342	Missense	G	A	0.367	p.V384I
3329	*ABLIM3*	22885	5	148619355	Missense	C	T	0.096	p.R370W
3329	*ACSM6*	142827	10	96985105	Missense	G	A	0.133	p.R420H
3329	*ACTRT2*	140625	1	2938807	Missense	G	A	0.107	p.G186D
3329	*AFF3*	3899	2	100199429	Frame_Shift_Ins	A	AT	0.147	p.M875fs
3329	*AGER*	177	6	32151050	Splice_Site	C	T	0.066	c.468+3G>A
3329	*ANKRD50*	57182	4	125591596	Missense	C	A	0.09	p.G946C
3329	*ARHGEF10*	9639	8	1791593	Missense	G	A	0.25	p.A10T
3329	*BAI3*	577	6	70071146	Missense	A	T	0.087	p.K1327N
3329	*BCL9L*	283149	11	118770860	Missense	C	T	0.222	p.A1058T
3329	*BEND2*	139105	X	18230715	Missense	A	C	0.136	p.F154L
3329	*BNIP3*	664	10	133787327	Missense	C	T	0.5	p.R56Q
3329	*C15orf43*	145645	15	45253735	Missense	A	T	0.139	p.I101F
3329	*C20orf144*	128864	20	32251404	Missense	G	C	0.061	p.D65H
3329	*CCDC50*	152137	3	191093310	Missense	A	G	0.073	p.K303R
3329	*CCDC73*	493860	11	32676427	Missense	T	C	0.267	p.E246G
3329	*CDH3*	1001	16	68713829	Missense	C	A	0.118	p.H273Q
3329	*CLCNKA*	1187	1	16357022	Missense	C	T	0.167	p.T492I
3329	*DHRS4L1*	728635	14	24520160	Missense	A	C	0.615	p.T146P
3329	*DICER1*	23405	14	95569720	Missense	G	A	0.07	p.A1338V
3329	*DMGDH*	29958	5	78326750	Missense	G	A	0.143	p.A530V
3329	*ENAH*	55740	1	225707016	Missense	C	A	0.103	p.R229I
3329	*FAM205A*	259308	9	34723988	Missense	C	A	0.4	p.R1083S
3329	*FCHSD2*	9873	11	72712136	Nonsense	G	A	0.077	p.Q96X
3329	*FKBP15*	23307	9	115936793	Missense	C	T	0.071	p.R765H
3329	*GHSR*	2693	3	172163018	Missense	T	C	0.154	p.Q345R
3329	*GPR179*	440435	17	36483837	Missense	G	A	0.096	p.T1872I

3329	*HARS2*	23438	5	140076953	Missense	G	A	0.099	p.V387I
3329	*HSPG2*	3339	1	22213759	Missense	G	A	0.07	p.R343C
3329	*IGFN1*	91156	1	201175274	Missense	A	G	0.091	p.Q418R
3329	*IKZF1*	10320	7	50450292	Missense	A	G	0.062	p.N159S
3329	*IL22RA1*	58985	1	24447468	Missense	G	C	0.077	p.R518G
3329	*JAKMIP2*	9832	5	147040551	Missense	G	A	0.3	p.S196L
3329	*JMJD1C*	221037	10	65140170	Missense	A	C	0.095	p.F81V
3329	*KCNIP1*	30820	5	170148846	Missense	C	T	0.06	p.T61M
3329	*KCNT1*	57582	9	138662241	Missense	C	T	0.074	p.R573C
3329	*KIAA1683*	80726	19	18377496	Missense	C	G	0.045	p.S285T
3329	*KRT38*	8687	17	39595484	Nonsense	G	A	0.225	p.Q235X
3329	*KRTAP4-9*	100132386	17	39262047	Missense	A	G	0.25	p.K136R
3329	*.OC10050705.*	100507053	4	100134863	Missense	C	A	0.153	p.L54F
3329	*MAGEC1*	9947	X	140993875	Missense	G	C	0.5	p.A229P
3329	*MDGA2*	161357	14	47566315	Missense	T	C	0.116	p.I15V
3329	*MRGPRX1*	259249	11	18956196	Missense	C	T	0.125	p.A46T
3329	*MUC16*	94025	19	9058907	Missense	G	C	0.078	p.H9513Q
3329	*MUC16*	94025	19	9076083	Missense	G	A	0.069	p.T3788I
3329	*MYO15A*	51168	17	18069672	Splice_Site	C	A	0.2	c.9788-3C>A
3329	*NDST1*	3340	5	149924975	Missense	C	A	0.075	p.A691E
3329	*NOTCH2*	4853	1	120539668	Missense	T	A	0.75	p.T235S
3329	*OR1S2*	219958	11	57970830	Missense	G	A	0.111	p.P275L
3329	*OR52E6*	390078	11	5862984	Missense	G	C	0.071	p.F48L
3329	*OR56A1*	120796	11	6048642	Missense	G	A	0.133	p.S98L
3329	*PABPC1*	26986	8	101724606	Missense	G	A	0.118	p.T319I
3329	*PABPC1L*	80336	20	43561710	Splice_Site	C	T	0.107	c.1331-3C>T
3329	*PCDHA11*	56138	5	140249266	Missense	C	T	0.088	p.S193L
3329	*PCDHA9*	9752	5	140263778	Missense	C	T	0.092	p.A642V
3329	*PLAT*	5327	8	42045064	Missense	G	T	0.136	p.Q131K
3329	*PLCB3*	5331	11	64029509	Missense	G	A	0.082	p.V667I
3329	*POM121L12*	285877	7	53104193	Missense	A	T	0.075	p.S277C
3329	*PPP1R26*	9858	9	138378218	Missense	C	T	0.091	p.A621V
3329	*PRRC2A*	7916	6	31604011	Missense	G	A	0.052	p.G1884S
3329	*RP1L1*	94137	8	10468172	Missense	G	A	0.069	p.R1146W
3329	*SLC15A1*	6564	13	99358482	Missense	C	A	0.118	p.G392V
3329	*SORL1*	6653	11	121403182	Missense	G	A	0.111	p.G536R
3329	*STC1*	6781	8	23709782	Missense	C	A	0.105	p.L78F
3329	*SUDS3*	64426	12	118852225	Missense	G	A	0.133	p.R325H
3329	*TACC2*	10579	10	123989927	Missense	T	G	0.061	p.L2700W
3329	*TGS1*	96764	8	56705333	Missense	T	A	0.146	p.S453T
3329	*TMEM121*	80757	14	105995790	Missense	G	A	0.143	p.E207K
3329	*TMEM63C*	57156	14	77706020	Missense	A	C	0.292	p.H294P
3329	*TRIOBP*	11078	22	38121152	Missense	C	A	0.062	p.N863K
3329	*TSSK2*	23617	22	19119751	Missense	C	T	0.064	p.T280M
3329	*TUBD1*	51174	17	57963537	Missense	A	G	0.182	p.M76T
3329	*UBR7*	55148	14	93684890	Nonsense	G	T	0.154	p.E207X
3329	*USH2A*	7399	1	215960153	Missense	A	C	0.304	p.C3416G
3329	*WDR43*	23160	2	29150552	Missense	T	G	0.178	p.S431A
3329	*WDR59*	79726	16	74926444	Nonsense	G	A	0.167	p.Q674X
3329	*YEATS2*	55689	3	183474408	Missense	A	G	0.178	p.I495V
3329	*ZMAT1*	84460	X	101139254	Missense	G	T	0.15	p.P211Q
3329	*ZNF335*	63925	20	44596429	Missense	C	T	0.125	p.R253Q
3329	*ZNF83*	55769	19	53117469	Missense	T	C	0.075	p.K117E
3327	*ABCC8*	6833	11	17414592	Missense	C	A	0.192	p.K1565N
3327	*ABCG1*	9619	21	43710302	Missense	C	T	0.105	p.A434V
3327	*ACAN*	176	15	89392960	Missense	G	A	0.048	p.R675Q
3327	*ACSBG1*	23205	15	78466127	Missense	T	C	0.075	p.M633V
3327	*ACTB*	60	7	5568970	Missense	C	A	0.111	p.R62I
3327	*AIM1L*	55057	1	26673108	Missense	C	T	0.079	p.R14Q
3327	*APC2*	10297	19	1453137	Missense	T	C	0.35	p.M46T
3327	*ARPP21*	10777	3	35779794	Missense	C	T	0.184	p.T544M
3327	*ATRX*	546	X	76931736	Missense	T	C	0.133	p.N1265S
3327	*ATXN7L1*	222255	7	105254613	Missense	G	A	0.257	p.P723L
3327	*BAIAP2*	10458	17	79082271	Splice_Site	C	T	0.176	c.1501-4C>T
3327	*BCYRN1*	618	X	70837255	Frame_Shift_Del	AGTTCT	A	0.167	p.E21fs
3327	*BCYRN1*	618	X	70837252	Frame_Shift_Ins	T	TGAAA	0.158	p.S24fs
3327	*CCDC116*	164592	22	21988599	Missense	G	A	0.094	p.G121R
3327	*CCT4*	10575	2	62099221	Missense	C	T	0.167	p.R496Q
3327	*CDH9*	1007	5	26988328	Missense	G	A	0.087	p.A38V
3327	*CEP192*	55125	18	13056682	Missense	G	A	0.154	p.V1365M
3327	*CEP55*	55165	10	95276742	Missense	G	C	0.103	p.A244P
3327	*CFAP46*	54777	10	134646913	Nonsense	G	A	0.133	p.R2356X
3327	*CLASP1*	23332	2	122218726	Missense	C	T	0.095	p.R328K
3327	*CLOCK*	9575	4	56314986	Missense	G	A	0.091	p.S500F
3327	*CMYA5*	202333	5	79026773	Missense	T	C	0.231	p.Y729H
3327	*CNST*	163882	1	246784733	Missense	C	A	0.333	p.L128I
3327	*COL4A2*	1284	13	111145633	Splice_Site	C	T	0.111	c.3634+4C>T
3327	*CPN1*	1369	10	101835685	Missense	C	T	0.2	p.E135K
3327	*CXorf67*	340602	X	51150445	Missense	A	C	0.296	p.S193R
3327	*DES*	1674	2	220283420	Missense	C	T	0.304	p.T79M

3327	*DFFA*	1676	1	10532419	Missense	C	A	0.343	p.G33C
3327	*DHX58*	79132	17	40262767	Missense	C	T	0.222	p.D179N
3327	*DIAPH3*	81624	13	60544092	Missense	C	A	0.133	p.C687F
3327	*EFS*	10278	14	23829242	Missense	C	T	0.138	p.D149N
3327	*ETV1*	2115	7	13940418	Missense	T	G	0.143	p.Y386S
3327	*EVC2*	132884	4	5690902	Missense	T	C	0.07	p.S230G
3327	*EXOC3L4*	91828	14	103566731	Missense	C	T	0.167	p.R59W
3327	*FAM186B*	84070	12	49994472	Missense	C	A	0.216	p.Q317H
3327	*FAM47C*	442444	X	37026830	Missense	C	T	0.667	p.A116V
3327	*FBXO32*	114907	8	124518679	Missense	G	A	0.4	p.R263W
3327	*FGF2*	2247	4	123814308	Missense	C	T	0.048	p.R40Q
3327	*FOXP1*	27086	3	71008447	Missense	T	A	0.148	p.D662V
3327	*FRY*	10129	13	32676096	Splice_Site	C	T	0.111	c.271-4C>T
3327	*FSIP2*	401024	2	186667115	Missense	C	T	0.082	p.A4450V
3327	*GAREM*	64762	18	29848570	Missense	C	T	0.119	p.G631E
3327	*GOLGA2*	2801	9	131019738	Missense	T	C	0.143	p.R902G
3327	*GPRIN1*	114787	5	176026795	Missense	T	C	0.231	p.Q14R
3327	*HABP2*	3026	10	115341758	Missense	C	T	0.188	p.T321M
3327	*HAS2*	3037	8	122641081	Missense	C	A	0.098	p.S167I
3327	*HMHA1*	23526	19	1068738	Missense	G	A	0.062	p.R139H
3327	*HPD*	3242	12	122285073	Missense	G	T	0.262	p.T176K
3327	*HRNR*	388697	1	152193944	Missense	A	T	0.286	p.V54E
3327	*ICAM5*	7087	19	10403368	Missense	G	A	0.143	p.A348T
3327	*IGFLR1*	79713	19	36231312	Missense	C	G	0.273	p.G104A
3327	*ITGAE*	3682	17	3628212	Missense	T	C	0.087	p.I328T
3327	*KCNV2*	169522	9	2718209	Missense	T	C	0.161	p.V157A
3327	*KIAA1467*	57613	12	13220112	Missense	C	T	0.114	p.R342W
3327	*KIF5C*	3800	2	149866665	Missense	C	T	0.258	p.A856V
3327	*KIRREL3*	84623	11	126396493	Missense	C	T	0.097	p.D75N
3327	*KLHL30*	377007	2	239049642	Missense	G	A	0.231	p.V83M
3327	*KLHL40*	131377	3	42729655	Missense	T	A	0.333	p.W392R
3327	*LAMA5*	3911	20	60898710	Missense	A	G	0.2	p.F1956L
3327	*LIN7A*	8825	12	81205289	Missense	C	A	0.107	p.Q219H
3327	*.OC10192776:*	101927765	1	234509259	Missense	G	C	0.1	p.S16T
3327	*LRP6*	4040	12	12283719	Missense	C	T	0.154	p.C1360Y
3327	*LSM10*	84967	1	36859418	Nonsense	G	A	0.138	p.R105X
3327	*MCM8*	84515	20	5935790	Missense	G	A	0.1	p.E127K
3327	*MMP26*	56547	11	5013190	Splice_Site	T	C	0.222	c.596-4T>C
3327	*MRPL12*	6182	17	79674250	Missense	G	A	0.25	p.G193S
3327	*MS4A14*	84689	11	60184191	Missense	G	A	0.094	p.G617R
3327	*MSN*	4478	X	64953045	Splice_Site	G	A	0.273	c.699-1G>A
3327	*MUC5B*	727897	11	1260149	Missense	T	C	0.188	p.C1116R
3327	*MYO7A*	4647	11	76924915	Missense	C	A	0.227	p.T2110N
3327	*NALCN*	259232	13	102029124	Missense	G	T	0.094	p.L191I
3327	*NAV3*	89795	12	78574732	Missense	T	C	0.138	p.S1867P
3327	*NOV*	4856	8	120428860	Splice_Site	A	G	0.16	c.84+4A>G
3327	*NSD1*	64324	5	176710863	Missense	A	G	0.176	p.T1760A
3327	*OR4N5*	390437	14	20612441	Nonsense	C	T	0.151	p.Q183X
3327	*OR5D18*	219438	11	55587715	Missense	G	A	0.132	p.A204T
3327	*PCDHB2*	56133	5	140475658	Missense	C	G	0.136	p.F428L
3327	*PCOLCE2*	26577	3	142567248	Missense	G	A	0.142	p.R87C
3327	*PELI3*	246330	11	66241386	Missense	G	A	0.06	p.R277Q
3327	*PHKG2*	5261	16	30768183	Missense	G	A	0.071	p.R329Q
3327	*PRKCE*	5581	2	46372368	Missense	G	A	0.2	p.E577K
3327	*PTCHD4*	442213	6	47846106	Missense	C	T	0.206	p.R825Q
3327	*PTPRQ*	374462	12	81064141	Missense	G	C	0.265	p.V1986L
3327	*PVRL2*	5819	19	45381597	In_Frame_Del	TGAG	T	0.12	p.387_388del
3327	*QRFPR*	84109	4	122301741	Missense	G	A	0.235	p.T21M
3327	*REPS2*	9185	X	17086525	Missense	A	G	0.455	p.K404E
3327	*RGAG1*	57529	X	109693960	Missense	G	A	0.167	p.V39I
3327	*RP1L1*	94137	8	10468858	Missense	G	A	0.25	p.A917V
3327	*SIDT2*	51092	11	117062663	In_Frame_Del	TCAA	T	0.117	p.602_603del
3327	*SLC22A23*	63027	6	3273371	Missense	T	C	0.164	p.D379G
3327	*SMOC1*	64093	14	70480183	Missense	T	G	0.25	p.S341A
3327	*TEX40*	25858	11	64070970	Missense	G	C	0.2	p.M123I
3327	*TNFAIP2*	7127	14	103593950	Missense	C	G	0.088	p.Q282E
3327	*VCAN*	1462	5	82816870	Missense	A	C	0.113	p.Q915H
3327	*WNT8B*	7479	10	102240866	Missense	G	T	0.348	p.R118L
3327	*WT1*	7490	11	32410647	Missense	T	G	0.097	p.N504T
3327	*ZC3H3*	23144	8	144623574	Missense	T	C	0.07	p.I6M
3327	*ZNF101*	94039	19	19790159	Missense	A	C	0.055	p.M121L
3327	*ZNF470*	388566	19	57089898	Missense	G	A	0.167	p.G701R
3327	*ZNF577*	84765	19	52376701	Missense	C	T	0.182	p.R181K
3327	*ZNF646*	9726	16	31088022	Missense	C	T	0.125	p.S126F
3327	*ZP1*	22917	11	60640911	Missense	C	A	0.065	p.P435Q
3326	*ANKH*	56172	5	14749408	Missense	G	T	0.167	p.A232E
3326	*ARFGAP1*	55738	20	61919086	Missense	C	T	0.055	p.S369L
3326	*ARHGEF18*	23370	19	7529491	Missense	G	A	0.133	p.R594Q
3326	*ARID3A*	1820	19	965056	Missense	A	C	0.467	p.T392P
3326	*ARMCX4*	100131755	X	100745825	Missense	T	C	0.25	p.L750P

3326	*ARMCX4*	100131755	X	100743826	Missense	A	G	0.2	p.R84G
3326	*ATF7*	11016	12	53946348	Missense	C	T	0.167	p.R41Q
3326	*BIRC5*	332	17	76212078	Missense	A	C	0.286	p.T85P
3326	*BRINP2*	57795	1	177250051	Missense	G	A	0.24	p.S580N
3326	*BSN*	8927	3	49688508	Missense	C	A	0.222	p.A661E
3326	*BSPRY*	54836	9	116124649	Missense	C	A	0.167	p.Q178K
3326	*C12orf50*	160419	12	88374118	Missense	C	G	0.089	p.G412A
3326	*C20orf194*	25943	20	3235874	Missense	C	A	0.125	p.R1106S
3326	*C8orf44*	56260	8	67590068	Missense	T	C	0.07	p.M42T
3326	*CALHM1*	255022	10	105217991	Missense	C	T	0.222	p.R173Q
3326	*'DC169-SOHL*	100526761	13	36744910	Missense	C	T	0.105	p.A416T
3326	*CD93*	22918	20	23065209	Missense	G	A	0.123	p.P541S
3326	*CDH8*	1006	16	61689413	Missense	C	T	0.2	p.A623T
3326	*CENPF*	1063	1	214815399	Missense	A	C	0.143	p.T1240P
3326	*CEP70*	80321	3	138290107	Splice_Site	C	A	0.182	c.160+1G>T
3326	*CFAP45*	25790	1	159842768	Missense	C	A	0.083	p.D515Y
3326	*CFHR4*	10877	1	196876458	Missense	A	G	0.12	p.N209S
3326	*CLDN17*	26285	21	31538656	Missense	C	T	0.068	p.A94T
3326	*CNTN4*	152330	3	2928746	Nonsense	C	T	0.385	p.R260X
3326	*COG5*	10466	7	107002806	Missense	A	G	0.125	p.F330L
3326	*CRACR2B*	283229	11	828916	Missense	G	A	0.095	p.R77Q
3326	*CTSG*	1511	14	25043671	Missense	T	C	0.07	p.N125S
3326	*DMKN*	93099	19	36004171	Missense	C	A	0.111	p.E69D
3326	*DOCK7*	85440	1	63021596	Missense	C	G	0.176	p.L832F
3326	*DSC3*	1825	18	28605748	Missense	C	T	0.118	p.R203H
3326	*EPG5*	57724	18	43490580	Missense	G	A	0.154	p.R1371C
3326	*FAM135A*	57579	6	71187020	Missense	A	C	0.3	p.H176P
3326	*FAM221B*	392307	9	35826038	Missense	T	C	0.08	p.K41E
3326	*FAT3*	120114	11	92535022	Missense	C	A	0.083	p.T2948N
3326	*FBXW5*	54461	9	139836103	Missense	G	A	0.103	p.T377M
3326	*FCRL3*	115352	1	157668390	Missense	T	C	0.097	p.N28D
3326	*FOXA2*	3170	20	22562697	Missense	G	T	0.192	p.H389N
3326	*FSIP2*	401024	2	186654867	Missense	G	C	0.12	p.D1091H
3326	*FSIP2*	401024	2	186654592	Missense	A	G	0.094	p.N999S
3326	*FSIP2*	401024	2	186664963	Missense	C	A	0.079	p.L3733I
3326	*FSIP2*	401024	2	186658438	Missense	C	T	0.07	p.T2281M
3326	*FXR1*	8087	3	180669158	Nonsense	G	T	0.111	p.G150X
3326	*GMPPA*	29926	2	220366590	Missense	C	T	0.086	p.A87V
3326	*GPR151*	134391	5	145895246	Missense	G	A	0.086	p.A144V
3326	*GPRIN3*	285513	4	90169925	Missense	A	G	0.081	p.V446A
3326	*HCN1*	348980	5	45645378	Missense	G	C	0.255	p.A253G
3326	*HDHD1*	8226	X	6975782	Missense	C	G	0.222	p.C198S
3326	*HHIPL1*	84439	14	100138655	Missense	C	T	0.267	p.R578W
3326	*HLX*	3142	1	221053545	Missense	T	C	0.075	p.S116P
3326	*HRNR*	388697	1	152192114	Missense	C	T	0.4	p.R664Q
3326	*IGFN1*	91156	1	201181217	Missense	A	G	0.2	p.D2399G
3326	*IGFN1*	91156	1	201195161	Missense	G	A	0.167	p.E3566K
3326	*JAG2*	3714	14	105615534	Missense	C	T	0.125	p.E576K
3326	*KLHL12*	59349	1	202887323	Missense	C	A	0.222	p.K181N
3326	*.OC10192916.*	101929162	12	108136024	Missense	T	C	0.406	p.Y528C
3326	*LRP1B*	53353	2	141625375	Missense	C	T	0.333	p.D1455N
3326	*LRRC17*	10234	7	102574999	In_Frame_Del	TGAA	T	0.15	p.214_214del
3326	*MAP6*	4135	11	75378924	Missense	G	T	0.238	p.P164Q
3326	*MIR3134*	100422990	9	114982463	Missense	T	C	0.068	p.T545A
3326	*MLH3*	27030	14	75513828	Missense	G	A	0.066	p.P844L
3326	*MTERF1*	7978	7	91503228	Missense	C	T	0.061	p.A274T
3326	*MTHFSD*	64779	16	86565826	Missense	C	G	0.067	p.G314R
3326	*MYLK3*	91807	16	46746678	Nonsense	G	A	0.222	p.R666X
3326	*NFAM1*	150372	22	42807746	Splice_Site	G	T	0.167	c.122-4C>A
3326	*NOL9*	79707	1	6593483	Missense	A	G	0.133	p.L365P
3326	*NSFL1C*	55968	20	1426393	Missense	C	T	0.25	p.D290N
3326	*OR10T2*	128360	1	158368964	Missense	C	T	0.086	p.C98Y
3326	*OR10T2*	128360	1	158368974	Missense	A	G	0.081	p.F95L
3326	*OR4L1*	122742	14	20528528	Missense	G	A	0.083	p.G109S
3326	*OR4L1*	122742	14	20528505	Missense	T	A	0.068	p.M101K
3326	*OR6N1*	128372	1	158736445	Missense	C	T	0.097	p.A10T
3326	*OVGP1*	5016	1	111957311	Missense	G	C	0.091	p.H604Q
3326	*PARP10*	84875	8	145057230	Missense	G	A	0.111	p.A809V
3326	*PCDHA2*	56146	5	140183058	Missense	G	A	0.1	p.C759Y
3326	*PCDHB7*	56129	5	140553581	Missense	G	C	0.125	p.V389L
3326	*PGBD3*	267004	10	50732280	Missense	C	T	0.075	p.G399D
3326	*PILRA*	29992	7	99971834	Missense	A	G	0.067	p.R78G
3326	*PLXNB2*	23654	22	50717129	Splice_Site	T	G	0.118	c.4547-4A>C
3326	*PLXNB2*	23654	22	50728062	Missense	T	C	0.05	p.K318E
3326	*PODXL2*	50512	3	127388011	Missense	C	A	0.125	p.S464Y
3326	*POLN*	353497	4	2176454	Missense	G	A	0.25	p.R425C
3326	*PPP1R13L*	10848	19	45900076	Missense	C	T	0.167	p.D147N
3326	*PRR21*	643905	2	240981262	Missense	A	C	0.2	p.S380A
3326	*PRUNE*	58497	1	151006537	Missense	G	A	0.12	p.G397S
3326	*PTCHD2*	57540	1	11596501	Missense	G	C	0.143	p.A1313P

3326	*PYY*	5697	17	42030531	Missense	G	C	0.094	p.T72R
3326	*RETSAT*	54884	2	85578010	Nonsense	G	A	0.111	p.R164X
3326	*RNF43*	54894	17	56448297	Missense	C	T	0.107	p.R117H
3326	*RNF43*	54894	17	56492800	Missense	T	C	0.067	p.I47V
3326	*RTKN2*	219790	10	63958112	Missense	T	C	0.056	p.H462R
3326	*RTN2*	6253	19	45998110	Missense	C	T	0.065	p.R78H
3326	*SBSPON*	157869	8	73982044	Missense	C	T	0.667	p.D225N
3326	*SLC43A3*	29015	11	57177579	Missense	G	A	0.158	p.A359V
3326	*SLC6A5*	9152	11	20622975	Missense	G	A	0.06	p.G102S
3326	*SMAP1*	60682	6	71569090	Missense	T	C	0.079	p.M416T
3326	*ST6GAL2*	84620	2	107459974	Missense	C	T	0.071	p.G154R
3326	*ST6GALNAC5*	81849	1	77528748	Missense	C	T	0.237	p.R290C
3326	*STAB1*	23166	3	52556890	Missense	A	G	0.103	p.I2282V
3326	*TAF13*	6884	1	109608851	Missense	C	T	0.2	p.R37Q
3326	*TBC1D26*	353149	17	15641610	Missense	A	G	1	p.Y99C
3326	*TDGF1*	6997	3	46620614	Missense	T	C	0.081	p.V22A
3326	*TFIP11*	24144	22	26894831	Splice_Site	G	A	0.286	c.1436+4C>T
3326	*TMEM248*	55069	7	66415947	Missense	C	T	0.214	p.P202L
3326	*TNFRSF1A*	7132	12	6438603	Missense	C	T	0.06	p.E415K
3326	*TRAM1L1*	133022	4	118005505	Missense	C	T	0.333	p.G349R
3326	*UBE2F-SCLY*	100533179	2	238978059	Missense	C	T	0.07	p.S150L
3326	*UBE2Q2*	92912	15	76165830	Missense	C	T	0.2	p.S170L
3326	*UNC80*	285175	2	210685216	Missense	C	A	0.147	p.A715D
3326	*VWDE*	221806	7	12406989	Missense	C	G	0.064	p.K964N
3326	*WDR60*	55112	7	158672580	Missense	G	A	0.143	p.R260Q
3326	*WNT16*	51384	7	120979089	Missense	C	T	0.115	p.T263I
3326	*ZBTB11*	27107	3	101383562	Splice_Site	G	A	0.094	c.1624-4C>T
3326	*ZC3H12B*	340554	X	64722934	Missense	C	A	0.105	p.H786N
3326	*ZCCHC14*	23174	16	87445839	Missense	C	T	0.103	p.V693M
3326	*ZNF683*	257101	1	26691174	Missense	C	A	0.091	p.R288L
3326	*ZSCAN5B*	342933	19	56704366	Missense	C	T	0.088	p.G19E
3325	*ACVR2A*	92	2	148676144	Missense	A	C	0.25	p.K315N
3325	*ADAMTS7*	11173	15	79092760	Missense	T	C	0.387	p.D77G
3325	*AMDHD2*	51005	16	2578104	Missense	G	T	0.073	p.W224C
3325	*ANKRD22*	118932	10	90591731	Missense	A	C	0.36	p.V25G
3325	*ANKRD50*	57182	4	125591283	Missense	C	G	0.212	p.C1050S
3325	*AP5B1*	91056	11	65547333	Missense	G	A	0.043	p.L211F
3325	*AR*	367	X	66765161	Missense	A	T	0.062	p.Q58L
3325	*ATP6V0A2*	23545	12	124203252	Splice_Site	A	G	0.297	c.196+4A>G
3325	*BDNF-AS*	497258	11	27679916	Missense	C	T	0.039	p.V66M
3325	*C16orf95*	100506581	16	87350773	Missense	C	A	0.083	p.A26S
3325	*C17orf112*	100506650	17	51063874	Missense	G	C	0.174	p.G40R
3325	*C2orf54*	79919	2	241831296	Missense	C	G	0.091	p.A21P
3325	*CACHD1*	57685	1	65145363	Missense	G	C	0.231	p.L1059F
3325	*CCER1*	196477	12	91348075	Missense	C	T	0.194	p.G149S
3325	*CDC42EP3*	10602	2	37873636	Missense	C	T	0.235	p.S32N
3325	*CEP55*	55165	10	95279506	Missense	A	T	0.093	p.H378L
3325	*CETN1*	1068	18	580623	Missense	T	C	0.066	p.M72T
3325	*CGNL1*	84952	15	57730888	Missense	A	G	0.261	p.T231A
3325	*COL6A5*	256076	3	130188092	Missense	C	T	0.259	p.A2415V
3325	*CRB2*	286204	9	126133684	Missense	G	A	0.062	p.D755N
3325	*CYP4A11*	1579	1	47395874	Missense	A	C	0.154	p.I491M
3325	*DACT2*	168002	6	168708816	Missense	T	G	0.125	p.T541P
3325	*DAPK1*	1612	9	90258316	Missense	G	A	0.241	p.C315Y
3325	*DDX26B*	203522	X	134713724	Missense	A	G	0.207	p.I674V
3325	*DDX60L*	91351	4	169362557	Missense	C	G	0.07	p.V409L
3325	*DHRS4L1*	728635	14	24520160	Missense	A	C	0.875	p.T146P
3325	*EPHB4*	2050	7	100417778	Missense	C	T	0.192	p.G317S
3325	*FAM135A*	57579	6	71187020	Missense	A	C	0.264	p.H176P
3325	*FAM83A*	84985	8	124206324	Missense	G	A	0.122	p.A237T
3325	*FAT1*	2195	4	187629624	Missense	A	G	0.184	p.V453A
3325	*FGFR4*	2264	5	176522367	Missense	C	T	0.229	p.S519L
3325	*FGG*	2266	4	155533013	Missense	C	A	0.255	p.M115I
3325	*FRMPD1*	22844	9	37745747	Missense	G	T	0.309	p.A1240S
3325	*FRY*	10129	13	32808741	Missense	T	A	0.185	p.V1853D
3325	*GFRAL*	389400	6	55266625	Missense	T	C	0.071	p.S387P
3325	*GPATCH2*	55105	1	217804268	Missense	C	T	0.333	p.G16R
3325	*GRIN2D*	2906	19	48923009	Missense	C	A	0.475	p.L677M
3325	*GUCY1A2*	2977	11	106810251	Nonsense	G	A	0.278	p.R381X
3325	*HCAR3*	8843	12	123200527	Missense	T	C	0.625	p.H253R
3325	*HIST1H4D*	8360	6	26189076	Missense	C	T	0.042	p.A77T
3325	*HLA-B*	3106	6	31323309	Missense	C	A	0.278	p.C227F
3325	*HOXB7*	3217	17	46688256	Missense	T	C	0.049	p.T9A
3325	*HS6ST2*	90161	X	132092324	Nonsense	G	A	0.333	p.R103X
3325	*ITGA1*	3672	5	52145300	Nonsense	G	T	0.297	p.E55X
3325	*KALRN*	8997	3	123987830	Missense	A	C	0.155	p.T231P
3325	*KIAA1683*	80726	19	18377496	Missense	C	G	0.051	p.S285T
3325	*LRP3*	4037	19	33696931	Missense	G	A	0.506	p.D419N
3325	*MACF1*	23499	1	39844955	Missense	G	A	0.333	p.E2391K
3325	*MAP2*	4133	2	210560393	Missense	G	A	0.303	p.V1167I

3325	*MINK1*	50488	17	4788294	Missense	C	A	0.24	p.L169I
3325	*MMEL1*	79258	1	2526746	Missense	A	G	0.081	p.M518T
3325	*MOB2*	81532	11	1492591	Missense	C	T	0.304	p.V142I
3325	*MROH2B*	133558	5	41047824	Missense	T	C	0.167	p.Q576R
3325	*MSH6*	2956	2	48030639	Frame_Shift_Ins	A	AC	0.213	p.T783fs
3325	*MUC6*	4588	11	1017325	Missense	A	C	0.162	p.Y1826D
3325	*MUC7*	4589	4	71347171	Missense	C	T	0.16	p.A237V
3325	*MUC7*	4589	4	71347077	Missense	T	C	0.092	p.S206P
3325	*NFIB*	4781	9	14398569	Missense	T	C	0.32	p.N21S
3325	*NFIB*	4781	9	14113074	Missense	G	A	0.229	p.T464I
3325	*NRAP*	4892	10	115377290	Missense	T	C	0.125	p.D966G
3325	*NUGGC*	389643	8	27925204	Missense	T	C	0.044	p.S180G
3325	*OR51T1*	401665	11	4903802	Missense	C	T	0.265	p.R252C
3325	*OR56A3*	390083	11	5968900	Missense	G	A	0.309	p.M108I
3325	*OR56A3*	390083	11	5969451	Missense	A	G	0.229	p.N292S
3325	*OXTR*	5021	3	8809222	Missense	C	T	0.073	p.A218T
3325	*PGM1*	5236	1	64117428	Missense	A	G	0.308	p.K457E
3325	*PLSCR2*	57047	3	146179647	Missense	T	A	0.229	p.N5I
3325	*PRSS27*	83886	16	2762757	Missense	A	C	0.526	p.V246G
3325	*RBMS3*	27303	3	29628700	Splice_Site	A	C	0.306	c.399+4A>C
3325	*RNF43*	54894	17	56435885	Missense	G	T	0.091	p.L418M
3325	*RTFDC1*	51507	20	55093224	Nonsense	C	A	0.07	p.S305X
3325	*RTP5*	285093	2	242815059	Missense	C	T	0.071	p.A451V
3325	*RUNX1T1*	862	8	93004077	Nonsense	C	A	0.215	p.E234X
3325	*SGTA*	6449	19	2757459	Splice_Site	C	A	0.263	c.828-4G>T
3325	*SLC16A8*	23539	22	38477342	Missense	T	A	0.167	p.R235W
3325	*SLC22A13*	9390	3	38307697	Missense	C	A	0.167	p.P116T
3325	*SLC25A5*	292	X	118603925	Missense	G	A	0.165	p.R138H
3325	*SLC43A3*	29015	11	57185305	Missense	C	T	0.279	p.C196Y
3325	*SLITRK2*	84631	X	144905880	Missense	G	A	0.17	p.R646H
3325	*SMTN*	6525	22	31489858	Missense	G	T	0.111	p.S628I
3325	*SPTB*	6710	14	65241965	Missense	C	A	0.077	p.A1574S
3325	*SSPO*	23145	7	149486405	Missense	G	A	0.179	p.D1461N
3325	*THEG*	51298	19	362268	Missense	C	A	0.188	p.A358S
3325	*TIFAB*	497189	5	134785467	Missense	G	A	0.303	p.R55C
3325	*TKTL1*	8277	X	153553724	Missense	G	C	0.163	p.V453L
3325	*TLR4*	7099	9	120475863	Missense	A	T	0.275	p.N446I
3325	*TUBGCP3*	10426	13	113212589	Missense	C	T	0.167	p.V157M
3325	*WDR90*	197335	16	716273	Missense	T	C	0.041	p.C1555R
3325	*ZNF236*	7776	18	74637216	Missense	C	T	0.064	p.R1243C
3325	*ZNF441*	126068	19	11888453	Missense	A	G	0.25	p.I11V
3325	*ZNF831*	128611	20	57768743	Missense	C	T	0.068	p.A890V
3324	*ABCA8*	10351	17	66903989	Missense	T	G	0.3	p.N724H
3324	*ACSM6*	142827	10	96985105	Missense	G	A	0.278	p.R420H
3324	*ADAMTS16*	170690	5	5237162	Missense	A	G	0.344	p.T702A
3324	*ANK3*	288	10	61846611	Missense	T	G	0.456	p.K1192T
3324	*ANKRD24*	170961	19	4219622	Missense	G	A	0.071	p.R1013Q
3324	*ANKRD31*	256006	5	74400816	Missense	T	G	0.306	p.N1466T
3324	*APOA4*	337	11	116692334	Missense	C	T	0.07	p.S147N
3324	*ARHGAP28*	79822	18	6851058	Missense	C	G	0.079	p.T31S
3324	*ARHGAP29*	9411	1	94652126	Missense	G	A	0.51	p.T570I
3324	*ASCL4*	121549	12	108169146	Missense	C	T	0.387	p.R52C
3324	*BANK1*	55024	4	102984282	Missense	A	C	0.303	p.E733D
3324	*BTBD11*	121551	12	108045467	Missense	A	C	0.314	p.K1003T
3324	*C19orf73*	55150	19	49621964	Missense	T	C	0.077	p.S106G
3324	*C6orf58*	352999	6	127911369	Missense	T	G	0.295	p.L271R
3324	*CCNB2*	9133	15	59406996	Missense	T	C	0.1	p.L173P
3324	*CD109*	135228	6	74530219	Missense	C	G	0.364	p.A1365G
3324	*CELA3B*	23436	1	22307538	Missense	C	T	0.06	p.R79W
3324	*CLUH*	23277	17	2600186	Missense	G	A	0.128	p.A633V
3324	*CNTN1*	1272	12	41327330	Missense	A	C	0.288	p.Q257H
3324	*COBLL1*	22837	2	165548727	Splice_Site	T	G	0.222	c.3414+4A>C
3324	*COL22A1*	169044	8	139728466	Splice_Site	T	G	0.32	c.2463+4A>C
3324	*CPED1*	79974	7	120686989	Missense	C	T	0.38	p.A161V
3324	*CPNE8*	144402	12	39047772	Missense	A	G	0.409	p.L536P
3324	*CYP4F22*	126410	19	15636235	Missense	T	G	0.292	p.F30V
3324	*DDX3X*	1654	X	41204711	Missense	G	A	0.74	p.G409S
3324	*DGAT2L6*	347516	X	69419150	Missense	T	G	0.556	p.F39V
3324	*DHX37*	57647	12	125455946	Missense	G	A	0.06	p.R365W
3324	*DISC1*	27185	1	231902929	Frame_Shift_Del	AG	A	0.353	p.R470fs
3324	*DNAH11*	8701	7	21641193	Missense	G	T	0.279	p.S1202I
3324	*DNAJC27*	51277	2	25194748	Missense	T	C	0.183	p.K9R
3324	*DST*	667	6	56324965	Missense	T	G	0.317	p.T5577P
3324	*DVL2*	1856	17	7129551	Missense	T	C	0.053	p.K615R
3324	*DZANK1*	55184	20	18446022	Splice_Site	C	A	0.214	.
3324	*EBF2*	64641	8	25718821	Splice_Site	T	A	0.286	c.1164+4A>T
3324	*EGLN3*	112399	14	34419859	Missense	A	C	0.074	p.F34V
3324	*FAM47E-STBD*	100631383	4	77192868	Missense	G	A	0.148	p.E273K
3324	*FAM65C*	140876	20	49218991	Missense	G	A	0.319	p.T422I
3324	*FAM69C*	125704	18	72114011	Missense	A	C	0.2	p.F236V

3324	*FAM83B*	222584	6	54806031	Missense	A	C	0.4	p.E754D
3324	*FAT1*	2195	4	187538942	Missense	T	G	0.061	p.Q2933P
3324	*FBXO15*	201456	18	71797887	Missense	A	C	0.25	p.I37M
3324	*FLT4*	2324	5	180048811	Missense	T	A	0.268	p.K584M
3324	*FRMPD2*	143162	10	49482597	Missense	G	A	0.328	p.T5M
3324	*FZD1*	8321	7	90895013	Missense	A	C	0.204	p.K273T
3324	*GAR1*	54433	4	110739148	Missense	G	C	0.306	p.D91H
3324	*GAREM*	64762	18	29867745	Missense	T	C	0.038	p.K272R
3324	*GDF3*	9573	12	7842851	Missense	G	A	0.329	p.P240S
3324	*GIPC3*	126326	19	3586562	Missense	G	A	0.306	p.E99K
3324	*GPR125*	166647	4	22475411	Missense	T	C	0.474	p.S105G
3324	*GPR157*	80045	1	9165685	Missense	G	A	0.071	p.R218C
3324	*GRIA4*	2893	11	105781225	Missense	A	G	0.3	p.D408G
3324	*HCRTR2*	3062	6	55113563	Missense	C	G	0.397	p.T117S
3324	*HKR1*	284459	19	37853253	Missense	A	G	0.25	p.R186G
3324	*HSD3B1*	3283	1	120056673	Missense	A	C	0.442	p.N176T
3324	*HUWE1*	10075	X	53610641	Missense	C	A	0.064	p.K1799N
3324	*IAPP*	3375	12	21531254	Missense	A	C	0.327	p.N55T
3324	*IBSP*	3381	4	88731906	Missense	T	C	0.407	p.L132P
3324	*IBSP*	3381	4	88731881	Missense	T	C	0.321	p.Y124H
3324	*IGSF3*	3321	1	117146423	Missense	C	T	0.444	p.G503S
3324	*IL17REL*	400935	22	50439514	Missense	G	T	0.5	p.H36N
3324	*IQCA1*	79781	2	237272565	Missense	G	A	0.279	p.P576L
3324	*IQGAP3*	128239	1	156531643	Missense	T	C	0.133	p.E343G
3324	*ISYNA1*	51477	19	18546734	Splice_Site	G	A	0.367	c.976-3C>T
3324	*KBTBD12*	166348	3	127641968	Missense	C	A	0.048	p.Q22K
3324	*KCNK2*	3776	1	215259839	Missense	T	A	0.333	p.W44R
3324	*KIAA0100*	9703	17	26961940	Missense	T	C	0.386	p.K889E
3324	*KIF6*	221458	6	39507787	Missense	T	C	0.367	p.K546R
3324	*KRT27*	342574	17	38933970	Missense	C	A	0.25	p.E329D
3324	*KRTAP11-1*	337880	21	32253513	Missense	A	T	0.065	p.C111S
3324	*LCA5*	167691	6	80197570	Missense	T	G	0.375	p.E415D
3324	*LILRA4*	23547	19	54848157	Missense	A	G	0.111	p.Y404H
3324	*LRP1B*	53353	2	141771191	Missense	T	C	0.259	p.T772A
3324	*MAGED1*	9500	X	51644764	Missense	C	A	0.081	p.T748N
3324	*MARVELD2*	153562	5	68716362	Splice_Site	A	C	0.375	c.1146+4A>C
3324	*MED12L*	116931	3	150840650	Missense	A	C	0.382	p.Q95H
3324	*MFAP1*	4236	15	44097413	Missense	G	T	0.413	p.T400N
3324	*MTF2*	22823	1	93592788	Splice_Site	G	A	0.319	c.922-1G>A
3324	*MUC16*	94025	19	9065656	Missense	T	C	0.276	p.S7264G
3324	*MUC16*	94025	19	9062182	Frame_Shift_Del	TG	T	0.213	p.T8421fs
3324	*MYH13*	8735	17	10243710	Missense	C	T	0.188	p.G635R
3324	*MYO18B*	84700	22	26422950	Frame_Shift_Del	CA	C	0.333	p.T2337fs
3324	*NAT2*	10	8	18258105	Missense	A	C	0.378	p.T198P
3324	*NCAPG2*	54892	7	158447333	Missense	C	A	0.133	p.Q900H
3324	*NHS*	4810	X	17744510	Missense	T	G	0.609	p.F762V
3324	*NKX2-1*	7080	14	36987192	Missense	T	G	0.14	p.N166T
3324	*NRG3*	10718	10	84711262	Missense	T	G	0.258	p.I143M
3324	*NRIP1*	8204	21	16338248	Missense	T	G	0.323	p.K756Q
3324	*OR11G2*	390439	14	20665706	Missense	T	G	0.347	p.V71G
3324	*OR2F1*	26211	7	143657892	Missense	T	A	0.036	p.F277I
3324	*OR2L13*	284521	1	248112794	Missense	G	C	0.667	p.G212A
3324	*OR9Q1*	219956	11	57886024	Missense	T	G	0.307	p.K298T
3324	*PCDHA8*	56140	5	140228918	Missense	T	A	0.667	p.S280T
3324	*PDPR*	55066	16	70176575	Missense	A	G	0.118	p.K531E
3324	*PGLYRP2*	114770	19	15582863	Missense	C	T	0.107	p.R394Q
3324	*PIH1D1*	55011	19	49950298	Missense	C	T	0.095	p.V224I
3324	*PIK3C2G*	5288	12	18716387	Missense	T	G	0.28	p.L1064R
3324	*PLA2G4E*	123745	15	42292348	Missense	T	A	0.333	p.Q269L
3324	*PTPN14*	5784	1	214558072	Missense	A	C	0.25	p.L376V
3324	*PYGL*	5836	14	51379778	Missense	A	C	0.343	p.V530G
3324	*RIMS2*	9699	8	105264025	Missense	G	A	0.074	p.G1343R
3324	*RUNX1T1*	862	8	92972522	Missense	G	A	0.413	p.P561L
3324	*RYR3*	6263	15	33855189	Missense	G	A	0.371	p.R375H
3324	*SAG*	6295	2	234237198	Missense	C	T	0.19	p.A196V
3324	*SCLT1*	132320	4	129857846	Missense	A	C	0.305	p.L598R
3324	*SHISA7*	729956	19	55944629	Missense	C	A	0.167	p.R504M
3324	*SLC5A7*	60482	2	108608661	Missense	T	G	0.412	p.L93R
3324	*SLCO5A1*	81796	8	70744812	Missense	G	A	0.067	p.L33F
3324	*SNCB*	6620	5	176048214	Splice_Site	C	A	0.182	c.372+1G>T
3324	*SOGA3*	387104	6	127836023	Missense	G	A	0.37	p.A424V
3324	*SPECC1*	92521	17	20109196	Missense	A	T	0.158	p.I612F
3324	*SPIB*	6689	19	50926264	Missense	T	C	0.07	p.L84P
3324	*SSPO*	23145	7	149509419	Missense	C	T	0.067	p.R3273W
3324	*TACC3*	10460	4	1729953	Missense	G	A	0.075	p.C275Y
3324	*TACC3*	10460	4	1729988	Missense	G	A	0.067	p.G287S
3324	*TCF7L2*	6934	10	114901072	Missense	A	C	0.082	p.T228P
3324	*TET2*	54790	4	106157290	Nonsense	C	T	0.6	p.Q731X
3324	*TMEM117*	84216	12	44782218	Missense	A	C	0.323	p.R436S
3324	*TMPRSS15*	5651	21	19642349	Missense	A	C	0.342	p.N999K

3324	*TOR1A*	1861	9	132584959	Missense	G	C	0.395	p.I115M
3324	*TRAM1L1*	133022	4	118005907	Missense	A	C	0.267	p.L215V
3324	*UNC13A*	23025	19	17729726	Missense	A	G	0.41	p.S1440P
3324	*UNC5D*	137970	8	35606112	Missense	G	A	0.246	p.V612I
3324	*XIRP2-AS1*	100874011	2	167992437	Missense	A	C	0.353	p.S143R
3324	*ZBBX*	79740	3	167023579	Missense	A	C	0.467	p.L526R
3324	*ZNF205*	7755	16	3169442	Missense	C	T	0.263	p.P261S
3324	*ZNF281*	23528	1	200378064	Missense	C	A	0.33	p.S221I
151	*ABCA1*	19	9	107591295	Missense	T	C	0.258	p.I673V
151	*ABCA9-AS1*	104355297	17	67003947	Missense	G	A	0.129	p.R1130C
151	*ACOX1*	51	17	73949540	Missense	G	C	0.2	p.I312M
151	*ADRA1D*	146	20	4228640	Missense	C	T	0.143	p.R322H
151	*AGL*	178	1	100316589	Splice_Site	A	G	0.063	.
151	*AKAP13*	11214	15	86124483	Missense	C	G	0.043	p.P1062A
151	*ANKRD26*	22852	10	27317840	Missense	C	T	0.06	p.V1305I
151	*ANKRD52*	283373	12	56639301	Missense	C	G	0.286	p.R755P
151	*ARFGEF1*	10565	8	68200276	Missense	T	C	0.061	p.Y314C
151	*ARPC1A*	10552	7	98963542	Missense	G	T	0.13	p.R368L
151	*ATXN7L1*	222255	7	105254506	Missense	G	T	0.061	p.L759M
151	*C12orf50*	160419	12	88391873	Missense	G	C	0.139	p.I76M
151	*CACNA1B*	774	9	141016207	Missense	A	T	0.179	p.T2197S
151	*CBR4*	84869	4	169931175	Missense	C	T	0.167	p.M22I
151	*CCKAR*	886	4	26491040	Missense	G	A	0.102	p.T60M
151	*CD200R1*	131450	3	112642568	Missense	C	G	0.092	p.E335Q
151	*CDH20*	28316	18	59167652	Missense	A	G	0.212	p.D193G
151	*CHD5*	26038	1	6169924	Missense	C	T	0.06	p.E1837K
151	*CHPT1*	56994	12	102108345	Missense	T	C	0.054	p.F162S
151	*CLCN6*	1185	1	11896069	Nonsense	C	A	0.115	p.Y613X
151	*COL6A3*	1293	2	238303686	Missense	C	T	0.157	p.G85R
151	*COL6A5*	256076	3	130103709	Missense	G	A	0.06	p.E455K
151	*CSMD3*	114788	8	113933907	Missense	G	A	0.159	p.R488W
151	*CTAGE1*	64693	18	19996606	Missense	A	G	0.15	p.M390T
151	*CYB5RL*	606495	1	54661150	Missense	C	T	0.12	p.R47K
151	*DCHS2*	54798	4	155241572	Missense	G	A	0.047	p.S1205L
151	*DENND4A*	10260	15	65983315	Missense	T	G	0.261	p.D1205A
151	*DHX57*	90957	2	39053704	Missense	C	T	0.069	p.D923N
151	*DLK1*	8788	14	101200896	Missense	T	G	0.149	p.V272G
151	*DMD*	1756	X	32503194	Missense	T	C	0.214	p.D759G
151	*DMD*	1756	X	32380996	Missense	C	T	0.103	p.R1622H
151	*DMXL1*	1657	5	118510977	Missense	G	A	0.146	p.A2235T
151	*DRD3*	1814	3	113890815	Missense	C	T	0.078	p.G9S
151	*DSCAML1*	57453	11	117403235	Missense	G	T	0.06	p.H232N
151	*DSCAS*	101927698	18	28714604	Missense	T	A	0.103	p.N603Y
151	*EGFLAM*	133584	5	38412674	Missense	C	T	0.094	p.T473M
151	*EIF3A*	8661	10	120797796	Missense	C	G	0.136	p.D1228H
151	*EP300*	2033	22	41546084	Nonsense	C	G	0.108	p.S900X
151	*FAM186A*	121006	12	50727706	Missense	G	C	0.086	p.H2228Q
151	*FAM214A*	56204	15	52901283	Missense	T	C	0.048	p.T610A
151	*FAM217A*	222826	6	4069166	Missense	C	T	0.045	p.V431I
151	*FAM221B*	392307	9	35826038	Missense	T	C	0.06	p.K41E
151	*FAM221B*	392307	9	35826058	Missense	T	C	0.06	p.H34R
151	*FAT1*	2195	4	187542633	Missense	C	G	0.168	p.D1703H
151	*FBN2*	2201	5	127728992	Missense	G	T	0.161	p.P434H
151	*FSCB*	84075	14	44974966	Missense	G	A	0.133	p.P409S
151	*FSCB*	84075	14	44975052	Missense	A	G	0.062	p.L380P
151	*FSTL5*	56884	4	162459386	Missense	G	A	0.122	p.A415V
151	*FUT1*	2523	19	49253779	Missense	C	T	0.082	p.E254K
151	*GAL3ST3*	89792	11	65810064	Missense	C	T	0.286	p.G404S
151	*GNL3*	26354	3	52727257	Missense	G	A	0.051	p.V355M
151	*GRIN2B*	2904	12	13761578	Missense	C	G	0.078	p.E657Q
151	*HEG1*	57493	3	124731689	Missense	T	A	0.068	p.T912S
151	*HNRNPA3*	220988	2	178081295	Missense	G	C	0.375	p.E206D
151	*IGSF22*	283284	11	18738329	Missense	C	T	0.096	p.E398K
151	*ITIH4*	3700	3	52852272	Splice_Site	C	T	0.182	c.2179+1G>A
151	*KCTD3*	51133	1	215793652	Missense	G	A	0.122	p.V714I
151	*KRT76*	51350	12	53165980	Missense	G	T	0.217	p.N345K
151	*LACC1*	144811	13	44457925	Missense	A	G	0.107	p.I254V
151	*LAMA2*	3908	6	129714390	Missense	C	A	0.06	p.T1812N
151	*.OC10012908:*	100129083	19	51920635	Missense	C	G	0.08	p.C41S
151	*LRP2*	4036	2	170003432	Missense	T	G	0.054	p.I4210L
151	*MEIG1*	644890	10	15008493	Missense	A	C	0.103	p.K9T
151	*MKI67*	4288	10	129911856	Missense	C	G	0.071	p.E497D
151	*MKI67*	4288	10	129903016	Missense	T	C	0.052	p.N2363S
151	*MKI67*	4288	10	129905429	Missense	C	T	0.043	p.V1559M
151	*MKKS*	8195	20	10386013	Missense	C	A	0.061	p.G532V
151	*MS4A14*	84689	11	60184277	Missense	A	C	0.06	p.Q645H
151	*MUC19*	283463	12	40837264	Splice_Site	T	C	0.051	c.4212+2T>C
151	*MUC6*	4588	11	1017789	Missense	A	C	0.13	p.M1671R
151	*NLRP9*	338321	19	56249615	Missense	G	C	0.086	p.I42M
151	*OR10A7*	121364	12	55615094	Missense	G	A	0.04	p.G96S

151	*OR10H3*	26532	19	15852242	Missense	C	A	0.06	p.L14I
151	*OR10S1*	219873	11	123847596	Missense	G	T	0.068	p.P268Q
151	*OR2A25*	392138	7	143771937	Missense	G	C	0.073	p.A209P
151	*OR2F2*	135948	7	143632693	Missense	A	T	0.186	p.H123L
151	*OR4D9*	390199	11	59283152	Missense	A	G	0.15	p.Y256C
151	*OR52N5*	390075	11	5799468	Missense	C	T	0.091	p.V133I
151	*OR6K3*	391114	1	158687896	Missense	C	T	0.077	p.G4R
151	*PALM2*	114299	9	112705460	Missense	G	A	0.173	p.G331S
151	*PCDH18*	54510	4	138450874	Missense	A	C	0.254	p.M790R
151	*PCDHA5*	56143	5	140249527	Missense	C	T	0.185	p.S280F
151	*PDK1*	5163	2	173429230	Splice_Site	G	A	0.081	c.411-1G>A
151	*PDZRN4*	29951	12	41966304	Missense	G	A	0.084	p.E575K
151	*PEMT*	10400	17	17409601	Missense	G	A	0.097	p.T161M
151	*PI4KA*	5297	22	21161673	Missense	C	T	0.156	p.R382H
151	*PITRM1*	10531	10	3202065	Missense	T	C	0.062	p.I328V
151	*PKD1L2*	114780	16	81253745	Missense	C	G	0.068	p.K77N
151	*PKD1L2*	114780	16	81253759	Missense	A	G	0.067	p.W73R
151	*PLK4*	10733	4	128814964	Missense	G	T	0.042	p.E830D
151	*PLXNA2*	5362	1	208225726	Missense	C	T	0.185	p.G980E
151	*POLE2*	5427	14	50122413	Missense	C	G	0.227	p.E302Q
151	*POU6F2*	11281	7	39500263	Missense	A	G	0.295	p.Q507R
151	*PTPN1*	5770	20	49195089	Missense	G	A	0.159	p.G209R
151	*ROBO2*	6092	3	77684138	Missense	G	A	0.14	p.G1358E
151	*RP11-87M18.2*		X	36403036	Missense	A	G	0.071	p.H606R
151	*RRBP1*	6238	20	17600357	Missense	G	A	0.082	p.S766L
151	*SASS6*	163786	1	100575933	Missense	G	A	0.057	p.A259V
151	*SCGB1D1*	10648	11	61957776	Missense	T	A	0.103	p.L7H
151	*SHROOM3*	57619	4	77675505	Missense	C	T	0.059	p.P1290L
151	*SLC25A43*	203427	X	118587003	Missense	C	T	0.13	p.P334L
151	*SLFN5*	162394	17	33592621	Missense	C	T	0.047	p.A797V
151	*SMAP2*	64744	1	40882598	Missense	C	G	0.22	p.P332A
151	*SPP1*	6696	4	88898941	Missense	C	T	0.067	p.A24V
151	*SVEP1*	79987	9	113168795	Missense	G	A	0.065	p.R3029W
151	*TLN2*	83660	15	63017302	Missense	C	T	0.294	p.T1085M
151	*TMEM104*	54868	17	72832569	Missense	G	A	0.067	p.A412T
151	*TMEM5*	10329	12	64199051	Missense	A	T	0.25	p.M261L
151	*TMX2*	51075	11	57505845	Frame_Shift_Ins	A	AC	0.147	p.K128fs
151	*TTC21B*	79809	2	166744896	Missense	C	T	0.093	p.G1118S
151	*TTN*	7273	2	179398509	Missense	C	A	0.089	p.G34278V
151	*TTN*	7273	2	179404628	Missense	T	A	0.063	p.I32722F
151	*TUBG2*	27175	17	40818699	Missense	A	G	0.049	p.M413V
151	*UGT3A2*	167127	5	36049012	Missense	T	G	0.12	p.K274N
151	*VWDE*	221806	7	12406989	Missense	C	G	0.067	p.K964N
151	*WAPAL*	23063	10	88277457	Missense	C	T	0.051	p.V124I
151	*WDR87*	83889	19	38384356	Missense	C	T	0.245	p.V624M
151	*XIRP2*	129446	2	168115668	Nonsense	T	A	0.131	p.L904X
151	*ZIM3*	114026	19	57646570	Missense	T	C	0.091	p.I379V
151	*ZNF197*	10168	3	44683542	Missense	C	T	0.061	p.A307V
151	*ZNF264*	9422	19	57723007	Missense	G	C	0.053	p.R181T
151	*ZNF264*	9422	19	57723013	Missense	G	A	0.05	p.R183H
151	*ZNF365*	22891	10	64159333	Missense	G	T	0.097	p.A337S
151	*ZNF610*	162963	19	52869278	Missense	G	C	0.085	p.R216P
151	*ZNF624*	57547	17	16527795	Missense	C	A	0.063	p.K135N
149	*ABCA8*	10351	17	66879946	Missense	C	G	0.114	p.K1231N
149	*ACAN*	176	15	89382175	Missense	T	G	0.359	p.L118V
149	*ACSM2A*	123876	16	20494408	Missense	C	T	0.087	p.S513L
149	*ADAMTS18*	170692	16	77401545	Missense	A	G	0.058	p.Y191H
149	*AGO3*	192669	1	36474632	Missense	A	C	0.769	p.Y339S
149	*ALK*	238	2	29416481	Missense	T	C	0.044	p.K1491R
149	*ARRDC3*	57561	5	90669563	Nonsense	G	A	0.217	p.Q376X
149	*ATP13A5*	344905	3	193081122	Missense	G	T	0.062	p.S96Y
149	*ATP8B4*	79895	15	50226313	Missense	G	T	0.052	p.H452N
149	*AUTS2*	26053	7	70228020	Missense	G	T	0.067	p.A303S
149	*AXIN2*	8313	17	63554591	Missense	G	A	0.1	p.P50S
149	*BDP1*	55814	5	70806649	Missense	T	A	0.059	p.F1244I
149	*BDP1*	55814	5	70806457	Missense	G	A	0.052	p.G1180S
149	*BHMT2*	23743	5	78379627	Missense	G	A	0.111	p.G320S
149	*BIRC3*	330	11	102196019	Missense	A	G	0.038	p.K260R
149	*C10orf71*	118461	10	50531972	Missense	A	C	0.064	p.D461A
149	*C17orf102*	400591	17	32904586	Missense	C	T	0.057	p.R155K
149	*C17orf77*	146723	17	72588326	Missense	A	C	0.062	p.R47S
149	*C3orf20*	84077	3	14755617	Missense	C	G	0.091	p.L422V
149	*C3orf20*	84077	3	14755572	Missense	A	G	0.089	p.I407V
149	*C7orf72*	100130988	7	50180960	Missense	A	C	0.237	p.K354T
149	*CAND2*	23066	3	12858028	Missense	T	C	0.083	p.S533P
149	*CASD1*	64921	7	94174854	Splice_Site	T	C	0.045	c.1477-3T>C
149	*CCDC90B*	60492	11	82996986	Missense	A	C	0.069	p.F10L
149	*CDCA2*	157313	8	25364834	Missense	A	T	0.063	p.R884S
149	*CDRT4*	284040	17	15341183	Missense	A	C	0.044	p.H122Q
149	*CENPBD1*	92806	16	90037828	Missense	G	A	0.069	p.T168I

149	*CEP295*	85459	11	93431931	Missense	C	A	0.07	p.Q1285K
149	*CFAP46*	54777	10	134748331	Missense	C	T	0.079	p.S264N
149	*CHGB*	1114	20	5903388	Missense	A	C	0.057	p.N200H
149	*CISD3*	284106	17	36889559	Missense	C	T	0.065	p.R79C
149	*CLTCL1*	8218	22	19184095	Missense	T	C	0.074	p.M1316V
149	*CNTN5*	53942	11	100226883	Missense	T	A	0.038	p.S1079T
149	*COA6*	388753	1	234509259	Missense	G	C	0.083	p.S16T
149	*COIL*	8161	17	55038196	Missense	T	C	0.263	p.E62G
149	*COL24A1*	255631	1	86488232	Missense	G	A	0.047	p.P731S
149	*COL28A1*	340267	7	7495735	Missense	T	C	0.061	p.I437M
149	*COL6A6*	131873	3	130361856	Missense	G	A	0.077	p.R1739Q
149	*COL9A1*	1297	6	71004064	Missense	C	A	0.141	p.A168S
149	*CST5*	1473	20	23860178	Missense	A	G	0.059	p.C46R
149	*CTC1*	80169	17	8132763	Missense	T	C	0.107	p.I1005V
149	*CTNNA3*	29119	10	67680352	Missense	G	C	0.125	p.I808M
149	*CXorf67*	340602	X	51150032	Missense	C	T	0.105	p.S55L
149	*CYLC2*	1539	9	105767901	Missense	G	A	0.2	p.D330N
149	*DCHS2*	54798	4	155157430	Missense	T	G	0.047	p.N2337H
149	*DNAH17*	8632	17	76528790	Missense	A	G	0.122	p.I963T
149	*DNAH17*	8632	17	76503593	Missense	T	C	0.073	p.T1511A
149	*DSEL*	92126	18	65181638	Missense	C	T	0.041	p.G80R
149	*DUSP27*	92235	1	167095881	Missense	G	A	0.085	p.A505T
149	*DUSP27*	92235	1	167097739	Missense	C	A	0.047	p.T1124N
149	*DYSF*	8291	2	71908233	Missense	G	C	0.1	p.D2056H
149	*EDDM3B*	64184	14	21238322	Missense	C	G	0.082	p.L5V
149	*EEF1D*	1936	8	144671956	Missense	G	A	0.081	p.A99V
149	*ELP2*	55250	18	33725931	Missense	G	A	0.071	p.V370M
149	*EPRS*	2058	1	220156704	Missense	T	C	0.064	p.I1043V
149	*ERMARD*	55780	6	170176467	Missense	A	G	0.071	p.K520E
149	*ETAA1*	54465	2	67631831	Missense	G	A	0.034	p.E673K
149	*EYS*	346007	6	65622463	Missense	A	G	0.049	p.L852P
149	*FAM200B*	285550	4	15689928	Missense	G	A	0.05	p.S443N
149	*FAM221A*	340277	7	23737891	Missense	A	G	0.079	p.S240G
149	*FAM83H*	286077	8	144809376	In_Frame_Del	GCGC	G	0.667	p.751_752del
149	*FBXO7*	25793	22	32875190	Missense	G	A	0.095	p.M115I
149	*FCRL3*	115352	1	157667057	Nonsense	C	T	0.093	p.W239X
149	*FDXR*	2232	17	72860653	Missense	G	A	0.103	p.R282W
149	*FOXN1*	8456	17	26851602	Missense	C	T	0.089	p.R69C
149	*FSIP2*	401024	2	186672866	Missense	G	T	0.085	p.S6367I
149	*FYB*	2533	5	39119723	Missense	C	A	0.05	p.V718F
149	*GDPD4*	220032	11	76990447	Splice_Site	GA	G	0.25	c.54-4T>-
149	*GJA9-MYCBP*	100527950	1	39340282	Missense	C	T	0.061	p.V497I
149	*GNAQ*	2776	9	80537112	Missense	T	A	0.17	p.T96S
149	*GNAQ*	2776	9	80537095	Nonsense	G	T	0.13	p.Y101X
149	*GOLGB1*	2804	3	121414061	Missense	C	T	0.043	p.G1765D
149	*GOLGB1*	2804	3	121415720	Missense	T	C	0.042	p.Y1212C
149	*GPC4*	2239	X	132437337	Missense	G	A	0.1	p.A442V
149	*GPR124*	25960	8	37692878	Missense	G	A	0.136	p.G599R
149	*GPR15*	2838	3	98250986	Missense	C	T	0.045	p.P37S
149	*HDAC6*	10013	X	48661113	Missense	G	A	0.071	p.G5D
149	*HEATR1*	55127	1	236718620	Missense	T	C	0.086	p.N1967D
149	*HEATR1*	55127	1	236749649	Missense	T	C	0.065	p.M607V
149	*HECTD4*	283450	12	112688167	Missense	A	C	0.692	p.V1110G
149	*HIF3A*	64344	19	46823702	Missense	A	G	0.12	p.Q343R
149	*HKDC1*	80201	10	71026510	Missense	C	A	0.053	p.N917K
149	*HSPA6*	3310	1	161495040	Missense	C	T	0.07	p.L198F
149	*HSPA8*	3312	11	122929401	Frame_Shift_Del	ATT	A	0.241	p.N487fs
149	*HTR4*	3360	5	147889136	Missense	C	G	0.19	p.R320T
149	*IDO2*	169355	8	39872935	Nonsense	T	A	0.071	p.Y359X
149	*IFNGR2*	3460	21	34787312	Missense	A	G	0.114	p.Q64R
149	*IFT74*	80173	9	26978170	Missense	A	G	0.079	p.I55M
149	*IGFN1*	91156	1	201170855	Missense	C	G	0.318	p.N214K
149	*IL18R1*	8809	2	102992516	Missense	A	G	0.1	p.I206M
149	*IL4I1*	259307	19	50412217	Missense	C	G	0.156	p.S283T
149	*ISG15*	9636	1	949608	Missense	G	A	0.062	p.S83N
149	*KCNJ11*	3767	11	17409572	Missense	T	C	0.125	p.K23E
149	*KCNJ15*	3772	21	39671476	Missense	G	A	0.045	p.G98D
149	*KIAA1210*	57481	X	118222360	Missense	T	A	0.151	p.I945F
149	*KIAA1407*	57577	3	113720517	Missense	T	A	0.043	p.E696D
149	*KIAA1522*	57648	1	33231380	Missense	T	G	0.206	p.F4V
149	*KLHL15*	80311	X	24007151	Splice_Site	A	G	0.13	c.706-4T>C
149	*KLK1*	3816	19	51323232	Missense	T	C	0.073	p.K186E
149	*KLK4*	9622	19	51412668	Missense	A	C	0.16	p.S22A
149	*KLRF2*	100431172	12	10041364	Missense	G	A	0.046	p.V68I
149	*KMT2D*	8085	12	49431915	Nonsense	G	T	0.077	p.S3075X
149	*KRT86*	3892	12	52699033	Missense	G	A	0.179	p.V249I
149	*KRTAP16-1*	100505753	17	39464736	Missense	C	G	0.067	p.S257T
149	*KRTAP29-1*	100533177	17	39458893	Missense	G	A	0.043	p.R71C
149	*LAMB1*	3912	7	107603453	Missense	C	G	0.114	p.G585A
149	*LARP7*	51574	4	113565835	Missense	G	A	0.056	p.E11K

149	*LDLRAD2*	401944	1	22141206	Missense	A	C	0.2	p.N134T
149	*LDLRAP1*	26119	1	25889632	Missense	T	C	0.109	p.S202P
149	*LGR6*	59352	1	202287250	Missense	G	A	0.063	p.A555T
149	*LIPT2*	387787	11	74203230	Missense	A	G	0.153	p.F216L
149	*OC10037922*	100379224	19	44610665	Missense	A	G	0.043	p.M118V
149	*OC10037922*	100379224	19	44612231	Missense	A	G	0.041	p.K640E
149	*OC10192714*	101927142	2	101591304	Missense	A	G	0.081	p.T394A
149	*OC10192865*	101928651	5	60982841	Missense	G	A	0.055	p.A57T
149	*OC10192929*	101929294	8	24339679	Missense	G	A	0.073	p.V244M
149	*LOC442028*	442028	2	95537572	Missense	C	T	0.211	p.T83M
149	*LOXHD1*	125336	18	44063598	Missense	G	A	0.089	p.A2036V
149	*LOXL1-AS1*	100287616	15	74219582	Missense	G	A	0.133	p.G153D
149	*LRIT3*	345193	4	110773067	Missense	G	A	0.088	p.S175N
149	*LRRC34*	151827	3	169514585	Missense	A	T	0.056	p.L286I
149	*LRRC41*	10489	1	46746164	Missense	C	T	0.143	p.V609I
149	*LRRC61*	65999	7	150034378	Missense	A	G	0.082	p.N143S
149	*MAGEB16*	139604	X	35820795	Missense	A	G	0.111	p.H161R
149	*MAGEB6*	158809	X	26213093	Missense	C	T	0.058	p.A377V
149	*MAGEC1*	9947	X	140996239	Nonsense	G	T	0.18	p.E1017X
149	*MAGI1*	9223	3	65342217	Missense	G	A	0.239	p.R1409C
149	*MALRD1*	340895	10	19676561	Missense	G	A	0.082	p.E1516K
149	*MALRD1*	340895	10	19676553	Missense	A	G	0.069	p.D1513G
149	*MAPT*	4137	17	44068860	Missense	G	A	0.185	p.R472Q
149	*MARVELD2*	153562	5	68715310	Missense	C	T	0.046	p.T33I
149	*MAVS*	57506	20	3838441	Missense	C	G	0.111	p.Q93E
149	*MC1R*	4157	16	89986154	Missense	G	A	0.15	p.R163Q
149	*MC5R*	4161	18	13826391	Missense	C	G	0.038	p.F209L
149	*MCAT*	27349	22	43529314	Missense	G	C	0.088	p.A303G
149	*MLXIP*	22877	12	122617989	Missense	A	G	0.08	p.E396G
149	*MOV10L1*	54456	22	50547158	Missense	A	C	0.061	p.K210Q
149	*MRPL30*	51263	2	99812070	Missense	G	A	0.067	p.A130T
149	*MTA2*	9219	11	62362781	Nonsense	G	A	0.092	p.R480X
149	*MTMR11*	10903	1	149906413	Missense	T	C	0.047	p.M159V
149	*MUC16*	94025	19	9072313	Missense	A	G	0.048	p.S5045P
149	*MYADML2*	255275	17	79899272	Missense	A	G	0.286	p.S116P
149	*MYO3A*	53904	10	26463043	Missense	A	T	0.043	p.T1284S
149	*MYO9B*	4650	19	17213320	Missense	G	A	0.16	p.A265T
149	*NADK*	65220	1	1686040	Missense	G	T	0.073	p.N407K
149	*NBPF10*	100132406	1	145561334	Missense	C	T	0.06	p.A341V
149	*NIPBL*	25836	5	36985704	Nonsense	C	T	0.104	p.R808X
149	*NKAPL*	222698	6	28227436	Missense	A	G	0.055	p.Y96C
149	*NKAPL*	222698	6	28227604	Missense	C	A	0.041	p.T152N
149	*NLRP4*	147945	19	56369189	Missense	G	A	0.066	p.A144T
149	*NPEPPS*	9520	17	45664593	Splice_Site	C	T	0.143	c.981-3C>T
149	*NT5C1B*	93034	2	18765974	Missense	G	A	0.095	p.R237C
149	*NUP214*	8021	9	134020092	Missense	C	T	0.045	p.P574S
149	*NXPE4*	54827	11	114441943	Missense	G	A	0.064	p.A167V
149	*OBSCN*	84033	1	228548197	Missense	G	A	0.174	p.R6535H
149	*OR13G1*	441933	1	247835674	Missense	G	A	0.049	p.R224C
149	*OR14J1*	442191	6	29274486	Missense	T	C	0.043	p.M7T
149	*OR1J2*	26740	9	125273574	Missense	G	A	0.072	p.R165Q
149	*OR2J2*	26707	6	29142064	Missense	A	G	0.043	p.T218A
149	*OR5H14*	403273	3	97868795	Missense	A	G	0.061	p.Y189C
149	*OR5H6*	79295	3	97983942	Missense	A	G	0.091	p.T272A
149	*OR5R1*	219479	11	56185159	Missense	A	G	0.045	p.F184L
149	*OR8D4*	338662	11	123777736	Missense	T	C	0.05	p.F200L
149	*OTOP3*	347741	17	72937605	Missense	G	A	0.158	p.R64Q
149	*PANK4*	55229	1	2444414	Missense	G	A	0.093	p.A547V
149	*PCDHA1*	56147	5	140187620	Missense	A	G	0.248	p.N283S
149	*PCLO*	27445	7	82764539	Missense	G	C	0.131	p.P776R
149	*PCLO*	27445	7	82582846	Missense	C	T	0.044	p.V2475I
149	*PCLO*	27445	7	82544616	Missense	C	T	0.04	p.G4229D
149	*PCM1*	5108	8	17827260	Splice_Site	A	AC	0.152	c.3584+3->C
149	*PCM1*	5108	8	17796382	Missense	A	G	0.037	p.N159S
149	*PER2*	8864	2	239155053	Missense	C	T	0.095	p.G1244E
149	*PFAS*	5198	17	8159232	Splice_Site	A	G	0.077	c.680+4A>G
149	*PGLYRP2*	114770	19	15587185	Missense	C	T	0.06	p.R99Q
149	*PGM3*	5238	6	83880167	Missense	C	T	0.049	p.D494N
149	*PIGR*	5284	1	207112462	Splice_Site	A	T	0.185	c.388+2T>A
149	*PKP3*	11187	11	397652	Missense	C	G	0.167	p.A353G
149	*PLA2G7*	7941	6	46679250	Missense	G	T	0.049	p.H216N
149	*PLEKHO2*	80301	15	65157482	Missense	C	T	0.07	p.P290S
149	*PLIN5*	440503	19	4529204	Missense	C	T	0.073	p.R134Q
149	*PLXND1*	23129	3	129279570	Splice_Site	T	C	0.083	c.4994-4A>G
149	*POLR1E*	64425	9	37501844	Splice_Site	G	A	0.061	c.1100+3G>A
149	*PPAN-P2RY11*	692312	19	10224548	Missense	G	A	0.095	p.A507T
149	*PPP1R3A*	5506	7	113519940	Nonsense	G	A	0.205	p.Q403X
149	*PPP4R1*	9989	18	9562042	Missense	C	T	0.043	p.S576N
149	*PRB2*	653247	12	11546309	Missense	C	G	0.2	p.A235P
149	*PRDM10*	56980	11	129794950	Missense	T	C	0.048	p.T573A

149	*PRR25*	388199	16	855717	Missense	C	G	0.118	p.T92S
149	*PRR30*	339779	2	27360534	Missense	G	A	0.071	p.R222C
149	*PRSS48*	345062	4	152212486	Missense	C	T	0.055	p.R290C
149	*PTPRG*	5793	3	62189201	Missense	G	A	0.277	p.E578K
149	*PURG*	29942	8	30889848	Missense	T	C	0.218	p.K151E
149	*RAB3GAP1*	22930	2	135893372	Missense	A	G	0.046	p.N598S
149	*RARS2*	57038	6	88239266	Missense	T	C	0.079	p.K291R
149	*RDH8*	50700	19	10131999	Missense	T	C	0.219	p.M222T
149	*RDM1*	201299	17	34257637	Missense	T	C	0.068	p.H32R
149	*REPIN1*	29803	7	150068371	Missense	T	C	0.065	p.L14P
149	*REST*	5978	4	57797414	Missense	C	T	0.057	p.P797L
149	*RHBDF2*	79651	17	74475975	Missense	C	A	0.077	p.A67S
149	*RIF1*	55183	2	152331418	Missense	C	G	0.066	p.L2418V
149	*RIF1*	55183	2	152311570	Missense	G	A	0.049	p.G836S
149	*RIF1*	55183	2	152320118	Missense	G	A	0.047	p.V1362M
149	*RMI1*	80010	9	86617265	Missense	A	G	0.081	p.N455S
149	*RND3*	390	2	151326539	Missense	A	C	0.203	p.L233V
149	*RTEL1*	51750	20	62326110	Missense	A	C	0.125	p.Q1066H
149	*RTL1*	388015	14	101348584	Missense	C	G	0.073	p.E848Q
149	*S100Z*	170591	5	76171252	Missense	A	C	0.091	p.E23A
149	*SASH1*	23328	6	148865257	Missense	A	G	0.062	p.Q884R
149	*SCML1*	6322	X	17771387	Missense	G	A	0.1	p.G289R
149	*SERPINB12*	89777	18	61233907	Missense	C	T	0.05	p.S294L
149	*SLC16A1*	6566	1	113456546	Missense	A	T	0.034	p.D490E
149	*SLC25A26*	115286	3	66287056	Missense	G	A	0.06	p.S41N
149	*SLC25A37*	51312	8	23423669	Missense	A	G	0.098	p.I87V
149	*SLITRK3*	22865	3	164906186	Missense	C	A	0.13	p.L811F
149	*SORCS3*	22986	10	106974213	Missense	G	T	0.047	p.V797L
149	*SPANXC*	64663	X	140785696	Missense	T	C	0.222	p.K74E
149	*ST20-MTHFS*	100528021	15	80191343	Missense	G	A	0.05	p.P57L
149	*STON2*	85439	14	81744736	Missense	A	G	0.052	p.S307P
149	*SWT1*	54823	1	185171869	Missense	A	G	0.067	p.H536R
149	*TECPR1*	25851	7	97852399	Missense	G	A	0.133	p.P944L
149	*TEK*	7010	9	27220147	Splice_Site	A	C	0.172	c.2756+4A>C
149	*TEKT4*	150483	2	95537622	Missense	A	G	0.25	p.S100G
149	*TENM4*	26011	11	78380543	Missense	G	A	0.267	p.R2283W
149	*TFPI2*	7980	7	93519584	Missense	G	A	0.239	p.R46W
149	*TGFBR2*	7048	3	30713126	Splice_Site	T	A	0.037	c.455-4T>A
149	*THBS4*	7060	5	79375724	Splice_Site	G	C	0.064	c.2512-4G>C
149	*TIGD2*	166815	4	90035549	Missense	A	G	0.043	p.H475R
149	*TMEM132C*	92293	12	128899673	Missense	A	G	0.051	p.H161R
149	*TMEM135*	65084	11	87013438	Missense	G	A	0.07	p.G218R
149	*TMEM184A*	202915	7	1595068	Missense	G	A	0.097	p.A18V
149	*TMPRSS9*	360200	19	2389872	Missense	G	C	0.16	p.S30T
149	*TOP2B*	7155	3	25646268	Missense	G	A	0.044	p.T1486M
149	*TOPBP1*	11073	3	133368362	Missense	T	G	0.053	p.K457Q
149	*TRIM15*	89870	6	30140065	Missense	G	A	0.064	p.G446D
149	*TRIM31*	11074	6	30071330	Missense	C	T	0.052	p.E421K
149	*TRIML2*	205860	4	189013026	Missense	C	T	0.062	p.R222K
149	*TRIOBP*	11078	22	38130656	Missense	G	C	0.182	p.G1438A
149	*TRPM6*	140803	9	77502160	Missense	G	A	0.158	p.T2I
149	*TSPEAR*	54084	21	46117838	Missense	C	G	0.125	p.S241C
149	*TTN*	7273	2	179458591	Missense	C	T	0.07	p.R19479H
149	*TXNDC2*	84203	18	9887546	Missense	G	A	0.103	p.G290D
149	*UBE2J1*	51465	6	90039670	Missense	G	C	0.058	p.L229V
149	*UBP1*	7342	3	33458266	Missense	T	C	0.049	p.N109S
149	*UBQLNL*	143630	11	5536852	Missense	A	G	0.048	p.Y274H
149	*ULK4*	54986	3	41756986	Missense	A	T	0.043	p.L844M
149	*UNC79*	57578	14	94120117	Missense	T	G	0.407	p.V1900G
149	*USP8*	9101	15	50784955	Missense	C	A	0.122	p.N764K
149	*USP8*	9101	15	50784950	Missense	C	T	0.1	p.R763W
149	*USP8*	9101	15	50785055	Missense	G	C	0.043	p.A798P
149	*UVSSA*	57654	4	1377556	Missense	T	C	0.073	p.W622R
149	*VARS2*	57176	6	30888161	Missense	T	C	0.075	p.W449R
149	*VWA8*	23078	13	42442546	Missense	A	G	0.085	p.M383T
149	*WAPAL*	23063	10	88277457	Missense	C	T	0.054	p.V124I
149	*XYLT1*	64131	16	17202872	Nonsense	C	A	0.229	p.E854X
149	*YIF1B*	90522	19	38800176	Missense	G	A	0.138	p.P53S
149	*ZBTB22*	9278	6	33284348	Missense	G	A	0.173	p.R116C
149	*ZDHHC23*	254887	3	113673125	Missense	A	G	0.06	p.K247R
149	*ZFC3H1*	196441	12	72020212	Missense	G	C	0.159	p.S1382C
149	*ZNF264*	9422	19	57723013	Missense	G	A	0.044	p.R183H
149	*ZNF397*	84307	18	32834186	Missense	T	G	0.04	p.Q238P
149	*ZNF529*	57711	19	37039069	Missense	A	C	0.06	p.L131V
149	*ZNF680*	340252	7	63981559	Missense	T	C	0.07	p.N525D
149	*ZNF764*	92595	16	30567162	Missense	C	T	0.125	p.V194I
149	*ZNF880*	400713	19	52887437	Missense	A	C	0.078	p.N202H
149	*ZNF880*	400713	19	52887427	Missense	A	C	0.063	p.R198S
9638	*ANKLE1*	126549	19	17397501	Missense	T	G	0.111	p.L645V
9638	*ATP13A5*	344905	3	193052783	Missense	C	A	0.091	p.G350V

9638	*BASP1*	10409	5	17275764	Missense	G	A	0.277	p.E147K
9638	*BRF2*	55290	8	37702708	Missense	G	A	0.151	p.P187L
9638	*BYSL*	705	6	41899280	Missense	G	A	0.152	p.G284E
9638	*CPSF1*	29894	8	145624380	Missense	G	A	0.219	p.H506Y
9638	*DCTN5*	84516	16	23672555	Missense	G	A	0.254	p.A101T
9638	*DDX3X*	1654	X	41205613	Missense	G	A	0.719	p.A483T
9638	*DENND1A*	57706	9	126144544	Missense	C	T	0.21	p.D733N
9638	*DNMBP*	23268	10	101645511	Missense	G	T	0.185	p.A1244D
9638	*EGFL6*	25975	X	13618120	Missense	C	T	0.1	p.R103W
9638	*ENAM*	10117	4	71510366	Missense	A	T	0.068	p.S1075C
9638	*ESCO1*	114799	18	19154143	Missense	G	A	0.068	p.T221M
9638	*EXOC2*	55770	6	629884	Missense	G	C	0.086	p.P125A
9638	*FAM173A*	65990	16	771835	Missense	C	A	0.046	p.A101D
9638	*FAM184B*	27146	4	17710691	Missense	C	T	0.225	p.V240M
9638	*FZD1*	8321	7	90895963	Missense	G	A	0.271	p.A590T
9638	*GATA4*	2626	8	11565846	Missense	G	A	0.205	p.A9T
9638	*GBF1*	8729	10	104136145	Nonsense	C	T	0.203	p.R1334X
9638	*GEM*	2669	8	95262720	Nonsense	G	A	0.203	p.R237X
9638	*GPC6*	10082	13	94482467	Missense	C	A	0.138	p.T127N
9638	*HPSE2*	60495	10	100219374	Missense	T	A	0.043	p.Y579F
9638	*ITGA8*	8516	10	15590522	Nonsense	G	A	0.176	p.R938X
9638	*KIAA0513*	9764	16	85112529	Splice_Site	A	G	0.189	c.824-2A>G
9638	*KIR2DL1*	3802	19	55286665	Missense	T	A	0.159	p.L140Q
9638	*KLHL38*	340359	8	124665039	Missense	C	A	0.143	p.R43L
9638	*KRTAP4-11*	653240	17	39274291	Missense	T	C	0.333	p.M93V
9638	*LINGO2*	158038	9	27949105	Missense	T	C	0.068	p.N522S
9638	*MAGEB16*	139604	X	35820696	Missense	G	A	0.1	p.C128Y
9638	*MUC17*	140453	7	100681211	Missense	G	C	0.077	p.V2172L
9638	*MUC4*	4585	3	195505664	Missense	G	A	0.062	p.P4263S
9638	*MYHAS*	100128560	17	10346774	Missense	C	T	0.181	p.R1913Q
9638	*MYO6*	4646	6	76566846	Missense	C	T	0.154	p.A419V
9638	*NKX6-1*	4825	4	85414591	Missense	T	C	0.198	p.N319D
9638	*NOTCH3*	4854	19	15289953	Missense	G	A	0.158	p.R1201C
9638	*OR51G1*	79324	11	4944647	Missense	T	G	0.4	p.K308T
9638	*OR52E8*	390079	11	5877979	Nonstop	T	A	0.07	p.X318Y
9638	*OR6N1*	128372	1	158735595	Missense	C	T	0.08	p.R293H
9638	*ORC4*	5000	2	148716428	Missense	T	C	0.286	p.N78S
9638	*PCK2*	5106	14	24572931	Missense	C	T	0.092	p.R561W
9638	*PHC1*	1911	12	9085218	Nonsense	C	T	0.167	p.Q389X
9638	*PKD1L1*	168507	7	47867021	Missense	G	C	0.199	p.P2261A
9638	*PLD1*	5337	3	171395488	Splice_Site	T	G	0.375	c.1868-4A>C
9638	*PUS7*	54517	7	105108822	Missense	C	A	0.1	p.G496V
9638	*RYR3*	6263	15	33954413	Missense	C	T	0.25	p.T1561M
9638	*SLC1A5*	6510	19	47278793	Missense	C	T	0.217	p.A534T
9638	*SLC26A4*	5172	7	107335127	Missense	T	C	0.077	p.I468T
9638	*SLC27A5*	10998	19	59022291	Missense	C	T	0.093	p.R232Q
9638	*SLC28A1*	9154	15	85467279	Missense	G	T	0.206	p.V341F
9638	*SSH2*	85464	17	27998966	Missense	G	C	0.246	p.L266V
9638	*TRIM56*	81844	7	100730966	Missense	G	A	0.212	p.D125N
9638	*TRPM6*	140803	9	77377716	Missense	G	T	0.27	p.H1291N
9638	*WDR91*	29062	7	134870946	Missense	T	C	0.22	p.D734G
9638	*ZNF33B*	7582	10	43089179	Missense	G	T	0.176	p.H407N
9638	*ZNF649-AS1*	101928571	19	52394377	Nonsense	G	A	0.196	p.R338X
9638	*ZSCAN16-AS1*	100129195	6	28097571	Missense	G	A	0.292	p.C297Y
9634	*CATSPER1*	117144	11	65789246	Missense	T	C	0.123	p.N512D
9634	*CERCAM*	51148	9	131198152	Missense	A	C	0.153	p.S586R
9634	*CFAP46*	54777	10	134672644	Missense	C	A	0.135	p.R1769I
9634	*CSMD2*	114784	1	34204872	Missense	C	T	0.108	p.S706N
9634	*CYP2J2*	1573	1	60381701	Missense	C	A	0.154	p.L94F
9634	*DNAH17*	8632	17	76422617	Missense	G	A	0.132	p.P4279L
9634	*DOLPP1*	57171	9	131849037	Missense	G	A	0.221	p.V214I
9634	*DSCAM*	1826	21	41719761	Missense	C	T	0.115	p.R349H
9634	*FGFR1*	2260	8	38287254	Missense	C	T	0.107	p.V135I
9634	*FSHR*	2492	2	49381414	Missense	G	T	0.28	p.A48D
9634	*GABRR2*	2570	6	89967528	Missense	G	A	0.14	p.A420V
9634	*GNAQ*	2776	9	80537112	Missense	T	A	0.088	p.T96S
9634	*GNAQ*	2776	9	80537095	Nonsense	G	T	0.065	p.Y101X
9634	*GRM6*	2916	5	178419057	Missense	C	A	0.191	p.A178S
9634	*IPO5*	3843	13	98662459	Nonsense	C	G	0.2	p.S697X
9634	*ITPR2*	3709	12	26839433	Missense	C	A	0.086	p.A377S
9634	*KDM2A*	22992	11	67013588	Splice_Site	G	A	0.273	c.1965+1G>A
9634	*KRTAP4-7*	100132476	17	39240819	Missense	C	G	0.143	p.L121V
9634	*LAMB2*	3913	3	49167738	Missense	C	T	0.155	p.R384H
9634	*LILRA1*	11024	19	55106288	Missense	A	C	0.045	p.I77L
9634	*LMBRD1*	55788	6	70506702	Splice_Site	T	C	0.127	c.69+3A>G
9634	*LRRC49*	54839	15	71300939	Missense	A	C	0.294	p.K454T
9634	*MAGED1*	9500	X	51638851	Missense	C	T	0.194	p.R306C
9634	*MUC21*	394263	6	30955179	Missense	G	C	0.096	p.E409D
9634	*MUC21*	394263	6	30954485	Missense	G	A	0.056	p.S178N
9634	*MUC3A*	4584	7	100550974	Missense	A	C	0.119	p.T519P

9634	*MUC5B*	727897	11	1262189	Missense	C	T	0.105	p.T1360M
9634	*MUC7*	4589	4	71347185	Missense	T	C	0.106	p.S242P
9634	*NBPF8*	728841	1	148025797	Missense	T	A	0.133	p.K352M
9634	*NRXN1*	9378	2	50149175	Missense	C	A	0.185	p.Q1517H
9634	*OGDHL*	55753	10	50960187	Missense	G	A	0.075	p.R196C
9634	*PAK2*	5062	3	196509577	Missense	C	G	0.1	p.S20R
9634	*POTEG*	404785	14	19553642	Missense	A	G	0.188	p.S76G
9634	*PRB2*	653247	12	11546749	Missense	T	C	0.103	p.K88R
9634	*RETSAT*	54884	2	85570857	Missense	G	A	0.095	p.A533V
9634	*RETSAT*	54884	2	85570849	Missense	C	T	0.068	p.G536R
9634	*RYR2*	6262	1	237819173	Missense	C	A	0.097	p.A2673E
9634	*SERPINB7*	8710	18	61471710	Missense	G	T	0.107	p.E328D
9634	*SLC20A2*	6575	8	42275345	Missense	C	T	0.122	p.M645I
9634	*SPTBN5*	51332	15	42144487	Nonsense	G	A	0.109	p.Q3493X
9634	*ST6GALNAC1*	55808	17	74622824	Missense	C	A	0.046	p.R275L
9634	*TAF1A*	9015	1	222761824	Missense	T	C	0.112	p.M28V
9634	*TBC1D26*	353149	17	15641610	Missense	A	G	0.118	p.Y99C
9634	*TRIB3*	57761	20	368883	Missense	G	A	0.128	p.E104K
9634	*WDFY4*	57705	10	50109851	Missense	G	T	0.159	p.C2510F
9634	*ZNRF3*	84133	22	29445329	Missense	T	C	0.165	p.M287T
9634	*ZPLD1*	131368	3	102196348	Missense	G	C	0.152	p.M394I
9626	*ARID1B*	57492	6	157256690	Missense	G	A	0.138	p.E673K
9626	*BRD9*	65980	5	878525	Missense	T	A	0.091	p.M406L
9626	*CACNA1E*	777	1	181731752	Missense	G	A	0.046	p.V1550M
9626	*CCM2*	83605	7	45113105	Missense	A	G	0.075	p.K284E
9626	*CCSER1*	401145	4	91229489	Missense	C	A	0.067	p.F18L
9626	*DOCK8*	81704	9	368103	Missense	A	T	0.052	p.M589L
9626	*FGD2*	221472	6	36979483	Missense	T	G	0.542	p.V127G
9626	*GNAQ*	2776	9	80537112	Missense	T	A	0.068	p.T96S
9626	*GPR112*	139378	X	135426656	Missense	C	T	0.056	p.A264V
9626	*LRRC46*	90506	17	45914313	Missense	G	A	0.069	p.V265I
9626	*MUC21*	394263	6	30955179	Missense	G	C	0.185	p.E409D
9626	*NKAPL*	222698	6	28228281	Nonsense	C	T	0.048	p.R378X
9626	*NOTUM*	147111	17	79915727	Missense	C	A	0.143	p.G217V
9626	*OR6C75*	390323	12	55759444	Missense	C	G	0.05	p.Q184E
9626	*PCDH15*	65217	10	55582472	Missense	A	G	0.037	p.S1679P
9626	*PCDHGA2*	56113	5	140718786	Missense	G	C	0.134	p.S83T
9626	*PCDHGA3*	56112	5	140730393	Missense	C	T	0.099	p.P189L
9626	*PCDHGA7*	56108	5	140866334	Missense	C	A	0.041	p.Q532K
9626	*PCMTD1*	115294	8	52733128	Missense	T	G	0.13	p.N286T
9626	*PGR*	5241	11	100998489	Missense	G	A	0.15	p.A274V
9626	*RNF2*	6045	1	185069041	Missense	G	A	0.103	p.A286T
9626	*SCCPDH*	51097	1	246887755	Missense	G	C	0.087	p.V11L
9626	*SEC31B*	25956	10	102269096	Missense	C	G	0.079	p.A126P
9626	*SLC6A5*	9152	11	20625882	Missense	A	C	0.109	p.K197N
9626	*SPDYE6*	729597	7	101988983	Missense	G	A	0.261	p.P297L
9626	*SPHKAP*	80309	2	228882809	Missense	G	T	0.066	p.P921T
9626	*TENM2*	57451	5	167303069	Missense	T	G	0.056	p.V194G
9626	*TMEM14B*	81853	6	10756728	Missense	C	T	0.098	p.R108C
9626	*UBR5*	51366	8	103297838	Missense	G	A	0.082	p.S1796L
9626	*USP8*	9101	15	50784950	Missense	C	T	0.125	p.R763W
9626	*USP8*	9101	15	50784955	Missense	C	A	0.118	p.N764K
9626	*ZNF227*	7770	19	44739059	Missense	A	G	0.113	p.D159G
9624	*A2M*	2	12	9244021	Missense	C	A	0.057	p.A749S
9624	*ABCC8*	6833	11	17418477	Missense	C	A	0.079	p.A1370S
9624	*ADAMTSL1*	92949	9	18776926	Missense	C	T	0.071	p.P900L
9624	*ANKRD22*	118932	10	90591731	Missense	A	C	0.5	p.V25G
9624	*ARHGAP25*	9938	2	69049941	Missense	T	C	0.059	p.M557T
9624	*ASH1L*	55870	1	155448155	Missense	C	A	0.198	p.M1502I
9624	*ASPM*	259266	1	197111871	Missense	C	G	0.131	p.C504S
9624	*BTC*	685	4	75695364	Frame_Shift_Del	GA	G	0.129	p.G22fs
9624	*CCDC90B*	60492	11	82984731	Nonsense	C	A	0.25	p.E185X
9624	*CDH19*	28513	18	64211304	Missense	G	T	0.182	p.P373Q
9624	*CETN1*	1068	18	580556	Missense	G	A	0.059	p.A50T
9624	*CHIT1*	1118	1	203186950	Nonsense	C	T	0.4	p.W358X
9624	*DCT*	1638	13	95121106	Nonsense	C	T	0.137	p.W163X
9624	*DEFB1*	1672	8	6728315	Missense	G	T	0.116	p.S32Y
9624	*DISP1*	84976	1	223165455	Splice_Site	G	C	0.102	c.889+3G>C
9624	*FBXO10*	26267	9	37537688	Missense	A	G	0.083	p.S280P
9624	*IGFL1*	374918	19	46733771	Missense	G	A	0.114	p.R107H
9624	*KIF20B*	9585	10	91497912	Missense	A	G	0.091	p.K1065R
9624	*KPNA7*	402569	7	98790650	Missense	T	G	0.182	p.T210P
9624	*KRTAP9-8*	83901	17	39394482	Missense	T	C	0.286	p.I60T
9624	*LDHB*	3945	12	21791326	Missense	C	G	0.129	p.S225T
9624	*MAPK1*	5594	22	22127164	Missense	C	T	0.172	p.E322K
9624	*MCM6*	4175	2	136609088	Missense	G	C	0.149	p.L601V
9624	*MERTK*	10461	2	112754933	Missense	C	T	0.158	p.A495V
9624	*MYLK*	4638	3	123471350	Missense	G	C	0.136	p.H67Q
9624	*MYO10*	4651	5	16670671	Missense	C	A	0.06	p.E1949D
9624	*NCAM2*	4685	21	22746187	Missense	T	C	0.105	p.L350P

9624	*NPY2R*	4887	4	156135107	Missense	G	T	0.092	p.A6S
9624	*NWD1*	284434	19	16870139	Missense	C	T	0.09	p.R490C
9624	*OR5P2*	120065	11	7817732	Missense	A	T	0.088	p.F253Y
9624	*PCDHB13*	56123	5	140594182	Missense	G	A	0.067	p.V163I
9624	*PCNA*	5111	20	5095961	Missense	C	G	0.12	p.A252P
9624	*PIP5KL1*	138429	9	130692077	Missense	G	A	0.078	p.R40C
9624	*PNKP*	11284	19	50370325	Missense	C	T	0.077	p.C46Y
9624	*RNU6-71P*	100873774	6	56484342	Missense	G	A	0.16	p.A1497V
9624	*RREB1*	6239	6	7247248	Missense	T	C	0.054	p.L1522P
9624	*SLFN12L*	100506736	17	33807186	Missense	C	G	0.097	p.A15P
9624	*TLN2*	83660	15	62985112	Missense	C	A	0.071	p.A396E
9624	*ZNF233*	353355	19	44778405	Missense	A	C	0.065	p.K531T
9623	*ARL15*	54622	5	53409226	Missense	G	A	0.449	p.R90W
9623	*ASXL1*	171023	20	31021635	Frame_Shift_Ins	G	GT	0.27	p.R545fs
9623	*BAZ2A*	11176	12	57009232	Missense	G	A	0.311	p.A101V
9623	*CASC3*	22794	17	38324174	Nonsense	C	T	0.333	p.Q575X
9623	*CCDC60*	160777	12	119942914	Missense	G	A	0.338	p.R230H
9623	*CEMIP*	57214	15	81224312	Missense	C	T	0.333	p.R909C
9623	*CLDN10*	9071	13	96205009	In_Frame_Del	TAGCACGC	T	0.244	p.1_6del
9623	*COL6A6*	131873	3	130281936	Missense	T	G	0.378	p.F30C
9623	*CR1*	1378	1	207796417	Splice_Site	A	C	0.323	c.7352+4A>C
9623	*CRB2*	286204	9	126133082	Missense	C	T	0.344	p.R584C
9623	*FAM198A*	729085	3	43073996	Missense	C	T	0.279	p.R81C
9623	*FBXO40*	51725	3	121340536	Missense	T	C	0.063	p.V87A
9623	*FER1L6*	654463	8	125047601	Missense	T	A	0.362	p.S790R
9623	*FHDC1*	85462	4	153896230	Missense	C	T	0.276	p.S596F
9623	*FLNC*	2318	7	128482693	Missense	C	T	0.214	p.T777I
9623	*GALNT16*	57452	14	69792727	Missense	G	A	0.316	p.R184H
9623	*GRIA2*	2891	4	158256830	Missense	C	T	0.333	p.P378L
9623	*GSDMC*	56169	8	130789697	Missense	C	T	0.321	p.R46H
9623	*HDAC9*	9734	7	18688277	Missense	C	A	0.211	p.Q475K
9623	*ISG15*	9636	1	949608	Missense	G	A	0.065	p.S83N
9623	*ITGA8*	8516	10	15649687	Missense	C	T	0.25	p.V585I
9623	*KALRN*	8997	3	124132413	Missense	C	T	0.384	p.R813C
9623	*KALRN*	8997	3	123987945	Missense	C	T	0.35	p.P269L
9623	*KIF2B*	84643	17	51901271	Missense	C	T	0.356	p.R293C
9623	*KLHL38*	340359	8	124659205	Missense	A	G	0.345	p.I467T
9623	*LHX5*	64211	12	113901201	Missense	C	T	0.235	p.A335T
9623	*LINGO1*	84894	15	77907582	Missense	G	A	0.531	p.R217W
9623	*.OC10192905*	101929057	6	90383952	Missense	A	G	0.286	p.M4373T
9623	*LYSMD4*	145748	15	100269680	Missense	G	C	0.057	p.A181G
9623	*MACROD2*	140733	20	16025281	Missense	A	T	0.359	p.I198L
9623	*MPO*	4353	17	56349062	Missense	C	T	0.5	p.A662T
9623	*MYT1*	4661	20	62863653	Missense	T	A	0.377	p.S938T
9623	*NBEAL2*	23218	3	47037954	Missense	A	C	0.385	p.D782A
9623	*NFKBIA*	4792	14	35872949	Missense	G	T	0.148	p.R95S
9623	*NID2*	22795	14	52507563	Missense	G	A	0.464	p.A611V
9623	*NOTCH4*	4855	6	32170336	Missense	C	T	0.333	p.C1091Y
9623	*NPAP1*	23742	15	24921090	Missense	G	A	0.429	p.A26T
9623	*NWD2*	57495	4	37435537	In_Frame_Del	CAAG	C	0.325	p.201_202del
9623	*OR10G8*	219869	11	123900676	Missense	T	G	0.277	p.V116G
9623	*OTOG*	340990	11	17618539	Missense	A	C	0.625	p.T1223P
9623	*PDXDC1*	23042	16	15122780	Missense	G	C	0.333	p.G417A
9623	*PLEKHS1*	79949	10	115534596	Missense	A	G	0.438	p.E264G
9623	*POTEE*	445582	2	132021547	Missense	C	T	0.16	p.P840L
9623	*POU4F2*	5458	4	147560497	Nonsense	C	T	0.444	p.R69X
9623	*PTPRG*	5793	3	62204600	Missense	T	A	0.321	p.V744E
9623	*PUM2*	23369	2	20511382	Missense	G	T	0.081	p.Q131K
9623	*SLC1A1*	6505	9	4576117	Missense	C	A	0.526	p.S331Y
9623	*SLIT2*	9353	4	20544188	Nonsense	C	T	0.242	p.R739X
9623	*SPATA18*	132671	4	52943071	Missense	T	A	0.261	p.N295K
9623	*STAG3*	10734	7	99796123	Missense	G	A	0.364	p.A424T
9623	*SYNE1*	23345	6	152763329	Missense	C	T	0.722	p.A1297T
9623	*TBX15*	6913	1	119427953	Missense	C	T	0.357	p.R298Q
9623	*THBS1*	7057	15	39874652	Missense	G	A	0.38	p.R109Q
9623	*TRAM1L1*	133022	4	118005706	Missense	G	A	0.333	p.R282W
9623	*TRPM7*	54822	15	50853939	Missense	T	C	0.393	p.N1845S
9623	*UNC5C*	8633	4	96137274	Splice_Site	C	T	0.255	c.1733+1G>A
9623	*VCAN*	1462	5	82817997	Missense	C	A	0.339	p.T1291K
9623	*WSCD2*	9671	12	108618630	Missense	C	T	0.071	p.T266I
9623	*ZC3HAV1*	56829	7	138793981	Frame_Shift_Del	AC	A	0.229	p.L32fs
9623	*ZFP36L2*	678	2	43451739	In_Frame_Del	CCTG	C	0.429	p.401_401del
9623	*ZMAT4*	79698	8	40554867	Missense	C	T	0.345	p.M82I
9623	*ZNF142*	7701	2	219509618	Missense	C	A	0.087	p.A541S
9623	*ZNF845*	91664	19	53854344	Missense	A	G	0.304	p.Q139R
9622	*ALMS1*	7840	2	73649987	Missense	A	C	0.075	p.I217L
9622	*ALPP*	250	2	233245026	Missense	G	A	0.2	p.R263H
9622	*ANPEP*	290	15	90349543	Missense	G	A	0.072	p.P91L
9622	*BMP4*	652	14	54418679	Missense	G	A	0.082	p.R88W
9622	*BPI*	671	20	36937420	Missense	A	G	0.068	p.I116V

9622	*BZRAP1*	9256	17	56383744	Missense	T	C	0.077	p.H1728R
9622	*CDC14B*	8555	9	99296809	Missense	C	A	0.15	p.D215Y
9622	*COL1A2*	1278	7	94055756	Missense	G	A	0.065	p.D1007N
9622	*CSE1L*	1434	20	47695100	Missense	A	C	0.2	p.N475H
9622	*DYSF*	8291	2	71827924	Missense	G	T	0.097	p.Q1297H
9622	*FAM129C*	199786	19	17654181	Missense	C	T	0.094	p.T493M
9622	*FAM131B*	9715	7	143053723	Missense	C	T	0.047	p.A307T
9622	*FAM173A*	65990	16	771835	Missense	C	A	0.073	p.A101D
9622	*GNAQ*	2776	9	80537112	Missense	T	A	0.086	p.T96S
9622	*GNAQ*	2776	9	80537223	Missense	T	G	0.049	p.M59L
9622	*JAK1*	3716	1	65325872	Missense	G	A	0.079	p.A417V
9622	*KHK*	3795	2	27317406	Missense	A	C	0.067	p.T91P
9622	*LOXL3*	84695	2	74760769	Missense	T	G	0.128	p.E742D
9622	*LRP5L*	91355	22	25750656	Missense	G	A	0.217	p.R188W
9622	*MRO*	83876	18	48346048	Missense	G	A	0.064	p.A15V
9622	*MROH2A*	339766	2	234725502	Missense	T	C	0.068	p.W981R
9622	*NECAB2*	54550	16	84035446	Missense	C	G	0.086	p.L353V
9622	*NLRP3*	114548	1	247587317	Missense	G	A	0.048	p.G191D
9622	*OR10H1*	26539	19	15918177	Missense	G	T	0.135	p.A224D
9622	*PAQR6*	79957	1	156215945	Missense	C	G	0.14	p.V47L
9622	*SDK2*	54549	17	71348705	Missense	T	C	0.051	p.I1889V
9622	*SF3B2*	10992	11	65825521	Splice_Site	G	T	0.4	c.778-4G>T
9622	*SH3RF2*	153769	5	145442133	Missense	G	A	0.04	p.V687I
9622	*SLC5A9*	200010	1	48695030	Missense	G	T	0.2	p.V160F
9622	*STAT3*	6774	17	40475060	In_Frame_Del	CCTT	C	0.188	p.616_617del
9622	*TMPRSS2*	7113	21	42852497	Missense	C	T	0.061	p.V160M
9622	*TRPV4*	59341	12	110224646	Splice_Site	C	T	0.091	c.2029-4G>A
9622	*TUBGCP3*	10426	13	113176740	Missense	C	A	0.065	p.D547Y
9622	*UBR3*	130507	2	170734017	Missense	T	A	0.143	p.N286K
9622	*VNN1*	8876	6	133035098	Missense	G	A	0.071	p.T26I
9622	*ZNF479*	90827	7	57193730	Missense	T	C	0.333	p.H86R
9616	*ADAMTSL1*	92949	9	18680437	Missense	T	C	0.435	p.Y422H
9616	*ADH1A*	124	4	100200616	Missense	T	C	0.265	p.N357S
9616	*ALDOA*	226	16	30078612	Nonsense	A	T	0.235	p.K13X
9616	*ANGPTL1*	9068	1	178820338	Missense	T	G	0.22	p.I468L
9616	*ANKRD11*	29123	16	89351199	Missense	G	C	0.188	p.S584C
9616	*ASH1L*	55870	1	155307524	Missense	A	C	0.156	p.L2941W
9616	*BBS9*	27241	7	33644833	Nonsense	G	T	0.263	p.E887X
9616	*CAMK2B*	816	7	44272450	Missense	C	T	0.14	p.V383I
9616	*CDKN2A*	1029	9	21994286	Nonsense	G	T	0.329	p.C15X
9616	*CISH*	1154	3	50645426	Missense	C	T	0.188	p.R147H
9616	*CMYA5*	202333	5	79034412	Missense	C	T	0.191	p.P3275L
9616	*FAM166A*	401565	9	140139794	Missense	C	T	0.467	p.A163T
9616	*GPRC6A*	222545	6	117130704	Missense	G	A	0.111	p.P91S
9616	*GRIN2A*	2903	16	9858147	Missense	T	G	0.25	p.N1085T
9616	*HIP1R*	9026	12	123345987	Missense	G	C	0.7	p.A1029P
9616	*IRAK3*	11213	12	66641705	Missense	G	T	0.171	p.M515I
9616	*KALRN*	8997	3	124437917	Missense	G	A	0.278	p.G2854D
9616	*KIAA0226L*	80183	13	46946157	Missense	C	T	0.071	p.G152R
9616	*KMT2D*	8085	12	49435696	Splice_Site	T	C	0.238	c.6183+4A>G
9616	*KSR2*	283455	12	118298234	Missense	G	T	0.145	p.S32R
9616	*L1TD1*	54596	1	62673208	Missense	C	T	0.073	p.A303V
9616	*LAMA5*	3911	20	60887592	Splice_Site	T	C	0.11	c.9228-4A>G
9616	*LRRC1*	55227	6	53787583	Missense	T	G	0.16	p.S523A
9616	*NBPF20*	100288142	1	145075705	Missense	C	T	0.083	p.R53H
9616	*NOS3*	4846	7	150698654	Missense	C	T	0.071	p.A484V
9616	*PBX1*	5087	1	164768975	Nonsense	C	T	0.127	p.R184X
9616	*PCDHGA3*	56112	5	140751765	Missense	G	A	0.206	p.A602T
9616	*PCDHGB2*	56103	5	140746223	Missense	G	C	0.277	p.A776P
9616	*PCOLCE-AS1*	100129845	7	100187845	Frame_Shift_Del	GGCCA	G	0.144	p.G63fs
9616	*PDLIM5*	10611	4	95532347	Missense	A	C	0.276	p.T335P
9616	*PDS5B*	23047	13	33225940	Splice_Site	G	A	0.143	c.109-1G>A
9616	*PRDM9*	56979	5	23518031	Missense	C	G	0.324	p.H115D
9616	*PTPN14*	5784	1	214560201	Missense	G	A	0.276	p.T351M
9616	*RNU6-81P*	100873780	2	132237927	Missense	C	A	0.156	p.R221S
9616	*RP1L1*	94137	8	10464785	Missense	C	G	0.2	p.D2275H
9616	*SH3RF3*	344558	2	110015203	Missense	G	A	0.213	p.R368Q
9616	*SLC13A1*	6561	7	122774474	Missense	G	C	0.105	p.L308V
9616	*SLC26A8*	116369	6	35945009	Missense	A	G	0.139	p.L382P
9616	*SLC4A5*	57835	2	74489356	Missense	C	T	0.312	p.R240H
9616	*SORBS2*	8470	4	186560075	Missense	C	T	0.129	p.A446T
9616	*SUN5*	140732	20	31583459	Missense	G	A	0.244	p.A167V
9616	*TBC1D10A*	83874	22	30688780	Missense	G	A	0.222	p.R371C
9616	*TLR3*	7098	4	187004302	Nonsense	C	T	0.259	p.R488X
9616	*TUBA3D*	113457	2	132240243	Missense	A	T	0.086	p.D392V
9616	*UBR2*	23304	6	42643830	Nonsense	C	T	0.25	p.Q1430X
9616	*UNC79*	57578	14	94120180	Missense	T	A	0.2	p.L1921Q
9616	*WDR64*	128025	1	241815799	Missense	A	G	0.18	p.K5E
9616	*XPO1*	7514	2	61724012	Splice_Site	A	G	0.103	c.888+2T>C
9616	*ZBTB7B*	51043	1	154987461	Missense	G	T	0.292	p.A143S

9616	*ZKSCAN2*	342357	16	25266632	Missense	C	T	0.211	p.E161K
9616	*ZNF354A*	6940	5	178140480	Missense	C	G	0.147	p.E133D
8751	*ADRBK2*	157	22	26068297	Missense	G	T	0.143	p.V180F
8751	*ALG6*	29929	1	63881564	Missense	G	T	0.133	p.S308I
8751	*AMDHD2*	51005	16	2578503	Missense	C	T	0.122	p.R305W
8751	*ANKRD30B*	374860	18	14748488	Missense	C	T	0.079	p.R24W
8751	*ANKRD35*	148741	1	145558841	Missense	C	T	0.104	p.R154C
8751	*ASAP3*	55616	1	23782472	Missense	C	T	0.118	p.G84S
8751	*ASPSCR1*	79058	17	79935602	Missense	C	A	0.125	p.T27K
8751	*ASTN1*	460	1	176926934	Missense	G	T	0.061	p.D589E
8751	*ATP7B*	540	13	52524488	Missense	T	C	0.066	p.K721R
8751	*ATP7B*	540	13	52548140	Missense	A	C	0.048	p.S295A
8751	*ATXN1*	6310	6	16327398	Missense	C	A	0.046	p.V382L
8751	*B4GALT1*	2683	9	33166805	Missense	G	T	0.062	p.H121Q
8751	*BCYRN1*	618	X	70444065	Missense	G	T	0.053	p.V170F
8751	*BRCA1*	672	17	41243950	Nonsense	G	A	0.137	p.Q1200X
8751	*C2orf54*	79919	2	241831296	Missense	C	G	0.04	p.A21P
8751	*C5orf42*	65250	5	37238998	Missense	C	A	0.222	p.C300F
8751	*CACNA1H*	8912	16	1256117	Missense	G	A	0.167	p.V873M
8751	*CDKL2*	8999	4	76539579	Nonsense	G	A	0.091	p.R75X
8751	*CELSR3*	1951	3	48699499	Missense	T	A	0.184	p.N190I
8751	*CEP164*	22897	11	117252460	Missense	C	A	0.25	p.P485T
8751	*CEP170B*	283638	14	105355905	In_Frame_Del	GGCTCCC	T	0.07	p.1195_1199del
8751	*CNTLN*	54875	9	17340838	Missense	A	C	0.156	p.D553A
8751	*COL4A5*	1287	X	107845184	Missense	C	A	0.15	p.P704H
8751	*CSNK1A1*	1452	5	148891427	Missense	T	C	0.286	p.D230G
8751	*CUBN*	8029	10	16957083	Missense	G	T	0.083	p.D2433E
8751	*CYB5R2*	51700	11	7689765	Missense	G	A	0.152	p.T139M
8751	*DCTN5*	84516	16	23669925	Missense	C	A	0.056	p.P72Q
8751	*DDB1*	1642	11	61084007	Missense	C	G	0.114	p.E420Q
8751	*DENND6B*	414918	22	50751508	Missense	C	A	0.103	p.Q459H
8751	*DHX15*	1665	4	24557977	Missense	C	A	0.064	p.R253L
8751	*DNAH17*	8632	17	76454743	Missense	C	G	0.105	p.S3294T
8751	*DNAH5*	1767	5	13931340	Missense	C	T	0.097	p.G24E
8751	*DOCK3*	1795	3	51417592	Missense	C	A	0.183	p.P1846H
8751	*DSC3*	1825	18	28598703	Missense	T	C	0.115	p.I336V
8751	*EIF4A1*	1973	17	7480933	Missense	T	C	0.059	p.I272T
8751	*ERCC6L2*	375748	9	98703807	Missense	C	A	0.167	p.A619D
8751	*ERICH6*	131831	3	150421324	Missense	G	T	0.061	p.T121K
8751	*ERMP1*	79956	9	5830763	Missense	C	A	0.105	p.A202S
8751	*FAM154A*	158297	9	18928895	Missense	G	T	0.165	p.P194T
8751	*FAM47B*	170062	X	34961516	Missense	C	T	0.128	p.R190W
8751	*FBXO16*	157574	8	28321131	Missense	G	T	0.061	p.Q114K
8751	*FLII*	2314	17	18154618	Missense	G	T	0.057	p.H519Q
8751	*FZD10*	11211	12	130647715	Missense	G	T	0.064	p.E76D
8751	*GCFC2*	6936	2	75893054	Splice_Site	C	A	0.125	c.2228+1G>T
8751	*GPR39*	2863	2	133402778	Missense	G	A	0.093	p.A321T
8751	*GRK6*	2870	5	176863227	Missense	T	A	0.043	p.V404D
8751	*GUCY1B3*	2983	4	156725760	Missense	C	G	0.13	p.H499D
8751	*GYS2*	2998	12	21713332	Missense	C	G	0.108	p.R386P
8751	*HIST1H4F*	8361	6	26240948	Missense	T	G	0.178	p.Y99D
8751	*HLA-DQB2*	3120	6	32725563	Missense	G	T	0.143	p.H248Q
8751	*HOOK1*	51361	1	60325897	Missense	C	A	0.125	p.L477I
8751	*IGDCC4*	57722	15	65686839	Missense	C	A	0.058	p.D542Y
8751	*INTS7*	25896	1	212118137	Missense	G	T	0.048	p.Q864K
8751	*KIAA0391*	9692	14	35739664	Missense	G	T	0.064	p.R494S
8751	*KIAA1109*	84162	4	123095771	Missense	G	T	0.125	p.R86L
8751	*KIF13B*	23303	8	28929734	Missense	C	A	0.081	p.V1541F
8751	*KIF7*	374654	15	90191868	Missense	G	T	0.07	p.T354K
8751	*LAMA2*	3908	6	129511346	Splice_Site	G	T	0.067	c.1468-4G>T
8751	*LENG1*	79165	19	54659646	Missense	C	A	0.086	p.R203L
8751	*LTN1*	26046	21	30342977	Missense	C	A	0.071	p.V404F
8751	*MAGIX*	79917	X	49021637	Missense	G	T	0.1	p.R206L
8751	*MAP1A*	4130	15	43816082	Missense	C	T	0.13	p.T804M
8751	*MAST4*	375449	5	66055633	Missense	C	A	0.064	p.Q154K
8751	*MCTP1*	79772	5	94206594	Missense	C	G	0.137	p.D742H
8751	*MIR548AZ*	102466162	14	64588745	Missense	C	A	0.333	p.P4392T
8751	*MROH2A*	339766	2	234688067	Missense	C	A	0.154	p.D21E
8751	*MYO1G*	64005	7	45006306	Missense	G	C	0.224	p.F638L
8751	*NCOA4*	8031	10	51586609	Splice_Site	G	T	0.1	c.1888-1G>T
8751	*NUS1*	116150	6	118014285	Missense	A	G	0.182	p.K166E
8751	*OR8U8*	504189	11	56143158	Missense	A	G	0.088	p.H20R
8751	*P4HA2*	8974	5	131554262	Nonsense	G	A	0.086	p.Q20X
8751	*PASD1*	139135	X	150842486	Missense	G	A	0.041	p.S668N
8751	*PATE1*	160065	11	125617688	Missense	A	T	0.068	p.E73V
8751	*PCDH10*	57575	4	134072874	Missense	G	A	0.156	p.A527T
8751	*PIBF1*	10464	13	73396015	Missense	C	T	0.105	p.S234F
8751	*PIBF1*	10464	13	73396017	Missense	G	A	0.105	p.E235K
8751	*PITRM1*	10531	10	3193557	Splice_Site	AAAAGAA	T	0.143	.
8751	*PKHD1L1*	93035	8	110450623	Missense	G	T	0.154	p.S1233I

8751	*PNKD*	25953	2	219143777	Missense	C	A	0.067	p.V136F
8751	*PRPF6*	24148	20	62616356	Missense	G	T	0.094	p.D113Y
8751	*PRSS37*	136242	7	141536287	Nonsense	C	A	0.077	p.E205X
8751	*PTPN13*	5783	4	87705670	Missense	C	A	0.133	p.T2031N
8751	*PTPN4*	5775	2	120723130	Missense	G	T	0.111	p.V823F
8751	*PYGL*	5836	14	51382134	Missense	G	T	0.045	p.N441K
8751	*RAI14*	26064	5	34823332	Missense	G	A	0.073	p.R465Q
8751	*RBCK1*	10616	20	389407	Nonsense	C	T	0.081	p.R9X
8751	*RNF150*	57484	4	142053846	Missense	C	A	0.082	p.W39C
8751	*RPE*	6120	2	210880950	Missense	A	G	0.1	p.E54G
8751	*RRP9*	9136	3	51969661	Missense	G	T	0.053	p.H261Q
8751	*RYR2*	6262	1	237995876	Missense	C	A	0.222	p.Q4945K
8751	*SEMA5B*	54437	3	122629803	Missense	G	T	0.111	p.H1115N
8751	*SERGEF*	26297	11	18026044	Missense	C	T	0.111	p.G131S
8751	*SGK494*	124923	17	26940645	Missense	C	A	0.088	p.G46V
8751	*SIGLEC9*	27180	19	51630482	Missense	C	A	0.071	p.A315E
8751	*SMC4*	10051	3	160143863	Missense	G	T	0.133	p.R802L
8751	*SORCS3*	22986	10	106976871	Missense	C	A	0.143	p.H909N
8751	*SPACA1*	81833	6	88769192	Missense	G	T	0.125	p.D166Y
8751	*SPNS3*	201305	17	4349351	Missense	G	T	0.2	p.W137C
8751	*STAB2*	55576	12	104149092	Missense	C	A	0.111	p.L2243M
8751	*STPG2*	285555	4	99049712	Missense	C	A	0.154	p.S85I
8751	*SYNE1*	23345	6	152453297	Missense	C	A	0.062	p.S8685I
8751	*TACO1*	51204	17	61684795	Nonsense	G	T	0.111	p.E222X
8751	*TARS*	6897	5	33462241	Missense	C	G	0.143	p.H623D
8751	*TC2N*	123036	14	92278674	Missense	G	T	0.111	p.H95N
8751	*TNK1*	8711	17	7287440	Missense	A	G	0.19	p.D245G
8751	*TTC13*	79573	1	231114501	Missense	C	A	0.133	p.V26F
8751	*TTC24*	164118	1	156553200	Missense	C	A	0.043	p.D370E
8751	*UBD*	10537	6	29527451	Splice_Site	C	A	0.133	c.27+1G>T
8751	*UPF2*	26019	10	12071002	Missense	T	C	0.087	p.N296S
8751	*USH2A*	7399	1	216262424	Missense	T	C	0.136	p.K1606E
8751	*UTRN*	7402	6	145069554	Missense	C	A	0.043	p.D2704E
8751	*WIPF3*	644150	7	29915545	Missense	C	A	0.081	p.Q64K
8751	*ZBBX*	79740	3	166958708	Splice_Site	C	T	0.087	c.2277-1G>A
8751	*ZC3H3*	23144	8	144547955	Missense	C	A	0.06	p.V747L
8751	*ZDHHC8*	29801	22	20128826	Missense	C	A	0.064	p.S327R
8221	*ALPPL2*	251	2	233273244	Missense	C	A	0.153	p.L273M
8221	*AP3S1*	1176	5	115249078	Missense	C	T	1	p.P158L
8221	*ARHGAP22*	58504	10	49667878	Missense	G	A	0.421	p.R170C
8221	*BASP1*	10409	5	17275887	Missense	G	A	0.282	p.A188T
8221	*BBS9*	27241	7	33407475	Splice_Site	G	A	0.379	c.1789+1G>A
8221	*BRD8*	10902	5	137503739	Missense	G	A	0.417	p.S297F
8221	*C1QB*	713	1	22986040	Missense	T	C	0.283	p.C31R
8221	*CA3*	761	8	86358504	Missense	C	T	0.232	p.P214L
8221	*CAMSAP3*	57662	19	7677450	Missense	G	C	0.366	p.V718L
8221	*CEP164*	22897	11	117253636	Missense	A	C	0.371	p.T568P
8221	*CNTROB*	116840	17	7838452	Missense	A	C	0.342	p.T195P
8221	*CTNNA2*	1496	2	80530511	Missense	G	A	0.276	p.S145L
8221	*CYP2F1*	1572	19	41633809	Missense	G	A	0.308	p.R433H
8221	*DDX4*	54514	5	55088574	Missense	C	T	0.068	p.P470S
8221	*DMXL2*	23312	15	51772183	Missense	T	C	0.583	p.M2240V
8221	*DNAAF1*	123872	16	84205892	Missense	G	A	0.392	p.E519K
8221	*DNAH5*	1767	5	13727663	Missense	G	A	0.582	p.R3996C
8221	*ENTHD2*	146705	17	79202760	Missense	C	G	0.346	p.E516Q
8221	*FAM205A*	259308	9	34725438	Missense	A	G	0.5	p.L600S
8221	*FBXO16*	157574	8	28314360	Missense	G	T	0.333	p.P144T
8221	*FKRP*	79147	19	47259734	Missense	G	C	0.231	p.E343Q
8221	*FLG*	2312	1	152284798	Missense	G	T	0.125	p.S855Y
8221	*GALNS*	2588	16	88889073	Missense	G	A	0.359	p.H430Y
8221	*GPAT2*	150763	2	96688929	Missense	G	A	0.079	p.R692C
8221	*GPR98*	84059	5	90049445	Missense	G	T	0.361	p.D3726Y
8221	*HECTD4*	283450	12	112688167	Missense	A	C	0.435	p.V1110G
8221	*HRNR*	388697	1	152190938	Missense	C	T	0.273	p.R1056H
8221	*HRNR*	388697	1	152187510	Missense	C	T	0.07	p.G2199S
8221	*IL7R*	3575	5	35871314	Missense	C	T	0.333	p.T179M
8221	*LOC401052*	401052	3	10049098	Missense	T	G	0.308	p.H96P
8221	*MBD1*	4152	18	47806297	Frame_Shift_Del	GCGAA	G	0.208	p.F21fs
8221	*MPLKIP*	136647	7	40172713	Nonsense	T	GGCTTAT	0.085	p.Q162_Q163delinsLX
8221	*MUC17*	140453	7	100683771	Missense	G	A	0.065	p.S3025N
8221	*MUC6*	4588	11	1017325	Missense	A	C	0.067	p.Y1826D
8221	*MYADM*	91663	19	54377469	Missense	C	G	0.356	p.T229S
8221	*NBPF8*	728841	1	147597264	Missense	G	C	1	p.L120V
8221	*OR4A15*	81328	11	55135435	Missense	C	T	0.053	p.P26S
8221	*OR4N2*	390429	14	20296064	Missense	T	C	0.243	p.F153L
8221	*OR52E6*	390078	11	5863060	Missense	T	C	0.041	p.E23G
8221	*OR7D4*	125958	19	9324835	Missense	C	T	0.068	p.G227R
8221	*POTEF*	728378	2	130832256	Missense	A	G	0.067	p.V930A
8221	*PRKCG*	5582	19	54403979	Frame_Shift_Ins	G	GA	0.287	p.G517fs
8221	*QRICH2*	84074	17	74287594	Missense	G	A	0.047	p.H906Y

8221	*RHOF*	54509	12	122217559	Missense	C	T	0.044	p.A161T
8221	*SCN5A*	6331	3	38674610	Missense	C	A	0.207	p.K63N
8221	*SEMA5A*	9037	5	9224845	Missense	C	A	0.278	p.R196L
8221	*SGTB*	54557	5	64981284	Missense	G	T	0.279	p.S130R
8221	*SIGLEC8*	27181	19	51957531	Missense	G	A	0.355	p.A396V
8221	*SLC30A4*	7782	15	45814162	Missense	C	T	0.352	p.G131S
8221	*TMEM47*	83604	X	34675139	Missense	G	A	0.073	p.S3L
8221	*TRIM56*	81844	7	100731821	In_Frame_Del	'GGAGATG(	C	0.208	p.410_418del
8221	*TYW1*	55253	7	66563540	Missense	T	C	0.064	p.V466A
8221	*UGT2A1*	10941	4	70465059	Nonsense	G	A	0.305	p.R301X
8221	*ZDHHC14*	79683	6	158093930	Missense	A	G	0.489	p.M400V
8219	*4-Mar*	57574	2	217234619	Missense	G	A	0.175	p.P122L
8219	*ABCA4*	24	1	94528713	Missense	C	T	0.167	p.R572Q
8219	*ABHD17A*	81926	19	1879987	Missense	A	C	0.058	p.S205A
8219	*ADORA3*	140	1	112045880	Missense	C	T	0.098	p.V33I
8219	*AJAP1*	55966	1	4772717	Missense	G	A	0.032	p.G263R
8219	*ALPPL2*	251	2	233273207	Splice_Site	G	A	0.028	c.784-4G>A
8219	*AMOTL1*	154810	11	94592756	Missense	C	T	0.173	p.R671W
8219	*ANGPTL4*	51129	19	8429272	Missense	G	A	0.134	p.A23T
8219	*BRPF3*	27154	6	36178239	Missense	G	A	0.03	p.E705K
8219	*C3orf52*	79669	3	111821702	Missense	G	A	0.143	p.D96N
8219	*C9orf9*	11092	9	135762805	Missense	T	G	0.267	p.V65G
8219	*CASQ2*	845	1	116310967	Missense	T	C	0.044	p.T66A
8219	*CCDC158*	339965	4	77305725	Missense	C	A	0.152	p.A128S
8219	*CCDC40*	55036	17	78032413	Missense	C	T	0.142	p.T427M
8219	*CDH16*	1014	16	66949992	Missense	G	A	0.126	p.R134W
8219	*CDH3*	1001	16	68725767	Missense	G	T	0.103	p.C647F
8219	*CDHR5*	53841	11	624658	Missense	C	T	0.117	p.V54I
8219	*CDKN2A*	1029	9	21971120	Nonsense	G	A	0.137	p.R80X
8219	*CELA2B*	51032	1	15808872	Missense	G	A	0.048	p.D114N
8219	*CELSR3*	1951	3	48697921	Missense	C	T	0.102	p.R716H
8219	*CEP164*	22897	11	117253636	Missense	A	C	0.417	p.T568P
8219	*CHD7*	55636	8	61765616	Missense	G	A	0.071	p.R2111Q
8219	*CLPTM1L*	81037	5	1325888	Missense	C	A	0.171	p.R375I
8219	*COL21A1*	81578	6	55925584	Missense	A	G	0.119	p.F788L
8219	*CUX2*	23316	12	111744758	Missense	C	T	0.057	p.R298W
8219	*CYP2A13*	1553	19	41595958	Missense	C	T	0.148	p.A117V
8219	*CYP4A11*	1579	1	47395874	Missense	A	C	0.093	p.I491M
8219	*DAB2IP*	153090	9	124543775	Missense	A	T	0.094	p.D1088V
8219	*DNAH1*	25981	3	52414111	Missense	C	A	0.147	p.S2523Y
8219	*DNMT1*	1786	19	10291181	Missense	T	C	0.071	p.H97R
8219	*EME2*	197342	16	1826148	Missense	G	A	0.142	p.R350H
8219	*EPHA7*	2045	6	93956703	Missense	C	A	0.214	p.V845F
8219	*ERBB2*	2064	17	37876044	Missense	G	T	0.056	p.V620L
8219	*EVX1*	2128	7	27285725	Missense	G	T	0.062	p.G302V
8219	*FAM135A*	57579	6	71187020	Missense	A	C	0.25	p.H176P
8219	*FAM135A*	57579	6	71248050	Nonsense	C	T	0.091	p.R1196X
8219	*FBXO41*	150726	2	73496751	Missense	G	A	0.033	p.S3L
8219	*FHDC1*	85462	4	153896380	Missense	G	A	0.026	p.R646Q
8219	*FXYD6*	53826	11	117711896	Missense	C	T	0.078	p.R59H
8219	*GCC2*	9648	2	109066089	Missense	G	C	0.188	p.D3H
8219	*GPAT2*	150763	2	96688961	Missense	G	A	0.057	p.S681L
8219	*GPAT2*	150763	2	96688929	Missense	G	A	0.051	p.R692C
8219	*GRM3*	2913	7	86415913	Missense	G	A	0.118	p.A269T
8219	*GSTM1*	2944	1	110233138	Missense	G	C	0.088	p.K173N
8219	*GYS2*	2998	12	21733432	Missense	C	G	0.161	p.Q49H
8219	*H6PD*	9563	1	9322153	Missense	C	T	0.027	p.R261C
8219	*HCAR3*	8843	12	123201205	Missense	G	A	0.056	p.A27V
8219	*HDAC4*	9759	2	240111555	Missense	C	T	0.029	p.E105K
8219	*KLC1*	3831	14	104129155	Missense	G	A	0.035	p.E230K
8219	*KMT2D*	8085	12	49433256	Nonsense	G	A	0.11	p.Q2731X
8219	*KMT2D*	8085	12	49420655	Missense	A	G	0.105	p.C5032R
8219	*KRTAP10-6*	386674	21	46011891	Missense	C	T	0.07	p.V159I
8219	*KRTAP4-7*	100132476	17	39240580	Missense	G	A	0.145	p.R41H
8219	*LOC401052*	401052	3	10049098	Missense	T	G	0.5	p.H96P
8219	*LPIN3*	64900	20	39978786	Missense	C	G	0.138	p.S284C
8219	*MGA*	23269	15	42021428	Nonsense	C	T	0.172	p.R1242X
8219	*MPV17L*	255027	16	15501888	Missense	T	C	0.286	p.W147R
8219	*MUC17*	140453	7	100681460	Missense	A	G	0.051	p.T2255A
8219	*MUC17*	140453	7	100684419	Missense	T	C	0.038	p.I3241T
8219	*MUC4*	4585	3	195513338	Missense	C	T	0.25	p.A1705T
8219	*MUC5B*	727897	11	1268036	Missense	C	T	0.3	p.P3309L
8219	*MYH7B*	57644	20	33588866	Nonsense	G	T	0.093	p.E1836X
8219	*MYH9*	4627	22	36702602	Missense	A	T	0.132	p.L632Q
8219	*NBPF12*	149013	1	146400170	Missense	G	A	0.286	p.G216S
8219	*NEDD4*	4734	15	56141063	Missense	G	T	0.1	p.P457T
8219	*NOTCH2*	4853	1	120480552	Missense	A	G	0.182	p.C1089R
8219	*NUDT10*	170685	X	51076083	Missense	C	G	0.2	p.P89R
8219	*OBSCN*	84033	1	228487637	Missense	C	A	0.143	p.A4510D
8219	*OR4M2*	390538	15	22369506	Missense	A	G	0.051	p.K311E

8219	*OR4N2*	390429	14	20295878	Missense	A	G	0.057	p.I91V
8219	*OR4N4*	283694	15	22383017	Missense	G	C	0.041	p.R182P
8219	*OR4N5*	390437	14	20612098	Missense	A	C	0.068	p.L68F
8219	*OR4Q3*	441669	14	20216004	Missense	C	A	0.051	p.Q140K
8219	*OR5H14*	403273	3	97868278	Missense	T	C	0.286	p.F17L
8219	*PABPC1*	26986	8	101724623	Frame_Shift_Ins	C	CA	0.303	p.E313fs
8219	*PABPC1*	26986	8	101724625	Frame_Shift_Del	CTT	C	0.286	p.K312fs
8219	*PABPC1*	26986	8	101724606	Missense	G	A	0.214	p.T319I
8219	*PABPC1*	26986	8	101717832	Missense	G	C	0.1	p.Q558E
8219	*PARD3B*	117583	2	205969205	Missense	T	C	0.205	p.L187P
8219	*PCMT1*	5110	6	150114745	Missense	G	A	0.075	p.V178I
8219	*PCSK6*	5046	15	101970191	Splice_Site	C	G	0.082	c.732+1G>C
8219	*PEG3*	5178	19	57325494	Missense	G	A	0.148	p.P1439L
8219	*PER3*	8863	1	7845542	Missense	T	G	0.089	p.M57R
8219	*PKHD1L1*	93035	8	110476775	Nonsense	C	T	0.178	p.R2572X
8219	*PPP1R16A*	84988	8	145726525	Missense	C	T	0.117	p.R351W
8219	*PPP1R37*	284352	19	45649680	Missense	G	A	0.044	p.E676K
8219	*RBM20*	282996	10	112572151	Missense	C	T	0.093	p.R666W
8219	*REG3G*	130120	2	79254164	Missense	C	A	0.133	p.A67D
8219	*RIMBP2*	23504	12	130935849	Missense	C	T	0.093	p.G115D
8219	*RNF126*	55658	19	651633	Missense	C	T	0.098	p.E141K
8219	*SEC14L5*	9717	16	5057362	Missense	C	T	0.061	p.P483S
8219	*SEC23A*	10484	14	39543728	Missense	C	T	0.158	p.E332K
8219	*SF3B3*	23450	16	70563087	Missense	C	G	0.19	p.R128G
8219	*SGPP1*	81537	14	64153157	Missense	C	T	0.109	p.C331Y
8219	*SH3PXD2A*	9644	10	105363548	Splice_Site	T	G	0.118	c.1345-2A>C
8219	*SIGLEC12*	89858	19	52002827	Missense	C	T	0.092	p.D318N
8219	*SLC30A10*	55532	1	220101599	Missense	G	A	0.116	p.R62W
8219	*SMC4*	10051	3	160150844	Missense	G	T	0.21	p.K1162N
8219	*SND1*	27044	7	127669034	Missense	G	A	0.175	p.H554Y
8219	*SORCS1*	114815	10	108923879	Missense	C	T	0.132	p.G136R
8219	*SPAM1*	6677	7	123593649	Missense	A	T	0.102	p.I9F
8219	*SPDYE6*	729597	7	101988870	Missense	G	A	0.214	p.R335C
8219	*STK25*	10494	2	242438696	Missense	G	C	0.145	p.I187M
8219	*STK39*	27347	2	168986103	Nonsense	AG	A	0.236	p.L346X
8219	*STXBP6*	29091	14	25288326	Missense	G	A	0.149	p.R176C
8219	*TBC1D26*	353149	17	15641610	Missense	A	G	0.067	p.Y99C
8219	*TMC7*	79905	16	19070833	Missense	C	A	0.157	p.S708Y
8219	*TMEM14B*	81853	6	10756728	Missense	C	T	0.167	p.R108C
8219	*TMEM151A*	256472	11	66061904	Missense	C	T	0.053	p.R63C
8219	*TUBB4B*	10383	9	140137185	Missense	C	T	0.035	p.S172L
8219	*UBL4B*	164153	1	110655326	Missense	C	T	0.12	p.S57F
8219	*ULBP2*	80328	6	150263287	Missense	C	G	0.224	p.R27G
8219	*UPF1*	5976	19	18966795	Nonsense	C	T	0.079	p.Q536X
8219	*VASP*	7408	19	46025691	Missense	C	T	0.082	p.S191L
8219	*VSX1*	30813	20	25059442	Missense	C	T	0.041	p.R217H
8219	*XIRP2*	129446	2	168106146	Nonsense	T	A	0.118	p.Y2748X
8219	*ZFHX4*	79776	8	77617275	Missense	G	A	0.101	p.V318I
8219	*ZNF222*	7673	19	44536834	Missense	C	G	0.043	p.T336S
8219	*ZNF273*	10793	7	64388350	Missense	G	T	0.067	p.C215F
8219	*ZNF423*	23090	16	49670092	Missense	G	A	0.121	p.R991C
8219	*ZNF598*	90850	16	2051014	Missense	C	T	0.032	p.E398K
8219	*ZNF681*	148213	19	23926688	Missense	A	G	0.5	p.I555T
8219	*ZNF780B*	163131	19	40541357	Missense	C	T	0.167	p.G470E
8219	*ZNF93*	81931	19	20044524	Missense	A	G	0.667	p.K254E
8210	*ADAMTS8*	11095	11	130275472	Missense	C	A	0.149	p.S884I
8210	*ADNP2*	22850	18	77895175	Missense	C	T	0.277	p.P627S
8210	*AKAP6*	9472	14	33293295	Missense	G	C	0.231	p.E2092D
8210	*ALDH1L1*	10840	3	125873433	Missense	G	T	0.37	p.D228E
8210	*AUP1*	550	2	74756261	Missense	T	A	0.091	p.T113S
8210	*BBOX1-AS1*	103695435	11	27141284	Missense	A	G	0.194	p.K243R
8210	*CACNA1C*	775	12	2675592	Missense	T	C	0.35	p.Y505H
8210	*CARD6*	84674	5	40852749	Missense	C	T	0.239	p.P439S
8210	*CEP120*	153241	5	122685675	Nonsense	G	A	0.169	p.R897X
8210	*CES2*	8824	16	66973233	Missense	C	A	0.154	p.A196D
8210	*CHSY3*	337876	5	129241112	Missense	G	A	0.361	p.R197H
8210	*CNTROB*	116840	17	7838452	Missense	A	C	0.333	p.T195P
8210	*CNTROB*	116840	17	7838356	Missense	G	A	0.297	p.E163K
8210	*CSMD1*	64478	8	2910136	Missense	T	A	0.089	p.N2503I
8210	*DDX25*	29118	11	125788550	Missense	G	A	0.196	p.V356M
8210	*DENND2A*	27147	7	140301486	Missense	A	G	0.242	p.S238P
8210	*DHX9*	1660	1	182812436	Missense	T	G	0.333	p.V40G
8210	*ELL3*	80237	15	44069069	Missense	G	C	0.048	p.Q11E
8210	*FAM198A*	729085	3	43074074	Missense	T	A	0.25	p.S107T
8210	*FANCA*	2175	16	89839766	Missense	G	C	0.083	p.P643A
8210	*FBXO28*	23219	1	224301957	Missense	G	T	0.055	p.Q42H
8210	*FEZF2*	55079	3	62357891	Missense	A	G	0.194	p.L218P
8210	*FMO3*	2328	1	171086458	Missense	G	A	0.255	p.R492Q
8210	*FSIP2*	401024	2	186672791	Missense	T	G	0.233	p.V6342G
8210	*GALNT6*	11226	12	51773546	Missense	C	G	0.4	p.R7P

8210	GOLGB1	2804	3	121413186	Missense	C	T	0.124	p.E2057K
8210	GPR62	118442	3	51989672	Missense	G	A	0.073	p.A2T
8210	GUCY1A2	2977	11	106681150	Missense	A	C	0.235	p.F421V
8210	HTR3D	200909	3	183756702	Missense	G	A	0.047	p.R435H
8210	HTRA3	94031	4	8293220	Nonsense	C	T	0.097	p.Q278X
8210	IGSF9B	22997	11	133790369	Missense	G	A	0.119	p.S1084F
8210	ISM1	140862	20	13279843	Missense	A	G	0.246	p.T378A
8210	ITGAX	3687	16	31374535	Missense	C	G	0.06	p.P517R
8210	ITPR3	3710	6	33643602	Missense	A	T	0.3	p.Q1084L
8210	KIAA1217	56243	10	24832383	Missense	T	C	0.076	p.L1395P
8210	KRT6A	3853	12	52885485	Missense	T	G	0.222	p.E192D
8210	LOC401052	401052	3	10049098	Missense	T	G	0.545	p.H96P
8210	MDN1	23195	6	90428947	Splice_Site	A	G	0.293	c.5968-3T>C
8210	MEGF6	1953	1	3410641	Missense	A	G	0.25	p.C1393R
8210	MGAT4C	25834	12	86373774	Missense	G	T	0.274	p.L244I
8210	MLH1	4292	3	37070421	Missense	A	G	0.244	p.E278G
8210	MXRA5	25878	X	3228144	Missense	C	G	0.069	p.E2700D
8210	NBPF3	84224	1	21799922	Missense	G	A	0.432	p.E262K
8210	NFASC	23114	1	204951007	Missense	A	G	0.136	p.N777D
8210	'T5C1B-RDH1	100526794	2	18757622	Missense	G	A	0.218	p.A446V
8210	OR5T3	390154	11	56020112	Missense	C	T	0.344	p.A146V
8210	PABPC1	26986	8	101724606	Missense	G	A	0.192	p.T319I
8210	PCDHGA2	56113	5	140725208	Missense	C	G	0.32	p.D536E
8210	PCGF6	84108	10	105108528	Missense	T	C	0.167	p.R168G
8210	PDP1	54704	8	94929311	Splice_Site	C	T	0.059	.
8210	PPFIA2	8499	12	81671174	Missense	T	A	0.111	p.I1078F
8210	RASL11A	387496	13	27847182	Nonsense	C	T	0.222	p.Q94X
8210	ROBO2	6092	3	77671485	Missense	C	T	0.074	p.A628V
8210	RPS6KA6	27330	X	83442896	Missense	G	T	0.154	p.F4L
8210	RWDD2B	10069	21	30380178	Missense	G	T	0.308	p.A210E
8210	SCRN2	90507	17	45916270	Missense	C	T	0.067	p.G220E
8210	SEMA3D	223117	7	84628854	Missense	C	G	0.11	p.G746R
8210	SEMA3E	9723	7	83095913	Missense	T	A	0.3	p.E114V
8210	SEMA3G	56920	3	52475330	Missense	C	T	0.215	p.D255N
8210	SERPINE2	5270	2	224866369	Nonsense	G	C	0.219	p.Y83X
8210	SI	6476	3	164737524	Missense	C	T	0.3	p.D1097N
8210	SIRPB1	10326	20	1546911	Missense	C	G	0.088	p.A363P
8210	SLC7A14	57709	3	170244554	Missense	G	T	0.327	p.L58I
8210	SORCS1	114815	10	108427559	Missense	C	A	0.29	p.D731Y
8210	SPTAN1	6709	9	131370483	Missense	A	C	0.378	p.K1473N
8210	STXBP5	134957	6	147527111	Missense	T	G	0.136	p.V52G
8210	TCHH	7062	1	152081482	Missense	C	T	0.083	p.R1404H
8210	TINF2	26277	14	24709911	Missense	T	C	0.323	p.N259D
8210	TMEM106C	79022	12	48359115	Missense	C	T	0.067	p.R80C
8210	TMEM189	387521	20	48746208	Missense	C	T	0.382	p.R118Q
8210	TMEM260	54916	14	57101635	Missense	G	T	0.07	p.W581C
8210	TRAM1L1	133022	4	118006089	Missense	T	A	0.272	p.K154M
8210	UNC80	285175	2	210698825	Missense	G	A	0.283	p.E954K
8210	USP48	84196	1	22021633	Nonsense	G	A	0.051	p.R937X
8210	USP8	9101	15	50784955	Missense	C	A	0.098	p.N764K
8210	USP8	9101	15	50784950	Missense	C	T	0.079	p.R763W
8210	WDFY3	23001	4	85662943	Missense	T	G	0.088	p.I2069L
8210	YES1	7525	18	743097	Missense	C	A	0.227	p.G294V
8210	ZNF311	282890	6	28963727	Missense	A	T	0.273	p.L351H
8210	ZNF331	55422	19	54081147	Missense	G	A	0.235	p.G445R
8210	ZNF521	25925	18	22806918	Missense	C	A	0.214	p.V322F
8208	ADAM21	8747	14	70924335	Missense	C	T	0.1	p.P40L
8208	ADARB2	105	10	1405398	Missense	C	T	0.2	p.R301Q
8208	ADRBK2	157	22	26100145	Missense	G	A	0.136	p.V433I
8208	AEBP1	165	7	44147685	Splice_Site	T	C	0.119	c.940+2T>C
8208	ARHGAP22	58504	10	49791064	Missense	C	A	0.219	p.Q56H
8208	ARHGAP42	143872	11	100830640	Missense	T	G	0.278	p.F397V
8208	ARHGAP42	143872	11	100665883	Nonsense	G	T	0.263	p.E100X
8208	ARID1A	8289	1	27087486	Frame_Shift_Ins	C	CA	0.216	p.P687fs
8208	ARPP21	10777	3	35748548	Missense	T	A	0.169	p.S257T
8208	ATP2C2	9914	16	84449147	Missense	G	A	0.191	p.V192I
8208	BBS4	585	15	73023725	Missense	G	T	0.154	p.C264F
8208	BEND2	139105	X	18220008	Missense	A	C	0.229	p.N320K
8208	BRD8	10902	5	137503739	Missense	G	A	0.556	p.S297F
8208	BTBD19	149478	1	45275892	Missense	T	C	0.217	p.C32R
8208	C10orf126	283080	10	29137210	Missense	A	C	0.141	p.N30T
8208	C16orf78	123970	16	49433091	Missense	A	C	0.198	p.K234Q
8208	CAMSAP3	57662	19	7675643	Missense	G	A	0.3	p.D347N
8208	CCDC154	645811	16	1486514	Frame_Shift_Del	ACT	A	0.223	p.S473fs
8208	CELSR3	1951	3	48698827	Missense	G	A	0.234	p.S414L
8208	CERCAM	51148	9	131196765	Missense	T	C	0.243	p.Y470H
8208	CFTR	1080	7	117232304	Frame_Shift_Del	GA	G	0.157	p.E695fs
8208	CHD2	1106	15	93499712	Nonsense	G	A	0.143	p.W611X
8208	CHRD	8646	3	184104344	Missense	T	G	0.588	p.V666G
8208	CHST1	8534	11	45672368	Missense	C	T	0.212	p.G36R

8208	*CHSY3*	337876	5	129241112	Missense	G	A	0.317	p.R197H
8208	*CLEC14A*	161198	14	38724047	Missense	G	A	0.058	p.S394F
8208	*CMYA5*	202333	5	79031372	Missense	G	C	0.071	p.V2262L
8208	*COL22A1*	169044	8	139609182	Missense	C	T	0.18	p.R1466Q
8208	*COL9A1*	1297	6	70942475	Splice_Site	C	G	0.254	c.1586-1G>C
8208	*CORO6*	84940	17	27946777	Missense	A	C	0.175	p.D71E
8208	*CRB1*	23418	1	197404281	Missense	T	G	0.217	p.C1072W
8208	*CYFIP1*	23191	15	22956564	Missense	T	G	0.208	p.F601V
8208	*DCAF8L1*	139425	X	27998800	Missense	A	C	0.217	p.L218V
8208	*DPEP3*	64180	16	68012250	Missense	G	T	0.24	p.D227E
8208	*DTNA*	1837	18	32374129	Missense	A	G	0.138	p.N93D
8208	*DZANK1*	55184	20	18446022	Splice_Site	C	A	0.182	.
8208	*EHMT1*	79813	9	140611226	Missense	G	C	0.176	p.R78S
8208	*ENAM*	10117	4	71509199	Missense	T	A	0.309	p.Y686N
8208	*FAM189A2*	9413	9	71986497	Missense	T	G	0.132	p.F32C
8208	*FAM83H*	286077	8	144808396	Missense	C	A	0.165	p.V1079F
8208	*FDFT1*	2222	8	11695964	Missense	G	A	0.25	p.R367Q
8208	*FER1L6-AS2*	157376	8	125072943	Missense	T	C	0.182	p.F1047S
8208	*FUCA2*	2519	6	143828529	Missense	T	A	0.25	p.Y86F
8208	*GHITM*	27069	10	85904630	Splice_Site	G	A	0.156	c.342-1G>A
8208	*GPAT2*	150763	2	96688961	Missense	G	A	0.047	p.S681L
8208	*GPIHBP1*	338328	8	144295183	Missense	G	T	0.089	p.C14F
8208	*GRIK4*	2900	11	120690497	Missense	G	T	0.158	p.V127F
8208	*HHATL*	57467	3	42741254	Missense	T	G	0.237	p.K57T
8208	*HMP19*	51617	5	173491322	Splice_Site	A	C	0.195	c.213+4A>C
8208	*HPS1*	3257	10	100193820	Missense	C	T	0.19	p.V143I
8208	*HSPG2*	3339	1	22199882	Missense	C	G	0.281	p.G1260A
8208	*HSPG2*	3339	1	22191440	Missense	C	T	0.183	p.A1508T
8208	*ING1*	3621	13	111372190	Missense	T	C	0.132	p.W251R
8208	*KCNK10*	54207	14	88729614	Missense	A	T	0.09	p.L112M
8208	*KIF7*	374654	15	90190110	Missense	G	A	0.206	p.P580L
8208	*KIF7*	374654	15	90176073	Missense	C	A	0.103	p.S958I
8208	*KMT2D*	8085	12	49435753	Frame_Shift_Del	CTTGACCA	G	0.068	p.D2037fs
8208	*LAMA1*	284217	18	6977814	Missense	T	G	0.44	p.K2086T
8208	*LMNA*	4000	1	156105716	Nonsense	C	T	0.103	p.R321X
8208	*LRIT1*	26103	10	85993997	Missense	A	C	0.27	p.F243V
8208	*LRRIQ3*	127255	1	74507126	Missense	C	A	0.206	p.A497S
8208	*MGAT4C*	25834	12	86383228	Missense	C	A	0.121	p.V33F
8208	*MUC2*	4583	11	1093286	Missense	C	G	0.091	p.T1702S
8208	*MYO18A*	399687	17	27448585	Missense	G	A	0.111	p.P451S
8208	*MYO7B*	4648	2	128338363	Missense	C	T	0.228	p.T349M
8208	*MYT1*	4661	20	62839425	Missense	G	T	0.226	p.E292D
8208	*NPHP4*	261734	1	5964826	Missense	G	C	0.092	p.T665S
8208	*OR11L1*	391189	1	248004607	Missense	T	C	0.22	p.T198A
8208	*PCDH17*	27253	13	58207755	Missense	G	A	0.164	p.G359R
8208	*PDCD11*	22984	10	105184755	Missense	C	A	0.257	p.S926R
8208	*PIEZO2*	63895	18	10752785	Missense	C	G	0.157	p.S1314T
8208	*PKHD1L1*	93035	8	110476928	Missense	G	A	0.226	p.G2623R
8208	*PLG*	5340	6	161139492	Splice_Site	A	C	0.198	c.950+4A>C
8208	*POLR3B*	55703	12	106820987	Missense	C	T	0.353	p.L372F
8208	*POP1*	10940	8	99170291	Missense	C	T	0.143	p.T956M
8208	*POTEG*	404785	14	19553556	Missense	G	A	0.25	p.G47E
8208	*POU3F3*	5455	2	105473140	Missense	C	T	0.159	p.T391I
8208	*PPEF2*	5470	4	76794377	Missense	T	G	0.247	p.D470A
8208	*PRKRA*	8575	2	179309187	Missense	G	T	0.151	p.Q120K
8208	*PRRC2A*	7916	6	31605084	Missense	G	A	0.225	p.V2106I
8208	*RADIL*	55698	7	4917614	Missense	C	T	0.182	p.A53T
8208	*RAPGEF1*	2889	9	134505657	Missense	C	T	0.277	p.V237M
8208	*RINL*	126432	19	39367111	Missense	T	C	0.063	p.N24D
8208	*RNU6-81P*	100873780	2	132237750	Missense	G	A	0.07	p.G162S
8208	*SAFB2*	9667	19	5587279	Missense	T	G	0.14	p.Y946S
8208	*SEMA3B*	7869	3	50312998	Missense	C	T	0.216	p.R210W
8208	*SERPINB3*	6317	18	61323148	Missense	C	T	0.131	p.D306N
8208	*SPTA1*	6708	1	158585162	Missense	C	T	0.206	p.R2211H
8208	*ST6GAL2*	84620	2	107460182	Missense	G	T	0.225	p.H84Q
8208	*STAT3*	6774	17	40481640	Missense	T	A	0.379	p.T389S
8208	*SVEP1*	79987	9	113170505	Missense	G	T	0.24	p.L2459I
8208	*TAAR2*	9287	6	132938609	Missense	A	C	0.151	p.L246V
8208	*TBC1D26*	353149	17	15641610	Missense	A	G	0.086	p.Y99C
8208	*TECTA*	7007	11	121037431	Missense	A	G	0.253	p.E1843G
8208	*TOR1AIP2*	163590	1	179815786	Missense	C	T	0.286	p.R278Q
8208	*TTN*	7273	2	179634421	Missense	T	G	0.273	p.T2963P
8208	*UCN3*	114131	10	5415778	Missense	T	A	0.172	p.I32N
8208	*UGT2A1*	10941	4	70504805	Missense	G	T	0.153	p.A386E
8208	*YAP1*	10413	11	102100625	Missense	T	G	0.187	p.L494W
8208	*ZER1*	10444	9	131504925	Missense	T	C	0.241	p.N487D
8208	*ZER1*	10444	9	131504936	Missense	A	C	0.205	p.L483R
8208	*ZFP82*	284406	19	36884113	Missense	G	A	0.066	p.R377C
8208	*ZNF462*	58499	9	109688513	Missense	T	G	0.178	p.F774V
8208	*ZNF880*	400713	19	52888359	Missense	G	A	0.146	p.R509Q

8199	*ANKRD30B*	374860	18	14748484	Missense	C	A	0.246	p.S22R
8199	*ARID1A*	8289	1	27099361	Nonsense	C	T	0.151	p.Q1200X
8199	*ATP11AUN*	400165	13	113333878	Missense	A	C	0.25	p.D62A
8199	*ATRX*	546	X	76953110	Missense	G	A	0.63	p.S68L
8199	*BNIP2*	663	15	59981515	Missense	A	G	0.077	p.V42A
8199	*CELSR2*	1952	1	109811510	Missense	G	A	0.064	p.V2171I
8199	*CGNL1*	84952	15	57731056	Missense	G	A	0.141	p.V287I
8199	*CNOT3*	4849	19	54646728	Missense	G	A	0.075	p.R5H
8199	*CSMD1*	64478	8	2944708	Missense	C	A	0.196	p.G2462V
8199	*DROSHA*	29102	5	31515657	Missense	G	A	0.158	p.S321L
8199	*EVI5*	7813	1	93163526	Missense	G	T	0.222	p.A263D
8199	*FN1*	2335	2	216279651	Missense	G	A	0.118	p.T617I
8199	*FSIP2*	401024	2	186671920	Missense	A	T	0.103	p.N6052Y
8199	*GAL3ST2*	64090	2	242716380	Missense	A	T	0.07	p.M4L
8199	*GALNT14*	79623	2	31168729	Missense	G	A	0.214	p.T201M
8199	*GKAP1*	80318	9	86383855	Missense	C	A	0.105	p.D206Y
8199	*GPRC5C*	55890	17	72428181	Missense	C	T	0.064	p.R2W
8199	*GUCY1B3*	2983	4	156696170	Missense	A	T	0.208	p.Y23F
8199	*HELZ2*	85441	20	62194212	Missense	C	T	0.152	p.R1988H
8199	*HS3ST6*	64711	16	1961674	Missense	G	C	0.136	p.L316V
8199	*IL31RA*	133396	5	55206444	Missense	G	A	0.097	p.S510N
8199	*KNOP1*	400506	16	19725985	Missense	G	A	0.212	p.H125Y
8199	*LAMA2*	3908	6	129591877	Missense	C	T	0.16	p.L811F
8199	*LEF1*	51176	4	108969874	Missense	G	T	0.082	p.A372D
8199	*LOC401052*	401052	3	10049098	Missense	T	G	1	p.H96P
8199	*MYBPC2*	4606	19	50957397	Missense	G	A	0.061	p.V624I
8199	*MYLK3*	91807	16	46773999	Missense	C	A	0.167	p.V180L
8199	*NAE1*	8883	16	66839931	Splice_Site	T	A	0.167	c.1331-2A>T
8199	*NBPF8*	728841	1	148025797	Missense	T	A	0.294	p.K352M
8199	*OR51A2*	401667	11	4976013	Missense	A	G	0.267	p.W311R
8199	*OR51A2*	401667	11	4976768	Missense	C	T	0.176	p.G59E
8199	*OR51B5*	282763	11	5462374	Missense	C	T	0.07	p.R124H
8199	*POT1*	25913	7	124511011	Missense	G	A	0.286	p.P70L
8199	*PREX2*	80243	8	68965472	Missense	C	T	0.116	p.R362W
8199	*PTPN13*	5783	4	87671727	Nonsense	A	T	0.175	p.K919X
8199	*REPS2*	9185	X	17153504	Missense	A	G	0.522	p.M595V
8199	*SCARA3*	51435	8	27516598	Missense	T	C	0.183	p.V304A
8199	*SERTAD1*	29950	19	40929363	Missense	T	C	0.071	p.T31A
8199	*SHANK2*	22941	11	70331960	Missense	G	T	0.073	p.P892T
8199	*SLC2A5*	6518	1	9101924	Missense	A	G	0.179	p.V164A
8199	*SLC46A3*	283537	13	29287062	Missense	A	G	0.094	p.F272S
8199	*SYNJ1*	8867	21	34059327	Frame_Shift_Ins	T	TCC	0.2	p.G342fs
8199	*TDRD1*	56165	10	115986855	Missense	C	G	0.188	p.S1067C
8199	*THOC5*	8563	22	29913074	Missense	G	A	0.125	p.A542V
8199	*TMX4*	56255	20	7980441	Missense	G	C	0.135	p.I135M
8199	*UGT2A1*	10941	4	70513350	Missense	G	A	0.167	p.L5F
8199	*UNC13C*	440279	15	54307147	Missense	C	T	0.138	p.L683F
8199	*ZNF133*	7692	20	18287008	Missense	A	G	0.143	p.E63G
8198	*AARS*	16	16	70301677	Missense	G	C	0.065	p.D369E
8198	*ACIN1*	22985	14	23549285	Missense	G	A	0.061	p.S478F
8198	*AKAP13*	11214	15	86122654	Missense	T	C	0.082	p.M452T
8198	*AKAP13*	11214	15	86123988	Missense	G	A	0.07	p.V897M
8198	*ARHGEF15*	22899	17	8216468	Missense	T	C	0.16	p.L277P
8198	*ASGR2*	433	17	7010631	Missense	C	T	0.073	p.D120N
8198	*BCAS1*	8537	20	52645166	Missense	A	G	0.079	p.V163A
8198	*BCLAF1*	9774	6	136590722	Missense	G	A	0.167	p.A691V
8198	*BRINP2*	57795	1	177247854	Missense	C	G	0.091	p.L390V
8198	*C17orf58*	284018	17	65988049	Missense	T	C	0.095	p.I92V
8198	*C6orf136*	221545	6	30619209	Missense	C	T	0.094	p.R425W
8198	*CCDC182*	101927581	17	55822467	Missense	G	A	0.1	p.A56V
8198	*CEP95*	90799	17	62523253	Missense	C	T	0.25	p.R393W
8198	*CFB*	629	6	31914180	Missense	G	A	0.094	p.R32Q
8198	*CHID1*	66005	11	870446	Missense	G	A	0.286	p.A363V
8198	*CLDN17*	26285	21	31538692	Missense	C	T	0.07	p.A82T
8198	*CNPPD1*	27013	2	220037666	Missense	A	G	0.138	p.L292P
8198	*CR1L*	1379	1	207851611	Missense	A	G	0.073	p.R116G
8198	*CUBN*	8029	10	17147521	Missense	G	T	0.108	p.P389T
8198	*DESI1*	27351	22	41997181	Missense	C	T	0.182	p.A143T
8198	*DNHD1*	144132	11	6579106	Missense	C	A	0.108	p.H2861N
8198	*EMILIN3*	90187	20	39992391	Missense	G	A	0.125	p.T134M
8198	*ERV3-1*	2086	7	64452830	Missense	C	T	0.065	p.C192Y
8198	*FREM1*	158326	9	14801769	Missense	C	T	0.1	p.R1192H
8198	*FSIP1*	161835	15	39910052	Missense	C	G	0.071	p.G528A
8198	*GMNN*	51053	6	24780892	Missense	A	C	0.083	p.N18T
8198	*GNRH1*	2796	8	25280756	Missense	G	A	0.111	p.R31C
8198	*HIVEP1*	3096	6	12123249	Missense	A	G	0.056	p.N1074S
8198	*HKDC1*	80201	10	71026510	Missense	C	A	0.064	p.N917K
8198	*HPS4*	89781	22	26854441	Missense	G	A	0.06	p.H606Y
8198	*IGFN1*	91156	1	201181217	Missense	A	G	0.121	p.D2399G
8198	*ITLN1*	55600	1	160851826	Missense	A	T	0.111	p.V109D

8198	*KIAA0040*	9674	1	175129955	Missense	G	C	0.073	p.N65K
8198	*KXD1*	79036	19	18679379	Missense	C	G	0.114	p.P157A
8198	*LCORL*	254251	4	17885365	Missense	T	C	0.143	p.K596R
8198	*.OC10192946*	101929468	4	105412464	In_Frame_Del	TGCC	T	0.12	p.165_165del
8198	*LRRC8E*	80131	19	7963976	Missense	T	C	0.103	p.M190T
8198	*MED14*	9282	X	40541897	Nonsense	G	A	0.1	p.R775X
8198	*MFSD8*	256471	4	128842876	Missense	C	G	0.067	p.G385R
8198	*MKNK1-AS1*	100507423	1	47028368	Missense	C	T	0.2	p.G306R
8198	*MROH9*	80133	1	170928671	Missense	T	C	0.12	p.V74A
8198	*MXRA5*	25878	X	3240343	Missense	G	A	0.111	p.A1128V
8198	*MYH15*	22989	3	108159977	Missense	G	A	0.065	p.T949I
8198	*MYH6*	4624	14	23861811	Missense	A	G	0.15	p.V1101A
8198	*MYH6*	4624	14	23854194	Missense	C	A	0.107	p.Q1740H
8198	*MYOM3*	127294	1	24406535	Missense	G	A	0.1	p.P853S
8198	*NDUFA6*	4700	22	42486723	Missense	G	A	0.061	p.A35V
8198	*OR2AE1*	81392	7	99474427	Missense	A	G	0.068	p.I77T
8198	*OR52L1*	338751	11	6007899	Missense	C	T	0.098	p.D88N
8198	*OR52N1*	79473	11	5809811	Missense	G	T	0.057	p.T79N
8198	*P2RX4*	5025	12	121666646	Missense	A	G	0.062	p.S258G
8198	*PCDHGA8*	9708	5	140772941	Missense	T	G	0.073	p.N187K
8198	*PCSK1*	5122	5	95728974	Missense	G	C	0.083	p.Q618E
8198	*POLQ*	10721	3	121208176	Missense	T	C	0.064	p.H1201R
8198	*R3HDM1*	23518	2	136393658	Missense	A	G	0.068	p.M270V
8198	*REST*	5978	4	57796900	Missense	G	A	0.077	p.V626I
8198	*S100A7A*	338324	1	153391729	Missense	G	A	0.075	p.A84T
8198	*SNX18*	112574	5	53815495	Missense	A	C	0.063	p.E571D
8198	*SP1*	6667	12	53776124	In_Frame_Del	AATGGCAC	C	0.038	p.132_134del
8198	*SPOCK1*	6695	5	136834078	Missense	C	A	0.179	p.W57L
8198	*TFCP2L1*	29842	2	122038783	Missense	G	A	0.107	p.R43C
8198	*TFR2*	7036	7	100230864	Missense	G	C	0.097	p.I238M
8198	*TMEM104*	54868	17	72784932	Missense	G	A	0.086	p.V58M
8198	*TNN*	63923	1	175092674	Missense	C	T	0.1	p.P930L
8198	*ZMAT3*	64393	3	178748788	Splice_Site	C	A	0.182	c.271-1G>T
8198	*ZNF326*	284695	1	90473260	Missense	A	G	0.053	p.N189S
8194	*ACKR2*	1238	3	42907112	Missense	A	C	0.102	p.Y373S
8194	*AHNAK2*	113146	14	105420215	Missense	T	C	0.111	p.T525A
8194	*ANKRD11*	29123	16	89350300	Missense	C	T	0.397	p.D884N
8194	*ANO2*	57101	12	6030405	Missense	A	G	0.071	p.V108A
8194	*AOAH*	313	7	36763672	Missense	C	T	0.062	p.D28N
8194	*AP3B1*	8546	5	77406125	Nonsense	G	T	0.133	p.S768X
8194	*ASCL3*	56676	11	8959545	Missense	C	A	0.094	p.R55L
8194	*ATXN3*	4287	14	92548719	Missense	C	T	0.06	p.A234T
8194	*BPIFB3*	359710	20	31656632	Missense	C	G	0.071	p.H334Q
8194	*C15orf54*	400360	15	39544367	Missense	G	A	0.452	p.G11S
8194	*C17orf58*	284018	17	65989064	Missense	A	G	0.419	p.F67L
8194	*C19orf53*	28974	19	13885484	Missense	A	G	0.087	p.K39R
8194	*C1orf131*	128061	1	231374715	Missense	G	C	0.083	p.A113G
8194	*CACNA1S*	779	1	201017805	Missense	A	C	0.238	p.V1449G
8194	*CASZ1*	54897	1	10711011	Missense	C	T	0.412	p.V935M
8194	*CCDC102B*	79839	18	66504349	Missense	A	T	0.321	p.R117W
8194	*CCDC171*	203238	9	15920397	Missense	C	T	0.333	p.R1244C
8194	*CD8B*	926	2	87042751	Missense	G	A	0.068	p.T195I
8194	*CD93*	22918	20	23065209	Missense	G	A	0.083	p.P541S
8194	*CDH17*	1015	8	95143172	Missense	T	G	0.062	p.E739A
8194	*CFB*	629	6	31916708	Missense	A	C	0.385	p.T380P
8194	*CIZ1*	25792	9	130950141	Splice_Site	C	T	0.222	c.358+1G>A
8194	*CMTR2*	55783	16	71318577	Missense	T	C	0.066	p.N416S
8194	*COL17A1*	1308	10	105824333	Missense	G	A	0.069	p.T210M
8194	*CSMD3*	114788	8	113318391	Missense	G	C	0.226	p.T2599R
8194	*DHX8*	1659	17	41570872	Missense	A	G	0.391	p.N308S
8194	*DKK2*	27123	4	107845794	Missense	C	T	0.05	p.R146Q
8194	*DMXL2*	23312	15	51791559	Missense	A	G	0.058	p.S1288P
8194	*DPF3*	8110	14	73137942	Missense	G	A	0.054	p.H336Y
8194	*EHBP1*	23301	2	62934373	Missense	C	T	0.136	p.S16F
8194	*FAM129A*	116496	1	184868364	Missense	C	T	0.3	p.R45H
8194	*FAM65C*	140876	20	49225260	Missense	C	T	0.382	p.G230S
8194	*FGFR2*	2263	10	123278280	Missense	C	T	0.34	p.A335T
8194	*FMO3*	2328	1	171083242	Missense	A	G	0.069	p.E308G
8194	*FREM3*	166752	4	144617606	Missense	T	A	0.205	p.N1408I
8194	*GCOM1*	145781	15	58001174	Missense	A	C	0.182	p.T523P
8194	*GOLGA1*	2800	9	127685506	Splice_Site	T	C	0.077	c.433-4A>G
8194	*GPR137C*	283554	14	53098902	Missense	G	A	0.078	p.V248I
8194	*GPR150*	285601	5	94956626	Missense	G	A	0.286	p.R216H
8194	*GRIN2A*	2903	16	9857958	Missense	G	A	0.068	p.P1148L
8194	*HELB*	92797	12	66725160	Missense	C	T	0.068	p.P966L
8194	*HLA-A*	3105	6	29910693	Missense	A	G	0.154	p.Q78R
8194	*HLA-DQB2*	3120	6	32725563	Missense	G	T	0.143	p.H248Q
8194	*HNRNPUL1*	11100	19	41807592	Missense	C	T	0.2	p.A457V
8194	*HPS1*	3257	10	100179851	Missense	T	C	0.075	p.Q603R
8194	*HS6ST3*	266722	13	96743624	Missense	C	T	0.355	p.R170W

8194	*IFNAR1*	3454	21	34715699	Missense	G	C	0.083	p.V168L
8194	*IKBKE*	9641	1	206665052	Missense	C	T	0.079	p.A602V
8194	*INADL*	10207	1	62516683	Missense	G	C	0.077	p.V1360L
8194	*IQCC*	55721	1	32671899	Splice_Site	G	T	0.471	c.186+1G>T
8194	*ITGB2*	3689	21	46313371	Missense	G	A	0.154	p.T391M
8194	*KANK1*	23189	9	740903	Missense	G	A	0.556	p.G1222D
8194	*KIAA0391*	9692	14	35739609	Missense	C	T	0.222	p.S476L
8194	*KIAA0586*	9786	14	58949289	Missense	C	A	0.353	p.N1054K
8194	*KIAA1211L*	343990	2	99439046	Missense	C	A	0.286	p.G564C
8194	*KIAA1614*	57710	1	180905448	Missense	G	T	0.088	p.L801F
8194	*KIAA1755*	85449	20	36869159	Missense	G	T	0.273	p.S458R
8194	*KIF20B*	9585	10	91497912	Missense	A	G	0.068	p.K1065R
8194	*KRT18*	3875	12	53346568	Missense	G	A	0.222	p.R412Q
8194	*KRT8*	3856	12	53298675	Missense	A	C	0.25	p.S31A
8194	*LDHD*	197257	16	75148761	Missense	C	T	0.07	p.A145T
8194	*LHCGR*	3973	2	48915492	Missense	G	A	0.424	p.H482Y
8194	*LRIF1*	55791	1	111493914	Missense	G	C	0.179	p.P531R
8194	*MAVS*	57506	20	3838441	Missense	C	G	0.071	p.Q93E
8194	*MEP1B*	4225	18	29793211	Missense	C	T	0.286	p.S423L
8194	*MPV17L*	255027	16	15489800	Missense	C	T	0.333	p.P16S
8194	*MUC16*	94025	19	9083644	Missense	G	A	0.4	p.P2724L
8194	*MUC21*	394263	6	30955179	Missense	G	C	0.097	p.E409D
8194	*NBAS*	51594	2	15674686	Missense	T	C	0.06	p.I243V
8194	*NCKAP5*	344148	2	133538745	Missense	C	A	0.103	p.R1643S
8194	*NEK10*	152110	3	27346385	Missense	G	A	0.133	p.P294L
8194	*NGF*	4803	1	115829313	Missense	G	A	0.049	p.A35V
8194	*NLRP8*	126205	19	56499279	Nonstop	G	C	0.075	p.X1049Y
8194	*NOC3L*	64318	10	96116960	Missense	C	G	0.35	p.G160A
8194	*NPC1*	4864	18	21120444	Missense	T	C	0.064	p.I858V
8194	*OBSL1*	23363	2	220416942	Splice_Site	G	C	0.143	c.5309-4C>G
8194	*OR10AD1*	121275	12	48597053	Missense	A	G	0.107	p.V8A
8194	*OR2J2*	26707	6	29141849	Missense	T	C	0.046	p.V146A
8194	*OR5T3*	390154	11	56020681	Missense	A	G	0.081	p.K336E
8194	*OR7G3*	390883	19	9237482	Missense	C	T	0.389	p.V49I
8194	*P2RY4*	5030	X	69478942	Missense	T	G	0.182	p.N178T
8194	*PARK2*	5071	6	162394338	Missense	C	T	0.25	p.V244I
8194	*PCDH7*	5099	4	30725394	Missense	G	T	0.089	p.V784L
8194	*PCDHB2*	56133	5	140475658	Missense	C	G	0.097	p.F428L
8194	*PCDHGA9*	56107	5	140784974	Frame_Shift_Ins	C	CT	0.378	p.L819fs
8194	*PINK1-AS*	100861548	1	20972111	Missense	G	A	0.143	p.A340T
8194	*PLA2G4B*	100137049	15	42134097	Missense	C	T	0.231	p.R422C
8194	*PODXL*	5420	7	131195712	Missense	G	A	0.093	p.S194L
8194	*POTEB3*	102724631	15	21051218	Missense	C	A	0.429	p.K414N
8194	*POU6F2*	11281	7	39125590	Missense	T	C	0.083	p.V50A
8194	*PRDM2*	7799	1	14105139	Missense	T	A	0.076	p.D283E
8194	*PSMB9*	5698	6	32825090	Missense	G	A	0.065	p.R60H
8194	*RAG1*	5896	11	36595600	Missense	A	G	0.063	p.H249R
8194	*RBL2*	5934	16	53514529	Missense	C	T	0.222	p.P978S
8194	*RCAN3*	11123	1	24861704	Missense	G	A	0.051	p.R164Q
8194	*RNU6-81P*	100873780	2	132237927	Missense	C	A	0.333	p.R221S
8194	*SATB2*	23314	2	200188627	Missense	T	C	0.077	p.I481V
8194	*SCN3A*	6328	2	165971986	Missense	G	A	0.3	p.P1165S
8194	*SKIDA1*	387640	10	21805652	Missense	C	T	0.4	p.R367Q
8194	*SKOR1*	390598	15	68117300	Missense	T	A	0.115	p.W200R
8194	*SLC12A2*	6558	5	127486939	Missense	G	A	0.077	p.R705H
8194	*SLC25A32*	81034	8	104427541	Missense	C	T	0.067	p.P108L
8194	*SLC25A39*	51629	17	42398518	Missense	C	T	0.25	p.G192D
8194	*SLC26A6*	65010	3	48670827	Splice_Site	G	T	0.073	c.183-4C>A
8194	*SLCO2B1*	11309	11	74883577	Missense	G	A	0.064	p.R312Q
8194	*SWAP70*	23075	11	9769562	Missense	C	G	0.082	p.Q505E
8194	*SYNGAP1*	8831	6	33405878	Missense	C	T	0.4	p.A399V
8194	*TAF1C*	9013	16	84212897	Missense	T	A	0.442	p.T660S
8194	*TET2*	54790	4	106156909	Nonsense	C	T	0.718	p.Q604X
8194	*TLR1*	7096	4	38799710	Missense	T	C	0.08	p.N248S
8194	*'MX2-CTNND.*	100528016	11	57512373	Missense	T	C	0.061	p.M458V
8194	*TNFRSF8*	943	1	12186058	Missense	A	G	0.083	p.S402G
8194	*TRIO*	7204	5	14399078	Nonsense	C	T	0.27	p.R1505X
8194	*TTYH2*	94015	17	72248408	Missense	C	A	0.119	p.D384E
8194	*UBP1*	7342	3	33458266	Missense	T	C	0.075	p.N109S
8194	*UBQLN3*	50613	11	5529920	Missense	G	C	0.094	p.T290S
8194	*USP24*	23358	1	55559689	Missense	A	T	0.289	p.L2092Q
8194	*VN1R1*	57191	19	57967133	Missense	G	A	0.074	p.S241F
8194	*WDR73*	84942	15	85186876	In_Frame_Del	GCTCCGTG'	A	0.119	p.315_321del
8194	*ZC3H18*	124245	16	88695232	Missense	G	C	0.333	p.A870P
8194	*ZGRF1*	55345	4	113540718	Missense	C	G	0.073	p.L160F
8194	*ZNF230*	7773	19	44515514	Missense	C	A	0.103	p.D441E
8194	*ZNF230*	7773	19	44515074	Missense	C	A	0.061	p.L295I
8194	*ZNF264*	9422	19	57723013	Missense	G	A	0.061	p.R183H
8194	*ZNF264*	9422	19	57723007	Missense	G	C	0.06	p.R181T
8194	*ZNF345*	25850	19	37368669	Missense	C	T	0.727	p.P313S

8194	*ZNF419*	79744	19	58004346	Missense	G	C	0.1	p.E141Q
8194	*ZSCAN5B*	342933	19	56704404	Frame_Shift_Del	TG	T	0.14	p.T6fs
8194	*ZSCAN5B*	342933	19	56704400	Frame_Shift_Ins	A	AG	0.128	p.S8fs
8194	*ZSWIM3*	140831	20	44505973	Missense	T	C	0.075	p.V259A
8191	*ABCA6*	23460	17	67106942	Missense	C	A	0.148	p.D758Y
8191	*ABCC9*	10060	12	22025607	Missense	G	T	0.209	p.A717D
8191	*ADAM21*	8747	14	70924602	Missense	T	G	0.131	p.F129C
8191	*ADAM21*	8747	14	70924613	Nonsense	C	T	0.081	p.R133X
8191	*AIM1L*	55057	1	26671248	Missense	A	G	0.071	p.V634A
8191	*ALPPL2*	251	2	233273207	Splice_Site	G	A	0.078	c.784-4G>A
8191	*ANKRD12*	23253	18	9255982	Missense	A	G	0.135	p.K906R
8191	*ARMC4*	55130	10	28196642	Missense	C	T	0.263	p.V854M
8191	*ARMC4*	55130	10	28250610	Missense	C	A	0.1	p.D425Y
8191	*ASPSCR1*	79058	17	79973185	Missense	G	A	0.127	p.D530N
8191	*ATP8B1*	5205	18	55373719	Splice_Site	C	T	0.194	c.279+3G>A
8191	*ATR*	545	3	142281612	Missense	A	G	0.111	p.M211T
8191	*BCHE*	590	3	165547699	Missense	T	C	0.125	p.R375G
8191	*C6orf118*	168090	6	165715729	Nonsense	T	A	0.278	p.K28X
8191	*CA8*	767	8	61135288	Missense	C	T	0.222	p.G220S
8191	*CADPS2*	93664	7	122078454	Missense	C	A	0.146	p.C806F
8191	*CAP2*	10486	6	17507534	Missense	G	T	0.15	p.W145C
8191	*CASD1*	64921	7	94174854	Splice_Site	T	C	0.079	c.1477-3T>C
8191	*CEP164*	22897	11	117267884	Missense	A	G	0.094	p.Q1119R
8191	*CHL1*	10752	3	384664	Splice_Site	C	T	0.273	c.680-3C>T
8191	*CTRC*	11330	1	15771229	Missense	G	A	0.269	p.V208I
8191	*CXXC5*	51523	5	139060788	Missense	G	C	0.213	p.G227A
8191	*DDX55*	57696	12	124104551	Missense	A	G	0.075	p.N556S
8191	*DENND2A*	27147	7	140273781	Nonsense	G	A	0.163	p.R425X
8191	*DMBT1*	1755	10	124330427	Missense	C	T	0.036	p.S54L
8191	*DMD*	1756	X	32632562	Missense	C	T	0.455	p.R324K
8191	*DOT1L*	84444	19	2226772	Missense	G	C	0.075	p.V1418L
8191	*DSCAM*	1826	21	42080436	Missense	C	T	0.153	p.C102Y
8191	*ETAA1*	54465	2	67631831	Missense	G	A	0.034	p.E673K
8191	*F5*	2153	1	169519112	Missense	C	T	0.125	p.R513K
8191	*F5*	2153	1	169510139	Missense	G	A	0.036	p.L1397F
8191	*FAM221B*	392307	9	35826038	Missense	T	C	0.054	p.K41E
8191	*FAM221B*	392307	9	35826058	Missense	T	C	0.051	p.H34R
8191	*FRMD4B*	23150	3	69299233	Missense	C	G	0.061	p.E173D
8191	*GAREM*	64762	18	29847959	Missense	C	T	0.172	p.E835K
8191	*GJB7*	375519	6	87994015	Missense	A	G	0.067	p.F206L
8191	*GLCE*	26035	15	69548518	Missense	G	A	0.036	p.E125K
8191	*GLYATL3*	389396	6	49485266	Missense	C	A	0.071	p.Q104K
8191	*GPC3*	2719	X	132833929	Missense	C	T	0.182	p.R410Q
8191	*GPR158*	57512	10	25888180	Missense	A	G	0.07	p.I1209V
8191	*HDAC5*	10014	17	42164885	Missense	G	C	0.067	p.D593E
8191	*HEATR1*	55127	1	236719193	Missense	A	G	0.048	p.V1854A
8191	*IBSP*	3381	4	88732746	Missense	A	G	0.055	p.D213G
8191	*IBSP*	3381	4	88732763	Missense	A	G	0.045	p.R219G
8191	*IDUA*	3425	4	982884	Missense	C	T	0.068	p.D615N
8191	*IGFN1*	91156	1	201177278	Missense	C	T	0.046	p.A1086V
8191	*IL16*	3603	15	81598438	Missense	C	T	0.035	p.P1204S
8191	*ITGA1*	3672	5	52096889	Missense	C	A	0.043	p.L221M
8191	*ITGA8*	8516	10	15634287	Missense	C	T	0.125	p.R743Q
8191	*ITIH3*	3699	3	52833448	Missense	A	G	0.246	p.N284D
8191	*KAZN*	23254	1	14925496	Missense	G	T	0.208	p.M1I
8191	*KCNG1*	3755	20	49626512	Missense	C	G	0.279	p.G122R
8191	*KIF20B*	9585	10	91497912	Missense	A	G	0.13	p.K1065R
8191	*KLHDC7B*	113730	22	50988180	Missense	C	T	0.069	p.P529S
8191	*KLHL3*	26249	5	137013251	Missense	C	T	0.25	p.V207I
8191	*KREMEN2*	79412	16	3017106	Missense	C	T	0.167	p.S279L
8191	*KRTAP9-8*	83901	17	39394482	Missense	T	C	0.5	p.I60T
8191	*LACTB*	114294	15	63414129	Missense	C	T	0.241	p.S20L
8191	*LIF*	3976	22	30639942	Missense	C	T	0.226	p.V103M
8191	*LRP2*	4036	2	170099970	Missense	A	G	0.283	p.S1165P
8191	*LRRTM4*	80059	2	77746099	Missense	G	A	0.192	p.A299V
8191	*LYPD3*	27076	19	43965712	Nonsense	G	A	0.169	p.Q278X
8191	*MARK3*	4140	14	103969233	Missense	C	A	0.263	p.A644D
8191	*MAST3*	23031	19	18255378	Missense	G	A	0.267	p.G867E
8191	*MCM7*	4176	7	99697288	Missense	C	T	0.036	p.C67Y
8191	*MKRN1*	23608	7	140171778	Missense	G	C	0.209	p.D9E
8191	*MMP25*	64386	16	3100259	Missense	C	T	0.313	p.R125C
8191	*MYO3A*	53904	10	26463130	Missense	C	A	0.055	p.R1313S
8191	*MYO9A*	4649	15	72324901	Missense	T	G	0.222	p.N290T
8191	*NCKAP5*	344148	2	133542574	Missense	C	T	0.055	p.V604M
8191	*NHSL1*	57224	6	138794475	Missense	A	G	0.239	p.Y151H
8191	*NRIP1*	8204	21	16337855	Missense	G	A	0.188	p.P887S
8191	*NSD1*	64324	5	176637576	Missense	T	C	0.056	p.S457P
8191	*NUAK2*	81788	1	205272846	Nonsense	G	C	0.143	p.S540X
8191	*OBSCN*	84033	1	228468476	Missense	C	T	0.222	p.R3155W
8191	*OR14C36*	127066	1	248512767	Missense	G	T	0.047	p.D231Y

8191	*OR14C36*	127066	1	248512749	Missense	G	A	0.041	p.G225R
8191	*OR2A2*	442361	7	143806941	Missense	G	T	0.228	p.R89I
8191	*OR2AE1*	81392	7	99474427	Missense	A	G	0.036	p.I77T
8191	*OR4E2*	26686	14	22133648	Missense	G	A	0.041	p.V118M
8191	*OR4L1*	122742	14	20528633	Missense	T	C	0.155	p.F144L
8191	*OR4X1*	390113	11	48286256	Missense	C	T	0.054	p.P282S
8191	*OR5H1*	26341	3	97851590	Missense	T	C	0.5	p.F17L
8191	*OR6Y1*	391112	1	158516998	Missense	G	T	0.188	p.H300N
8191	*OTOP3*	347741	17	72937605	Missense	G	A	0.073	p.R64Q
8191	*PAK2*	5062	3	196509577	Missense	C	G	0.111	p.S20R
8191	*PCNXL4*	64430	14	60591887	Missense	G	A	0.067	p.G766S
8191	*PDZD2*	23037	5	32087374	Missense	A	G	0.043	p.T1274A
8191	*PRKCZ*	5590	1	2100985	Missense	G	A	0.067	p.G335S
8191	*PSMD6*	9861	3	64009508	Missense	T	C	0.071	p.N2D
8191	*RAD9B*	144715	12	110960196	Missense	G	A	0.061	p.G211R
8191	*RAPGEF3*	10411	12	48134723	Nonsense	C	T	0.288	p.W633X
8191	*RETSAT*	54884	2	85570849	Missense	C	T	0.167	p.G536R
8191	*RSPH3*	83861	6	159407446	Missense	C	T	0.063	p.R213Q
8191	*RTP5*	285093	2	242814360	Missense	T	C	0.062	p.I218T
8191	*SELE*	6401	1	169696946	Missense	G	A	0.05	p.H468Y
8191	*SEMA6D*	80031	15	48058071	Missense	G	A	0.061	p.S478N
8191	*SETD2*	29072	3	47158225	Nonsense	G	A	0.15	p.R1492X
8191	*SH3TC1*	54436	4	8218729	Missense	C	T	0.22	p.T225M
8191	*SLCO1C1*	53919	12	20905250	Missense	C	T	0.158	p.S677F
8191	*SLFN12*	55106	17	33749546	Missense	A	G	0.065	p.C168R
8191	*SOS2*	6655	14	50626524	Missense	C	A	0.167	p.V493F
8191	*SPATA18*	132671	4	52935984	Splice_Site	C	A	0.209	c.423-3C>A
8191	*SPHKAP*	80309	2	228882679	Missense	C	T	0.153	p.R964K
8191	*SPNS3*	201305	17	4356394	Missense	T	A	0.273	p.V336D
8191	*SPOCD1*	90853	1	32280610	Missense	T	C	0.061	p.T109A
8191	*SRSF4*	6429	1	29485976	Missense	A	C	0.143	p.S91R
8191	*SYNJ2*	8871	6	158489706	Missense	C	T	0.181	p.A582V
8191	*TCP10*	6953	6	167789489	Missense	G	A	0.213	p.T218M
8191	*TDG*	6996	12	104376700	Missense	A	C	0.069	p.K201T
8191	*TENM1*	10178	X	123630915	Missense	C	A	0.6	p.V1216L
8191	*TENM3*	55714	4	183721406	Missense	G	A	0.161	p.D2668N
8191	*TFEC*	22797	7	115614307	Frame_Shift_Ins	C	CATCTA(	0.19	p.E62fs
8191	*FSF12-TNFSF*	407977	17	7462969	Missense	A	G	0.053	p.N176S
8191	*TTN*	7273	2	179634421	Missense	T	G	0.226	p.T2963P
8191	*UGCG*	7357	9	114676902	Missense	A	G	0.281	p.K39R
8191	*USP8*	9101	15	50784950	Missense	C	T	0.118	p.R763W
8191	*USP8*	9101	15	50784955	Missense	C	A	0.108	p.N764K
8191	*VPS35*	55737	16	46710538	Nonsense	G	A	0.154	p.Q291X
8191	*WDR93*	56964	15	90260145	Missense	T	A	0.154	p.S254T
8191	*ZFYVE9*	9372	1	52729497	Missense	G	A	0.24	p.R744K
8191	*ZNF106*	64397	15	42758294	Missense	G	A	0.07	p.R25W
8191	*ZNF229*	7772	19	44934151	Missense	C	T	0.053	p.E269K
8191	*ZNF292*	23036	6	87968565	Missense	A	G	0.068	p.I1740V
8191	*ZNF326*	284695	1	90470688	Splice_Site	A	G	0.303	c.98-4A>G
8191	*ZNF799*	90576	19	12502753	Missense	G	C	0.195	p.H153Q
8191	*ZPBP2*	124626	17	38028634	Missense	G	T	0.071	p.S173I
8188	*ACAN*	176	15	89389030	Missense	G	T	0.042	p.G449V
8188	*ADAM29*	11086	4	175898555	Missense	C	T	0.067	p.P627S
8188	*ADAMTS16*	170690	5	5209210	Missense	G	A	0.188	p.A486T
8188	*ADAR*	103	1	154573967	Missense	T	C	0.045	p.K384R
8188	*ANKRD31*	256006	5	74443132	Missense	C	T	0.053	p.D702N
8188	*ANKZF1*	55139	2	220097027	Missense	C	T	0.042	p.R103C
8188	*AOAH*	313	7	36763672	Missense	C	T	0.049	p.D28N
8188	*CAMKK1*	84254	17	3775848	Missense	T	C	0.052	p.E413G
8188	*CAST*	831	5	96084271	Missense	G	A	0.067	p.R365Q
8188	*CCDC50*	152137	3	191093175	Missense	T	A	0.056	p.I258N
8188	*CHRNA2*	1135	8	27328511	Missense	G	A	0.062	p.T22I
8188	*CLEC1B*	51266	12	10150961	Missense	G	A	0.062	p.S28F
8188	*CSMD2*	114784	1	34068093	Missense	A	C	0.273	p.F2198V
8188	*CSRNP1*	64651	3	39184959	Missense	C	T	0.041	p.V453I
8188	*CXorf22*	170063	X	35969297	Missense	G	A	0.065	p.V236M
8188	*CYLC2*	1539	9	105767536	Missense	G	A	0.121	p.G208D
8188	*CYLC2*	1539	9	105767481	Missense	A	G	0.094	p.K190E
8188	*DGKH*	160851	13	42803263	Missense	T	C	0.088	p.V1201A
8188	*DOCK8*	81704	9	433978	Splice_Site	A	G	0.056	c.4886+3A>G
8188	*EHHADH*	1962	3	184922294	Missense	C	T	0.064	p.A274T
8188	*EIF2AK3*	9451	2	88874891	Missense	C	A	0.073	p.A704S
8188	*FARP2*	9855	2	242432704	Splice_Site	G	C	0.041	c.2896-4G>C
8188	*FHOD3*	80206	18	34324091	Missense	G	A	0.065	p.V1326I
8188	*GML*	2765	8	143922620	Missense	C	T	0.051	p.R54C
8188	*GNAQ*	2776	9	80537095	Nonsense	G	T	0.129	p.Y101X
8188	*RNPUL2-BSC*	100534595	11	62458275	Missense	T	C	0.068	p.K268R
8188	*KDM7A*	80853	7	139827350	Missense	G	A	0.051	p.A198V
8188	*KIF1B*	23095	1	10327514	Missense	G	A	0.068	p.R169H
8188	*KRT8*	3856	12	53298699	Missense	G	A	0.125	p.R23C

8188	*KRTAP4-8*	728224	17	39254054	Missense	A	T	0.15	p.C95S
8188	*KRTAP9-2*	83899	17	39383172	Missense	C	T	0.121	p.T89I
8188	*LCA5*	167691	6	80228535	Missense	T	G	0.051	p.D26A
8188	*.OC101929655*		15	81625385	Missense	C	T	0.069	p.C893Y
8188	*LRRC8E*	80131	19	7963952	Missense	G	A	0.07	p.R182Q
8188	*MADD*	8567	11	47306628	Missense	G	A	0.053	p.R765Q
8188	*MAGEF1*	64110	3	184428903	Missense	T	G	0.04	p.E236A
8188	*MPHOSPH10*	10199	2	71366957	Missense	C	A	0.07	p.L425M
8188	*MTERF1*	7978	7	91503228	Missense	C	T	0.075	p.A274T
8188	*MUC21*	394263	6	30955179	Missense	G	C	0.346	p.E409D
8188	*MUC22*	100507679	6	30995786	Missense	A	G	0.041	p.T860A
8188	*MYCT1*	80177	6	153019099	Frame_Shift_Del	GAGAT	G	0.333	p.R21fs
8188	*MYZAP*	100820829	15	57925836	Missense	C	T	0.07	p.A277V
8188	*NAF1*	92345	4	164048199	Missense	G	C	0.066	p.L368V
8188	*NBPF25P*	101929780	1	148582481	Missense	A	G	0.222	p.T239A
8188	*NEB*	4703	2	152527572	Missense	C	T	0.125	p.V1491M
8188	*NOP56*	10528	20	2635212	Missense	A	G	0.047	p.I121V
8188	*NPSR1*	387129	7	34889182	Missense	A	G	0.062	p.Q344R
8188	*NUP160*	23279	11	47857253	Missense	T	C	0.12	p.T351A
8188	*OR2T6*	254879	1	248551636	Missense	T	G	0.041	p.S243A
8188	*OR51B5*	282763	11	5462255	Missense	C	G	0.062	p.V164L
8188	*OR51I1*	390063	11	5462105	Missense	T	C	0.054	p.T214A
8188	*OR51Q1*	390061	11	5443969	Missense	A	C	0.053	p.Y180S
8188	*OR5B12*	390191	11	58207203	Missense	C	T	0.053	p.C141Y
8188	*OR5H15*	403274	3	97888337	Missense	C	T	0.065	p.P265L
8188	*OR6C70*	390327	12	55863224	Missense	C	G	0.101	p.K233N
8188	*OR8D1*	283159	11	124180282	Missense	A	C	0.091	p.C127W
8188	*OVGP1*	5016	1	111957311	Missense	G	C	0.051	p.H604Q
8188	*PCDH15*	65217	10	55755491	Missense	C	T	0.079	p.R934Q
8188	*PCLO*	27445	7	82508649	Splice_Site	T	A	0.062	c.13654+4A>T
8188	*PRR21*	643905	2	240981375	Missense	T	A	0.067	p.K342M
8188	*RAB3GAP1*	22930	2	135893372	Missense	A	G	0.067	p.N598S
8188	*RAB4B*	53916	19	41289936	Missense	G	A	0.057	p.R129Q
8188	*RETSAT*	54884	2	85570857	Missense	G	A	0.138	p.A533V
8188	*RETSAT*	54884	2	85570849	Missense	C	T	0.129	p.G536R
8188	*RNU6-71P*	100873774	6	56463410	Missense	T	C	0.087	p.Q1812R
8188	*RNU6-81P*	100873780	2	132237927	Missense	C	A	0.182	p.R221S
8188	*RXRA*	6256	9	137323779	Missense	C	T	0.054	p.R358W
8188	*SCLT1*	132320	4	129867280	Missense	T	A	0.12	p.S441C
8188	*SH3RF2*	153769	5	145393364	Missense	C	T	0.047	p.R267C
8188	*SH3RF2*	153769	5	145442203	Missense	G	C	0.041	p.G710A
8188	*SIGLEC12*	89858	19	52001394	Missense	A	T	0.053	p.L428H
8188	*SMYD4*	114826	17	1704296	Missense	C	A	0.077	p.R131I
8188	*SPATA16*	83893	3	172835125	Missense	T	C	0.071	p.M133V
8188	*SPATA16*	83893	3	172835082	Missense	C	T	0.066	p.G147E
8188	*SPOCD1*	90853	1	32279629	Missense	G	A	0.046	p.R436W
8188	*TRMT12*	55039	8	125463250	Missense	T	C	0.043	p.W28R
8188	*TTN*	7273	2	179634421	Missense	T	G	0.333	p.T2963P
8188	*TTN*	7273	2	179629461	Missense	C	T	0.105	p.V3261M
8188	*TTN*	7273	2	179397561	Missense	C	T	0.08	p.R34594H
8188	*TTN*	7273	2	179421694	Missense	A	G	0.065	p.I29396T
8188	*TTN*	7273	2	179623758	Missense	C	T	0.057	p.S3419N
8188	*USP8*	9101	15	50784950	Missense	C	T	0.185	p.R763W
8188	*USP8*	9101	15	50784955	Missense	C	A	0.133	p.N764K
8188	*VPS53*	55275	17	526907	Missense	G	T	0.086	p.L328I
8188	*WDYHV1*	55093	8	124448804	Missense	T	A	0.07	p.F48I
8188	*ZNF268*	10795	12	133780851	Missense	G	C	0.056	p.R860T
8188	*ZNF292*	23036	6	87968565	Missense	A	G	0.079	p.I1740V
8188	*ZNF438*	220929	10	31138817	Missense	G	A	0.053	p.P163S
8188	*ZNF568*	374900	19	37487866	Missense	A	T	0.136	p.S425C
8188	*ZNF568*	374900	19	37487873	Missense	C	A	0.13	p.A427E
8186	*ACAN*	176	15	89381891	Splice_Site	C	T	0.301	c.71-3C>T
8186	*ACCSL*	390110	11	44076778	Missense	A	T	0.257	p.E359V
8186	*ACTN1*	87	14	69376113	Missense	G	T	0.368	p.S172R
8186	*ARHGEF37*	389337	5	149001551	Missense	A	C	0.043	p.M421L
8186	*ARMC4*	55130	10	28250610	Missense	C	A	0.093	p.D425Y
8186	*ARMCX4*	100131755	X	100749127	Missense	A	G	0.065	p.I1851V
8186	*ASCL3*	56676	11	8959545	Missense	C	A	0.105	p.R55L
8186	*ATG4D*	84971	19	10657649	Missense	C	T	0.267	p.R210C
8186	*ATL2*	64225	2	38604351	Missense	A	G	0.073	p.W18R
8186	*ATP10B*	23120	5	160061559	Missense	C	G	0.212	p.V395L
8186	*BRMS1*	25855	11	66105194	Missense	G	A	0.077	p.A273V
8186	*C19orf57*	79173	19	14000870	Missense	C	T	0.056	p.G267R
8186	*C6orf10*	10665	6	32299822	Missense	G	A	0.073	p.P145L
8186	*C6orf15*	29113	6	31079643	Missense	T	C	0.045	p.K165E
8186	*C9orf114*	51490	9	131584830	Missense	G	A	0.129	p.V279M
8186	*CCDC129*	223075	7	31682417	Missense	A	G	0.299	p.K488R
8186	*CHST15*	51363	10	125805330	Missense	C	A	0.186	p.E133D
8186	*CPNE4*	131034	3	131624196	Missense	G	T	0.207	p.A49E
8186	*DNAH7*	56171	2	196681503	Missense	G	A	0.062	p.R3204C

8186	*DNTT*	1791	10	98078272	Missense	C	T	0.247	p.H123Y
8186	*DPP9*	91039	19	4700246	Missense	C	A	0.429	p.Q352H
8186	*EXD3*	54932	9	140262426	Missense	C	T	0.083	p.A160T
8186	*FAM107A*	11170	3	58555583	Missense	T	C	0.111	p.Y2C
8186	*FHAD1*	114827	1	15708540	Missense	A	G	0.051	p.I1325V
8186	*FLG*	2312	1	152279729	Missense	C	T	0.051	p.G2545R
8186	*FMN1*	342184	15	33446311	Missense	G	C	0.052	p.P269A
8186	*FNDC1*	84624	6	159653372	Missense	C	T	0.227	p.P610S
8186	*FRMPD4*	9758	X	12735245	Missense	C	A	0.267	p.D889E
8186	*FUT6*	2528	19	5831672	Missense	G	C	0.222	p.R303G
8186	*GATA3*	2625	10	8097678	Missense	C	A	0.061	p.N20K
8186	*GNAQ*	2776	9	80537112	Missense	T	A	0.107	p.T96S
8186	*GNAQ*	2776	9	80537095	Nonsense	G	T	0.1	p.Y101X
8186	*H6PD*	9563	1	9324213	Missense	C	T	0.056	p.P554L
8186	*HCN1*	348980	5	45267278	Missense	C	T	0.278	p.V566M
8186	*KANK1*	23189	9	712156	Missense	T	G	0.053	p.S464A
8186	*KANK3*	256949	19	8389519	Missense	C	T	0.255	p.D760N
8186	*KCNJ5*	3762	11	128772576	Missense	C	A	0.263	p.G105V
8186	*KCNMB2*	10242	3	178546013	Missense	C	T	0.389	p.T92M
8186	*KCTD3*	51133	1	215768764	Missense	G	A	0.13	p.S295N
8186	*KMT2C*	58508	7	151945291	Missense	G	A	0.5	p.P743L
8186	*LILRA4*	23547	19	54848157	Missense	A	G	0.114	p.Y404H
8186	*LOC113230*	113230	19	14185226	Missense	T	C	0.069	p.V198A
8186	*LRG1*	116844	19	4538599	Missense	G	A	0.07	p.P133S
8186	*LTF*	4057	3	46495823	Frame_Shift_Ins	C	CACTCCTA	0.219	p.V225fs
8186	*MAP1A*	4130	15	43818052	Missense	G	A	0.062	p.D1461N
8186	*MAP2K3*	5606	17	21203941	Missense	G	A	0.075	p.A84T
8186	*MBTD1*	54799	17	49297755	Missense	C	A	0.186	p.S75I
8186	*MECOM*	2122	3	168806883	Missense	C	G	0.172	p.D1164H
8186	*MGAM*	8972	7	141750064	Missense	T	C	0.242	p.C873R
8186	*MGAM*	8972	7	141736744	Missense	G	A	0.232	p.R733Q
8186	*MROH2A*	339766	2	234704345	Missense	T	C	0.087	p.V341A
8186	*MTHFD1L*	25902	6	151265685	Missense	G	A	0.088	p.A502T
8186	*MUC17*	140453	7	100677210	Missense	C	T	0.053	p.T838I
8186	*MYO7B*	4648	2	128388862	Missense	G	C	0.042	p.E1647D
8186	*MYO9B*	4650	19	17273802	Missense	T	A	0.25	p.L514Q
8186	*NEK1*	4750	4	170354703	Missense	C	T	0.2	p.E960K
8186	*NMRAL1*	57407	16	4511926	Missense	G	A	0.065	p.P252L
8186	*NPEPPS*	9520	17	45664593	Splice_Site	C	T	0.138	c.981-3C>T
8186	*NRCAM*	4897	7	107847998	Missense	C	G	0.185	p.W394S
8186	*OBSCN*	84033	1	228528563	Missense	C	G	0.15	p.Q6848E
8186	*ODF3L1*	161753	15	76019798	Missense	C	T	0.141	p.H248Y
8186	*OR51M1*	390059	11	5411395	Missense	C	A	0.049	p.P256H
8186	*OR52I2*	143502	11	4608262	Missense	A	C	0.13	p.I74L
8186	*OR6T1*	219874	11	123814356	Missense	G	A	0.063	p.R64W
8186	*OR9Q1*	219956	11	57947716	Missense	C	T	0.042	p.S267L
8186	*PAX1*	5075	20	21687604	Missense	G	T	0.202	p.G272V
8186	*PAX6*	5080	11	31822387	Missense	T	G	0.156	p.R139S
8186	*PLA2R1*	22925	2	160862163	Missense	G	A	0.192	p.R612C
8186	*PLEKHG2*	64857	19	39915758	Missense	C	G	0.065	p.P1329A
8186	*PMFBP1*	83449	16	72174385	Missense	C	T	0.2	p.V100I
8186	*POM121L2*	94026	6	27279774	Missense	C	T	0.042	p.R59Q
8186	*POTEG*	404785	14	19553451	Missense	C	T	0.5	p.S12F
8186	*PPARGC1B*	133522	5	149212430	Missense	G	A	0.068	p.R265Q
8186	*PRRC2B*	84726	9	134351102	Nonsense	C	T	0.229	p.R1196X
8186	*RBM33*	155435	7	155465602	Missense	G	A	0.115	p.G55D
8186	*RNFT2*	84900	12	117217115	Missense	G	A	0.06	p.V282M
8186	*RREB1*	6239	6	7230680	Missense	G	T	0.051	p.G783V
8186	*SBK3*	100130827	19	56052465	Missense	G	A	0.075	p.A276V
8186	*SCNN1G*	6340	16	23221162	Missense	C	T	0.18	p.S390L
8186	*SEMA5A*	9037	5	9054230	Missense	T	G	0.176	p.E886D
8186	*SKIV2L2*	23517	5	54640404	Missense	T	G	0.279	p.F321V
8186	*SLC17A4*	10050	6	25779393	Missense	G	A	0.166	p.E437K
8186	*SORCS3*	22986	10	106976772	Nonsense	G	T	0.244	p.E876X
8186	*SYCP2*	10388	20	58452471	Missense	G	A	0.261	p.S1040L
8186	*TCHH*	7062	1	152081921	Missense	G	C	0.07	p.L1258V
8186	*TEPP*	374739	16	58019396	Missense	G	A	0.047	p.S233N
8186	*THAP8*	199745	19	36530332	Missense	G	A	0.064	p.R189W
8186	*TMC4*	147798	19	54664209	Missense	G	C	0.045	p.Q683E
8186	*TMEM178B*	100507421	7	141137414	Missense	G	A	0.091	p.R168H
8186	*TP53*	7157	17	7579472	Missense	G	C	0.077	p.P33R
8186	*TREML4*	285852	6	41196605	Missense	T	C	0.055	p.W73R
8186	*TRIM25*	7706	17	54991054	Missense	G	C	0.216	p.P99R
8186	*TSPEAR*	54084	21	46101939	Missense	C	T	0.037	p.V34M
8186	*UBR4*	23352	1	19439210	Missense	G	T	0.191	p.T3870N
8186	*USP34*	9736	2	61575224	Missense	A	G	0.284	p.M689T
8186	*USP8*	9101	15	50784950	Missense	C	T	0.167	p.R763W
8186	*USP8*	9101	15	50784955	Missense	C	A	0.159	p.N764K
8186	*ZBTB32*	27033	19	36205622	Missense	C	G	0.314	p.L32V
8186	*ZFHX3*	463	16	72830973	Missense	G	A	0.277	p.P1870S

8186	*ZNF117*	51351	7	64439701	Missense	C	T	0.094	p.C83Y
8186	*ZNF219*	51222	14	21560752	In_Frame_Del	GGAGGCT	G	0.03	p.233_235del
8186	*ZNF302*	55900	19	35173717	Missense	T	A	0.15	p.H59Q
8186	*ZNF776*	284309	19	58264887	Missense	A	G	0.234	p.H130R
8186	*ZZZ3*	26009	1	78034094	Nonsense	G	A	0.133	p.Q797X
8184	*ACSL6*	23305	5	131329858	Missense	A	G	0.034	p.F46L
8184	*ADAM21*	8747	14	70924335	Missense	C	T	0.083	p.P40L
8184	*AGR3*	155465	7	16901609	Missense	A	G	0.214	p.M101T
8184	*AIF1L*	83543	9	133981629	Missense	T	C	0.046	p.C50R
8184	*AKNA*	80709	9	117124731	Missense	G	A	0.042	p.P624L
8184	*ALPK2*	115701	18	56205314	Missense	G	A	0.297	p.A702V
8184	*ALPK2*	115701	18	56205262	Missense	A	C	0.043	p.H719Q
8184	*ALX4*	60529	11	44331509	Missense	C	G	0.036	p.R35T
8184	*AOAH*	313	7	36763672	Missense	C	T	0.053	p.D28N
8184	*ARID3A*	1820	19	971949	Missense	G	A	0.074	p.G556S
8184	*ATP8B4*	79895	15	50226313	Missense	G	T	0.073	p.H452N
8184	*B3GALTL*	145173	13	31821240	Splice_Site	C	T	0.114	c.347+4C>T
8184	*BAGE4*	85317	21	11098728	Missense	C	T	0.063	p.G4R
8184	*BAI1*	575	8	143546035	Missense	G	C	0.068	p.R159P
8184	*BDP1*	55814	5	70800538	Missense	G	A	0.075	p.V778M
8184	*BIRC7*	79444	20	61870727	Missense	G	C	0.053	p.E223Q
8184	*BLZF1*	8548	1	169345868	Missense	A	G	0.083	p.Q40R
8184	*BMP2*	650	20	6750891	Missense	C	T	0.266	p.R40C
8184	*BTNL8*	79908	5	180377470	Missense	G	A	0.042	p.A352T
8184	*BVES-AS1*	154442	6	105606539	Missense	A	G	0.338	p.S228P
8184	*C15orf39*	56905	15	75503147	Missense	G	A	0.117	p.G945D
8184	*C15orf39*	56905	15	75499861	Missense	G	A	0.068	p.G491D
8184	*C17orf75*	64149	17	30668290	Nonsense	G	A	0.353	p.R52X
8184	*C17orf96*	100170841	17	36830562	Missense	G	C	0.13	p.L63V
8184	*C17orf97*	400566	17	260299	Missense	G	A	0.071	p.E56K
8184	*C5orf64*	285668	5	60982841	Missense	G	A	0.053	p.A57T
8184	*C6orf106*	64771	6	34622443	Missense	G	A	0.097	p.P91L
8184	*C6orf58*	352999	6	127902333	Missense	C	A	0.32	p.Q194K
8184	*CABYR*	26256	18	21735686	Missense	C	T	0.098	p.T74M
8184	*CAMK2D*	817	4	114381329	Missense	T	C	0.095	p.D404G
8184	*CAPRIN2*	65981	12	30883198	Missense	G	T	0.342	p.A360D
8184	*CAPRIN2*	65981	12	30881809	Missense	T	C	0.047	p.M519V
8184	*CASD1*	64921	7	94174854	Splice_Site	T	C	0.085	c.1477-3T>C
8184	*CASP9*	842	1	15832543	Missense	T	C	0.061	p.Q138R
8184	*CC2D2A*	57545	4	15518357	Missense	A	C	0.125	p.E376A
8184	*CCDC102B*	79839	18	66513615	Missense	A	G	0.079	p.K298R
8184	*CCDC170*	80129	6	151939181	Missense	G	A	0.047	p.V683I
8184	*CCHCR1*	54535	6	31111148	Missense	T	A	0.053	p.K648M
8184	*CCHCR1*	54535	6	31111125	Missense	G	A	0.047	p.R656C
8184	*CD93*	22918	20	23065638	Nonsense	G	A	0.294	p.Q398X
8184	*CEBPZ*	10153	2	37458668	Missense	G	A	0.033	p.P15S
8184	*CHTF18*	63922	16	840378	Missense	A	G	0.047	p.K244R
8184	*COL1A1*	1277	17	48271809	Splice_Site	C	G	0.289	c.1516-1G>C
8184	*COL24A1*	255631	1	86591837	Missense	G	A	0.075	p.A61V
8184	*COL4A4*	1286	2	227915832	Missense	G	A	0.07	p.P1004L
8184	*CPA6*	57094	8	68421768	Missense	G	C	0.2	p.S173C
8184	*CPN2*	1370	3	194063152	Missense	C	T	0.3	p.G94R
8184	*CSMD2*	114784	1	34090751	Missense	T	A	0.032	p.E1760D
8184	*CSNK2A3*	283106	11	11374269	Missense	A	G	0.064	p.I133T
8184	*CTCFL*	140690	20	56098205	Missense	C	T	0.407	p.E225K
8184	*CTNND2*	1501	5	11110976	Missense	T	G	0.33	p.Q819H
8184	*CTNS*	1497	17	3563963	Missense	C	G	0.036	p.P380A
8184	*CYB5R1*	51706	1	202935911	Missense	T	C	0.035	p.N44S
8184	*CYP11B1*	1584	8	143961102	Missense	C	T	0.062	p.R43Q
8184	*CYP1B1*	1545	2	38302177	Missense	C	A	0.054	p.A119S
8184	*CYP4B1*	1580	1	47282772	Missense	C	T	0.055	p.R376C
8184	*DBI*	1622	2	120125053	Nonsense	C	G	0.057	p.Y28X
8184	*DBI*	1622	2	120125058	Missense	C	T	0.056	p.A30V
8184	*DNAH17*	8632	17	76449472	Missense	G	C	0.238	p.D3494E
8184	*DNAH5*	1767	5	13913926	Missense	T	C	0.235	p.T488A
8184	*DNAJB4*	11080	1	78478739	Missense	G	T	0.281	p.L72F
8184	*DSPP*	1834	4	88534065	Missense	G	A	0.083	p.D243N
8184	*DYNC2LI1*	51626	2	44021826	Missense	T	A	0.064	p.F184Y
8184	*EDNRB*	1910	13	78472390	Missense	G	A	0.237	p.A515V
8184	*ERCC6L2*	375748	9	98683569	Missense	G	T	0.214	p.G435V
8184	*ESPNL*	339768	2	239039841	Missense	A	G	0.053	p.Q829R
8184	*EXOC2*	55770	6	656343	Missense	T	C	0.035	p.Q201R
8184	*F13A1*	2162	6	6174866	Missense	G	A	0.06	p.P565L
8184	*FAM129C*	199786	19	17660300	Missense	G	A	0.062	p.G603S
8184	*FAM178B*	51252	2	97637905	Missense	T	C	0.044	p.I99M
8184	*FAM198A*	729085	3	43095101	Missense	A	G	0.056	p.Q460R
8184	*FAM221B*	392307	9	35826038	Missense	T	C	0.05	p.K41E
8184	*FASN*	2194	17	80037030	Missense	G	A	0.057	p.R2509W
8184	*FLG*	2312	1	152280782	Missense	A	G	0.054	p.Y2194H
8184	*FLG*	2312	1	152281690	Missense	C	T	0.036	p.R1891Q

8184	*FN1*	2335	2	216241226	Missense	A	G	0.263	p.I1961T
8184	*FNDC3B*	64778	3	171969109	Missense	C	G	0.224	p.P190A
8184	*FOXA1*	3169	14	38061604	Missense	G	T	0.28	p.P129T
8184	*FOXRED2*	80020	22	36900271	Missense	T	C	0.031	p.N308S
8184	*FRAS1*	80144	4	79300993	Missense	G	A	0.062	p.E1136K
8184	*GDF15*	9518	19	18499422	Missense	C	G	0.05	p.H202D
8184	*GLDN*	342035	15	51689772	Missense	G	A	0.111	p.S265N
8184	*GLI2*	2736	2	121747406	Missense	G	A	0.076	p.D1306N
8184	*GNRH2*	2797	20	3025107	Missense	C	T	0.04	p.A16V
8184	*HDHD1*	8226	X	6975782	Missense	C	G	0.049	p.C198S
8184	*HIVEP1*	3096	6	12120588	Missense	C	T	0.054	p.T187M
8184	*HLA-A*	3105	6	29910692	Nonsense	C	T	0.3	p.Q78X
8184	*HLA-DQB1*	3119	6	32632598	Missense	A	T	1	p.F119Y
8184	*ICT1*	3396	17	73008804	Missense	G	C	0.088	p.R8P
8184	*IGSF21*	84966	1	18704773	Missense	C	T	0.064	p.R453W
8184	*IL2RB*	3560	22	37524619	Missense	G	T	0.049	p.D391E
8184	*INPP5B*	3633	1	38397369	Missense	C	T	0.034	p.G6S
8184	*INPP5F*	22876	10	121586882	Missense	A	G	0.05	p.N997D
8184	*KAT6B*	23522	10	76781905	In_Frame_Del	GGAA	G	0.119	p.1097_1097del
8184	*KBTBD12*	166348	3	127641968	Missense	C	A	0.048	p.Q22K
8184	*KIAA1211*	57482	4	57181995	Missense	C	T	0.08	p.S776L
8184	*KIF4B*	285643	5	154395458	Missense	G	A	0.043	p.R680H
8184	*KMT2D*	8085	12	49437567	Splice_Site	T	A	0.284	c.5320-2A>T
8184	*KRTAP4-7*	100132476	17	39240819	Missense	C	G	0.111	p.L121V
8184	*KRTAP9-1*	728318	17	39346518	Missense	T	C	0.036	p.I127T
8184	*LANCL2*	55915	7	55433884	Missense	A	C	0.051	p.T56P
8184	*LOC100996693*	100996693	2	220361598	Missense	A	G	0.247	p.I66V
8184	*LOC400794*	400794	1	165513659	Missense	G	T	0.265	p.K42N
8184	*LOC401052*	401052	3	10049098	Missense	T	G	0.636	p.H96P
8184	*LRGUK*	136332	7	133848257	Missense	G	T	0.079	p.D302Y
8184	*LRP1*	4035	12	57577973	Missense	C	T	0.219	p.A2012V
8184	*LRP1B*	53353	2	141114025	Nonsense	C	A	0.333	p.E3806X
8184	*LRRC2*	79442	3	46580591	Missense	G	T	0.081	p.A145E
8184	*MAGEA11*	4110	X	148797410	Splice_Site	C	T	0.22	c.267-3C>T
8184	*MFSD1*	64747	3	158520011	Missense	C	T	0.067	p.P73S
8184	*MFSD6L*	162387	17	8701799	Missense	G	T	0.072	p.P214T
8184	*MGAT1*	4245	5	180219304	Missense	C	T	0.036	p.R223Q
8184	*MICAL2*	9645	11	12265546	Missense	T	C	0.282	p.S891P
8184	*MINK1*	50488	17	4797305	Missense	G	A	0.044	p.V834I
8184	*MKNK2*	2872	19	2050823	Missense	G	T	0.078	p.Q10K
8184	*MLXIP*	22877	12	122626247	Missense	G	T	0.321	p.S883I
8184	*MMAA*	166785	4	146576418	Missense	G	C	0.042	p.Q363H
8184	*MSLN*	10232	16	818559	Missense	A	G	0.099	p.M593V
8184	*MUC22*	100507679	6	30993776	Missense	A	T	0.035	p.T190S
8184	*MUC7*	4589	4	71347171	Missense	C	T	0.488	p.A237V
8184	*MYLK*	4638	3	123419733	Missense	A	G	0.048	p.L861P
8184	*N4BP2*	55728	4	40138676	Missense	A	G	0.111	p.T1587A
8184	*NAV2*	89797	11	20066751	Nonsense	C	A	0.256	p.S1169X
8184	*NCKAP5*	344148	2	133541454	Missense	A	G	0.091	p.I977T
8184	*NCKAP5*	344148	2	133541575	Missense	C	T	0.041	p.V937I
8184	*NCOA7*	135112	6	126210395	Missense	T	G	0.042	p.S399A
8184	*NHLRC2*	374354	10	115644040	Missense	G	A	0.074	p.V314I
8184	*NMS*	129521	2	101095857	Missense	G	A	0.115	p.A102T
8184	*NOL4*	8715	18	31537357	Missense	C	A	0.3	p.R454L
8184	*NOL8*	55035	9	95061229	Missense	A	G	0.182	p.S1036P
8184	*NPR3*	4883	5	32786389	Missense	A	G	0.091	p.N305D
8184	*NPTX1*	4884	17	78449948	Missense	C	T	0.055	p.G100D
8184	*NRXN1*	9378	2	50699451	Missense	C	T	0.204	p.E1117K
8184	*NWD1*	284434	19	16855265	Missense	A	G	0.059	p.I78V
8184	*OR10A5*	144124	11	6867707	Nonsense	C	A	0.25	p.S265X
8184	*OR10A6*	390093	11	7949791	Missense	A	C	0.179	p.V140G
8184	*OR10A6*	390093	11	7949860	Missense	G	A	0.156	p.A117V
8184	*OR10G2*	26534	14	22102374	Missense	T	C	0.12	p.R209G
8184	*OR10G4*	390264	11	123886865	Missense	T	A	0.333	p.V195E
8184	*OR10W1*	81341	11	58034543	Missense	C	T	0.038	p.R263Q
8184	*OR1N2*	138882	9	125315557	Missense	T	C	0.043	p.W37R
8184	*OR2A14*	135941	7	143826573	Missense	C	T	0.047	p.A123V
8184	*OR4C6*	219432	11	55433305	Nonsense	C	A	0.314	p.C221X
8184	*OR51B5*	282763	11	5364276	Missense	G	A	0.15	p.P160L
8184	*OR52E6*	390078	11	5862731	Missense	A	G	0.044	p.W133R
8184	*OR6T1*	219874	11	123814356	Missense	G	A	0.042	p.R64W
8184	*OVGP1*	5016	1	111957311	Missense	G	C	0.058	p.H604Q
8184	*PAG1*	55824	8	81888870	Missense	C	T	0.21	p.G403E
8184	*PAK2*	5062	3	196509577	Missense	C	G	0.083	p.S20R
8184	*PCDHA1*	56147	5	140174865	Missense	G	C	0.076	p.V106L
8184	*PCDHA1*	56147	5	140183058	Missense	G	A	0.057	p.C759Y
8184	*PCDHA1*	56147	5	140168070	Missense	A	G	0.046	p.Y732C
8184	*PCDHA3*	56145	5	140181734	Missense	A	G	0.1	p.I318V
8184	*PCDHA3*	56145	5	140186937	Missense	G	T	0.051	p.E55D
8184	*PCDHA5*	56143	5	140222641	Missense	A	G	0.071	p.K579E

8184	*PCDHB4*	56131	5	140502997	Missense	A	C	0.094	p.S473R
8184	*PCNT*	5116	21	47786524	Missense	A	G	0.047	p.T879A
8184	*PCNXL2*	80003	1	233122091	Missense	G	A	0.2	p.S1996F
8184	*PDE1C*	5137	7	31917651	Splice_Site	T	C	0.458	c.426-2A>G
8184	*PDXDC1*	23042	16	15111218	Missense	C	T	0.037	p.P301L
8184	*PEAR1*	375033	1	156883493	Missense	G	A	0.107	p.R885H
8184	*PEAR1*	375033	1	156883546	Missense	A	G	0.09	p.N903D
8184	*PLEKHA6*	22874	1	204237416	Missense	C	T	0.049	p.V43I
8184	*PMS2*	5395	7	6026988	Missense	G	A	0.065	p.P470S
8184	*PPAN-P2RY11*	692312	19	10224548	Missense	G	A	0.048	p.A507T
8184	*PRB4*	5545	12	11461706	Missense	G	T	0.143	p.P71T
8184	*PRDM2*	7799	1	14105139	Missense	T	A	0.065	p.D283E
8184	*PRH1*	5554	12	11214145	Nonsense	C	T	0.046	p.W250X
8184	*PRR5*	55615	22	45075738	Missense	T	C	0.069	p.M20T
8184	*PTPRN2*	5799	7	157931144	Missense	C	T	0.036	p.S325N
8184	*PTPRS*	5802	19	5244392	Missense	C	T	0.269	p.V364I
8184	*PTPRZ1*	5803	7	121681026	Missense	C	A	0.2	p.H1932N
8184	*RASAL2*	9462	1	178423608	Missense	C	A	0.222	p.A679E
8184	*RBBP6*	5930	16	24567014	Missense	G	A	0.18	p.D143N
8184	*RBMXL3*	139804	X	114425400	Missense	G	A	0.039	p.G466R
8184	*REST*	5978	4	57796900	Missense	G	A	0.062	p.V626I
8184	*REV3L*	5980	6	111632393	Nonsense	G	A	0.419	p.R2814X
8184	*RNU5D-1*	26830	5	80738539	Missense	G	A	0.167	p.S261L
8184	*RTP4*	64108	3	187088812	Missense	C	T	0.042	p.T131M
8184	*RTP5*	285093	2	242814009	Missense	G	A	0.039	p.G101E
8184	*SETD2*	29072	3	47125385	Missense	G	A	0.042	p.P1962L
8184	*SETX*	23064	9	135205006	Missense	G	C	0.058	p.A660G
8184	*SH3BP4*	23677	2	235949877	Missense	T	C	0.043	p.M155T
8184	*SH3TC2*	79628	5	148406199	Missense	G	A	0.191	p.R997W
8184	*SIPA1L1*	26037	14	72117205	Missense	C	G	0.227	p.R658G
8184	*SIX5*	147912	19	46268902	Missense	C	T	0.042	p.V693M
8184	*SLC15A2*	6565	3	121643804	Missense	C	T	0.051	p.L350F
8184	*SLC3A1*	6519	2	44508607	Frame_Shift_Del	GA	G	0.348	p.G231fs
8184	*SLC52A3*	113278	20	744415	Missense	G	A	0.036	p.P267L
8184	*SLC5A8*	160728	12	101560321	Missense	T	C	0.281	p.T493A
8184	*SLC6A16*	28968	19	49794028	Splice_Site	G	T	0.188	c.1779-4C>A
8184	*SLFN12*	55106	17	33749168	Missense	C	T	0.211	p.D294N
8184	*SLIT3*	6586	5	168180081	Missense	C	T	0.042	p.G618S
8184	*SPDYE2*	441273	7	102194965	Missense	A	G	0.115	p.K107R
8184	*SPEF2*	79925	5	35692775	Missense	T	A	0.082	p.N616K
8184	*SPEG*	10290	2	220350190	Missense	A	T	0.26	p.S2578C
8184	*STAP2*	55620	19	4327352	Missense	C	A	0.226	p.K207N
8184	*TON1-GTF2A1*	286749	2	48848003	Missense	G	T	0.167	p.R716I
8184	*STRN3*	29966	14	31381351	Missense	T	C	0.069	p.N471S
8184	*SUPT20H*	55578	13	37583831	Missense	G	A	0.088	p.T773M
8184	*SYNE2*	23224	14	64519455	Missense	A	G	0.06	p.I2942V
8184	*SYT8*	90019	11	1858262	Missense	C	T	0.034	p.T303M
8184	*TAF1B*	9014	2	10059770	Missense	G	T	0.086	p.E462D
8184	*TAOK3*	51347	12	118682751	Missense	C	T	0.064	p.S47N
8184	*TBC1D1*	23216	4	37903756	Missense	T	C	0.043	p.S14P
8184	*TELO2*	9894	16	1555589	Missense	A	G	0.081	p.Q674R
8184	*TEP1*	7011	14	20872881	Missense	G	T	0.079	p.N307K
8184	*TET2*	54790	4	106197207	Nonsense	G	A	0.628	p.W1847X
8184	*TIMM44*	10469	19	7999089	Missense	C	T	0.295	p.R143Q
8184	*TLL1*	7092	4	166996054	Missense	A	T	0.329	p.N738I
8184	*TMCO6*	55374	5	140023238	Missense	C	G	0.042	p.T299S
8184	*TMEM128*	85013	4	4249884	Missense	G	T	0.094	p.L16I
8184	*TMEM14B*	81853	6	10756728	Missense	C	T	0.122	p.R108C
8184	*TMEM156*	80008	4	39000305	Missense	A	G	0.119	p.S105P
8184	*TMEM177*	80775	2	120438523	Missense	A	G	0.043	p.I32V
8184	*TMEM8A*	58986	16	426432	Missense	T	C	0.053	p.I310V
8184	*TMPRSS6*	164656	22	37462936	Missense	A	G	0.043	p.V749A
8184	*TMPRSS9*	360200	19	2422177	Missense	G	A	0.049	p.S793N
8184	*TNFRSF11B*	4982	8	119945444	Missense	G	T	0.266	p.D42E
8184	*TNS1*	7145	2	218674697	Missense	C	T	0.079	p.V1604I
8184	*TOP2A*	7153	17	38572683	Nonsense	C	A	0.312	p.E87X
8184	*TSKU*	25987	11	76507582	Missense	G	A	0.037	p.V308I
8184	*TTC16*	158248	9	130489743	Missense	A	G	0.125	p.E588G
8184	*TTF1*	7270	9	135277130	Missense	C	A	0.043	p.G360V
8184	*TUBA3D*	113457	2	132237927	Missense	C	A	0.333	p.R221S
8184	*UBR4*	23352	1	19510394	Splice_Site	A	G	0.096	c.2099-4T>C
8184	*UCK2*	7371	1	165859601	Splice_Site	G	A	0.2	c.259+1G>A
8184	*UNC13B*	10497	9	35228038	Missense	C	T	0.273	p.P17S
8184	*UNC13D*	201294	17	73832495	Missense	C	T	0.283	p.M438I
8184	*UNC80*	285175	2	210824272	Missense	G	A	0.222	p.R2478Q
8184	*UVSSA*	57654	4	1349029	Missense	G	A	0.082	p.R391H
8184	*VN1R4*	317703	19	53770563	Missense	A	C	0.116	p.L119R
8184	*VPS13D*	55187	1	12302641	Missense	A	C	0.214	p.K57Q
8184	*WBSCR27*	155368	7	73249165	Missense	T	A	0.061	p.R216W
8184	*WBSCR28*	135886	7	73279482	Missense	T	C	0.053	p.W78R

8184	*WDR49*	151790	3	167217964	Missense	A	G	0.25	p.L651P
8184	*WDR91*	29062	7	134889141	Missense	G	A	0.042	p.P257L
8184	*WRAP53*	55135	17	7606722	Missense	C	G	0.035	p.A522G
8184	*ZNF239*	8187	10	44052630	Missense	A	G	0.212	p.F300L
8184	*ZNF428*	126299	19	44118191	Missense	G	A	0.043	p.D640N
8184	*ZNF614*	80110	19	52519608	Missense	C	T	0.049	p.V415I
8184	*ZNF804A*	91752	2	185800905	Missense	A	T	0.067	p.Q261L
8184	*ZNF813*	126017	19	53993994	Missense	G	A	0.073	p.D170N

*Based on NCBI human reference genome GRC Build 37 (hg19).

Supplementary Table 4. Validated somatic mutations in the discovery cohort by Sanger sequencing.

No.	Hugo Symbol	Entrez Gene Id	Chromosome	Start position*	End Position*	Nucleotide change	Forward primer	Reverse primer	Validation
1	*ACCSL*	390110	11	44076778	44076778	NM_001031854:exon9:c.A1076T	CCCTTCTGATGTACCACCCC	CCTTGTGGTTCTCGTTCCCA	YES
2	*ANK3*	288	10	61846611	61846611	NM_001149:exon8:c.A974C	CAGCAGTGACTTAACCAAACCTG	TCCTGCAAGCCAGTGCTATT	YES
3	*ANKRD22*	118932	10	90591731	90591731	NM_144590:exon2:c.T74G	AACGATTCTCACATGCCCTCG	CCTGCTTTTGTTTTTCCCGACC	YES
4	*ARHGAP29*	9411	1	94652126	94652126	NM_004815:exon16:c.C1709T	TCCCTGTTACCCAGAGTATGAGA	AGTGCTACTGATTGAGCTGGT	YES
5	*ATP6V0A2*	23545	12	124203252	124203252	NM_012463:exon2:c.196+4A>G	ACAAGAGTGTACATCCCCCAA	GGGTAACAACAAAATAACCAGGC	YES
6	*ATRX*	546	X	76953110	76953110	NM_000489:exon4:c.C203T	CAGTGTATTCCCCGCAGCAT	GTGACGATCCTGAAGACTTGGA	YES
7	*BNIP3*	664	10	133787327	133787327	NM_004052:exon2:c.G167A	GCCCACTGTCCTAGAGGTGA	GAGGGATGGTTCCTGGTGTG	YES
8	*BVES-AS1*	154442	6	105606539	105606539	NM_022361:exon4:c.T682C	TTGTGTCAGGGGTGATCTGC	GTCAGTGGGCTTTTTCAACCT	YES
9	*C17orf58*	284018	17	65989064	65989064	NM_181656:exon2:c.T199C	CTGGCAATGGTGTTTGGTCG	CAGCTGCAATAAGCCGTGTC	YES
10	*CCDC102B*	79839	18	66504349	66504349	NM_024781:exon2:c.A349T	TTTGTCACTTCCTTCTTCCTCCC	AAAAGACCATGCGGTGGTGG	YES
11	*CCDC129*	223075	7	31682417	31682417	NM_194300:exon10:c.A1433G	GCCACCCAAACCTCACTCAC	CAAGGCATTCGCTTCTTGGC	YES
12	*CCDC73*	493860	11	32676427	32676427	NM_001008391:exon10:c.A737G	CCTGATCCACAACCGCAGAT	TGCAAAGGCGGAGTATTTTTGT	YES
13	*CCDC90B*	60492	11	82984731	82984731	NM_001286120:exon5:c.G208T	GAAACCAGTCGAATCAGAGCAG	TGAGGAAGCCAGAGACTCTACA	YES
14	*CNST*	163882	1	246784733	246784733	NM_001139459:exon3:c.C382A	TAGCCATTAGCTCTGGCATCTG	TCCAAACACTGCTAGGGGAAT	YES
15	*CNTROB*	116840	17	7838452	7838452	NM_001037144:exon4:c.A583C	TGCCCCCTTTACTCCTTCAG	TTTTCAACACTTGGGTCCCCT	YES
16	*CXorf67*	340602	X	51150445	51150445	NM_203407:exon1:c.A577C	AAGATCCTGCTGCTTCCGTC	AGATGCGTGGCTTAGGAGTG	YES
17	*DDX3X*	1654	X	41204711	41204711	NM_001193417:exon11:c.G1177A	ACCCAGCCCAAATGGGTTAT	GGTGTCCGCCACACTATGAT	YES
18	*DGAT2L6*	347516	X	69419150	69419150	NM_198512:exon2:c.T115G	CCAGCCCAATGTGCTCTTCT	GCCCCTGTCTCTTACCTTGAC	YES
19	*DHX8*	1659	17	41570872	41570872	NM_001302623:exon7:c.A923G	AGTTATCCCAGGCCATCGTG	CAGATGTGTCTGGCTCTCCC	YES
20	*DMD*	1756	X	32632562	32632562	NM_000109:exon12:c.G1316A	TCCCATCAACCATGTCATCTGT	TAGATGCCCCCAAATGCGAA	YES
21	*DMXL2*	23312	15	51772183	51772183	NM_001174117:exon23:c.A4810G	TCATGAGTGAGAAAGGTAGCACTT	TCCACTACCACTGCCTACCA	YES
22	*DNAH5*	1767	5	13727663	13727663	NM_001369:exon70:c.C11986T	TTGGGTGGCATGACACAGTT	TGGACTTTTTACCTGGGCGAT	YES
23	*DPP9*	91039	19	4700246	4700246	NM_139159:exon10:c.G1056T	ATGTGCCTACAGACAGACCG	CGCATTCTAGGCACAGGTCC	YES
24	*ENTHD2*	146705	17	79202760	79202760	NM_144679:exon12:c.G1546C	CCTGCAGCCTCTGAGTTCAA	CCCTAGGGTCGTCCTCTCAA	YES
25	*FAM135A*	57579	6	71187020	71187020	NM_001162529:exon6:c.A527C	TTTAGCAAAGTACACGTTTTCACT	GCTGCACTTTAGCCCCCATA	YES
26	*FAM135A*	57579	6	71187020	71187020	NM_001162529:exon6:c.A527C	TCTTTAGCAAAGTACACGTTTTCAC	TGAAGCTGCACTTTAGCCCC	YES
27	*FAM205A*	259308	9	34725438	34725438	NM_001141917:exon4:c.T1799C	CTTGCCCTGGTGGATTTGTC	GTCCATCTCAGTGCTCACCA	YES
28	*FAM47C*	442444	X	37026830	37026830	NM_001013736:exon1:c.C347T	CTAGCGACTAGAGCGTCAGG	GTACATGGCCAAGGGATGCT	NO
29	*FN1*	2335	2	216241226	216241226	NM_212474:exon34:c.T5339C	TGAGCCAAGCAGGCACTAAT	AGCCCGTTTACATTGTGGGTAT	YES
30	*GPR125*	166647	4	22475411	22475411	NM_145290:exon2:c.A313G	TAGCTGGGAATTTAGTCACAGATTT	ATCCGAGCTGAAGAATGGCTC	YES
31	*GUCY1B3*	2983	4	156696170	156696170	NM_000857:exon3:c.A128T	TGGCAGGTATTGTATTGAGCAGT	CCACTTCTGTAACCCTGCGT	YES
32	*HCAR3*	8843	12	123200527	123200527	NM_006018:exon1:c.A758G	CACCGGAGTTGGCGATTAAC	AGTTGCTGATCCAGAATGGCAC	YES
33	*HSD3B1*	3283	1	120056673	120056673	NM_000862:exon4:c.A527C	TGGCTGTAGTACGACCAAATCT	AACTGTCCTCGGATGCTTGG	YES
34	*IGSF3*	3321	1	117146423	117146423	NM_001007237:exon6:c.G1447A	ATGCCAGCGTCATCCTTGAG	ACCGCTCACTCACCAAGAG	YES

35	*IL17REL*	400935	22	50439514	50439514	NM_001001694:exon4:c.C106A	CAGTCTTCGGGACTTGGGTG	CACAAAGCCTTTCCGAGCAC	YES
36	*IQCC*	55721	1	32671899	32671899	NM_001160042:exon2:c.426+1G>T	GCCAACCTCTGGAGATACCG	GCAGACAAGTTGGTGAGGGT	YES
37	*ITIH3*	3699	3	52833448	52833448	NM_002217:exon8:c.A850G	CCCCTAGAGGGTGGGATAGG	TGTGCCACTGGTGCTGATTA	YES
38	*JAKMIP2*	9832	5	147040551	147040551	NM_001282282:exon2:c.C461T	GGTGCTGGTGACAGAACTCA	ACTGTACTAACCAGCCTCCGA	YES
39	*KANK1*	23189	9	740903	740903	NM_153186:exon8:c.G3191A	CATCCCTTCAGTGGCTTCGT	CTGGTTACGGGTGCATTCCT	YES
40	*KCNMB2*	10242	3	178546013	178546013	NM_001278911:exon4:c.C275T	GCTGGCAAAGGGGAACTTCA	TAGCTCTTGGGCCTGTGTTT	YES
41	*KLHL38*	340359	8	124659205	124659205	NM_001081675:exon2:c.T1400C	CGCTCTTCTGGTTGGTTGGA	TGAGTTCCTGGGGGTCACAA	YES
42	*KRTAP9-8*	83901	17	39394482	39394482	NM_031963:exon1:c.T179C	TGATGATGAGACGGGCTTCCT	TGGGGTGGTAGCAGGTTCT	YES
43	*LINGO1*	84894	15	77907582	77907582	NM_032808:exon2:c.C667T	CTCAATGGTGCTGATGGGGT	CTGAAGCTCATCCCGCTAGG	YES
44	*LOC100996693*	100996693	2	220361598	220361598	NM_001286811:exon2:c.A196G	TCCTCTTCCCTACCAAGCCA	TAGGCCCAGAGGTAAACACG	YES
45	*LOC101929057*	101929057	6	90383952	90383952	NM_014611:exon79:c.T13118C	ACCGACGAGTTCCCTAGGTT	AAGCCCATGCTTGTTCAGAAAC	YES
46	*LOC401052*	401052	3	10049098	10049098	NM_001008737:exon4:c.A287C	TGCACACCCTAGTTTAGGCG	GGAACTCCAGGACTGACCCT	NO
47	*LOC401052*	401052	3	10049098	10049098	NM_001008737:exon4:c.A287C	TGCACACCCTAGTTTAGGCG	GGAACTCCAGGACTGACCCT	YES
48	*MACROD2*	140733	20	16025281	16025281	NM_001033087:exon13:c.A592T	ATCCTCTACCCCCACCAGTAAA	CTGTGGGGACTTGGCAAAAC	YES
49	*MPO*	4353	17	56349062	56349062	NM_000250:exon11:c.G1984A	CTCCCTCCAGTCCTTCAACTG	TGATGGAGCAGTATGGCACG	YES
50	*MYADM*	91663	19	54377469	54377469	NM_001020818:exon2:c.C686G	GCTATATGGCCACCGTACCC	GGCACACACGTAGTAGGCAT	YES
51	*NBEAL2*	23218	3	47037954	47037954	NM_015175:exon16:c.A2345C	CCTGACTTTAGCCACTGGGG	AGTAGCACAGTCGAGCCAAC	YES
52	*NHS*	4810	X	17744510	17744510	NM_001291868:exon6:c.T1690G	AGATGCTTGGTCTTCCCTGC	TACACCACCACCATGATGCC	YES
53	*NHSL1*	57224	6	138794475	138794475	NM_001144060:exon3:c.T307C	CATACAACTGAGTCCTTGGTCAT	ACTCTCAGGGACCCACCTTT	YES
54	*OR2L13*	284521	1	248112794	248112794	NM_001001963:exon1:c.G635C	AGTCCTGAGACCTTAGGCAGAA	TGTGATGTCCCAGCAATGGT	YES
55	*OR51A2*	401667	11	4976013	4976013	NM_001004748:exon1:c.T931C	TCATCAACCTGGCCGTTGTC	ACCAGACATTTCCAGGTTTTCTGT	NO
56	*OTOG*	340990	11	17618539	17618539	NM_001277269:exon29:c.A3703C	ACCATGGAGTTCTCGCTTCC	GACAGACTGCCAGTACCCCT	NO
57	*PCDHA8*	56140	5	140228918	140228918	NM_014005:exon1:c.T838A	AGAACGCCCTGCTCACTTAC	GTTGGCATCTGCGTCTAGGT	YES
58	*PGM1*	5236	1	64117428	64117428	NM_001172818:exon9:c.A1423G	AGGCCTTGGTGTGATCTGC	GTCTCTCTCTCACTTACACACCG	YES
59	*PLEKHS1*	79949	10	115534596	115534596	NM_001193435:exon8:c.A527G	CTTCCCACAGAAAACCACCG	GCTCTTAGGGCCCATCAAGTTT	YES
60	*RBMS3*	27303	3	29628700	29628700	NM_001003792:exon4:c.396+4A>C	CAAACTCCACGTTTGAGTGAGC	TTTTGACAGTCCTGCAGCCG	NO
61	*REPS2*	9185	X	17153504	17153504	NM_001080975:exon16:c.A1780G	GGCTAAAGGCATGTGGGACA	AGCGAGATTCCTACCTCACTCT	YES
62	*SLC1A1*	6505	9	4576117	4576117	NM_004170:exon9:c.C992A	GGAGGAAGGGGGATGCCTAT	AAACCGCAAGCTGGAGTCTG	YES
63	*SPEG*	10290	2	220350190	220350190	NM_005876:exon31:c.A7732T	GGGGTTCCTTCTGGTTCTCTG	CTAGGTTTGTCAAGGTGGCCC	YES
64	*TENM1*	10178	X	123630915	123630915	NM_001163278:exon20:c.G3646T	ACTCAGTGCTACAATAGGGGC	CCCAGCCCACAACAACAAAC	YES
65	*TET2*	54790	4	106156909	106156909	NM_001127208:exon3:c.C1810T	GCTGCTGTTGCTGGTTTTGA	CACCAAGCGGAATCCCATCT	YES
66	*TET2*	54790	4	106157290	106157290	NM_001127208:exon3:c.C2191T	AATCAAGGGCAGTCCCAAGG	GCTGTTGCTGGTTTTGAGGG	YES
67	*TET2*	54790	4	106197207	106197207	NM_001127208:exon11:c.G5540A	TCACGGACATGGTCCTTTCG	CTGGTGCAGAGGACAACGAT	YES
68	*TLL1*	7092	4	166996054	166996054	NM_012464:exon17:c.A2213T	TGACATTTGGATAGCCCACCA	TGTTGGGAACAGGAGAAACCAT	YES
69	*TLR4*	7099	9	120475863	120475863	NM_138557:exon2:c.A857T	TGAGGACCGACACACCAATG	TGGCTTGTCCAGTCTCGAAG	YES
70	*USP24*	23358	1	55559689	55559689	NM_015306:exon52:c.T6275A	ACGATGTAGCCCCAGTGGTA	CCCCATAGGCCGAACAATGA	YES
71	*USP34*	9736	2	61575224	61575224	NM_014709:exon15:c.T2066C	CCTGGCAGTGAGGTACAGTC	TTATTTTGAAGTAGGCTTACCCATC	YES

72	*ZBBX*	79740	3	167023579	167023579	NM_001199201:exon17:c.T1577G	ATGTTCTCTCCCGTATGAGTCAC	TGAGGAAAGCACCTCCTTTGA	YES
73	*ZDHHC14*	79683	6	158093930	158093930	NM_024630:exon9:c.A1243G	CTCTCCCAATGCACCTGGAC	TCATCTTTCCGGGGCATCAC	YES
74	*ZNF326*	284695	1	90470688	90470688	NM_182975:exon4:c.98-4A>G	GCTTCTCATCTTTGTGTTTTTCCCT	CACCACCACTGTGATTGTCCA	YES
75	*ZNF345*	25850	19	37368669	37368669	NM_001242472:exon3:c.C937T	TCCCATAAGCCTTCCCACAG	GGCTCAGATCTCACTCAGCA	NO
76	*ZNF681*	148213	19	23926688	23926688	NM_138286:exon4:c.T1664C	CCTGTGCAATAAGGTGTGAGC	GGCAAAGCTTTCTATCGATCC	YES

*Based on NCBI human reference genome GRC Build 37 (hg19).

Supplementary Table 5. List of 221 selected genes for the targeted sequencing.

ABCA2	*DNAH5*	*GRM6*	*NOTCH3*	*SMC4*
ABCC4	*DNMT1*	*HDAC4*	*NOTCH4*	*SOCS3*
ACAN	*DOCK8*	*HDAC5*	*NRAS*	*SORBS1*
ADAM21	*DROSHA*	*HDAC6*	*NRXN1*	*SPINK5*
AKAP13	*DSPP*	*HDAC9*	*NSD1*	*STAB1*
ALK	*DUSP27*	*HLA-B*	*OVGP1*	*STAT1*
ANKRD11	*DVL2*	*HLA-DQB2*	*PABPC1*	*STAT2*
ANKRD22	*ENDOG*	*HRNR*	*PABPC3*	*STAT3*
AOAH	*EP300*	*IGFN1*	*PABPC4*	*STAT4*
ARHGAP22	*ERBB2*	*IL17REL*	*PAK2*	*STAT5A*
ARHGEF10	*ERCC1*	*IL22RA1*	*PCDH15*	*STAT5B*
ARHGEF11	*ETV1*	*IL2RB*	*PDE4DIP*	*STAT6*
ARHGEF15	*FAM221B*	*IL31RA*	*PDGFRB*	*SYNE1*
ARID1A	*FAT1*	*IL6*	*PDK1*	*TAP1*
ARID1B	*FAT3*	*IL7R*	*PLCB1*	*TBC1D26*
ARID3A	*FAT4*	*ITGA8*	*PLCB2*	*TET2*
ASXL1	*FGFR1*	*JAK1*	*PLCB3*	*TGFBR2*
ATR	*FGFR2*	*JAK2*	*PRDM2*	*TLR4*
ATRX	*FGFR4*	*JAK3*	*PRRC2A*	*TMEM14B*
AXIN2	*FN1*	*JMJD1C*	*PTEN*	*TP53*
BAGE2	*FOXA1*	*KALRN*	*PTPN1*	*TRAM1L1*
BAI1	*FRY*	*KIF7*	*PTPN13*	*TYK2*
BAI3	*FSHR*	*KIR2DL1*	*PTPN14*	*UGT2A1*
BCOR	*FZD1*	*KMT2C*	*PTPN2*	*USP8*
BRCA1	*FZD10*	*KMT2D*	*PTPN3*	*WASF1*
CASP9	*GAREM*	*KRAS*	*PTPN4*	*XRCC1*
CCL23	*GATA3*	*KRT3*	*PTPRC*	*YAP1*
CCNB2	*GNA11*	*LEF1*	*PTPRF*	*ZFHX3*
CD93	*GNA12*	*LHCGR*	*PTPRG*	*ZNF264*
CDC27	*GNA13*	*LILRB3*	*PTPRM*	
CDH3	*GNA14*	*LRP1B*	*PTPRN2*	
CDKN2A	*GNA15*	*MAPK1*	*PTPRQ*	
CELSR2	*GNAI1*	*MGAT4C*	*PTPRR*	
CELSR3	*GNAI2*	*MLH1*	*PTPRS*	
CEP164	*GNAI3*	*MPL*	*PTPRT*	
CHD2	*GNAL*	*MROH2A*	*PTPRZ1*	
CNTROB	*GNAO1*	*MSH6*	*RAI1*	
CSMD1	*GNAQ*	*MSN*	*RB1*	
CSMD2	*GNAS*	*MUC21*	*REST*	
CSMD3	*GNAT1*	*MUC4*	*RETSAT*	
CYP24A1	*GNAT2*	*MUC6*	*RHOA*	

CYP4A11	*GPAT2*	*MUC7*	*RRM1*
DAPK1	*GPATCH4*	*NCKAP5*	*RUNX1T1*
DDX3X	*GPR112*	*NEK1*	*RYR2*
DHRS4L1	*GPR125*	*NFKBIA*	*RYR3*
DICER1	*GPR98*	*NLRP3*	*SETD2*
DNAH11	*GRIN2A*	*NOTCH1*	*SF3B3*
DNAH17	*GRM3*	*NOTCH2*	*SH3RF2*

Supplementary Table 6. Summary of targeted sequencing data of the 73 cases of NKTCL.

Patient ID	Average sequencing depth on target	Coverage of target region*	Fraction of target covered with at least 50×	Fraction of target covered with at least 100×
1	1149	100.00%	99.83%	99.48%
3	1181	100.00%	99.84%	99.61%
4	1232	100.00%	99.60%	98.97%
6	1177	100.00%	99.62%	99.12%
7	1195	100.00%	99.80%	99.47%
9	1559	100.00%	99.84%	99.55%
13	1528	100.00%	99.66%	99.27%
14	1157	100.00%	99.60%	99.06%
15	1481	100.00%	99.64%	99.19%
16	1349	100.00%	99.96%	99.87%
18	1416	100.00%	99.80%	99.49%
20	1102	100.00%	99.38%	98.58%
21	1149	100.00%	99.72%	99.31%
22	1124	99.99%	99.54%	99.17%
24	1161	100.00%	99.76%	99.41%
25	1036	99.99%	99.31%	98.41%
26	1652	100.00%	99.83%	99.53%
27	1428	99.99%	99.88%	99.70%
29	1322	100.00%	99.64%	99.25%
30	1184	100.00%	99.74%	99.35%
32	2290	100.00%	99.84%	99.59%
33	1087	100.00%	99.50%	98.83%
34	1856	100.00%	99.84%	99.63%
35	1158	100.00%	99.59%	99.06%
36	1961	100.00%	99.85%	99.55%
37	1187	100.00%	99.76%	99.35%
38	1157	100.00%	99.69%	99.17%
39	1169	100.00%	99.75%	99.36%
40	1506	100.00%	99.85%	99.60%
41	1099	100.00%	99.45%	98.60%
43	1498	100.00%	99.61%	99.13%
45	1685	100.00%	99.62%	99.15%
46	1116	100.00%	99.82%	99.53%
47	1354	100.00%	99.67%	99.11%
48	1444	100.00%	99.61%	99.07%
49	1797	100.00%	99.92%	99.77%
50	1234	100.00%	99.57%	99.08%
51	1206	100.00%	99.82%	99.55%
54	1351	100.00%	99.38%	98.47%
57	1266	99.99%	99.55%	98.99%
60	1047	99.98%	99.40%	98.56%

61	1165	100.00%	99.72%	99.27%
63	1456	99.99%	99.76%	99.43%
64	1566	100.00%	99.52%	98.90%
67	2615	100.00%	99.88%	99.66%
68	1575	100.00%	99.79%	99.39%
69	1196	100.00%	99.41%	98.61%
70	1254	100.00%	99.69%	99.26%
72	1283	100.00%	99.71%	99.10%
76	1149	100.00%	99.62%	99.05%
81	1852	99.99%	99.53%	99.05%
86	1102	100.00%	99.71%	98.72%
90	1129	100.00%	99.74%	99.38%
91	1176	100.00%	99.81%	99.51%
94	1873	100.00%	99.91%	99.68%
97	1308	99.99%	99.71%	99.41%
98	1544	100.00%	99.80%	99.55%
100	1077	100.00%	99.56%	99.14%
102	1164	100.00%	99.82%	99.52%
104	1379	100.00%	99.82%	99.54%
105	1279	100.00%	99.93%	99.75%
107	1296	100.00%	99.89%	99.72%
109	3352	100.00%	99.95%	99.83%
110	1617	100.00%	99.74%	99.40%
111	1011	100.00%	99.61%	99.10%
112	2271	100.00%	99.85%	99.59%
113	1401	100.00%	99.54%	99.07%
114	1612	100.00%	99.80%	99.48%
115	1128	100.00%	99.78%	99.45%
118	1395	100.00%	99.60%	99.12%
120	1726	99.99%	99.57%	99.10%
121	2032	100.00%	99.77%	99.44%
122	1416	100.00%	99.64%	99.25%
N1	1170	100.00%	99.76%	99.40%
N100	1216	100.00%	99.71%	99.36%
N102	1191	100.00%	99.78%	99.41%
N104	1264	100.00%	99.73%	99.29%
N105	1373	100.00%	99.78%	99.46%
N107	1433	100.00%	99.87%	99.62%
N109	1608	99.99%	99.74%	99.37%
N110	1185	99.99%	99.75%	99.37%
N111	1185	100.00%	99.69%	99.28%
N112	1168	100.00%	99.80%	99.47%
N113	1190	99.99%	99.67%	99.21%
N114	1470	100.00%	99.89%	99.60%
N115	1974	100.00%	99.89%	99.62%
N118	1480	100.00%	99.63%	99.16%

N120	1683	100.00%	99.77%	99.47%
N121	1531	100.00%	99.70%	99.31%
N122	1839	100.00%	99.78%	99.48%
N13	1710	100.00%	99.87%	99.54%
N14	1173	99.99%	99.78%	99.40%
N15	1395	100.00%	99.89%	99.54%
N16	1196	99.99%	99.88%	99.59%
N18	1163	99.99%	99.82%	99.49%
N20	1393	100.00%	99.76%	99.40%
N21	1203	100.00%	99.90%	99.63%
N22	1194	100.00%	99.86%	99.61%
N24	1496	100.00%	99.93%	99.65%
N25	1218	100.00%	99.85%	99.55%
N26	1206	100.00%	99.93%	99.76%
N27	1335	99.99%	99.82%	99.56%
N29	1296	100.00%	99.87%	99.60%
N3	1158	100.00%	99.84%	99.48%
N30	1243	100.00%	99.84%	99.46%
N32	1167	100.00%	99.80%	99.41%
N33	1287	100.00%	99.88%	99.59%
N34	1101	99.99%	99.79%	99.35%
N35	1271	100.00%	99.89%	99.58%
N36	1282	100.00%	99.77%	99.39%
N37	1247	99.99%	99.86%	99.54%
N38	1458	100.00%	99.92%	99.72%
N39	1233	100.00%	99.82%	99.51%
N4	1146	100.00%	99.86%	99.53%
N40	1560	100.00%	99.90%	99.62%
N41	1327	99.99%	99.83%	99.48%
N42	1195	99.99%	99.70%	99.22%
N43	1247	100.00%	99.71%	99.34%
N45	1865	100.00%	99.75%	99.42%
N46	1456	100.00%	99.83%	99.50%
N47	1331	100.00%	99.90%	99.66%
N48	1377	100.00%	99.71%	99.32%
N49	1281	100.00%	99.72%	99.25%
N50	1729	100.00%	99.81%	99.48%
N51	1895	100.00%	99.89%	99.66%
N54	1408	100.00%	99.75%	99.41%
N55	1577	100.00%	99.81%	99.46%
N57	1572	100.00%	99.77%	99.43%
N6	1260	100.00%	99.85%	99.48%
N60	1324	100.00%	99.90%	99.67%
N61	1266	99.99%	99.84%	99.54%
N63	1280	100.00%	99.73%	99.30%
N64	1458	100.00%	99.83%	99.47%

N65	1933	99.99%	99.94%	99.78%
N67	1393	100.00%	99.86%	99.50%
N68	1360	100.00%	99.86%	99.53%
N69	1225	99.99%	99.67%	99.20%
N7	1228	100.00%	99.79%	99.43%
N70	1938	100.00%	99.93%	99.74%
N72	1380	100.00%	99.88%	99.60%
N9	1778	99.99%	99.76%	99.42%
N90	1639	100.00%	99.85%	99.53%
N91	1343	100.00%	99.89%	99.66%
N94	1186	100.00%	99.93%	99.72%
N97	1688	99.99%	99.80%	99.49%
N98	2093	100.00%	99.82%	99.50%

*Based on NCBI human reference genome GRC Build 37 (hg19).

Supplementary Table 7. List of somatic non-silent mutations identified by targeted deep sequencing of 73 cases of NKTCL.

NO	Patient ID	Hugo Symbol	Entrez Gene Id	Chromosome	Position*	Variant Classification	Reference Allele	Tumor Allele	Variant allele frequency	Protein Change
1	1	*C21orf33*	8209	21	45563185	Missense	C	T	0.333	p.A176V
2	1	*ZNF473*	25888	19	50550045	Missense	G	T	0.133	p.G782V
3	1	*GNAI2*	2771	3	50294992	Frame_Shift_Ins	A	ATGATACC	0.02	p.K232fs
4	1	*NOTCH3*	4854	19	15288858	Missense	C	A	0.105	p.R1294L
5	3	*DNAH17*	8632	17	76449472	Missense	G	C	0.196	p.D3494E
6	3	*NRXN1*	9378	2	50699451	Missense	C	T	0.339	p.E1077K
7	3	*LRP1B*	53353	2	141114025	Nonsense	C	A	0.312	p.E3806X
8	3	*ZNF239*	8187	10	44052630	Missense	A	G	0.347	p.F300L
9	3	*PTPRZ1*	5803	7	121681026	Missense	C	A	0.171	p.H1065N
10	3	*FN1*	2335	2	216241226	Missense	A	G	0.297	p.I1780T
11	3	*FOXA1*	3169	14	38061604	Missense	G	T	0.298	p.P129T
12	3	*CD93*	22918	20	23065638	Nonsense	G	A	0.331	p.Q398X
13	3	*ZNF585B*	92285	19	37676424	Missense	C	T	0.087	p.R672Q
14	3	*DNAH5*	1767	5	13913926	Missense	T	C	0.314	p.T488A
15	3	*ADAM20*	8748	14	70989955	Missense	G	A	0.061	p.T557M
16	3	*TET2*	54790	4	106197207	Nonsense	G	A	0.683	p.W1847X
17	3	*KMT2D*	8085	12	49437567	Splice_Site	T	A	0.296	c.5320-2A>T
18	4	*FGFR2*	2263	10	123278280	Missense	C	T	0.352	p.A335T
19	4	*CREBBP*	1387	16	3779700	Missense	C	T	0.1	p.C1745Y
20	4	*ANKRD11*	29123	16	89350300	Missense	C	T	0.387	p.D884N
21	4	*GNAZ*	2781	22	23438591	Missense	G	A	0.091	p.E237K
22	4	*KRT77*	374454	12	53086212	Missense	C	T	0.1	p.E474K
23	4	*LHCGR*	3973	2	48915492	Missense	G	A	0.357	p.H482Y
24	4	*KIR3DL2*	3812	19	55377319	Missense	C	A	0.376	p.L337I
25	4	*TET2*	54790	4	106156909	Nonsense	C	T	0.783	p.Q604X
26	4	*MROH2A*	339766	2	234704914	Missense	C	T	0.041	p.R406C
27	4	*CSMD3*	114788	8	113318391	Missense	G	C	0.349	p.T2535R
28	6	*ZNF585A*	199704	19	37642891	Missense	G	A	0.091	p.A637V
29	6	*GAREM*	64762	18	29847959	Missense	C	T	0.176	p.E836K
30	6	*NCOA6*	23054	20	33345826	Missense	G	T	0.086	p.P242Q
31	7	*ZNF184*	7738	6	27419310	Missense	G	T	0.058	p.D676E
32	7	*ENDOG*	2021	9	131581150	Nonsense	G	T	0.071	p.E63X
33	7	*JMJD1C*	221037	10	65140170	Missense	A	C	0.095	p.F81V
34	7	*ZNF250*	58500	8	146107496	Missense	C	T	0.099	p.G358R
35	7	*BAI3*	577	6	70071146	Missense	A	T	0.087	p.K1327N
36	7	*KMT2D*	8085	12	49424057	Frame_Shift_Del	CAGTGCCCT	A	0.058	p.R4659fs
37	7	*PLCB3*	5331	11	64029509	Missense	G	A	0.055	p.V600I
38	9	*MAPK1*	5594	22	22127164	Missense	C	T	0.117	p.E322K
39	13	*DAPK1*	1612	9	90258316	Missense	G	A	0.225	p.C315Y
40	13	*NOTCH3*	4854	19	15285162	Missense	C	A	0.043	p.D1485Y
41	13	*DNAH9*	1770	17	11833313	Missense	T	C	0.133	p.I315T
42	13	*GRIN2D*	2906	19	48923009	Missense	C	A	0.5	p.L677M
43	13	*TLR4*	7099	9	120475863	Missense	A	T	0.236	p.N286I
44	13	*FGFR4*	2264	5	176522367	Missense	C	T	0.303	p.S479L
45	13	*KALRN*	8997	3	123987830	Missense	A	C	0.25	p.T231P
46	13	*FRY*	10129	13	32808741	Missense	T	A	0.255	p.V1853D
47	13	*FAT1*	2195	4	187629624	Missense	A	G	0.275	p.V453A
48	14	*MAPK1*	5594	22	22221708	In_Frame_Del	CCCGCCG	C	0.047	p.6_8del
49	14	*LEF1*	51176	4	108969874	Missense	G	T	0.139	p.A372D
50	14	*PDE4DIP*	9659	1	144906196	Missense	C	T	0.183	p.D976N
51	14	*CSMD1*	64478	8	2944708	Missense	C	A	0.143	p.G2462V
52	14	*ZKSCAN1*	7586	7	99631581	Missense	G	A	0.083	p.G272R
53	14	*DDX3X*	1654	X	41196704	Frame_Shift_Del	TACAGCCAC	G	0.353	p.G30fs
54	14	*FZD5*	7855	2	208631822	Missense	C	A	0.167	p.G548W
55	14	*PTPN13*	5783	4	87671727	Nonsense	A	T	0.171	p.K919X
56	14	*UGT2A1*	10941	4	70513350	Missense	G	A	0.164	p.L5F
57	14	*ARID1A*	8289	1	27099361	Nonsense	C	T	0.145	p.Q1200X
58	14	*ATRX*	546	X	76953110	Missense	G	A	0.486	p.S68L
59	15	*SYNE1*	23345	6	152763329	Missense	C	T	0.586	p.A1304T
60	15	*KALRN*	8997	3	123987945	Missense	C	T	0.397	p.P269L
61	15	*HDAC9*	9734	7	18688277	Missense	C	A	0.329	p.Q400K
62	15	*ASXL1*	171023	20	31021635	Frame_Shift_Ins	G	GT	0.315	p.R545fs
63	15	*POU4F2*	5458	4	147560497	Nonsense	C	T	0.385	p.R69X
64	15	*KALRN*	8997	3	124132413	Missense	C	T	0.362	p.R813C
65	15	*NFKBIA*	4792	14	35872949	Missense	G	T	0.105	p.R95S
66	15	*PTPRG*	5793	3	62204600	Missense	T	A	0.351	p.V744E
67	16	*GNAS*	2778	20	57470686	In_Frame_Del	AAGCACC	A	0.153	p.54_55del
68	16	*GRM3*	2913	7	86415913	Missense	G	A	0.114	p.A269T
69	16	*NOTCH2*	4853	1	120480552	Missense	A	G	0.164	p.C1089R
70	16	*KMT2D*	8085	12	49420655	Missense	A	G	0.104	p.C5032R
71	16	*CDH3*	1001	16	68725767	Missense	G	T	0.118	p.C647F

72	16	*HDAC4*	9759	2	240111555	Missense	C	T	0.027	p.E105K
73	16	*SMC4*	10051	3	160150844	Missense	G	T	0.177	p.K1187N
74	16	*KMT2D*	8085	12	49433256	Nonsense	G	A	0.117	p.Q2731X
75	16	*SYNE1*	23345	6	152787212	Missense	G	T	0.136	p.S552Y
76	16	*ERBB2*	2064	17	37876044	Missense	G	T	0.134	p.V635L
77	16	*ATRX*	546	X	76937942	Missense	C	T	0.026	p.V898I
78	16	*R3HCC1L*	27291	10	99922734	Splice_Site	T	C	0.091	–
79	18	*GPR98*	84059	5	90049445	Missense	G	T	0.329	p.D3726Y
80	18	*PTPRK*	5796	6	128304509	Nonsense	G	A	0.08	p.R1089X
81	18	*DNAH5*	1767	5	13727663	Missense	G	A	0.472	p.R3996C
82	18	*ARHGAP22*	58504	10	49667878	Missense	G	A	0.341	p.R80C
83	20	*DAPK1*	1612	9	90322148	Frame_Shift_Del	GC	G	0.349	p.A1388fs
84	20	*PTPRN2*	5799	7	157475615	Missense	C	G	0.31	p.K584N
85	20	*ZNF354B*	117608	5	178310466	Missense	G	A	0.125	p.S338N
86	21	*DDX3X*	1654	X	41205655	Missense	G	A	0.583	p.A481T
87	21	*ADAM20*	8748	14	70990018	Missense	G	A	0.214	p.P536L
88	21	*TFEB*	7942	6	41658930	Missense	G	A	0.167	p.R22C
89	21	*MSN*	4478	X	64959601	Nonsense	C	A	0.54	p.S527X
90	21	*LRP1B*	53353	2	141092049	Missense	C	A	0.254	p.V4066L
91	21	*STAT3*	6774	17	40474482	Missense	T	A	0.42	p.Y640F
92	22	*CCND1*	595	11	69462827	Missense	G	A	0.08	p.G214S
93	24	*ZFP1*	162239	16	75204081	Missense	G	A	0.25	p.G358D
94	24	*GPR125*	166647	4	22463415	Missense	G	C	0.2	p.L116V
95	24	*AMOT*	154796	X	112058834	Nonsense	G	A	0.133	p.Q382X
96	24	*CSMD3*	114788	8	113318320	Nonsense	G	A	0.15	p.R2559X
97	25	*DROSHA*	29102	5	31405796	Missense	C	T	0.077	p.A1291T
98	25	*DSPP*	1834	4	88535832	In_Frame_Ins	A	[illegible]GTGACAG[illegible]	0.062	[illegible]673delinsDSSDSSS
99	25	*BAI1*	575	8	143625693	Missense	A	G	0.25	p.E1557G
100	25	*ASXL1*	171023	20	31023163	Missense	A	G	0.144	p.E883G
101	25	*KMT2C*	58508	7	151875018	Missense	C	T	0.068	p.G2507E
102	25	*RYR2*	6262	1	237905623	Missense	G	A	0.056	p.G3707S
103	25	*ZFHX3*	463	16	72992201	Missense	C	T	0.048	p.G615D
104	25	*STAB1*	23166	3	52542344	Missense	G	A	0.06	p.G735D
105	25	*FLG2*	388698	1	152327548	Missense	C	T	0.25	p.G905E
106	25	*ANKRD11*	29123	16	89357429	Missense	T	C	0.158	p.N130S
107	25	*CSMD1*	64478	8	2954496	Missense	G	T	0.068	p.P2338Q
108	25	*SF3B3*	23450	16	70599104	Missense	G	A	0.191	p.R867Q
109	25	*CSMD3*	114788	8	113277741	Missense	G	A	0.063	p.S3027F
110	25	*ARID1A*	8289	1	27106891	Missense	G	A	0.048	p.V2168M
111	26	*GRM6*	2916	5	178419057	Missense	C	A	0.152	p.A178S
112	26	*RYR2*	6262	1	237819173	Missense	C	A	0.16	p.A2673E
113	26	*FSHR*	2492	2	49381414	Missense	G	T	0.166	p.A48D
114	26	*CELSR1*	9620	22	46806417	Missense	G	T	0.154	p.P1604H
115	26	*DNAH17*	8632	17	76422617	Missense	G	A	0.166	p.P4279L
116	26	*NRXN1*	9378	2	50149175	Missense	C	A	0.118	p.Q412H
117	26	*HLA-A*	3105	6	29911113	Frame_Shift_Ins	C	CG	0.111	p.R138fs
118	26	*CSMD2*	114784	1	34204872	Missense	C	T	0.159	p.S746N
119	27	*DDX3X*	1654	X	41205613	Missense	G	A	0.651	p.A467T
120	27	*CSMD3*	114788	8	113363409	Missense	C	A	0.22	p.C2003F
121	27	*ZSCAN16*	80345	6	28097571	Missense	G	A	0.333	p.C297Y
122	27	*KCTD17*	79734	22	37458621	Missense	G	A	1	p.G294E
123	27	*NOTCH3*	4854	19	15289953	Missense	G	A	0.211	p.R1201C
124	29	*MSN*	4478	X	64958992	Frame_Shift_Ins	A	AC	0.691	p.D502fs
125	29	*KRT5*	3852	12	52913745	In_Frame_Ins	A	ACCG	0.568	p.G112delinsGG
126	29	*DDX3X*	1654	X	41204711	Missense	G	A	0.768	p.G393S
127	29	*IL17REL*	400935	22	50439514	Missense	G	T	0.354	p.H36N
128	29	*GAREM*	64762	18	29867745	Missense	T	C	0.036	p.K272R
129	29	*FZD1*	8321	7	90895013	Missense	A	C	0.281	p.K273T
130	29	*DVL2*	1856	17	7129551	Missense	T	C	0.052	p.K615R
131	29	*TRAM1L1*	133022	4	118005907	Missense	A	C	0.325	p.L215V
132	29	*PTPN14*	5784	1	214558072	Missense	A	C	0.311	p.L376V
133	29	*ST6GALNAC5*	81849	1	77334276	Missense	C	A	0.154	p.P37Q
134	29	*TET2*	54790	4	106157290	Nonsense	C	T	0.612	p.Q731X
135	29	*GPR125*	166647	4	22475411	Missense	T	C	0.286	p.S105G
136	29	*DNAH11*	8701	7	21641193	Missense	G	T	0.35	p.S1202I
137	29	*FAT1*	2195	4	187525580	Nonsense	G	C	0.054	p.S3500X
138	29	*LRP1B*	53353	2	141771191	Missense	T	C	0.329	p.T772A
139	30	*FZD9*	8326	7	72848543	Missense	C	T	0.5	p.A69V
140	30	*ZNF852*	285346	3	44541451	Missense	C	T	0.182	p.C273Y
141	30	*NEK1*	4750	4	170354703	Missense	C	T	0.211	p.E863K
142	30	*MROH2A*	339766	2	234704345	Missense	T	C	0.112	p.V341A
143	30	*RYR1*	6261	19	39063910	Missense	G	A	0.095	p.V4693M
144	30	*ACAN*	176	15	89381891	Splice_Site	C	T	0.186	c.71-3C>T
145	32	*GNAQ*	2776	9	80537112	Missense	T	A	0.16	p.T96S
146	33	*CD93*	22918	20	23066788	In_Frame_Del	GAGCAGC	G	0.021	p.12_14del
147	33	*ZSCAN16*	80345	6	28097385	Missense	G	A	0.105	p.R235Q

148	34	*NCOA3*	8202	20	46279830	In_Frame_Del	GCAGCAA	G	0.313	p.1252_1253del
149	34	*GNA12*	2768	7	2883737	In_Frame_Del	GCCC	G	0.106	p.19_20del
150	34	*ARID1B*	57492	6	157100040	In_Frame_Del	[illegible]AGGAGCAG	G	0.307	p.326_331del
151	34	*PPP1R15A*	23645	19	49377540	In_Frame_Del	AGAG	A	0.426	p.351_351del
152	34	*LMNA*	4000	1	156108372	In_Frame_Del	GCAT	G	1.283	p.568_569del
153	34	*LILRB4*	11006	19	55175799	Missense	C	T	0.427	p.A202V
154	34	*NCKAP5*	344148	2	133547730	Missense	C	T	0.495	p.A320T
155	34	*DNAH17*	8632	17	76570793	Frame_Shift_Del	ACCT	A	0.507	p.E115fs
156	34	*FN1*	2335	2	216226765	In_Frame_Ins	T	TCAG	0.195	p.E2129delinsAE
157	34	*RAD9A*	5883	11	67164815	In_Frame_Ins	G	GGAA	1.04	p.E270delinsEE
158	34	*TP53*	7157	17	7577153	Missense	C	T	0.081	p.G130D
159	34	*DDX3X*	1654	X	41203046	Missense	G	A	0.636	p.G230S
160	34	*PTPRG*	5793	3	61547992	Missense	A	T	0.476	p.I11F
161	34	*FAT3*	120114	11	92534702	Missense	G	A	0.17	p.M2841I
162	34	*PRRC2B*	84726	9	134319671	Missense	T	C	0.5	p.V190A
163	34	*PTPRT*	11122	20	41420091	Missense	A	G	0.494	p.V77A
164	34	*TP53*	7157	17	7578493	Nonsense	C	T	0.116	p.W14X
165	34	*DNAH5*	1767	5	13789026	Splice_Site	G	A	0.503	c.8449-3C>T
166	36	*PCDH15*	65217	10	55617008	Nonsense	G	A	0.135	p.Q1174X
167	36	*PVRL1*	5818	11	119535660	Missense	G	A	0.667	p.R451C
168	36	*GNAQ*	2776	9	80537112	Missense	T	A	0.121	p.T96S
169	37	*GPR98*	84059	5	89990063	Missense	C	G	0.088	p.A2497G
170	37	*CSMD1*	64478	8	3072096	Missense	A	G	0.202	p.L1597P
171	37	*KIF7*	374654	15	90192418	Missense	C	T	0.175	p.R237H
172	37	*GNAS*	2778	20	57430184	Nonsense	C	T	0.165	p.R622X
173	38	*HDGFL1*	154150	6	22570340	Missense	C	T	0.111	p.A179V
174	38	*DDX3X*	1654	X	41202581	Missense	A	C	0.258	p.D203A
175	38	*PTPN3*	5774	9	112166751	Missense	C	T	0.225	p.G312R
176	38	*KAT6B*	23522	10	76781981	Nonsense	A	T	0.167	p.K939X
177	38	*CYP4A11*	1579	1	47398707	Missense	A	T	0.218	p.L412H
178	38	*TET2*	54790	4	106157735	Frame_Shift_Del	TTCACAGG	T	0.144	p.L879fs
179	38	*PTPN13*	5783	4	87692418	Missense	A	G	0.224	p.N1442S
180	38	*LRP1B*	53353	2	141214086	Missense	G	C	0.114	p.P3301A
181	38	*TET2*	54790	4	106196267	Nonsense	C	T	0.249	p.Q1534X
182	38	*ABCA2*	20	9	139915878	Missense	C	T	0.375	p.R288Q
183	38	*ZSCAN22*	342945	19	58850489	Nonsense	C	T	0.274	p.R425X
184	38	*CTCFL*	140690	20	56071319	Missense	C	G	0.364	p.R697P
185	38	*NRXN1*	9378	2	50724787	Nonsense	G	A	0.12	p.R855X
186	39	*STAB1*	23166	3	52535230	Missense	C	T	0.034	p.P43S
187	39	*PTPRS*	5802	19	5208380	Missense	C	T	0.043	p.R1390Q
188	39	*GRM4*	2914	6	34003461	Missense	G	A	0.083	p.S693L
189	40	*AXIN2*	8313	17	63533685	Missense	G	A	0.167	p.A490V
190	40	*MLH1*	4292	3	37092139	Missense	T	C	0.076	p.C687R
191	40	*DDX3X*	1654	X	41205858	Missense	G	C	0.336	p.G517A
192	40	*STAT3*	6774	17	40469200	Missense	G	A	0.039	p.P714L
193	41	*MAPK3*	5595	16	30129057	Missense	C	T	0.059	p.E237K
194	41	*DDX3X*	1654	X	41205827	Frame_Shift_Del	GAA	G	0.228	p.E507fs
195	41	*UGT2B4*	7363	4	70346452	Missense	A	G	0.248	p.F496S
196	41	*CSMD2*	114784	1	34630649	Missense	C	T	0.2	p.G17E
197	41	*TP53*	7157	17	7578217	Missense	G	A	0.351	p.T79I
198	41	*ZNF540*	163255	19	38103907	Missense	G	A	0.154	p.V544I
199	41	*DDX3X*	1654	X	41205833	Nonsense	T	TCGTA	0.239	Y509_V510delinsSX
200	41	*STAT3*	6774	17	40474482	Missense	T	A	0.263	p.Y640F
201	41	*DNAH5*	1767	5	13814713	Splice_Site	C	G	0.571	c.7230+1G>C
202	43	*CSMD3*	114788	8	113246646	Missense	G	A	0.063	p.A3394V
203	43	*SUPT20HL1*	100130302	X	24382339	Missense	C	T	0.091	p.P488S
204	43	*PCDH15*	65217	10	55779984	Missense	G	C	0.064	p.P836A
205	45	*ACAN*	176	15	89401424	Missense	G	T	0.25	p.G1870W
206	45	*MSN*	4478	X	64949397	Frame_Shift_Del	TC	T	0.554	p.I97fs
207	45	*ZFHX3*	463	16	72828800	Missense	G	A	0.032	p.P1680L
208	46	*ARID1A*	8289	1	27100181	In_Frame_Del	[illegible]GCAGCAGC[illegible]	C	0.024	p.1326_1329del
209	46	*DVL3*	1857	3	183887884	Missense	C	T	0.143	p.A530V
210	46	*DNAH11*	8701	7	21723558	Missense	G	T	0.23	p.D1873Y
211	46	*PTPRF*	5792	1	44063502	Missense	G	A	0.045	p.D633N
212	46	*STAT3*	6774	17	40474420	Missense	C	A	0.358	p.D661Y
213	46	*ZSCAN22*	342945	19	58850346	Missense	A	G	0.087	p.E377G
214	46	*ARID3A*	1820	19	968489	Missense	G	A	0.046	p.G527D
215	46	*MSN*	4478	X	64959733	Missense	T	G	0.486	p.I571S
216	46	*ARHGEF15*	22899	17	8215701	Missense	G	A	0.041	p.R115Q
217	46	*FAT3*	120114	11	92577287	Missense	C	T	0.188	p.T3585I
218	47	*MSN*	4478	X	64953130	Frame_Shift_Del	CA	C	0.285	p.K262fs
219	47	*DNAH17*	8632	17	76499048	Missense	G	T	0.116	p.L1668I
220	48	*FRMPD3*	84443	X	106846340	Missense	G	T	0.333	p.A1724S
221	48	*KMT2D*	8085	12	49433620	Nonsense	G	A	0.242	p.R2645X
222	48	*C9orf43*	257169	9	116187701	Splice_Site	G	A	0.167	c.942+1G>A
223	48	*HDAC4*	9759	2	240055940	Splice_Site	C	G	0.162	c.1294+1G>C

224	50	*DACH1*	1602	13	72440556	Missense	C	T	0.667	p.G118S
225	50	*FRY*	10129	13	32768341	Missense	A	T	0.043	p.N1218I
226	50	*NOTCH1*	4851	9	139391014	Missense	G	C	0.15	p.Q2393E
227	51	*RUNX2*	860	6	45390486	In_Frame_Del	GCGGCGGC(	A	0.635	p.58_64del
228	51	*DICER1*	23405	14	95599687	Nonsense	T	A	0.389	p.E37X
229	51	*MSN*	4478	X	64959744	Missense	G	A	0.426	p.E575K
230	51	*MICU3*	286097	8	16884984	Missense	G	C	0.5	p.E66Q
231	51	*STAT3*	6774	17	40475058	Missense	C	G	0.466	p.G618R
232	51	*CSMD1*	64478	8	3008996	Missense	A	T	0.033	p.I1985N
233	51	*ZNF624*	57547	17	16527226	Frame_Shift_Ins	A	AT	0.313	p.I325fs
234	51	*FOXF1*	2294	16	86544375	Missense	A	C	0.167	p.K67T
235	51	*PTPN13*	5783	4	87687597	Missense	T	C	0.519	p.L1228P
236	51	*FGFR4*	2264	5	176523107	Missense	A	C	0.469	p.N584T
237	51	*TP53*	7157	17	7577106	Missense	G	A	0.41	p.P146S
238	51	*NOTCH2NL*	388677	1	145273228	Missense	C	A	0.061	p.P28T
239	51	*THAP11*	57215	16	67876814	In_Frame_Ins	G	GCAA	0.397	p.Q119delinsQQ
240	51	*HRNR*	388697	1	152186699	Missense	T	A	0.106	p.Q2469L
241	51	*TBP*	6908	6	170871031	In_Frame_Ins	G	CAACAGCA	0.389	p.Q49delinsQQQQ
242	51	*TBP*	6908	6	170871073	In_Frame_Ins	G	CAACAGCA	0.457	p.Q63delinsQQQQ
243	51	*ABCA4*	24	1	94466425	Missense	C	T	0.091	p.R2149Q
244	51	*CDKN2A*	1029	9	21971133	Missense	G	C	0.448	p.R90G
245	51	*SYNE1*	23345	6	152563496	Missense	T	C	0.68	p.Y6520C
246	51	*BAGE3*	85318	21	11021109	Splice_Site	C	T	0.135	c.1492+1G>A
247	51	*TYK2*	7297	19	10469850	Splice_Site	C	A	0.5	c.2175+1G>T
248	54	*LILRB1*	10859	19	55144626	Missense	A	G	0.063	p.Y373C
249	57	*KMT2C*	58508	7	151893019	Missense	C	T	0.077	p.D1451N
250	60	*ABCA2*	20	9	139907742	Missense	G	T	0.087	p.Q1527K
251	61	*MSN*	4478	X	64953120	Frame_Shift_Ins	A	GCCCATT(	0.47	p.K258fs
252	61	*LRP1B*	53353	2	141747230	Splice_Site	G	T	0.149	c.2645-4C>A
253	63	*FZD10*	11211	12	130648277	Missense	A	T	0.077	p.I264F
254	63	*GNAQ*	2776	9	80537228	Missense	T	G	0.068	p.K57T
255	63	*CDHR5*	53841	11	618619	Missense	C	T	0.125	p.S641N
256	63	*ADAM29*	11086	4	175898117	Missense	G	A	0.19	p.V481M
257	63	*FZD1*	8321	7	90895847	Missense	T	C	0.069	p.V551A
258	63	*HLA-B*	3106	6	31323095	Nonsense	C	T	0.079	p.W298X
259	63	*DDX3X*	1654	X	41206206	Nonsense	G	A	0.307	p.W554X
260	63	*ARID1A*	8289	1	27099299	Splice_Site	G	A	0.08	c.3540-4G>A
261	64	*PDGFRB*	5159	5	149510198	Missense	T	C	0.168	p.E424G
262	67	*SYNE1*	23345	6	152770720	Missense	T	G	0.265	p.K1158T
263	67	*ZNF232*	7775	17	5009298	Missense	G	C	0.075	p.P386A
264	67	*GNAQ*	2776	9	80537112	Missense	T	A	0.182	p.T96S
265	67	*CSMD3*	114788	8	113275867	Splice_Site	C	T	0.271	c.9355+1G>A
266	67	*KMT2D*	8085	12	49419964	Splice_Site	C	G	0.259	c.15784+1G>C
267	68	*DDX3X*	1654	X	41198309	In_Frame_Ins	C	CTAAAAA	0.43	p.H42delinsLKN
268	68	*SYNE1*	23345	6	152712562	Missense	G	C	0.205	p.S2625R
269	68	*DDX3X*	1654	X	41198297	Frame_Shift_Del	ATATTCCTC	T	0.417	p.Y38fs
270	69	*ARID1A*	8289	1	27099101	Frame_Shift_Ins	A	AT	0.306	p.I1173fs
271	69	*BCOR*	54880	X	39934053	Frame_Shift_Del	ATTGATGT	A	0.429	p.N180fs
272	69	*MUC6*	4588	11	1017948	Missense	G	A	0.113	p.P1618L
273	69	*KMT2D*	8085	12	49445525	Frame_Shift_Ins	T	TG	0.187	p.P647fs
274	69	*FZD8*	8325	10	35928686	Missense	G	A	0.2	p.R558C
275	69	*STAT3*	6774	17	40475070	Missense	T	G	0.305	p.S614R
276	70	*RYR2*	6262	1	237656342	Missense	G	A	0.076	p.G639E
277	70	*FAT1*	2195	4	187554945	Missense	T	G	0.066	p.K1406Q
278	70	*CSMD3*	114788	8	113697935	Missense	G	T	0.066	p.P624T
279	70	*DNAH9*	1770	17	11593551	Missense	T	G	0.167	p.V1471G
280	72	*SETD2*	29072	3	47125218	In_Frame_Del	CAACTGTCC	C	0.354	p.2012_2017del
281	72	*PTPRD*	5789	9	8528666	Missense	C	T	0.385	p.E156K
282	72	*SETD2*	29072	3	47103738	Nonsense	G	A	0.417	p.Q2070X
283	72	*CSMD1*	64478	8	3076824	Missense	C	T	0.405	p.R1542Q
284	72	*ZNF80*	7634	3	113955221	Missense	C	T	0.091	p.R234Q
285	72	*ZNF3*	7551	7	99668869	Missense	C	T	0.143	p.R377H
286	76	*ZNF569*	148266	19	37904820	Missense	C	T	0.091	p.C247Y
287	76	*MSN*	4478	X	64951751	Frame_Shift_Del	TG	T	0.741	p.G202fs
288	76	*GRIN2B*	2904	12	13761758	Missense	C	T	0.08	p.G597R
289	76	*RYR1*	6261	19	39025431	Missense	G	A	0.118	p.M3772I
290	76	*BAI1*	575	8	143546345	Splice_Site	T	C	0.051	c.784+2T>C
291	81	*JAK1*	3716	1	65301158	Missense	C	T	0.33	p.G1097D
292	81	*LRP1B*	53353	2	141356340	Missense	T	G	0.156	p.K2352Q
293	81	*DDX3X*	1654	X	41205617	Missense	T	G	0.174	p.L468R
294	81	*ERBB2*	2064	17	37866383	Missense	C	A	0.088	p.P230T
295	81	*KMT2B*	9757	19	36224565	Missense	C	T	0.4	p.P2343S
296	81	*SYNE1*	23345	6	152720850	Missense	G	A	0.142	p.R2387C
297	86	*SOCS3*	9021	17	76355068	Missense	C	T	0.034	p.A37T
298	86	*JAK3*	3718	19	17948006	Missense	G	A	0.45	p.A573V
299	86	*CLCN1*	1180	7	143018557	Missense	T	C	0.125	p.F178S

300	86	*CDH3*	1001	16	68712749	Missense	C	T	0.378	p.H211Y
301	86	*JAK1*	3716	1	65312362	Missense	G	A	0.118	p.L653F
302	86	*ZNF432*	9668	19	52537272	Missense	G	A	0.125	p.R554C
303	86	*BCOR*	54880	X	39932398	Frame_Shift_Del	GGCGT	G	0.817	p.T733fs
304	90	*YEATS2*	55689	3	183493733	Missense	G	A	0.333	p.G800E
305	90	*KALRN*	8997	3	124237269	Nonsense	G	A	0.027	p.W1651X
306	91	*DDX3X*	1654	X	41201996	Frame_Shift_Del	CTT	C	0.174	p.F135fs
307	91	*FZD7*	8324	2	202901079	Missense	G	T	0.08	p.G570V
308	91	*CSMD1*	64478	8	2823398	Missense	C	T	0.062	p.S3060N
309	94	*ZNF432*	9668	19	52537964	Missense	C	G	0.186	p.G323A
310	94	*UGT2B11*	10720	4	70066294	Missense	C	A	0.148	p.W485L
311	97	*KMT2D*	8085	12	49426141	Frame_Shift_Del	TG	T	0.329	p.H4116fs
312	97	*TP53*	7157	17	7579415	Nonsense	C	T	0.372	p.W52X
313	97	*CSMD2*	114784	1	34006729	Splice_Site	C	A	0.04	c.9457+1G>T
314	97	*GPR125*	166647	4	22414802	Splice_Site	T	C	0.25	c.2232+3A>G
315	98	*HLA-A*	3105	6	29911899	Frame_Shift_Ins	A	AC	0.162	p.D207fs
316	100	*DDX3X*	1654	X	41206117	Missense	G	A	0.351	p.A525T
317	100	*GRM8*	2918	7	126173128	Missense	T	G	0.5	p.T770P
318	102	*LRP1B*	53353	2	141773389	Missense	A	C	0.036	p.F689C
319	102	*CSMD3*	114788	8	113317034	Missense	C	T	0.181	p.G2728S
320	102	*ZNF805*	390980	19	57765153	Missense	A	C	0.062	p.K189N
321	102	*MROH2A*	339766	2	234704873	Missense	A	C	0.204	p.K392T
322	102	*ACAN*	176	15	89400771	Missense	T	G	0.037	p.L1652R
323	102	*KRT76*	51350	12	53170504	Missense	T	G	0.036	p.N191T
324	102	*GPR98*	84059	5	89990430	Missense	A	C	0.035	p.Q2619H
325	102	*ZSCAN20*	7579	1	33960132	Nonsense	C	T	0.075	p.R730X
326	102	*ZNF184*	7738	6	27419963	Missense	A	T	0.095	p.S459T
327	102	*SYNE1*	23345	6	152647624	Missense	T	C	0.177	p.S4963G
328	102	*STAT3*	6774	17	40475068	Missense	G	C	0.307	p.S614R
329	102	*EP300*	2033	22	41553188	Missense	G	C	0.209	p.V1093L
330	102	*SYNE1*	23345	6	152647089	Splice_Site	T	A	0.028	c.15225+4A>T
331	104	*NRXN1*	9378	2	50463934	Missense	A	T	0.056	p.L145Q
332	104	*ZNF7*	7553	8	146067666	Missense	C	A	0.125	p.L296I
333	104	*FOXP2*	93986	7	114271582	Splice_Site	G	C	0.182	c.598-4G>C
334	105	*TET2*	54790	4	106197248	Missense	G	A	0.16	p.G1861R
335	105	*ACAN*	176	15	89402028	Missense	G	A	0.183	p.G2071E
336	105	*KRT73*	319101	12	53004551	Missense	C	A	0.167	p.R393S
337	105	*SH3RF2*	153769	5	145317703	Missense	G	A	0.202	p.R71H
338	105	*FAT4*	79633	4	126369618	Missense	T	A	0.18	p.S2483T
339	105	*STAT3*	6774	17	40475068	Missense	G	T	0.177	p.S614R
340	105	*PCDH15*	65217	10	55782668	Missense	G	T	0.188	p.T766N
341	107	*CDHR5*	53841	11	618988	Missense	G	A	0.087	p.A518V
342	107	*KMT2C*	58508	7	151935811	Missense	G	A	0.071	p.A878V
343	107	*DSPP*	1834	4	88535832	In_Frame_Ins	A	GTGACAG	0.409	673delinsDSSDSSS
344	107	*CSMD2*	114784	1	33985509	Missense	G	C	0.357	p.H3403D
345	107	*BAI1*	575	8	143566085	Missense	G	C	0.362	p.M756I
346	107	*TP53*	7157	17	7579416	Frame_Shift_Del	GGAGGGGG	A	0.549	p.P48fs
347	107	*PTPN21*	11099	14	88935394	Nonsense	G	A	0.29	p.R1088X
348	107	*KRT7*	3855	12	52639415	Missense	C	T	0.36	p.R402W
349	107	*NCKAP5*	344148	2	133887622	Missense	C	T	0.301	p.R90Q
350	107	*IL7R*	3575	5	35876286	Missense	A	G	0.467	p.S360G
351	107	*ACAN*	176	15	89401916	Missense	G	T	0.367	p.V2034F
352	107	*FAT1*	2195	4	187539780	Missense	C	T	0.508	p.V2654I
353	107	*ACAN*	176	15	89398757	Missense	G	A	0.401	p.V981I
354	109	*FAT1*	2195	4	187628916	Missense	T	C	0.225	p.Q689R
355	109	*KIF2B*	84643	17	51901323	Missense	C	T	0.2	p.T310M
356	110	*ATRX*	546	X	76764089	Nonsense	G	A	0.916	p.R2369X
357	110	*KMT2D*	8085	12	49434909	Nonsense	G	T	0.493	p.S2215X
358	110	*CSMD1*	64478	8	3474295	Missense	A	T	0.89	p.V345D
359	111	*STAT3*	6774	17	40474420	Missense	C	A	0.067	p.D661Y
360	112	*SYNE1*	23345	6	152646306	Missense	C	G	0.323	p.Q5119H
361	113	*PTPN13*	5783	4	87696369	Missense	A	G	0.026	p.N1661D
362	114	*CSMD3*	114788	8	113933949	Missense	C	T	0.044	p.E410K
363	114	*FAT1*	2195	4	187540421	Missense	A	T	0.321	p.I2440N
364	114	*BRCA1*	672	17	41256207	Missense	T	A	0.057	p.I78F
365	114	*ARHGAP22*	58504	10	49791002	Missense	G	A	0.286	p.P77L
366	115	*RHOA*	387	3	49412973	Missense	C	A	0.196	p.G17V
367	115	*GNAQ*	2776	9	80537112	Missense	T	A	0.164	p.T96S
368	118	*LRP1B*	53353	2	141459812	Missense	A	T	0.118	p.I2067N
369	118	*KALRN*	8997	3	124160810	Missense	C	T	0.088	p.R1071W
370	118	*PAK2*	5062	3	196537469	Missense	G	A	0.108	p.V240M
371	118	*ARID1A*	8289	1	27100390	Splice_Site	G	T	0.121	c.4101+1G>T
372	120	*KALRN*	8997	3	124044872	Missense	C	A	0.443	p.L378I
373	120	*KALRN*	8997	3	124153157	Missense	C	A	0.47	p.L943I
374	120	*KRT6A*	3853	12	52884718	Missense	A	G	0.556	p.M279T
375	120	*JAK3*	3718	19	17945969	Missense	C	T	0.058	p.R657Q

376	120	*JAK3*	3718	19	17945970	Missense	G	A	0.153	p.R657W
377	120	*JAK1*	3716	1	65311203	Missense	C	A	0.227	p.S703I
378	120	*FAT1*	2195	4	187539143	Missense	G	C	0.073	p.T2866R
379	120	*DNAH11*	8701	7	21939024	Missense	G	A	0.876	p.V4374M
380	121	*ARID1A*	8289	1	27094347	Nonsense	G	T	0.265	p.E1019X
381	121	*DDX3X*	1654	X	41206199	Missense	C	T	0.499	p.P552L

*Based on NCBI human reference genome GRC Build 37 (hg19).

Supplementary Table 8. List of non-silent muttions identified by whole exome sequencing of 26 cses of NKTCL without the mtched norml tissue.

Patient ID	Hugo Symbol	Entrez Gene Id	Chromo some	Position*	Variant Classification	Reference Allele	Tumor Allele	Variant allele frequency	Protein Change
143	*COMMD10*	16144	5	115426870	stopgain	C	A	0.158	p.S76X
143	*TAS2R38*	176817	7	141672829	stopgain	C	A	0.053	p.G221X
143	*CRYL1*	15974	13	21086613	stopgain	G	A	0.174	p.Q40X
143	*WDR89*	80666	14	64066363	stopgain	G	A	0.094	p.R100X
143	*PGD*	2631	1	10478930	missense	T	C	0.102	p.L386P
143	*KLF17*	173484	1	44595602	missense	T	G	0.062	p.L220R
143	*KLF17*	173484	1	44595614	missense	C	G	0.076	p.P224R
143	*FLG2*	1014342	1	152323534	missense	C	G	0.089	p.R2243T
143	*PTPN14*	5401	1	214556970	missense	C	T	0.087	p.R743H
143	*HK2*	189	2	75099527	missense	A	G	0.074	p.H159R
143	*SNRNP200*	14014	2	96942874	missense	G	A	0.074	p.R2013C
143	*FER1L5*	1293083	2	97365288	missense	A	G	0.231	p.M1538V
143	*SCN9A*	2977	2	167055838	missense	T	C	0.091	p.T1760A
143	*SCN9A*	2977	2	167056123	missense	T	C	0.114	p.N1665D
143	*SCN9A*	2977	2	167143021	missense	C	G	0.068	p.R476T
143	*ZNF804A*	194250	2	185802216	missense	T	C	0.061	p.I698T
143	*ERBB4*	5235	2	212566763	missense	T	A	0.198	p.N473I
143	*UGT1A1*	463	2	234669360	missense	A	G	0.063	p.S143G
143	*MTMR14*	22485	3	9704010	missense	C	T	0.060	p.A123V
143	*ZNF502*	33210	3	44763301	missense	C	T	0.133	p.T331I
143	*ZNF717*	1290209	3	75788230	missense	C	T	0.080	p.V132I
143	*IGF2BP2*	6548	3	185407149	missense	T	C	0.078	p.Q224R
143	*IGF2BP2*	6548	3	185407152	missense	G	C	0.080	p.T223S
143	*RTP1*	153708	3	186917701	missense	C	T	0.114	p.A212V
143	*BMP2K*	198892	4	79833077	missense	G	A	0.051	p.V1126M
143	*GK2*	33214	4	80328969	missense	T	C	0.064	p.K129R
143	*GK2*	33214	4	80329048	missense	A	T	0.080	p.L103I
143	*WDFY3*	14991	4	85623612	missense	C	A	0.175	p.E2830D
143	*OSTC*	21227	4	109571947	missense	G	A	0.061	p.G46R
143	*PCDH18*	19035	4	138452598	missense	C	A	0.160	p.Q215H
143	*SCOC*	1153663	4	141294788	missense	G	A	0.051	p.R33Q
143	*ANP32C*	12403	4	165118838	missense	G	A	0.087	p.S9L
143	*PRDM9*	20227	5	23521243	missense	T	C	0.143	p.S155P
143	*KCNN2*	170775	5	113822755	missense	T	A	0.190	p.N421K
143	*ZNF354B*	58230	5	178310417	missense	C	G	0.255	p.Q322E
143	*OR2V2*	206880	5	180582175	missense	A	T	0.085	p.N78I
143	*ERVFRD-1*	207582	6	11105377	missense	G	C	0.055	p.T56R
143	*FAM8A1*	16255	6	17601058	missense	G	A	0.100	p.A140T
143	*MUC22*	1198815	6	30995913	missense	A	G	0.057	p.D902G
143	*HLA-DQA2*	20056	6	32713061	missense	C	A	0.054	p.Q70K
143	*COL21A1*	30820	6	56035470	missense	T	C	0.129	p.T335A
143	*KHDC3L*	1017361	6	74073534	missense	T	G	0.116	p.V202G
143	*DOPEY1*	15018	6	83861669	missense	G	T	0.061	p.W1991L
143	*TRMT11*	1031712	6	126314922	missense	T	C	0.067	p.L31S
143	*TRMT11*	1031712	6	126314924	missense	C	T	0.066	p.L32F
143	*PCMT1*	5389	6	150123436	missense	G	A	0.074	p.S260N
143	*ARID1B*	20732	6	157528640	missense	G	A	0.057	p.R2122H
143	*PDCD2*	2598	6	170886760	missense	T	C	0.066	p.K275E
143	*SEMA3C*	6379	7	80374575	missense	G	A	0.125	p.R631C
143	*ZNF655*	138494	7	99170128	missense	G	A	0.055	p.E168K
143	*CYP3A4*	17460	7	99377697	missense	T	C	0.095	p.H28R
143	*PILRB*	178238	7	99956664	missense	T	G	0.050	p.L139W
143	*MUC17*	1040105	7	100675860	missense	T	C	0.111	p.L388P
143	*MUC17*	1040105	7	100676193	missense	A	C	0.119	p.N499T
143	*PLXNA4*	1105543	7	132069952	missense	T	C	0.070	p.N492D
143	*TNFRSF11B*	2546	8	119945254	missense	C	T	0.085	p.E106K
143	*IFNA14*	2172	9	21239862	missense	T	C	0.052	p.N25D
143	*CLTA*	7096	9	36198985	missense	G	A	0.114	p.G89S
143	*C9orf3*	32823	9	97522094	missense	A	G	0.198	p.D10G
143	*MSANTD3*	80655	9	103204567	missense	C	T	0.093	p.P116L
143	*TTF1*	7344	9	135276911	missense	A	G	0.061	p.V433A
143	*PPP1R26*	14811	9	138378056	missense	T	C	0.083	p.L567S
143	*TAF3*	31923	10	8006041	missense	C	T	0.062	p.P190S
143	*ANKRD30A*	52997	10	37508293	missense	G	A	0.050	p.S1162N
143	*ZNF37A*	3421	10	38406445	missense	A	C	0.051	p.K122N
143	*PGBD3*	170753	10	50724379	missense	A	G	0.055	p.V729A
143	*FGFR2*	23029	10	123244979	missense	C	T	0.191	p.V710M
143	*MKI67*	2417	10	129904259	missense	T	G	0.063	p.K1949Q
143	*MKI67*	2417	10	129904459	missense	T	C	0.057	p.K1882R
143	*OR52E2*	1005164	11	5080686	missense	G	A	0.067	p.H58Y
143	*OR10A4*	207186	11	6898219	missense	G	T	0.074	p.C114F
143	*MRGPRX3*	54031	11	18158756	missense	T	C	0.071	p.S3P
143	*BTBD18*	1145101	11	57512683	missense	C	A	0.095	p.Q354H
143	*GLYATL1*	80661	11	58722687	missense	A	G	0.116	p.T118A
143	*GLYATL1*	80661	11	58722698	missense	G	T	0.074	p.K121N
143	*CLPB*	30813	11	72005110	missense	C	T	0.085	p.D566N
143	*FKBP4*	2014	12	2909698	missense	T	G	0.138	p.H329Q

143	*DDX23*	4818	12	49239496	missense	G	A	0.077	p.R24W
143	*GALNT6*	7210	12	51773129	missense	G	A	0.063	p.A146V
143	*CTDSP2*	5730	12	58217698	missense	G	T	0.136	p.P227T
143	*CTDSP2*	5730	12	58217770	missense	G	C	0.133	p.L203V
143	*CTDSP2*	5730	12	58220811	missense	G	T	0.171	p.L108I
143	*CTDSP2*	5730	12	58220819	missense	A	G	0.081	p.V105A
143	*CTDSP2*	5730	12	58220831	missense	C	A	0.156	p.R101M
143	*TSPAN19*	1100917	12	85408282	missense	A	T	0.126	p.I244N
143	*RPL10L*	80746	14	47120818	missense	G	A	0.050	p.A41V
143	*PPP2R5E*	6246	14	63863387	missense	T	A	0.063	p.L199F
143	*WDR89*	80666	14	64066352	missense	T	A	0.069	p.R103S
143	*WDR89*	80666	14	64066367	missense	A	T	0.085	p.D98E
143	*WDR89*	80666	14	64066395	missense	C	T	0.099	p.C89Y
143	*WDR89*	80666	14	64066398	missense	G	C	0.102	p.A88G
143	*WDR89*	80666	14	64066402	missense	A	G	0.102	p.S87P
143	*TTC7B*	1010854	14	91059919	missense	G	A	0.111	p.S673L
143	*TMEM251*	1098621	14	93652617	missense	G	C	0.050	p.W37C
143	*SNURF*	22804	15	25207325	missense	C	T	0.184	p.R27C
143	*USP8*	5154	15	50769578	missense	G	A	0.080	p.G367D
143	*ITGAX*	1286375	16	31391603	missense	G	A	0.080	p.G1026D
143	*SALL1*	2968	16	51174697	missense	C	T	0.052	p.C479Y
143	*NOL3*	3946	16	67208844	missense	G	A	0.053	p.E268K
143	*MLYCD*	12213	16	83948772	missense	C	T	0.085	p.S387L
143	*METTL16*	24086	17	2344798	missense	G	T	0.082	p.L262I
143	*CTC1*	25099	17	8141735	missense	T	A	0.169	p.D137V
143	*SLFN12L*	1195790	17	33807072	missense	G	T	0.058	p.Q53K
143	*MRPL10*	148887	17	45901618	missense	T	C	0.093	p.M257V
143	*COX11*	4375	17	53040118	missense	C	A	0.070	p.K269N
143	*HLF*	2126	17	53398112	missense	G	A	0.070	p.A254T
143	*SLC16A3*	4207	17	80194643	missense	C	T	0.108	p.R88W
143	*TCEB3B*	16427	18	44559925	missense	T	C	0.063	p.K571E
143	*TCEB3B*	16427	18	44559927	missense	C	G	0.106	p.R570T
143	*LMNB2*	32737	19	2434474	missense	C	T	0.093	p.A341T
143	*MUC16*	24690	19	9066673	missense	G	C	0.057	p.H6925D
143	*GIPC1*	202494	19	14591568	missense	G	C	0.133	p.T104S
143	*WIZ*	21241	19	15538007	missense	G	T	0.082	p.S289R
143	*MAU2*	15329	19	19458149	missense	C	T	0.078	p.R428W
143	*ZNF225*	13362	19	44635647	missense	T	G	0.059	p.C294G
143	*ZNF415*	18355	19	53611776	missense	G	A	0.127	p.R508C
143	*KIR2DL4*	2255	19	55317499	missense	T	A	0.073	p.F152Y
143	*SYT5*	1297774	19	55686365	missense	C	A	0.087	p.Q237H
143	*ZBTB45*	32792	19	59028871	missense	A	G	0.057	p.F57S
143	*DZANK1*	1099407	20	18377154	missense	C	T	0.281	p.V525I
143	*MYL9*	6097	20	35176580	missense	C	A	0.065	p.F110L
143	*SALL4*	20436	20	50407959	missense	T	G	0.085	p.T355P
143	*TCP10L*	144659	21	33949205	missense	C	T	0.056	p.S176N
143	*DOPEY2*	5128	21	37571465	missense	T	C	0.200	p.V79A
143	*TTC3*	3316	21	38525560	missense	A	G	0.056	p.Q908R
143	*EIF3L*	16091	22	38251604	missense	A	C	0.089	p.N109T
143	*MXRA5*	15419	X	3240164	missense	G	T	0.070	p.P1188T
143	*OFD1*	3611	X	13762624	missense	A	G	0.062	p.N168S
143	*PIGA*	2641	X	15349697	missense	C	T	0.052	p.R119Q
143	*PHEX*	1282754	X	22108552	missense	C	G	0.167	p.D223E
143	*FAM104B*	1166703	X	55172521	missense	G	T	0.077	p.T114K
143	*FAM104B*	138362	X	55172689	missense	G	T	0.069	p.A59E
143	*TAF1*	138923	X	70608657	missense	T	A	0.132	p.M879K
143	*ARMCX4*	1256155	X	100746250	missense	T	C	0.082	p.S892P
143	*GLUD2*	12084	X	120183026	missense	T	G	0.075	p.S496R
145	*MAP3K6*	4672	1	27689342	stopgain	C	T	0.062	p.W381X
145	*USP34*	14709	2	61577773	stopgain	A	T	0.095	p.L436X
145	*REV1*	16316	2	100040723	stopgain	C	A	0.078	p.G523X
145	*MAP2*	31847	2	210574826	stopgain	G	T	0.051	p.E1641X
145	*ZBTB11*	14415	3	101390034	stopgain	G	A	0.057	p.R240X
145	*ENAM*	31889	4	71509124	stopgain	G	T	0.065	p.G661X
145	*C5orf42*	23073	5	37158435	stopgain	A	T	0.069	p.L2568X
145	*ITGA2*	2203	5	52351402	stopgain	C	T	0.052	p.R272X
145	*NUDT12*	31438	5	102894705	stopgain	A	T	0.063	p.L224X
145	*UQCRQ*	14402	5	132203218	stopgain	G	T	0.078	p.E65X
145	*CDKL3*	16508	5	133702107	stopgain	A	T	0.053	p.Y36X
145	*SLIT3*	3062	5	168233506	stopgain	G	A	0.213	p.R294X
145	*TBC1D32*	152730	6	121577283	stopgain	G	A	0.085	p.Q628X
145	*MTHFD1L*	15440	6	151286162	stopgain	C	T	0.160	p.Q668X
145	*RELN*	173054	7	103193910	stopgain	C	A	0.095	p.E2024X
145	*CREB3L2*	194071	7	137613091	stopgain	C	A	0.167	p.E42X
145	*UNC5D*	80872	8	35616959	stopgain	G	A	0.051	p.W762X
145	*MATN2*	30583	8	98991200	stopgain	G	T	0.065	p.G349X
145	*OXR1*	181354	8	107695493	stopgain	C	T	0.056	p.Q124X
145	*TAF2*	3184	8	120795650	stopgain	T	A	0.089	p.R695X
145	*KCNMA1*	2247	10	78799378	stopgain	C	T	0.085	p.W589X
145	*LRRC27*	30626	10	134169349	stopgain	A	T	0.061	p.K377X
145	*CFAP46*	1200049	10	134694433	stopgain	C	T	0.146	p.W1244X

145	*LMO7*	15842	13	76378636	stopgain	G	A	0.051	p.W343X
145	*SAMD4A*	15589	14	55203855	stopgain	C	T	0.069	p.R277X
145	*ESRRB*	4452	14	76948391	stopgain	C	T	0.083	p.Q183X
145	*CCDC88C*	1080414	14	91782119	stopgain	G	A	0.087	p.Q514X
145	*ASB2*	16150	14	94405789	stopgain	C	A	0.082	p.E428X
145	*CDYL2*	152342	16	80718807	stopgain	G	A	0.060	p.R82X
145	*CEP76*	24899	18	12698999	stopgain	C	A	0.065	p.E167X
145	*COX4I2*	32609	20	30231227	stopgain	G	T	0.091	p.E90X
145	*NDRG3*	32013	20	35284799	stopgain	C	A	0.098	p.E304X
145	*SYAP1*	32796	X	16754353	stopgain	C	A	0.091	p.S120X
145	*CXorf36*	176819	X	45051249	stopgain	C	T	0.054	p.W82X
145	*PLEKHG5*	198681	1	6535118	missense	C	A	0.056	p.K174N
145	*MTOR*	4958	1	11206776	missense	G	T	0.077	p.A1548D
145	*MFN2*	14874	1	12058890	missense	T	A	0.056	p.D221E
145	*VPS13D*	18156	1	12320777	missense	C	G	0.065	p.Q380E
145	*PHC2*	198040	1	33836635	missense	G	A	0.067	p.P132S
145	*CSMD2*	52896	1	34180265	missense	G	A	0.077	p.R1070C
145	*MTF1*	5955	1	38287942	missense	C	T	0.056	p.A540T
145	*COL9A2*	1852	1	40773405	missense	C	A	0.067	p.K308N
145	*DNAJC6*	14787	1	65852598	missense	C	T	0.121	p.R297W
145	*WLS*	24911	1	68603514	missense	T	A	0.058	p.M489L
145	*MTF2*	7358	1	93575896	missense	G	A	0.224	p.A39T
145	*DCST2*	144622	1	155006054	missense	G	T	0.054	p.L42I
145	*SLC50A1*	18845	1	155109307	missense	C	A	0.073	p.N54K
145	*OR10J3*	1004467	1	159283990	missense	G	A	0.074	p.L154F
145	*FASLG*	639	1	172634851	missense	C	A	0.061	p.L181I
145	*TNR*	3285	1	175362981	missense	C	T	0.053	p.V431M
145	*SWT1*	17673	1	185135752	missense	A	T	0.071	p.I45L
145	*PRG4*	5807	1	186280642	missense	T	A	0.060	p.F1236Y
145	*LHX9*	20204	1	197898138	missense	T	A	0.057	p.F306I
145	*TRAF3IP3*	25228	1	209936436	missense	A	T	0.066	p.K192M
145	*KCNH1*	172362	1	210970910	missense	A	G	0.197	p.C619R
145	*RCOR3*	18254	1	211449609	missense	T	C	0.052	p.M122T
145	*CENPF*	16343	1	214819387	missense	C	A	0.056	p.N2158K
145	*ARID4B*	31371	1	235416097	missense	A	G	0.196	p.L101P
145	*ERO1LB*	19891	1	236445020	missense	C	A	0.121	p.V22F
145	*ZNF695*	20394	1	247162673	missense	G	A	0.056	p.T79I
145	*BIRC6*	16252	2	32740298	missense	G	A	0.050	p.A3604T
145	*SOCS5*	144949	2	46986930	missense	C	A	0.050	p.R421S
145	*USP34*	14709	2	61463014	missense	G	A	0.074	p.T2333M
145	*UGP2*	6759	2	64112982	missense	G	T	0.194	p.V279F
145	*10-Sep*	178584	2	110325462	missense	C	T	0.108	p.S231N
145	*BCL2L11*	207003	2	111907653	missense	C	T	0.066	p.R83C
145	*BIN1*	139351	2	127826559	missense	G	A	0.070	p.R154W
145	*FAP*	4460	2	163029362	missense	C	A	0.056	p.A714S
145	*GALNT3*	4482	2	166615991	missense	C	A	0.082	p.D310Y
145	*TTC21B*	24753	2	166773804	missense	A	G	0.056	p.L621P
145	*SCRN3*	24583	2	175287699	missense	A	G	0.060	p.S281G
145	*TTN*	133378	2	179585713	missense	T	A	0.085	p.D7678V
145	*TTN*	133378	2	179594047	missense	C	T	0.058	p.S6279N
145	*MSTN*	5259	2	190922291	missense	T	C	0.063	p.E274G
145	*SLC39A10*	20342	2	196571444	missense	A	T	0.063	p.T441S
145	*IKZF2*	16260	2	213872667	missense	A	T	0.091	p.L307H
145	*FN1*	212482	2	216286882	missense	A	T	0.070	p.M493K
145	*CCDC108*	194302	2	219867730	missense	C	A	0.062	p.D1903Y
145	*SGPP2*	152386	2	223423403	missense	C	T	0.063	p.A329V
145	*BRPF1*	4634	3	9788033	missense	C	T	0.111	p.P1119L
145	*TRAK1*	1265608	3	42166993	missense	C	A	0.062	p.A58D
145	*NKTR*	5385	3	42672742	missense	A	G	0.067	p.S162G
145	*KLHL40*	152393	3	42727606	missense	G	A	0.085	p.D166N
145	*ABHD5*	16006	3	43759228	missense	G	A	0.062	p.R280Q
145	*PARP14*	17554	3	122437721	missense	C	A	0.052	p.H1575N
145	*NPHP3*	153240	3	132440866	missense	T	G	0.171	p.M112L
145	*ANAPC13*	15391	3	134197543	missense	T	A	0.054	p.E38D
145	*SLC35G2*	25246	3	136573802	missense	G	A	0.051	p.R167Q
145	*PLD1*	2662	3	171427470	missense	C	T	0.085	p.R314K
145	*CHRD*	3741	3	184106420	missense	A	T	0.162	p.D867V
145	*NELFA*	5663	4	1987886	missense	T	A	0.231	p.M275L
145	*SORCS2*	20777	4	7691261	missense	G	A	0.083	p.V513I
145	*CLNK*	52964	4	10515107	missense	G	T	0.078	p.P296H
145	*TMEM165*	18475	4	56290806	missense	A	T	0.065	p.R298S
145	*REST*	5612	4	57798179	missense	C	T	0.056	p.A1052V
145	*DCK*	788	4	71888211	missense	T	A	0.053	p.L112Q
145	*ANKRD17*	1286771	4	74088726	missense	C	T	0.067	p.E7K
145	*PPEF2*	6239	4	76797714	missense	G	T	0.118	p.P349H
145	*THAP9*	24672	4	83839689	missense	G	T	0.060	p.C775F
145	*ADH6*	1102470	4	100131429	missense	T	C	0.082	p.D126G
145	*FAT4*	24582	4	126371749	missense	T	C	0.063	p.V3193A
145	*KIAA0922*	15196	4	154507523	missense	C	T	0.125	p.P491S
145	*RXFP1*	21634	4	159567981	missense	G	A	0.088	p.G330R
145	*KLKB1*	892	4	187149371	missense	A	T	0.089	p.T8S

145	*TRIML2*	173553	4	189020217	missense	C	G	0.161	p.C148S
145	*MYO10*	12334	5	16673974	missense	G	T	0.098	p.S1663R
145	*C5orf42*	23073	5	37181016	missense	G	T	0.070	p.T1838N
145	*LIFR*	2310	5	38484919	missense	A	G	0.050	p.V850A
145	*RICTOR*	152756	5	38944624	missense	G	C	0.051	p.R1637G
145	*ARHGEF28*	1244364	5	73163755	missense	C	A	0.148	p.S736Y
145	*POC5*	152408	5	74988229	missense	T	C	0.052	p.R263G
145	*RASGRF2*	6909	5	80409671	missense	C	G	0.182	p.T801R
145	*ATP6AP1L*	1017971	5	81613895	missense	A	G	0.133	p.I151V
145	*CHSY3*	175856	5	129243869	missense	G	A	0.063	p.G301E
145	*HINT1*	5340	5	130495165	missense	C	T	0.082	p.R119Q
145	*CATSPER3*	178019	5	134305664	missense	G	T	0.058	p.R45I
145	*ANKHD1*	24668	5	139781806	missense	G	T	0.093	p.R85M
145	*TCOF1*	1195141	5	149771632	missense	C	T	0.053	p.T1137I
145	*F12*	505	5	176831338	missense	C	T	0.060	p.A293T
145	*SLC17A1*	5074	6	25820086	missense	A	G	0.056	p.Y89H
145	*HIST1H2AM*	3514	6	27860595	missense	G	T	0.080	p.N111K
145	*ZNF311*	1010877	6	28963421	missense	C	T	0.058	p.S453N
145	*MUC22*	1198815	6	30993390	missense	C	A	0.077	p.T61K
145	*HLA-DQA2*	20056	6	32713076	missense	A	C	0.063	p.S75R
145	*PTK7*	152882	6	43106613	missense	C	A	0.078	p.Q419K
145	*GPR111*	153839	6	47641199	missense	C	A	0.194	p.P15H
145	*GPR115*	153838	6	47682302	missense	G	A	0.055	p.V441M
145	*PHF3*	15153	6	64394089	missense	A	G	0.053	p.S68G
145	*SDK1*	152744	7	4089047	missense	G	T	0.098	p.W890C
145	*CYTH3*	4227	7	6205415	missense	G	C	0.053	p.R284G
145	*GPNMB*	2510	7	23296589	missense	G	A	0.121	p.G149D
145	*BAZ1B*	32408	7	72880718	missense	G	T	0.067	p.N960K
145	*GTF2IRD1*	16328	7	73973314	missense	G	A	0.061	p.G759D
145	*AKAP9*	147185	7	91709099	missense	T	A	0.059	p.V2543E
145	*SAP25*	1168682	7	100170048	missense	C	A	0.085	p.Q154H
145	*MUC17*	1040105	7	100678499	missense	T	A	0.081	p.L1268M
145	*NRCAM*	5010	7	107872884	missense	T	A	0.089	p.I105L
145	*CASP2*	32982	7	143001851	missense	T	A	0.095	p.M401K
145	*EZH2*	152998	7	148543608	missense	G	T	0.062	p.P67H
145	*CSMD1*	33225	8	2796234	missense	G	A	0.061	p.A3523V
145	*NAT1*	1291962	8	18079696	missense	C	A	0.056	p.A109D
145	*CCAR2*	21174	8	22472003	missense	T	C	0.052	p.L343P
145	*LETM2*	144652	8	38260109	missense	G	T	0.129	p.G351W
145	*VCPIP1*	25054	8	67546792	missense	T	A	0.056	p.M1205L
145	*PREX2*	24870	8	69032482	missense	G	A	0.068	p.D1186N
145	*DCAF4L2*	152418	8	88885412	missense	T	A	0.069	p.K263M
145	*OSGIN2*	1126111	8	90921857	missense	A	T	0.054	p.D36V
145	*INTS8*	17864	8	95850730	missense	C	T	0.091	p.R301W
145	*CSMD3*	198124	8	113318412	missense	C	A	0.172	p.C2592F
145	*FER1L6*	1039112	8	125088439	missense	G	A	0.066	p.A1365T
145	*SLA*	6748	8	134050923	missense	C	T	0.083	p.S226N
145	*SCRIB*	182706	8	144886022	missense	G	A	0.167	p.T1070M
145	*ARID3C*	1017363	9	34623626	missense	G	A	0.064	p.R221W
145	*TESK1*	6285	9	35607333	missense	G	T	0.054	p.V183F
145	*TRPM3*	206947	9	73225536	missense	A	G	0.056	p.W746R
145	*PRUNE2*	15225	9	79323241	missense	T	C	0.105	p.K1317E
145	*RMI1*	24945	9	86616935	missense	C	T	0.051	p.A345V
145	*KLF4*	4235	9	110250032	missense	G	A	0.080	p.P215S
145	*RC3H2*	1100588	9	125611995	missense	C	A	0.069	p.G1163C
145	*ABL1*	7313	9	133759761	missense	G	A	0.055	p.S714N
145	*NET1*	5863	10	5496309	missense	C	A	0.061	p.P284T
145	*FAM208B*	17782	10	5790986	missense	T	C	0.057	p.S1868P
145	*CUBN*	1081	10	17113480	missense	T	A	0.053	p.N857I
145	*SVIL*	21738	10	29843824	missense	A	T	0.066	p.N16K
145	*FZD8*	31866	10	35929151	missense	C	T	0.070	p.V403I
145	*CSGALNACT2*	18590	10	43651070	missense	A	G	0.051	p.Q158R
145	*GDF10*	4962	10	48429311	missense	G	T	0.133	p.A192D
145	*HK1*	33500	10	71144115	missense	C	A	0.078	p.L521I
145	*ZSWIM8*	15037	10	75559117	missense	C	A	0.057	p.P1512T
145	*KAT6B*	12330	10	76602713	missense	G	A	0.056	p.C33Y
145	*LRIT1*	15613	10	85992038	missense	C	G	0.073	p.R506P
145	*IFIT1*	1548	10	91152471	missense	T	A	0.211	p.M1K
145	*PLCE1*	16341	10	95987078	missense	A	G	0.138	p.T609A
145	*C10orf12*	15652	10	98741197	missense	C	A	0.055	p.A17D
145	*SORCS1*	52918	10	108427534	missense	T	A	0.074	p.N739I
145	*FAM160B1*	20940	10	116605797	missense	C	A	0.052	p.A385D
145	*DMBT1*	17579	10	124389424	missense	G	A	0.060	p.A1784T
145	*CHST15*	15892	10	125805716	missense	T	G	0.114	p.I5L
145	*TRIM68*	18073	11	4621730	missense	G	A	0.068	p.P412S
145	*SLC5A12*	178498	11	26743176	missense	G	A	0.056	p.A29V
145	*BDNF*	170735	11	27679862	missense	T	G	0.085	p.N84H
145	*KIAA1549L*	12194	11	33596318	missense	G	A	0.063	p.G1137E
145	*SLC1A2*	4171	11	35336579	missense	A	T	0.108	p.L92I
145	*CKAP5*	1008938	11	46776482	missense	C	T	0.063	p.E1609K
145	*SMTNL1*	1105565	11	57310351	missense	A	G	0.073	p.E79G

145	*AHNAK*	1620	11	62300307	missense	C	A	0.053	p.D528Y
145	*HNRNPUL2*	1079559	11	62491181	missense	A	G	0.066	p.F257L
145	*DPF2*	6268	11	65113431	missense	C	A	0.063	p.P269H
145	*KCNK7*	33347	11	65360542	missense	C	G	0.053	p.Q286H
145	*LRFN4*	24036	11	66626183	missense	G	A	0.053	p.G323D
145	*CORO1B*	20441	11	67210002	missense	C	T	0.055	p.R33H
145	*NUMA1*	6185	11	71727068	missense	G	T	0.055	p.A494D
145	*ARAP1*	15242	11	72408211	missense	A	T	0.055	p.C995S
145	*RELT*	152222	11	73103469	missense	C	T	0.053	p.T194M
145	*TPBGL*	1195528	11	74953106	missense	C	T	0.085	p.R338C
145	*TENM4*	1098816	11	78380529	missense	C	G	0.163	p.W2287C
145	*PCF11*	15885	11	82878277	missense	G	A	0.053	p.R643Q
145	*POU2AF1*	6235	11	111225009	missense	T	A	0.075	p.T250S
145	*CCDC15*	25004	11	124863125	missense	C	A	0.074	p.P734T
145	*WNK1*	213655	12	970360	missense	C	A	0.056	p.T601K
145	*CACNA2D4*	172364	12	1919749	missense	C	A	0.075	p.S873I
145	*CD9*	1769	12	6309691	missense	G	A	0.056	p.C9Y
145	*CD163L1*	174941	12	7527290	missense	G	A	0.072	p.H1053Y
145	*A2M*	14	12	9246128	missense	C	T	0.102	p.E725K
145	*LRP6*	2336	12	12311834	missense	A	G	0.156	p.V907A
145	*PKP2*	4572	12	32945649	missense	C	A	0.133	p.A836S
145	*SYT10*	198992	12	33535419	missense	C	A	0.068	p.R412L
145	*LMBR1L*	18113	12	49498244	missense	G	A	0.053	p.A136V
145	*FAM186B*	32130	12	49994839	missense	G	T	0.082	p.P195Q
145	*SLC4A8*	1258401	12	51890859	missense	G	A	0.077	p.S958N
145	*KRT3*	57088	12	53189427	missense	C	G	0.109	p.A134P
145	*GLI1*	5269	12	57865738	missense	G	A	0.063	p.G1072E
145	*CTDSP2*	5730	12	58217698	missense	G	T	0.065	p.P227T
145	*CTDSP2*	5730	12	58217770	missense	G	T	0.062	p.L203I
145	*CTDSP2*	5730	12	58220801	missense	G	T	0.084	p.T111N
145	*CTDSP2*	5730	12	58220811	missense	G	T	0.053	p.L108I
145	*CTDSP2*	5730	12	58220819	missense	A	G	0.097	p.V105A
145	*BBS10*	24685	12	76741028	missense	G	T	0.080	p.S246Y
145	*FGD6*	18351	12	95603229	missense	C	T	0.056	p.V611M
145	*TMEM132C*	1136103	12	129180598	missense	C	A	0.050	p.Q627K
145	*BRCA2*	59	13	32910581	missense	A	G	0.051	p.K697E
145	*EXOSC8*	181503	13	37578660	missense	C	T	0.050	p.A67V
145	*THSD1*	199263	13	52971819	missense	G	A	0.191	p.T190I
145	*PNP*	270	14	20944591	missense	G	A	0.060	p.R234Q
145	*EDDM3B*	22360	14	21238629	missense	A	T	0.056	p.Q107L
145	*HOMEZ*	20834	14	23746180	missense	G	A	0.052	p.A86V
145	*LRRC16B*	138360	14	24528549	missense	G	A	0.050	p.G566D
145	*FBXO34*	152231	14	55817500	missense	G	A	0.063	p.R131K
145	*KTN1*	4986	14	56117061	missense	T	A	0.082	p.D807E
145	*WDR89*	80666	14	64066367	missense	A	T	0.083	p.D98E
145	*WDR89*	80666	14	64066395	missense	C	T	0.133	p.C89Y
145	*WDR89*	80666	14	64066398	missense	G	C	0.115	p.A88G
145	*WDR89*	80666	14	64066402	missense	A	G	0.126	p.S87P
145	*AKAP5*	4857	14	64936364	missense	G	A	0.065	p.D418N
145	*MAP3K9*	33141	14	71216715	missense	G	A	0.058	p.A362V
145	*FOS*	5252	14	75747586	missense	G	T	0.058	p.R201L
145	*DDX24*	20414	14	94528621	missense	C	A	0.069	p.E355D
145	*SERPINA1*	1127707	14	94849473	missense	C	A	0.077	p.K34N
145	*SQRDL*	21199	15	45981393	missense	C	A	0.098	p.L425M
145	*SLC12A1*	1184832	15	48499939	missense	A	G	0.059	p.N8S
145	*CEP152*	14985	15	49034199	missense	G	A	0.054	p.R1312C
145	*LEO1*	138792	15	52257978	missense	G	A	0.054	p.S261L
145	*LDHAL6B*	33195	15	59500155	missense	G	A	0.086	p.S339N
145	*MYO1E*	4998	15	59510272	missense	C	T	0.167	p.A309T
145	*VPS13C*	20821	15	62255003	missense	G	A	0.053	p.A1127V
145	*VPS13C*	20821	15	62266596	missense	A	T	0.053	p.L810H
145	*NOX5*	24505	15	69347718	missense	A	G	0.070	p.K682E
145	*TLE3*	20908	15	70388588	missense	G	A	0.054	p.P13S
145	*CSPG4*	1897	15	75977720	missense	C	A	0.056	p.G1371V
145	*SCAPER*	20843	15	76914160	missense	A	T	0.063	p.Y640N
145	*IL16*	172217	15	81517751	missense	A	T	0.080	p.H4L
145	*IQGAP1*	3870	15	91017876	missense	T	A	0.095	p.L912H
145	*TNRC6A*	14494	16	24802979	missense	T	A	0.068	p.S1006T
145	*TNRC6A*	14494	16	24834817	missense	T	A	0.058	p.F1860I
145	*SRCAP*	6662	16	30733463	missense	G	A	0.093	p.G1188R
145	*HERPUD1*	14685	16	56966197	missense	T	C	0.111	p.V14A
145	*PSKH1*	6742	16	67942738	missense	G	A	0.056	p.G29D
145	*PMFBP1*	31293	16	72153955	missense	C	A	0.067	p.Q809H
145	*ZFHX3*	6885	16	72828030	missense	C	T	0.190	p.G2851S
145	*FANCA*	1286167	16	89874739	missense	C	T	0.063	p.V187I
145	*DOC2B*	3585	17	22359	missense	T	A	0.087	p.N141I
145	*INPP5K*	16532	17	1417221	missense	T	A	0.052	p.S33C
145	*C17orf85*	1114118	17	3732603	missense	G	T	0.077	p.Q139K
145	*ACAP1*	14716	17	7247217	missense	A	T	0.100	p.Q204L
145	*C17orf74*	175734	17	7330559	missense	G	A	0.050	p.A417T
145	*ALOXE3*	21628	17	8018940	missense	C	T	0.077	p.R140Q

145	*MYH2*	17534	17	10435038	missense	G	A	0.068	p.A870V
145	*MYH3*	2470	17	10545856	missense	T	C	0.073	p.Y589C
145	*ADORA2B*	676	17	15848699	missense	T	A	0.082	p.L46Q
145	*ZNF624*	20787	17	16525654	missense	G	A	0.093	p.S849L
145	*FLCN*	144997	17	17117077	missense	C	A	0.056	p.E544D
145	*ALKBH5*	17758	17	18087849	missense	G	A	0.062	p.D98N
145	*SLC6A4*	1045	17	28548960	missense	A	G	0.056	p.L6S
145	*ZNF830*	52857	17	33288595	missense	T	G	0.146	p.S4A
145	*SRCIN1*	25248	17	36720438	missense	G	A	0.067	p.P153S
145	*HCRT*	1524	17	40336452	missense	C	T	0.063	p.C39Y
145	*ATP6V0A1*	5177	17	40642572	missense	A	T	0.063	p.Y364F
145	*EMILIN2*	32048	18	2909745	missense	C	T	0.070	p.P918S
145	*TGIF1*	174886	18	3457688	missense	A	T	0.051	p.Q170L
145	*GREB1L*	1142966	18	19029526	missense	G	A	0.095	p.M483I
145	*LAMA3*	198129	18	21426358	missense	G	A	0.057	p.A1273T
145	*SETBP1*	15559	18	42530201	missense	C	T	0.159	p.P299L
145	*KDSR*	2035	18	61006107	missense	T	A	0.065	p.T235S
145	*ABCA7*	19112	19	1046922	missense	C	T	0.133	p.R582C
145	*LMNB2*	32737	19	2431819	missense	C	T	0.079	p.E558K
145	*HSD11B1L*	198708	19	5687929	missense	G	A	0.063	p.S224N
145	*ZNF846*	1077624	19	9869316	missense	T	C	0.050	p.Q146R
145	*CC2D1A*	17721	19	14034210	missense	C	A	0.080	p.S569Y
145	*HAUS8*	33417	19	17170858	missense	G	T	0.121	p.L92M
145	*CRTC1*	15321	19	18870394	missense	G	A	0.052	p.G236R
145	*HAUS5*	15302	19	36110961	missense	C	A	0.054	p.P485H
145	*GMFG*	4877	19	39819122	missense	G	A	0.125	p.R142C
145	*FCGBP*	3890	19	40366547	missense	G	A	0.105	p.P4563S
145	*FCGBP*	3890	19	40366549	missense	A	G	0.105	p.L4562P
145	*AXL*	21913	19	41765523	missense	G	T	0.077	p.R800L
145	*LYPD5*	182573	19	44301885	missense	C	T	0.080	p.G162D
145	*CD3EAP*	12099	19	45912071	missense	C	T	0.063	p.T282I
145	*PNMAL1*	18215	19	46973161	missense	C	A	0.051	p.D378Y
145	*ARHGAP35*	4491	19	47424543	missense	C	T	0.065	p.R871C
145	*BBC3*	14417	19	47729852	missense	G	A	0.118	p.A145V
145	*KPTN*	7059	19	47979789	missense	C	A	0.075	p.Q394H
145	*CLDND2*	152353	19	51871172	missense	C	T	0.073	p.G93D
145	*ZNF350*	21632	19	52468780	missense	T	A	0.138	p.Q309L
145	*PRKCG*	2739	19	54385809	missense	A	G	0.121	p.R21G
145	*LILRB3*	24318	19	54744781	missense	T	G	0.160	p.Y291S
145	*PPP1R12C*	17607	19	55604014	missense	G	A	0.075	p.P643L
145	*PPP6R1*	14931	19	55750803	missense	T	C	0.070	p.E574G
145	*ZNF582*	144690	19	56895321	missense	T	C	0.053	p.I489V
145	*ZNF606*	25027	19	58491143	missense	A	T	0.062	p.I302K
145	*ZNF135*	7134	19	58573071	missense	G	A	0.063	p.V53M
145	*ZNF324B*	207395	19	58965158	missense	G	A	0.056	p.M30I
145	*TMC2*	80751	20	2559828	missense	A	G	0.073	p.K228E
145	*ATRN*	139322	20	3543044	missense	C	A	0.055	p.F523L
145	*BPI*	1725	20	36938960	missense	A	T	0.061	p.S152C
145	*KREMEN1*	32045	22	29533438	missense	G	A	0.058	p.R247Q
145	*RRP7A*	15703	22	42915746	missense	C	T	0.200	p.R16H
145	*EFCAB6*	198856	22	44022409	missense	C	T	0.078	p.V795I
145	*PPP6R2*	14678	22	50874814	missense	G	A	0.065	p.R512H
145	*HDAC6*	6044	X	48664837	missense	T	C	0.073	p.L167P
145	*ZC4H2*	18684	X	64139054	missense	C	A	0.054	p.Q143H
145	*KIF4A*	12310	X	69639596	missense	G	A	0.074	p.S1153N
145	*HDX*	144657	X	83581198	missense	C	A	0.069	p.K645N
145	*CXorf57*	18015	X	105882762	missense	T	C	0.061	p.S527P
145	*ALG13*	1257237	X	110968248	missense	G	A	0.114	p.R459K
145	*AMOT*	133265	X	112035202	missense	T	A	0.061	p.K186M
145	*MTMR1*	3828	X	149924290	missense	T	G	0.060	p.L596V
145	*GABRQ*	18558	X	151815476	missense	C	A	0.061	p.T125N
145	*SLC10A3*	19848	X	153715967	missense	C	T	0.064	p.R409H
145	GNAQ	2072	9	80537112	missense	T	A	0.261	p.T96S
147	*SEC24D*	14822	4	119660349	stopgain	T	A	0.160	p.K778X
147	*WDR89*	80666	14	64066363	stopgain	G	A	0.108	p.R100X
147	*ATP2A1*	173201	16	28912148	stopgain	C	T	0.060	p.R671X
147	*KIAA1328*	20776	18	34647273	stopgain	G	T	0.206	p.E333X
147	*CACNA1I*	21096	22	40076984	stopgain	C	A	0.103	p.S1864X
147	*ZBTB48*	5341	1	6642217	missense	C	T	0.087	p.P264S
147	*TIE1*	5424	1	43783241	missense	C	T	0.062	p.A876V
147	*ERICH3*	1002912	1	75078377	missense	C	T	0.264	p.G373S
147	*COLGALT2*	15101	1	183942796	missense	T	C	0.219	p.E194G
147	*CD46*	172361	1	207940453	missense	G	A	0.058	p.D257N
147	*SYT14*	153262	1	210267899	missense	A	T	0.065	p.E187D
147	*ACVR1C*	145259	2	158406791	missense	C	G	0.276	p.A220P
147	*TTN*	133378	2	179583962	missense	G	A	0.221	p.T8052I
147	*AGXT*	30	2	241817009	missense	G	A	0.111	p.R301H
147	*XPC*	4628	3	14219989	missense	G	A	0.197	p.A27V
147	*CSRNP1*	33027	3	39186567	missense	G	A	0.054	p.A129V
147	*ZNF717*	1290209	3	75788230	missense	C	T	0.061	p.V132I
147	*ZIC4*	32153	3	147108985	missense	G	A	0.065	p.A246V

147	*PEX5L*	16559	3	179529592	missense	T	G	0.176	p.Q384P
147	*RFC1*	2913	4	39344058	missense	T	C	0.069	p.K80E
147	*PTPN13*	80685	4	87691072	missense	A	C	0.241	p.K1552T
147	*CDH12*	4061	5	21755750	missense	A	G	0.250	p.L612P
147	*MROH2B*	173489	5	41004501	missense	C	T	0.065	p.V1381M
147	*NUDT12*	31438	5	102895154	missense	A	C	0.174	p.I74M
147	*PCDHGB6*	32100	5	140789036	missense	G	A	0.140	p.V423I
147	*RIPK1*	3804	6	3113419	missense	G	A	0.170	p.R621Q
147	*SDK1*	152744	7	4091412	missense	C	A	0.065	p.P954H
147	*GRID2IP*	1145118	7	6537817	missense	C	T	0.175	p.G1148R
147	*CRHR2*	1883	7	30721564	missense	C	T	0.063	p.E52K
147	*TNS3*	22748	7	47336776	missense	A	C	0.258	p.F1194V
147	*PCLO*	33026	7	82580404	missense	G	A	0.278	p.T3167I
147	*FBXL13*	145032	7	102667927	missense	C	A	0.056	p.R99I
147	*SHH*	193	7	155599169	missense	C	A	0.061	p.W128L
147	*DLGAP2*	4745	8	1616708	missense	T	G	0.116	p.L595R
147	*PTK2B*	173176	8	27301727	missense	G	A	0.286	p.S718N
147	*MALRD1*	1142308	10	19413531	missense	G	A	0.063	p.R292H
147	*C10orf25*	1039380	10	45496249	missense	A	G	0.055	p.L20P
147	*TIAL1*	3252	10	121341701	missense	C	A	0.061	p.S88I
147	*CTDSP2*	5730	12	58217698	missense	G	T	0.058	p.P227T
147	*CTDSP2*	5730	12	58217763	missense	C	T	0.217	p.R205H
147	*CTDSP2*	5730	12	58217770	missense	G	C	0.085	p.L203V
147	*CTDSP2*	5730	12	58220811	missense	G	T	0.060	p.L108I
147	*LRIG3*	153377	12	59268296	missense	G	T	0.063	p.L919I
147	*LATS2*	14572	13	21557725	missense	C	T	0.242	p.R707Q
147	*WDR89*	80666	14	64066352	missense	T	A	0.067	p.R103S
147	*WDR89*	80666	14	64066367	missense	A	T	0.100	p.D98E
147	*WDR89*	80666	14	64066395	missense	C	T	0.132	p.C89Y
147	*WDR89*	80666	14	64066398	missense	G	C	0.135	p.A88G
147	*WDR89*	80666	14	64066402	missense	A	G	0.136	p.S87P
147	*SPTB*	1024858	14	65251050	missense	C	T	0.078	p.R1306Q
147	*DPH6*	80650	15	35834658	missense	T	C	0.186	p.H25R
147	*CRAMP1L*	20825	16	1706779	missense	G	A	0.182	p.R674H
147	*OR1E2*	3554	17	3336748	missense	T	G	0.097	p.M130L
147	*OR1E2*	3554	17	3336750	missense	G	A	0.083	p.P129L
147	*KRT40*	182497	17	39137138	missense	G	T	0.234	p.L292M
147	*DLGAP1*	4746	18	3879245	missense	T	C	0.152	p.K275R
147	*LAMA3*	198129	18	21293894	missense	G	A	0.238	p.C102Y
147	*TCEB3B*	16427	18	44559941	missense	A	T	0.250	p.D565E
147	*ZNF266*	6631	19	9524511	missense	T	G	0.093	p.N364H
147	*MED26*	4831	19	16688019	missense	G	A	0.202	p.R208C
147	*SYNE4*	1039876	19	36497837	missense	C	T	0.167	p.V145M
147	*CD40*	152854	20	44751261	missense	G	A	0.067	p.R90Q
147	*MIF*	2415	22	24236995	missense	G	A	0.172	p.A49T
147	*MIF*	2415	22	24237001	missense	G	A	0.167	p.G51S
150	*C2orf57*	152614	2	232458089	stopgain	A	T	0.154	p.R143X
150	*TLL1*	12464	4	166981338	stopgain	G	T	0.157	p.E669X
150	*LRRD1*	1161528	7	91774343	stopgain	C	T	0.050	p.W814X
150	*TMC1*	138691	9	75404222	stopgain	G	T	0.150	p.E405X
150	*LYZL1*	32517	10	29581448	stopgain	G	A	0.132	p.W93X
150	*WDR89*	80666	14	64066363	stopgain	G	A	0.087	p.R100X
150	*SMCHD1*	15295	18	2739512	stopgain	G	T	0.106	p.E1170X
150	*VPS16*	22575	20	2843264	stopgain	C	T	0.083	p.Q371X
150	*TCN2*	1184726	22	31022460	stopgain	C	G	0.052	p.Y385X
150	*SAMD13*	1134664	1	84791375	missense	G	A	0.070	p.V37I
150	*WDR63*	145172	1	85573786	missense	G	C	0.115	p.E542Q
150	*PRUNE*	21222	1	150990334	missense	T	C	0.058	p.L29S
150	*MUC1*	1204286	1	155161015	missense	G	A	0.138	p.T180I
150	*LMX1A*	177398	1	165175103	missense	T	C	0.071	p.Y329C
150	*LGR6*	21636	1	202288114	missense	G	A	0.088	p.G843S
150	*OBSCN*	1271223	1	228561708	missense	A	G	0.074	p.D8417G
150	*GAREML*	1168241	2	26410482	missense	G	A	0.138	p.G661S
150	*TBC1D8*	1102426	2	101650076	missense	G	A	0.144	p.T568M
150	*EVX2*	1080458	2	176948202	missense	C	A	0.170	p.K101N
150	*TTN*	133378	2	179585731	missense	C	G	0.095	p.S7672T
150	*SSUH2*	15931	3	8671409	missense	A	G	0.165	p.C155R
150	*ZNF717*	1290209	3	75788230	missense	C	T	0.082	p.V132I
150	*TF*	1063	3	133485253	missense	A	T	0.057	p.N488Y
150	*ANAPC13*	15391	3	134197439	missense	C	T	0.136	p.G73E
150	*ZNF732*	1137608	4	264941	missense	A	T	0.070	p.W568R
150	*SDAD1*	18115	4	76892532	missense	T	A	0.082	p.E264V
150	*WDFY3*	14991	4	85614039	missense	T	A	0.145	p.K3016N
150	*DCHS2*	17639	4	155160363	missense	A	T	0.079	p.I2029N
150	*GUCY1A3*	1256449	4	156632390	missense	A	G	0.053	p.K358R
150	*C1QTNF3*	181435	5	34042981	missense	G	C	0.116	p.L84V
150	*MAP3K1*	5921	5	56177851	missense	A	T	0.079	p.T942S
150	*HLA-DQA2*	20056	6	32712967	missense	C	G	0.063	p.F38L
150	*HLA-DQA2*	20056	6	32713076	missense	A	C	0.059	p.S75R
150	*HLA-DQA2*	20056	6	32713080	missense	A	G	0.058	p.K76R
150	*HLA-DQA2*	20056	6	32713090	missense	T	A	0.051	p.S79R

150	*HLA-DPB1*	2121	6	33048596	missense	C	T	0.095	p.P83L
150	*MYCT1*	25107	6	153019103	missense	T	G	0.098	p.D22E
150	*TRRAP*	3496	7	98609027	missense	G	T	0.119	p.D3722Y
150	*CLN8*	18941	8	1728650	missense	C	A	0.145	p.P260T
150	*PIP5K1B*	3558	9	71509373	missense	G	T	0.117	p.R197I
150	*FOXB2*	1013735	9	79635863	missense	C	G	0.121	p.H431Q
150	*GABBR2*	5458	9	101470769	missense	G	A	0.100	p.A84V
150	*NRP1*	3873	10	33496542	missense	C	T	0.157	p.G573R
150	*NCOA4*	1145260	10	51586656	missense	A	G	0.059	p.Q645R
150	*FAM13C*	198215	10	61028363	missense	G	A	0.140	p.R215W
150	*ARID5B*	32199	10	63810748	missense	G	T	0.108	p.A279S
150	*KCNMA1*	2247	10	78669852	missense	T	C	0.075	p.N979D
150	*CNNM2*	199077	10	104678734	missense	A	G	0.139	p.N166S
150	*CTBP2*	22802	10	126686646	missense	C	G	0.129	p.W151S
150	*CTBP2*	22802	10	126686649	missense	G	C	0.125	p.T150R
150	*CTBP2*	22802	10	126686656	missense	T	C	0.118	p.R148G
150	*CTBP2*	22802	10	126686659	missense	G	A	0.105	p.R147W
150	*JAKMIP3*	1105521	10	133949525	missense	C	A	0.060	p.A354D
150	*SLC22A12*	144585	11	64359201	missense	C	T	0.093	p.T58M
150	*LTBP3*	21070	11	65325216	missense	T	A	0.053	p.K72M
150	*TENM4*	1098816	11	78387252	missense	C	T	0.083	p.R1814H
150	*GRIA4*	1077243	11	105804609	missense	G	C	0.068	p.Q736H
150	*SCN2B*	4588	11	118039422	missense	T	C	0.053	p.N39D
150	*ESAM*	138961	11	124628309	missense	G	A	0.056	p.S62L
150	*SLCO1B7*	1009562	12	21229404	missense	T	C	0.079	p.I542T
150	*ADAMTS20*	25003	12	43769882	missense	A	C	0.185	p.F1764V
150	*KMT2D*	3482	12	49420390	missense	C	T	0.063	p.R5120H
150	*TFCP2*	5653	12	51495751	missense	T	A	0.063	p.D373V
150	*KRT3*	57088	12	53189427	missense	C	G	0.159	p.A134P
150	*SMARCC2*	139067	12	56572249	missense	C	T	0.117	p.V417M
150	*CTDSP2*	5730	12	58217698	missense	G	T	0.077	p.P227T
150	*CTDSP2*	5730	12	58217763	missense	C	T	0.194	p.R205H
150	*CTDSP2*	5730	12	58217770	missense	G	C	0.104	p.L203V
150	*CTDSP2*	5730	12	58220801	missense	G	T	0.076	p.T111N
150	*CTDSP2*	5730	12	58240188	missense	G	A	0.123	p.R11W
150	*CTDSP2*	5730	12	58240214	missense	T	G	0.103	p.E2A
150	*SRGAP1*	20762	12	64536346	missense	A	G	0.104	p.K1051R
150	*TBK1*	13254	12	64868050	missense	A	G	0.063	p.H194R
150	*SART3*	14706	12	108942902	missense	G	A	0.053	p.A134V
150	*CIT*	7174	12	120263095	missense	T	A	0.056	p.K277N
150	*HECTD1*	15382	14	31576195	missense	C	G	0.068	p.D2295H
150	*WDR89*	80666	14	64066326	missense	C	T	0.052	p.G112D
150	*WDR89*	80666	14	64066352	missense	T	A	0.080	p.R103S
150	*WDR89*	80666	14	64066367	missense	A	T	0.102	p.D98E
150	*WDR89*	80666	14	64066395	missense	C	T	0.110	p.C89Y
150	*WDR89*	80666	14	64066398	missense	G	C	0.114	p.A88G
150	*WDR89*	80666	14	64066402	missense	A	G	0.103	p.S87P
150	*SNW1*	12245	14	78202302	missense	A	T	0.063	p.V229D
150	*HSP90AA1*	5348	14	102551278	missense	T	C	0.177	p.K363E
150	*CCNB2*	4701	15	59406704	missense	C	T	0.124	p.S110F
150	*C16orf59*	25108	16	2514581	missense	G	C	0.080	p.W405S
150	*GTF3C1*	1520	16	27480623	missense	A	G	0.108	p.L1688P
150	*NLRC5*	32206	16	57060237	missense	G	A	0.193	p.G461E
150	*HYDIN*	1270974	16	70894662	missense	C	T	0.221	p.G3974R
150	*CDH15*	4933	16	89245863	missense	C	T	0.089	p.P28S
150	*RAI1*	30665	17	17697096	missense	G	C	0.082	p.Q278H
150	*CNTNAP1*	3632	17	40838074	missense	G	A	0.151	p.G272D
150	*CDH2*	1792	18	25585862	missense	C	A	0.080	p.E266D
150	*CELF4*	20180	18	34853104	missense	A	G	0.121	p.L275P
150	*ELANE*	1972	19	852389	missense	C	A	0.069	p.L21M
150	*ZNF565*	152477	19	36673426	missense	C	T	0.132	p.G481E
150	*DYRK1B*	6484	19	40316528	missense	C	T	0.125	p.A545T
150	*NUMBL*	4756	19	41186857	missense	A	G	0.056	p.W169R
150	*PINLYP*	1193621	19	44081333	missense	A	G	0.060	p.D21G
150	*TMC4*	144686	19	54668319	missense	G	A	0.066	p.T321I
150	*FCAR*	133279	19	55399635	missense	G	A	0.100	p.S208N
150	*ZNF787*	1002836	19	56600081	missense	C	T	0.108	p.D154N
150	*ZNF787*	1002836	19	56600086	missense	C	G	0.108	p.C152S
150	*MCM8*	182802	20	5948171	missense	G	A	0.081	p.S322N
150	*SSTR4*	1052	20	23016698	missense	G	A	0.071	p.G193D
150	*PYGB*	2862	20	25273134	missense	C	T	0.098	p.L688F
150	*ERGIC3*	198398	20	34136300	missense	G	A	0.106	p.R167H
150	*SMTN*	134270	22	31487142	missense	C	A	0.103	p.S378Y
150	*GTSE1*	16426	22	46704274	missense	G	T	0.135	p.A66S
150	*CHKB*	5198	22	51021156	missense	C	T	0.138	p.A19T
150	*MSL3*	78629	X	11776437	missense	T	A	0.108	p.C19S
150	*KLHL15*	30624	X	24006592	missense	C	T	0.128	p.G421R
150	*DMD*	4012	X	32407676	missense	G	A	0.050	p.A1364V
150	*CCNB3*	33670	X	50089691	missense	G	A	0.128	p.C1232Y
150	*FAM104B*	138362	X	55172647	missense	G	A	0.051	p.P73L
150	*FAM104B*	138362	X	55172686	missense	C	T	0.103	p.S60N

150	*FAM104B*	138362	X	55172689	missense	G	T	0.108	p.A59E
150	*STARD8*	14725	X	67942372	missense	A	C	0.088	p.S895R
3334	*PARM1*	15393	4	75937882	stopgain	G	A	0.140	p.W97X
3334	*B3GALT4*	3782	6	33246307	stopgain	C	T	0.056	p.R371X
3334	*MYB*	5375	6	135520050	stopgain	T	G	0.164	p.L508X
3334	*ZNF394*	32164	7	99097571	stopgain	C	T	0.196	p.W49X
3334	*CUL2*	3591	10	35349875	stopgain	G	A	0.174	p.Q82X
3334	*POLR3B*	18082	12	106857344	stopgain	C	T	0.143	p.Q887X
3334	*MGA*	1164273	15	41989087	stopgain	C	T	0.121	p.R627X
3334	*IGDCC3*	4884	15	65624427	stopgain	G	A	0.125	p.Q334X
3334	*FGF16*	3868	X	76711924	stopgain	C	T	0.152	p.R88X
3334	*UBXN11*	183008	1	26608897	missense	A	T	0.056	p.C486S
3334	*PTPRU*	133178	1	29587289	missense	C	A	0.071	p.Q340K
3334	*SPOCD1*	144569	1	32257864	missense	G	T	0.160	p.P972T
3334	*APH1A*	16022	1	150240375	missense	G	A	0.051	p.A89V
3334	*LY9*	1033667	1	160771648	missense	G	A	0.065	p.G175S
3334	*IPO9*	18085	1	201798384	missense	C	A	0.129	p.P16Q
3334	*OR6F1*	1005286	1	247875510	missense	G	A	0.324	p.P183L
3334	*TTN*	133437	2	179500421	missense	A	G	0.167	p.V13877A
3334	*ADAMTS9*	182920	3	64547324	missense	G	A	0.167	p.P1543L
3334	*C3orf38*	173824	3	88202433	missense	C	T	0.091	p.R63C
3334	*MMRN1*	7351	4	90874325	missense	A	G	0.167	p.K1148R
3334	*8-Sep*	15146	5	132099521	missense	C	A	0.073	p.K137N
3334	*OR2B6*	12367	6	27925134	missense	T	C	0.171	p.I39T
3334	*GPR115*	153838	6	47682045	missense	A	C	0.113	p.K355T
3334	*LRRC1*	18214	6	53787434	missense	G	A	0.225	p.R473K
3334	*PCLO*	33026	7	82545660	missense	G	A	0.168	p.P3881L
3334	*NRCAM*	5010	7	107880468	missense	G	A	0.093	p.A14V
3334	*PDIA4*	4911	7	148718202	missense	C	A	0.091	p.E42D
3334	*ZNF395*	18660	8	28210123	missense	C	T	0.091	p.G296D
3334	*COLEC10*	6438	8	120118317	missense	G	A	0.074	p.D241N
3334	*LOC100506422*	1004352	9	26114415	missense	G	T	0.269	p.R85L
3334	*KCNK4*	33310	11	64067005	missense	C	T	0.154	p.S330F
3334	*RSF1*	16578	11	77413349	missense	T	C	0.200	p.K309E
3334	*ASUN*	18164	12	27077302	missense	T	C	0.227	p.N264D
3334	*CTDSP2*	5730	12	58217763	missense	C	T	0.163	p.R205H
3334	*CTDSP2*	5730	12	58217770	missense	G	T	0.123	p.L203I
3334	*IL22*	20525	12	68646579	missense	G	A	0.063	p.R73C
3334	*OTOGL*	173591	12	80722476	missense	A	T	0.182	p.N1402Y
3334	*HSP90B1*	3299	12	104341190	missense	A	T	0.114	p.E788D
3334	*KCTD4*	198404	13	45768286	missense	C	A	0.051	p.E139D
3334	*SFTA3*	1101341	14	36946284	missense	C	G	0.173	p.K51N
3334	*PPP1R13B*	15316	14	104263737	missense	C	A	0.074	p.C43F
3334	*ZNF106*	22473	15	42727643	missense	C	T	0.220	p.G812E
3334	*ATP8B4*	24837	15	50226220	missense	T	C	0.118	p.S483G
3334	*ALPK3*	20778	15	85400798	missense	G	T	0.321	p.E1145D
3334	*HYDIN*	1270974	16	70942236	missense	A	G	0.270	p.F2772S
3334	*RAI1*	30665	17	17697096	missense	G	C	0.190	p.Q278H
3334	*WNT3*	30753	17	44845945	missense	G	A	0.138	p.T270M
3334	*WNT3*	30753	17	44846063	missense	C	T	0.051	p.G231S
3334	*KIAA1328*	20776	18	34647247	missense	C	T	0.062	p.T324I
3334	*CCDC68*	25214	18	52604147	missense	C	T	0.243	p.V130M
3334	*SNRPA*	4596	19	41265406	missense	G	A	0.078	p.R106H
3334	*ZNF71*	21216	19	57133622	missense	G	A	0.091	p.E323K
3334	*SNPH*	14723	20	1286258	missense	C	T	0.085	p.P349S
3334	*PKIG*	181805	20	43243324	missense	G	A	0.200	p.G43S
3334	*ALG12*	24105	22	50307034	missense	T	C	0.192	p.I98M
3334	*FAM104B*	138362	X	55172686	missense	C	T	0.118	p.S60N
3334	*FAM104B*	138362	X	55172689	missense	G	T	0.071	p.A59E
3334	*TAF1*	138923	X	70618475	missense	G	A	0.133	p.R1224Q
3334	*KLHL13*	33495	X	117043737	missense	G	A	0.073	p.T282M
8170	*EMC1*	15047	1	19561648	stopgain	C	A	0.222	p.E470X
8170	*FEZF2*	18008	3	62358300	stopgain	C	A	0.242	p.E82X
8170	*PLEKHG6*	18173	12	6436519	stopgain	C	A	0.053	p.Y590X
8170	*PDE4DIP*	14644	1	144882693	missense	A	C	0.222	p.L1109R
8170	*C1orf110*	178550	1	162824587	missense	T	G	0.222	p.N293H
8170	*NR4A2*	6186	2	157186023	missense	T	C	0.054	p.K226E
8170	*XIRP2*	152381	2	168107303	missense	A	T	0.143	p.E3134V
8170	*HOXD12*	21193	2	176964806	missense	G	A	0.232	p.G93R
8170	*ZNF804A*	194250	2	185802966	missense	A	T	0.214	p.K948M
8170	*HJURP*	18410	2	234749858	missense	G	A	0.206	p.S523L
8170	*CCDC80*	199512	3	112324500	missense	C	T	0.160	p.G873R
8170	*SHROOM3*	20859	4	77661420	missense	C	A	0.083	p.N698K
8170	*PRKG2*	6259	4	82125772	missense	A	G	0.211	p.F144L
8170	*WDFY3*	14991	4	85741281	missense	C	T	0.261	p.M550I
8170	*IRF2*	2199	4	185320210	missense	T	C	0.231	p.S185G
8170	*FAM193B*	1190946	5	176963483	missense	G	A	0.138	p.P318S
8170	*FAM8A1*	16255	6	17601035	missense	A	G	0.131	p.H132R
8170	*FAM8A1*	16255	6	17601040	missense	G	A	0.138	p.G134S
8170	*FAM8A1*	16255	6	17601041	missense	G	T	0.138	p.G134V
8170	*FAM8A1*	16255	6	17601044	missense	T	C	0.143	p.L135P

8170	*FAM8A1*	16255	6	17601058	missense	G	A	0.167	p.A140T
8170	*FAM8A1*	16255	6	17601086	missense	A	G	0.104	p.Q149R
8170	*URGCP*	17920	7	43916996	missense	C	A	0.077	p.R646L
8170	*RBM33*	53043	7	155504093	missense	C	G	0.213	p.P382R
8170	*XKR5*	207411	8	6669536	missense	C	T	0.302	p.G252E
8170	*DUSP26*	24025	8	33451125	missense	G	A	0.295	p.S121L
8170	*PLEC*	201384	8	144993140	missense	G	A	0.161	p.R3644C
8170	*VWA2*	1272046	10	116045791	missense	T	A	0.312	p.V364D
8170	*TSPAN18*	130783	11	44931338	missense	C	G	0.257	p.P49R
8170	*CHD4*	1297553	12	6710853	missense	C	T	0.143	p.R173Q
8170	*RERG*	32918	12	15274026	missense	C	T	0.154	p.R30Q
8170	*CTDSP2*	5730	12	58217763	missense	C	T	0.221	p.R205H
8170	*CTDSP2*	5730	12	58220819	missense	A	G	0.154	p.V105A
8170	*CTDSP2*	5730	12	58220831	missense	C	G	0.200	p.R101T
8170	*SOX1*	5986	13	112722212	missense	G	T	0.267	p.E80D
8170	*WDR89*	80666	14	64066395	missense	C	T	0.200	p.C89Y
8170	*WDR89*	80666	14	64066398	missense	G	C	0.226	p.A88G
8170	*WDR89*	80666	14	64066402	missense	A	G	0.226	p.S87P
8170	*CEP170B*	15005	14	105349647	missense	G	A	0.320	p.D285N
8170	*HYDIN*	1270974	16	70913372	missense	C	T	0.136	p.R3462Q
8170	*SLC46A1*	80669	17	26731702	missense	C	T	0.114	p.G338D
8170	*KIF2B*	32559	17	51901403	missense	T	A	0.182	p.F337I
8170	*RNF213*	1256071	17	78313316	missense	A	G	0.279	p.R1717G
8170	*KIAA1683*	25249	19	18377556	missense	G	A	0.289	p.T265I
8170	*ZNF573*	152360	19	38230261	missense	T	C	0.227	p.Q319R
8170	*PTOV1*	17432	19	50363244	missense	C	T	0.069	p.A348V
8170	*FPR2*	1462	19	52272390	missense	T	G	0.231	p.V160G
8170	*CDC25B*	21873	20	3781963	missense	C	A	0.111	p.F256L
8170	*DLGAP4*	183006	20	35125345	missense	C	A	0.055	p.A629D
8170	*FAM104B*	1166703	X	55172521	missense	G	T	0.061	p.T114K
8173	*TTC39A*	1297667	1	51753881	missense	T	C	0.100	p.K597R
8173	*TNN*	22093	1	175048576	missense	G	A	0.182	p.G173R
8173	*ARL8A*	138795	1	202107501	missense	T	A	0.105	p.I67F
8173	*USP40*	18218	2	234394436	missense	C	T	0.323	p.E1140K
8173	*ZBTB2*	20861	6	151687729	missense	C	A	0.138	p.D158Y
8173	*ANKMY2*	20319	7	16642097	missense	C	G	0.211	p.W350S
8173	*UPK3B*	182684	7	76144462	missense	G	A	0.116	p.A258T
8173	*NEFL*	6158	8	24813869	missense	C	T	0.265	p.R54H
8173	*PXDNL*	144651	8	52321782	missense	C	T	0.114	p.R801H
8173	*ZHX2*	14943	8	123966106	missense	G	A	0.078	p.E786K
8173	*ZNF34*	30580	8	145998654	missense	C	A	0.089	p.M560I
8173	*C9orf47*	1142413	9	91606466	missense	C	A	0.105	p.P91T
8173	*PITPNM1*	4910	11	67264870	missense	G	T	0.111	p.P660T
8173	*IGHMBP2*	2180	11	68700804	missense	C	A	0.211	p.R425S
8173	*CBL*	5188	11	119170416	missense	G	C	0.089	p.Q882H
8173	*DHH*	21044	12	49488109	missense	C	A	0.091	p.A63S
8173	*CTDSP2*	5730	12	58217698	missense	G	T	0.101	p.P227T
8173	*CTDSP2*	5730	12	58217763	missense	C	T	0.241	p.R205H
8173	*CTDSP2*	5730	12	58220811	missense	G	T	0.135	p.L108I
8173	*CTDSP2*	5730	12	58220819	missense	A	G	0.171	p.V105A
8173	*RYR3*	1243996	15	34077881	missense	C	T	0.148	p.T3096M
8173	*CCDC42*	144681	17	8644794	missense	C	T	0.083	p.E164K
8173	*CCDC47*	20198	17	61843370	missense	G	A	0.227	p.R56W
8173	*ST8SIA3*	15879	18	55027261	missense	T	C	0.182	p.L299P
8173	*ZNF414*	32370	19	8577341	missense	C	T	0.087	p.E154K
8173	*MYO9B*	4145	19	17273175	missense	G	T	0.143	p.D479Y
8173	*PSENEN*	172341	19	36237617	missense	C	A	0.083	p.R59S
8173	*DNMT3B*	175850	20	31380479	missense	C	G	0.075	p.D335E
8173	*MYH7B*	20884	20	33573984	missense	G	A	0.114	p.R338K
8173	*NOL12*	24313	22	38087309	missense	G	A	0.077	p.R203H
8173	*ZBED4*	14838	22	50279073	missense	G	A	0.073	p.R588Q
8173	*FAM104B*	1166703	X	55172521	missense	G	T	0.056	p.T114K
8173	*FAM104B*	138362	X	55172686	missense	C	T	0.295	p.S60N
8173	*FAM104B*	138362	X	55172689	missense	G	T	0.295	p.A59E
8173	*FAM155B*	15686	X	68725798	missense	A	C	0.077	p.M225L
8178	*ZNF148*	21964	3	124951522	stopgain	G	T	0.111	p.S683X
8178	*NPHP4*	15102	1	5924014	missense	C	T	0.080	p.R1359Q
8178	*MAP3K6*	4672	1	27683892	missense	G	A	0.051	p.A1086V
8178	*GRIK3*	831	1	37307363	missense	C	T	0.051	p.G502S
8178	*GNL2*	13285	1	38053006	missense	C	A	0.152	p.D159Y
8178	*MAST2*	15112	1	46489620	missense	G	T	0.065	p.R583L
8178	*CACHD1*	20925	1	65141112	missense	G	A	0.065	p.S868N
8178	*SMG7*	201569	1	183522153	missense	G	T	0.070	p.Q1118H
8178	*C2orf16*	32266	2	27802410	missense	T	A	0.085	p.S991T
8178	*FBXO11*	25133	2	48059946	missense	G	A	0.093	p.P288L
8178	*UGT1A1*	463	2	234669666	missense	C	G	0.051	p.Q245E
8178	*DSPP*	14208	4	88537243	missense	C	A	0.204	p.D1143E
8178	*FAM81B*	152548	5	94784119	missense	A	T	0.174	p.E392D
8178	*JADE2*	15288	5	133909450	missense	G	A	0.051	p.R517Q
8178	*DSP*	4415	6	7583880	missense	A	C	0.121	p.I2129L
8178	*MUC22*	1198815	6	30997575	missense	C	A	0.075	p.T1456N

8178	*MUC17*	1040105	7	100675423	missense	A	C	0.098	p.E242D
8178	*CPA6*	20361	8	68396943	missense	C	T	0.111	p.V240I
8178	*PHF20L1*	16018	8	133851694	missense	G	A	0.093	p.G752R
8178	*EPPK1*	31308	8	144943229	missense	T	A	0.054	p.K1398M
8178	*AFAP1L2*	32550	10	116056820	missense	G	T	0.083	p.P836T
8178	*CTBP2*	22802	10	126692019	missense	G	A	0.087	p.P34L
8178	*HPX*	613	11	6461433	missense	C	T	0.065	p.A100T
8178	*ZBED5*	21211	11	10875889	missense	A	C	0.075	p.Y202D
8178	*CTDSP2*	5730	12	58217763	missense	C	T	0.175	p.R205H
8178	*CTDSP2*	5730	12	58217770	missense	G	C	0.078	p.L203V
8178	*CTDSP2*	5730	12	58220831	missense	C	G	0.162	p.R101T
8178	*DCN*	133507	12	91572272	missense	G	C	0.085	p.Q20E
8178	*NIPA2*	30922	15	23006777	missense	G	T	0.075	p.T176K
8178	*ZNF646*	14699	16	31089060	missense	G	A	0.053	p.R472H
8178	*CDH3*	1793	16	68716208	missense	G	A	0.085	p.E334K
8178	*MFSD11*	24311	17	74735075	missense	G	A	0.111	p.S51N
8178	*RNF213*	1256071	17	78350182	missense	G	A	0.087	p.V4423M
8178	*CCDC57*	198082	17	80159490	missense	T	C	0.065	p.R111G
8178	*SETBP1*	15559	18	42532709	missense	C	A	0.051	p.P1135H
8178	*RYR1*	540	19	39014559	missense	G	T	0.074	p.G3482V
8178	*GRIN2D*	836	19	48917724	missense	T	C	0.105	p.L432P
8178	*NUCB1*	6184	19	49404175	missense	C	T	0.050	p.A41V
8178	*ZNF616*	178523	19	52619155	missense	C	T	0.114	p.R421H
8178	*TPST2*	3595	22	26936774	missense	C	T	0.057	p.G275S
8181	*CYP20A1*	177538	2	204131263	stopgain	G	A	0.158	p.W153X
8181	*FAT3*	1008781	11	92086907	stopgain	C	G	0.132	p.Y543X
8181	*RAPGEFL1*	16339	17	38340881	stopgain	C	T	0.161	p.R56X
8181	*LRRC7*	20794	1	70504679	missense	A	G	0.191	p.K1020E
8181	*GEN1*	182625	2	17961322	missense	G	A	0.067	p.A448T
8181	*LOC728819*	1101330	2	43902548	missense	A	G	0.081	p.V305A
8181	*TTN*	133378	2	179579126	missense	G	T	0.182	p.A8792E
8181	*NMUR1*	6056	2	232393181	missense	G	A	0.248	p.A184V
8181	*STAC*	3149	3	36484906	missense	C	A	0.219	p.S54R
8181	*SLC22A13*	4256	3	38307470	missense	T	A	0.150	p.V40D
8181	*SCN11A*	14139	3	38888425	missense	C	T	0.062	p.M1712I
8181	*SLC26A6*	134426	3	48665879	missense	C	A	0.167	p.K596N
8181	*RASSF1*	170714	3	50375431	missense	T	G	0.083	p.T92P
8181	*ZNF717*	1290209	3	75788230	missense	C	T	0.097	p.V132I
8181	*GSK3B*	2093	3	119582276	missense	G	T	0.053	p.F375L
8181	*CHST13*	152889	3	126261189	missense	G	A	0.069	p.G265D
8181	*RHO*	539	3	129247854	missense	G	C	0.056	p.S93T
8181	*RHO*	539	3	129249830	missense	C	T	0.062	p.A158V
8181	*RHO*	539	3	129249841	missense	G	A	0.068	p.V162I
8181	*RHO*	539	3	129251457	missense	G	T	0.109	p.A260S
8181	*RHO*	539	3	129251458	missense	C	T	0.109	p.A260V
8181	*RHO*	539	3	129251475	missense	G	C	0.105	p.V266L
8181	*ZIC1*	3412	3	147131192	missense	G	A	0.208	p.G400S
8181	*GUF1*	21927	4	44688015	missense	C	A	0.058	p.Q237K
8181	*FRAS1*	25074	4	79366841	missense	T	G	0.152	p.I1944S
8181	*FRAS1*	25074	4	79366855	missense	A	T	0.143	p.I1949F
8181	*ASIC5*	17419	4	156763420	missense	C	A	0.057	p.L316F
8181	*EXOC3*	7277	5	459572	missense	C	A	0.051	p.S463R
8181	*GPR98*	32119	5	90079810	missense	C	T	0.113	p.P4530L
8181	*SYCP2L*	1040274	6	10955396	missense	G	A	0.145	p.E668K
8181	*BTN2A1*	78476	6	26463551	missense	G	C	0.174	p.W170C
8181	*HTR1B*	863	6	78172069	missense	A	G	0.176	p.F351S
8181	*MAS1*	2377	6	160328915	missense	C	T	0.069	p.R310W
8181	*GIGYF1*	22574	7	100283069	missense	C	T	0.114	p.E364K
8181	*RALYL*	173848	8	85799868	missense	C	G	0.176	p.L239V
8181	*CNGB3*	19098	8	87680379	missense	G	A	0.184	p.P171S
8181	*MAPK15*	139021	8	144801025	missense	C	T	0.106	p.R123W
8181	*UNC13B*	6377	9	35382377	missense	A	C	0.173	p.K811T
8181	*TMOD1*	3275	9	100325079	missense	A	G	0.235	p.I155V
8181	*ITGA8*	3638	10	15649813	missense	G	T	0.167	p.Q543K
8181	*CTBP2*	22802	10	126686646	missense	C	G	0.103	p.W151S
8181	*CTBP2*	22802	10	126686649	missense	G	C	0.100	p.T150R
8181	*CTBP2*	22802	10	126686656	missense	T	C	0.098	p.R148G
8181	*CTBP2*	22802	10	126686659	missense	G	A	0.091	p.R147W
8181	*OR51E2*	30774	11	4703451	missense	C	T	0.143	p.R164Q
8181	*ORAOV1*	153451	11	69482763	missense	A	G	0.088	p.L82S
8181	*CTDSP2*	5730	12	58217698	missense	G	T	0.104	p.P227T
8181	*CTDSP2*	5730	12	58217770	missense	G	T	0.068	p.L203I
8181	*CTDSP2*	5730	12	58220801	missense	G	T	0.067	p.T111N
8181	*CTDSP2*	5730	12	58220811	missense	G	T	0.074	p.L108I
8181	*CTDSP2*	5730	12	58220831	missense	C	G	0.084	p.R101T
8181	*CTDSP2*	5730	12	58240188	missense	G	A	0.149	p.R11W
8181	*WDR89*	80666	14	64066352	missense	T	A	0.052	p.R103S
8181	*WDR89*	80666	14	64066395	missense	C	T	0.065	p.C89Y
8181	*WDR89*	80666	14	64066398	missense	G	C	0.065	p.A88G
8181	*WDR89*	80666	14	64066402	missense	A	G	0.064	p.S87P
8181	*ATXN3*	30660	14	92537397	missense	T	C	0.066	p.T130A

8181	*FRMD5*	32892	15	44487176	missense	T	C	0.193	p.D26G
8181	*SKOR1*	1258024	15	68114480	missense	G	A	0.160	p.R81K
8181	*CAPN15*	5632	16	602977	missense	A	C	0.225	p.S1007R
8181	*HYDIN*	1270974	16	71052037	missense	C	G	0.200	p.Q1213H
8181	*MTCL1*	15210	18	8825233	missense	C	A	0.193	p.S1242Y
8181	*CCDC178*	198995	18	30847231	missense	G	C	0.180	p.L403V
8181	*RGL3*	1161616	19	11517249	missense	C	T	0.183	p.R281Q
8181	*ZNF441*	152355	19	11891996	missense	G	A	0.077	p.G453R
8181	*ANKLE1*	1278444	19	17397489	missense	G	T	0.178	p.V641L
8181	*SLC7A9*	14270	19	33333154	missense	C	T	0.109	p.A382T
8181	*ZNF283*	181845	19	44351795	missense	A	G	0.091	p.K348E
8181	*KIR2DL3*	15868	19	55263673	missense	G	T	0.073	p.V276F
8181	*PXMP4*	7238	20	32298434	missense	G	A	0.144	p.P101L
8181	*ADRM1*	175573	20	60883218	missense	C	T	0.077	p.S333L
8181	*STS*	351	X	7171256	missense	C	T	0.102	p.L11F
8181	*MAGEB4*	2367	X	30260818	missense	A	G	0.172	p.Q189R
8181	*FAM104B*	1166703	X	55172521	missense	G	T	0.099	p.T114K
8181	*FAM104B*	138362	X	55172686	missense	C	T	0.208	p.S60N
8181	*FAM104B*	138362	X	55172689	missense	G	T	0.101	p.A59E
8181	*NLGN3*	181303	X	70387614	missense	T	C	0.208	p.V556A
8181	*DACH2*	53281	X	85969670	missense	G	T	0.173	p.A351S
8181	*GLRA4*	1024452	X	102968516	missense	T	A	0.130	p.I339L
8181	*SPANXN2*	1009615	X	142795149	missense	C	A	0.054	p.G177C
8185	*UBR4*	20765	1	19404482	stopgain	G	T	0.073	p.Y5104X
8185	*MCTP1*	24717	5	94248656	stopgain	G	C	0.250	p.S459X
8185	*PCDHGB2*	32096	5	140741230	stopgain	C	T	0.148	p.Q510X
8185	*PABPC1*	2568	8	101724951	stopgain	T	A	0.116	p.K269X
8185	*CMA1*	1836	14	24975387	stopgain	C	T	0.126	p.W149X
8185	*USP48*	32236	1	22050432	missense	T	C	0.102	p.D536G
8185	*EPHA10*	173641	1	38230692	missense	C	T	0.217	p.R16Q
8185	*ZBTB7B*	1256455	1	154987158	missense	C	G	0.206	p.L42V
8185	*ACTA1*	1100	1	229567918	missense	C	T	0.130	p.V211M
8185	*MYT1L*	15025	2	1920982	missense	G	A	0.154	p.P536L
8185	*DTNB*	183361	2	25799783	missense	C	A	0.132	p.C267F
8185	*PREB*	13388	2	27355188	missense	G	A	0.220	p.P279L
8185	*TTN*	133437	2	179408266	missense	C	T	0.152	p.R32145H
8185	*FSIP2*	173651	2	186672147	missense	C	G	0.138	p.D6127E
8185	*UGT1A6*	1072	2	234601805	missense	G	A	0.134	p.R52Q
8185	*IQCA1*	24726	2	237253244	missense	T	C	0.136	p.T638A
8185	*USP19*	6677	3	49153065	missense	G	C	0.194	p.P555R
8185	*PHLDB2*	145753	3	111632416	missense	G	A	0.122	p.S556N
8185	*RBM46*	144979	4	155719352	missense	A	T	0.061	p.T181S
8185	*PCDHB1*	13340	5	140431527	missense	C	G	0.113	p.Q158E
8185	*ZBED8*	22090	5	159820951	missense	A	C	0.131	p.F516C
8185	*BMP6*	1718	6	7727493	missense	C	T	0.094	p.P102L
8185	*FAM8A1*	16255	6	17601086	missense	A	G	0.077	p.Q149R
8185	*SGK1*	1143676	6	134583262	missense	T	A	0.118	p.M32L
8185	*GRM3*	840	7	86468673	missense	T	C	0.126	p.C615R
8185	*TAS2R5*	18980	7	141490378	missense	C	G	0.076	p.Q73E
8185	*PRKACG*	2732	9	71628351	missense	C	T	0.138	p.V220M
8185	*CUBN*	1081	10	16958017	missense	C	T	0.103	p.G2338E
8185	*KIAA1217*	19590	10	24762598	missense	C	T	0.138	p.R350W
8185	*TET1*	30625	10	70333419	missense	A	G	0.138	p.T442A
8185	*ZNF503*	32772	10	77159075	missense	G	A	0.262	p.A458V
8185	*CTBP2*	22802	10	126686649	missense	G	C	0.161	p.T150R
8185	*CTBP2*	22802	10	126686656	missense	T	C	0.152	p.R148G
8185	*CTBP2*	22802	10	126686659	missense	G	A	0.182	p.R147W
8185	*OR51B5*	1005567	11	5364465	missense	G	C	0.127	p.S97C
8185	*OR10AG1*	1005491	11	55735638	missense	G	A	0.173	p.T101M
8185	*CTDSP2*	5730	12	58217698	missense	G	T	0.063	p.P227T
8185	*CTDSP2*	5730	12	58217770	missense	G	C	0.084	p.L203V
8185	*CTDSP2*	5730	12	58220811	missense	G	T	0.070	p.L108I
8185	*CTDSP2*	5730	12	58220819	missense	A	G	0.104	p.V105A
8185	*CTDSP2*	5730	12	58220831	missense	C	G	0.063	p.R101T
8185	*CTDSP2*	5730	12	58240188	missense	G	A	0.140	p.R11W
8185	*PABPC3*	30979	13	25670691	missense	G	T	0.098	p.V119F
8185	*PABPC3*	30979	13	25670703	missense	G	T	0.098	p.G123C
8185	*PABPC3*	30979	13	25670712	missense	C	G	0.085	p.L126V
8185	*NBEA*	15678	13	36220460	missense	C	T	0.132	p.P2561L
8185	*CCDC168*	1146197	13	103388246	missense	T	A	0.143	p.K4934M
8185	*EFNB2*	4093	13	107187260	missense	A	G	0.103	p.V18A
8185	*FBN1*	138	15	48729575	missense	C	T	0.145	p.R2108H
8185	*MYLK3*	182493	16	46746582	missense	C	A	0.066	p.V698L
8185	*GNAO1*	138736	16	56309857	missense	A	T	0.158	p.D59V
8185	*TP53*	1276761	17	7577097	missense	C	G	0.121	p.D242H
8185	*LRRC3C*	1195545	17	38100199	missense	C	T	0.084	p.P14S
8185	*CCBE1*	133459	18	57136726	missense	G	A	0.182	p.R127W
8185	*PLIN4*	1080400	19	4511861	missense	A	G	0.051	p.L690P
8185	*MUC16*	24690	19	9072268	missense	G	A	0.129	p.P5060S
8185	*COL5A3*	15719	19	10112330	missense	T	C	0.208	p.D327G
8185	*ZSWIM4*	23072	19	13919661	missense	C	T	0.153	p.P242S

8185	*MARK4*	31417	19	45781804	missense	G	C	0.054	p.G313R
8185	*LILRB4*	1278429	19	55178163	missense	A	G	0.120	p.N336S
8185	*LILRB4*	1278429	19	55178164	missense	C	G	0.122	p.N336K
8185	*MZF1*	198055	19	59074322	missense	C	T	0.162	p.R441H
8185	*PTPRT*	133170	20	40979297	missense	T	A	0.164	p.K612N
8185	*TOB2*	16272	22	41832436	missense	A	T	0.099	p.F305Y
8185	*FAM104B*	138362	X	55172686	missense	C	T	0.154	p.S60N
8185	*FAM104B*	138362	X	55172689	missense	G	T	0.085	p.A59E
8185	*TNMD*	22144	X	99848988	missense	G	A	0.148	p.G93R
8185	*GPRASP1*	14710	X	101912313	missense	G	A	0.083	p.E1158K
8185	*IRS4*	3604	X	107975824	missense	C	T	0.077	p.D1251N
8185	*AGTR2*	686	X	115304551	missense	C	T	0.157	p.L340F
8185	*PGRMC1*	6667	X	118370610	missense	G	T	0.101	p.G95V
8192	*ATF7IP*	181352	12	14613566	stopgain	C	T	0.286	p.Q774X
8192	*LPPR4*	14839	1	99753615	missense	G	A	0.222	p.R106Q
8192	*TARS2*	25150	1	150471769	missense	G	C	0.227	p.K539N
8192	*B3GALT2*	3783	1	193149636	missense	T	C	0.063	p.I353V
8192	*NAGK*	17567	2	71297886	missense	A	C	0.074	p.K90Q
8192	*IMMT*	6839	2	86371761	missense	T	C	0.258	p.Y636C
8192	*LMAN2L*	30805	2	97403694	missense	T	C	0.237	p.N100D
8192	*HOXD8*	19558	2	176996254	missense	G	A	0.161	p.V79I
8192	*NFE2L2*	6164	2	178097161	missense	C	T	0.300	p.E185K
8192	*COL5A2*	393	2	189923194	missense	C	G	0.147	p.E730D
8192	*KALRN*	3947	3	123813728	missense	G	A	0.228	p.R15H
8192	*SLC7A14*	20949	3	170185163	missense	G	T	0.133	p.L666M
8192	*KIAA0232*	14743	4	6863846	missense	A	C	0.079	p.L579F
8192	*KIAA0232*	14743	4	6864121	missense	A	G	0.080	p.N671S
8192	*KIAA0232*	14743	4	6864126	missense	A	C	0.098	p.N673H
8192	*KIAA0232*	14743	4	6864129	missense	A	G	0.102	p.T674A
8192	*ANKRD17*	198889	4	74123997	missense	G	A	0.129	p.S130F
8192	*FRAS1*	25074	4	79343047	missense	C	T	0.306	p.S1524F
8192	*ANKRD50*	20337	4	125592212	missense	T	G	0.192	p.E740D
8192	*PCDH18*	19035	4	138453137	missense	C	T	0.217	p.E36K
8192	*SREK1*	139168	5	65466631	missense	A	T	0.302	p.K331I
8192	*GPR151*	194251	5	145894655	missense	C	G	0.200	p.G341A
8192	*UBLCP1*	145049	5	158697369	missense	A	C	0.206	p.E83A
8192	*OR11A1*	13937	6	29395328	missense	T	A	0.174	p.I31F
8192	*KIAA1919*	153369	6	111587684	missense	A	G	0.070	p.T307A
8192	*KIAA1919*	153369	6	111587691	missense	C	G	0.059	p.S309C
8192	*KIAA1919*	153369	6	111587695	missense	A	C	0.076	p.L310F
8192	*SEMA3E*	12431	7	83032052	missense	A	G	0.118	p.S347P
8192	*CLDN12*	12129	7	90042138	missense	G	A	0.068	p.V50I
8192	*RBM28*	18077	7	127977208	missense	T	C	0.250	p.D197G
8192	*CSMD1*	33225	8	2967743	missense	T	C	0.114	p.H2182R
8192	*CA1*	1738	8	86244763	missense	T	G	0.200	p.K157Q
8192	*RBM12B*	203390	8	94748223	missense	T	A	0.071	p.H139L
8192	*WISP1*	80838	8	134239843	missense	C	T	0.161	p.R332C
8192	*PTPDC1*	177995	9	96860265	missense	T	G	0.273	p.L419V
8192	*FAM170B*	1164484	10	50340202	missense	G	A	0.133	p.P103L
8192	*ANKRD1*	14391	10	92675373	missense	G	A	0.154	p.A259V
8192	*DMBT1*	17579	10	124396713	missense	C	T	0.143	p.S2147F
8192	*PCF11*	15885	11	82875436	missense	C	T	0.229	p.A232V
8192	*FZD4*	12193	11	86663373	missense	C	G	0.118	p.S142T
8192	*FZD4*	12193	11	86663375	missense	C	G	0.114	p.E141D
8192	*CTDSP2*	5730	12	58217763	missense	C	T	0.146	p.R205H
8192	*CTDSP2*	5730	12	58220819	missense	A	G	0.082	p.V105A
8192	*DNAH10*	207437	12	124387611	missense	G	A	0.200	p.A3138T
8192	*GPR133*	198827	12	131590337	missense	A	T	0.250	p.H605L
8192	*CPNE6*	6032	14	24546421	missense	G	C	0.233	p.R508P
8192	*SRP54*	3136	14	35498276	missense	C	T	0.138	p.A478V
8192	*HEATR4*	203309	14	73945333	missense	C	A	0.182	p.G1020V
8192	*RIN3*	24832	14	93118397	missense	C	A	0.138	p.Q335K
8192	*MYO5A*	1142495	15	52606035	missense	C	T	0.174	p.R1809Q
8192	*LDHAL6B*	33195	15	59500155	missense	G	A	0.222	p.S339N
8192	*SCAPER*	20843	15	76673745	missense	C	T	0.250	p.A981T
8192	*NETO2*	18092	16	47162398	missense	C	T	0.111	p.V107I
8192	*WDR59*	30581	16	74949892	missense	G	T	0.214	p.A367D
8192	*STAT3*	213662	17	40474420	missense	C	A	0.167	p.D661Y
8192	*HPN*	182983	19	35551061	missense	G	A	0.095	p.A148T
8192	*ZNF235*	4234	19	44793338	missense	G	T	0.225	p.Q84K
8192	*RIN2*	18993	20	19870249	missense	A	T	0.108	p.T51S
8192	*RBM12*	152838	20	34243109	missense	C	T	0.063	p.V46I
8192	*BAGE2*	182482	21	11098708	missense	C	T	0.089	p.G4R
8192	*ZBED4*	14838	22	50279349	missense	G	C	0.098	p.G680A
8195	*SLC30A6*	17964	2	32445309	stopgain	C	T	0.085	p.R345X
8195	*SIN3A*	15477	15	75692392	stopgain	C	A	0.250	p.E615X
8195	*SIN3A*	15477	15	75722530	stopgain	G	A	0.229	p.Q63X
8195	*CNKSR2*	14927	X	21450853	stopgain	C	T	0.229	p.R118X
8195	*PLEKHG5*	198681	1	6533164	missense	G	A	0.154	p.P366L
8195	*PPP2R5A*	6243	1	212530307	missense	G	A	0.111	p.E332K
8195	*PPP2R5A*	6243	1	212530318	missense	A	T	0.096	p.E335D

8195	*PPP2R5A*	6243	1	212530319	missense	A	G	0.094	p.I336V
8195	*FMN2*	20066	1	240255656	missense	A	T	0.203	p.N83Y
8195	*PCGF1*	32673	2	74732471	missense	C	T	0.263	p.G243S
8195	*CNTNAP5*	130773	2	125555750	missense	A	C	0.057	p.S1023R
8195	*SCRN3*	24583	2	175265866	missense	G	A	0.281	p.A164T
8195	*FSIP2*	173651	2	186673660	missense	G	T	0.250	p.V6632F
8195	*CPS1*	1875	2	211507213	missense	A	G	0.235	p.S995G
8195	*CMTM8*	178868	3	32398913	missense	C	T	0.238	p.R66W
8195	*SCN5A*	198056	3	38592776	missense	G	C	0.148	p.T1696S
8195	*PDZRN3*	15009	3	73433320	missense	T	G	0.118	p.E799D
8195	*ZNF717*	1290209	3	75787351	missense	C	A	0.217	p.G425W
8195	*MORC1*	14429	3	108751597	missense	C	T	0.231	p.C512Y
8195	*CPNE4*	153429	3	131442346	missense	G	T	0.235	p.H120N
8195	*TMPRSS11A*	182606	4	68777128	missense	C	T	0.065	p.D400N
8195	*UNC5C*	3728	4	96091432	missense	T	C	0.281	p.T835A
8195	*DNAH5*	1369	5	13844999	missense	C	A	0.227	p.A1740S
8195	*FABP7*	1446	6	123101502	missense	G	A	0.241	p.G47E
8195	*SNX9*	16224	6	158327174	missense	C	T	0.111	p.A212V
8195	*GRM3*	840	7	86468998	missense	G	T	0.066	p.R723L
8195	*ASZ1*	130768	7	117066904	missense	T	A	0.271	p.E64V
8195	*WWP1*	7013	8	87447715	missense	G	A	0.174	p.G546S
8195	*SLC24A2*	20344	9	19521052	missense	C	G	0.296	p.E526Q
8195	*RRP12*	15179	10	99160183	missense	G	A	0.089	p.A83V
8195	*CTR9*	14633	11	10791826	missense	C	G	0.286	p.L727V
8195	*KIF18A*	31217	11	28119340	missense	C	A	0.232	p.S52I
8195	*CBL*	5188	11	119170416	missense	G	C	0.057	p.Q882H
8195	*CTDSP2*	5730	12	58217698	missense	G	T	0.096	p.P227T
8195	*CTDSP2*	5730	12	58217770	missense	G	C	0.132	p.L203V
8195	*ESD*	1984	13	47356837	missense	T	C	0.086	p.T116A
8195	*ESD*	1984	13	47356861	missense	C	T	0.083	p.D108N
8195	*IPO5*	2271	13	98670793	missense	G	A	0.062	p.V909I
8195	*SAMD4A*	15589	14	55169062	missense	G	A	0.055	p.G160D
8195	*BTBD7*	1289133	14	93712523	missense	A	G	0.227	p.M744T
8195	*RNF111*	17610	15	59323686	missense	C	T	0.172	p.S222L
8195	*SMG1*	15092	16	18840853	missense	G	C	0.258	p.Q3120E
8195	*NLK*	16231	17	26369915	missense	G	A	0.125	p.A6T
8195	*PIGS*	33198	17	26881281	missense	A	T	0.095	p.L542Q
8195	*KIAA1468*	20854	18	59912099	missense	G	A	0.303	p.D575N
8195	*SERPINB8*	198833	18	61652456	missense	G	A	0.250	p.D233N
8195	*OR7A5*	17506	19	14938494	missense	A	T	0.265	p.L187H
8195	*NLRP5*	153447	19	56538759	missense	C	T	0.273	p.P387L
8195	*KIF16B*	24704	20	16360560	missense	C	G	0.340	p.R696T
8195	*ID1*	181353	20	30193624	missense	C	G	0.333	p.S145C
8195	*CECR2*	1290047	22	18018397	missense	C	G	0.129	p.P286A
8195	*ZBED4*	14838	22	50279333	missense	T	C	0.106	p.F675L
8195	*ZBED4*	14838	22	50279349	missense	G	C	0.098	p.G680A
8195	*DCAF8L2*	1136533	X	27765263	missense	T	C	0.125	p.L84P
8195	*CHDC2*	173695	X	36162684	missense	C	G	0.154	p.L423V
8195	*FAM104B*	138362	X	55172686	missense	C	T	0.196	p.S60N
8195	*FAM104B*	138362	X	55172689	missense	G	T	0.220	p.A59E
8195	*MECP2*	4992	X	153296388	missense	C	A	0.093	p.Q297H
8203	*MICAL2*	14632	11	12247761	stopgain	C	T	0.291	p.Q578X
8203	*TXNIP*	6472	1	145440407	missense	G	A	0.273	p.R238K
8203	*AAK1*	14911	2	69757827	missense	A	G	0.242	p.L223P
8203	*KLHL23*	144711	2	170606208	missense	G	A	0.311	p.R548Q
8203	*GAD1*	817	2	171716363	missense	G	A	0.295	p.E586K
8203	*TTN*	133437	2	179641235	missense	C	A	0.058	p.D1786Y
8203	*COL3A1*	90	2	189859520	missense	C	T	0.238	p.P473L
8203	*ZNF717*	1290209	3	75786765	missense	G	A	0.091	p.T620M
8203	*ZNF717*	1290209	3	75786930	missense	C	A	0.114	p.G565V
8203	*ZNF717*	1290209	3	75787269	missense	G	T	0.078	p.T452K
8203	*ZNF717*	1290209	3	75788230	missense	C	T	0.134	p.V132I
8203	*GABRA4*	1204266	4	46973232	missense	C	G	0.242	p.V229L
8203	*ZNF608*	20747	5	124080678	missense	G	A	0.330	p.S2L
8203	*ADAMTS19*	133638	5	128862044	missense	A	T	0.238	p.L321F
8203	*RBPMS*	6867	8	30242615	missense	A	G	0.098	p.N3D
8203	*RBPMS*	6867	8	30242616	missense	A	G	0.098	p.N3S
8203	*ACTL7B*	6686	9	111617804	missense	G	A	0.309	p.T136I
8203	*ITIH5*	32817	10	7605079	missense	C	A	0.051	p.M932I
8203	*PLCE1*	16341	10	95987090	missense	C	T	0.275	p.L613F
8203	*DYNC2H1*	1377	11	103106426	missense	C	T	0.328	p.A3198V
8203	*SORL1*	3105	11	121348861	missense	A	G	0.314	p.K146R
8203	*KCNJ5*	890	11	128781800	missense	G	A	0.088	p.R211Q
8203	*CTDSP2*	5730	12	58217396	missense	G	A	0.207	p.R269W
8203	*CTDSP2*	5730	12	58217398	missense	A	T	0.200	p.L268Q
8203	*CTDSP2*	5730	12	58217698	missense	G	T	0.135	p.P227T
8203	*CTDSP2*	5730	12	58217763	missense	C	T	0.276	p.R205H
8203	*CTDSP2*	5730	12	58217770	missense	G	T	0.223	p.L203I
8203	*CTDSP2*	5730	12	58220801	missense	G	T	0.100	p.T111N
8203	*CTDSP2*	5730	12	58220811	missense	G	T	0.164	p.L108I
8203	*CTDSP2*	5730	12	58220831	missense	C	G	0.141	p.R101T

8203	*NAV3*	14903	12	78582402	missense	G	T	0.279	p.R1967L
8203	*AGBL1*	152336	15	86807937	missense	A	G	0.059	p.Y466C
8203	*KSR1*	14238	17	25930880	missense	G	A	0.111	p.A396T
8203	*RNF213*	1256071	17	78320174	missense	C	T	0.271	p.P2680L
8203	*DTNA*	32979	18	32391989	missense	G	A	0.265	p.R172Q
8203	*ZNF776*	173632	19	58265723	missense	T	C	0.131	p.C409R
8203	*FAM104B*	138362	X	55172686	missense	C	T	0.051	p.S60N
8203	*ELF4*	1421	X	129215253	missense	C	T	0.120	p.G18R
8204	*OR52A1*	12375	11	5173263	stopgain	C	A	0.146	p.E113X
8204	*SLC6A5*	4211	11	20639423	stopgain	C	G	0.225	p.S418X
8204	*SOX11*	3108	2	5834153	missense	T	A	0.171	p.F434I
8204	*COL3A1*	90	2	189876394	missense	G	A	0.128	p.R1432Q
8204	*COL3A1*	90	2	189876408	missense	G	A	0.109	p.V1437M
8204	*COL3A1*	90	2	189876423	missense	G	A	0.064	p.V1442I
8204	*COL3A1*	90	2	189876477	missense	G	A	0.087	p.V1460I
8204	*CLK1*	4071	2	201718078	missense	C	T	0.070	p.R469K
8204	*CLK1*	4071	2	201718120	missense	T	C	0.063	p.Q455R
8204	*CNTN6*	14461	3	1415314	missense	C	A	0.221	p.Q605K
8204	*ZNF717*	1290209	3	75788230	missense	C	T	0.117	p.V132I
8204	*ATP6V1A*	1690	3	113524247	missense	G	T	0.069	p.A546S
8204	*MECOM*	5241	3	168834191	missense	C	T	0.167	p.S490N
8204	*IGFBP7*	1553	4	57907042	missense	T	A	0.094	p.Y178F
8204	*IGFBP7*	1553	4	57907049	missense	G	T	0.074	p.Q176K
8204	*LRAT*	4744	4	155665827	missense	G	A	0.242	p.A117T
8204	*PDE4D*	1197222	5	58334885	missense	C	A	0.148	p.G17V
8204	*PCDHAC1*	31882	5	140308533	missense	A	G	0.205	p.I686V
8204	*SPINK9*	1040433	5	147718098	missense	A	G	0.154	p.M49V
8204	*SPARC*	3118	5	151051201	missense	T	C	0.073	p.N88S
8204	*KCNK5*	3740	6	39163655	missense	T	G	0.116	p.I99L
8204	*GPR111*	153839	6	47649067	missense	G	T	0.204	p.D190Y
8204	*CTGF*	1901	6	132270628	missense	T	C	0.051	p.M276V
8204	*NHSL1*	20464	6	138745322	missense	C	T	0.191	p.A1573T
8204	*SYNJ2*	3898	6	158484842	missense	C	T	0.087	p.R383W
8204	*PCLO*	33026	7	82451940	missense	C	T	0.057	p.G4888S
8204	*COL1A2*	89	7	94058575	missense	T	C	0.085	p.Y1263H
8204	*COL1A2*	89	7	94058576	missense	A	G	0.085	p.Y1263C
8204	*KIAA1456*	20844	8	12878931	missense	G	A	0.168	p.R248H
8204	*CHMP5*	16410	9	33278185	missense	G	A	0.073	p.A191T
8204	*CEL*	1807	9	135945935	missense	C	G	0.150	p.D461E
8204	*CARS*	139273	11	3050290	missense	G	A	0.054	p.R323W
8204	*OR8K1*	1002907	11	56114355	missense	T	C	0.163	p.S281P
8204	*TRIM29*	12101	11	120008241	missense	C	T	0.133	p.G167S
8204	*DDX23*	4818	12	49231416	missense	C	T	0.074	p.R215Q
8204	*CTDSP2*	5730	12	58217698	missense	G	T	0.052	p.P227T
8204	*CTDSP2*	5730	12	58217770	missense	G	C	0.085	p.L203V
8204	*CTDSP2*	5730	12	58220811	missense	G	T	0.060	p.L108I
8204	*CTDSP2*	5730	12	58220831	missense	C	G	0.074	p.R101T
8204	*CTDSP2*	5730	12	58240188	missense	G	A	0.203	p.R11W
8204	*CTDSP2*	5730	12	58240214	missense	T	G	0.077	p.E2A
8204	*NBEA*	15678	13	36241553	missense	G	T	0.167	p.R2815I
8204	*FGF14*	175929	13	102527628	missense	A	G	0.173	p.I76T
8204	*SLC10A2*	452	13	103718361	missense	G	A	0.169	p.P80L
8204	*MYH6*	2471	14	23870064	missense	A	G	0.203	p.Y422H
8204	*TSSK4*	174944	14	24675211	missense	G	T	0.233	p.G28C
8204	*PLEKHD1*	1161498	14	69992721	missense	C	A	0.171	p.Q269K
8204	*C15orf41*	1290233	15	36872116	missense	C	T	0.057	p.P19S
8204	*LDHAL6B*	33195	15	59500155	missense	G	A	0.131	p.S339N
8204	*GP2*	1502	16	20335392	missense	C	T	0.229	p.R94H
8204	*MYO18A*	203318	17	27493253	missense	G	A	0.106	p.R236W
8204	*CDC27*	1293091	17	45229228	missense	A	C	0.083	p.N338K
8204	*ZBTB7C*	1039360	18	45556098	missense	G	A	0.234	p.R465C
8204	*SAFB*	2967	19	5654161	missense	A	G	0.200	p.Q539R
8204	*MUC16*	24690	19	9048519	missense	C	T	0.130	p.V11038I
8204	*DNMT1*	1379	19	10265118	missense	T	C	0.208	p.T624A
8204	*AP2A1*	130787	19	50308995	missense	C	T	0.061	p.A871V
8204	*DPRX*	1012728	19	54140130	missense	G	A	0.177	p.C155Y
8204	*LILRA2*	6866	19	55086228	missense	T	A	0.156	p.L128Q
8204	*LILRA2*	6866	19	55086245	missense	A	G	0.132	p.T134A
8204	*LILRA2*	6866	19	55086255	missense	G	A	0.128	p.G137E
8204	*LILRA2*	6866	19	55086276	missense	T	G	0.122	p.V144G
8204	*PANX2*	52839	22	50615632	missense	G	A	0.228	p.C164Y
8204	*CHDC2*	173695	X	36162684	missense	C	G	0.114	p.L423V
8204	*FAM104B*	138362	X	55172686	missense	C	T	0.161	p.S60N
8204	*ITGB1BP2*	12278	X	70523493	missense	A	C	0.067	p.E162A
8204	*RGAG4*	1024455	X	71350826	missense	C	A	0.096	p.A189S
8204	*RBMXL3*	1145346	X	114426324	missense	G	A	0.142	p.G774R
8204	*IL13RA1*	1560	X	117880954	missense	C	T	0.082	p.P89L
8206	*EHMT2*	25256	6	31855728	stopgain	G	A	0.082	p.R586X
8206	*TNR*	3285	1	175304931	missense	G	A	0.074	p.R1183W
8206	*IPO9*	18085	1	201822217	missense	C	A	0.059	p.L228M
8206	*PPP2R5A*	6243	1	212530318	missense	A	T	0.077	p.E335D

8206	*PPP2R5A*	6243	1	212530319	missense	A	G	0.061	p.I336V
8206	*OBSCN*	52843	1	228402494	missense	C	G	0.200	p.P508R
8206	*GNPAT*	14236	1	231408078	missense	C	T	0.083	p.R515C
8206	*GPR137B*	3272	1	236341799	missense	A	G	0.058	p.N184D
8206	*TTN*	133378	2	179540696	missense	C	T	0.076	p.E11469K
8206	*CPS1*	1875	2	211455575	missense	T	A	0.080	p.L304I
8206	*MOGAT1*	58165	2	223554125	missense	T	C	0.113	p.Y139H
8206	*GIGYF2*	15575	2	233712247	missense	C	A	0.130	p.P1217Q
8206	*GRM7*	181874	3	6903088	missense	A	G	0.054	p.R5G
8206	*ZNF717*	1290209	3	75788091	missense	T	A	0.211	p.K178M
8206	*ZNF717*	1290209	3	75788230	missense	C	T	0.121	p.V132I
8206	*C3orf30*	152539	3	118865407	missense	A	T	0.115	p.N124I
8206	*SMARCA5*	3601	4	144447566	missense	G	A	0.071	p.V252I
8206	*CDH9*	16279	5	26902759	missense	T	A	0.122	p.H360L
8206	*CDKN2AIPNL*	80656	5	133747430	missense	G	A	0.255	p.A39V
8206	*GPR116*	15234	6	46851907	missense	C	T	0.156	p.V144I
8206	*BACH2*	21813	6	90718350	missense	T	C	0.052	p.N72D
8206	*LTV1*	32860	6	144181623	missense	T	A	0.074	p.S286T
8206	*PDE10A*	1130690	6	165827038	missense	T	A	0.206	p.N410I
8206	*MUC17*	1040105	7	100678071	missense	C	T	0.058	p.P1125L
8206	*CHPF2*	19015	7	150931272	missense	G	A	0.273	p.A51T
8206	*XKR6*	173683	8	10755995	missense	C	T	0.057	p.D465N
8206	*BNIP3L*	4331	8	26265828	missense	G	A	0.058	p.A183T
8206	*MAK16*	32509	8	33346111	missense	G	A	0.051	p.A47T
8206	*RBP3*	2900	10	48389545	missense	C	T	0.052	p.D445N
8206	*ZNF503*	32772	10	77161167	missense	G	A	0.133	p.A4V
8206	*OR51Q1*	1004757	11	5444323	missense	A	C	0.060	p.N298T
8206	*NRXN2*	138732	11	64453141	missense	G	A	0.229	p.R377W
8206	*SF3B2*	6842	11	65819899	missense	T	C	0.156	p.L15P
8206	*ITPR2*	2223	12	26580925	missense	G	A	0.071	p.P2289L
8206	*CTDSP2*	5730	12	58217698	missense	G	T	0.073	p.P227T
8206	*CTDSP2*	5730	12	58217763	missense	C	T	0.185	p.R205H
8206	*CTDSP2*	5730	12	58217770	missense	G	C	0.073	p.L203V
8206	*CTDSP2*	5730	12	58220801	missense	G	T	0.055	p.T111N
8206	*CTDSP2*	5730	12	58220811	missense	G	T	0.074	p.L108I
8206	*CTDSP2*	5730	12	58220831	missense	C	G	0.117	p.R101T
8206	*WDR89*	80666	14	64066395	missense	C	T	0.057	p.C89Y
8206	*WDR89*	80666	14	64066402	missense	A	G	0.057	p.S87P
8206	*VPS39*	15289	15	42454639	missense	C	T	0.061	p.S750N
8206	*LDHAL6B*	33195	15	59500155	missense	G	A	0.079	p.S339N
8206	*UBAP1L*	1163692	15	65394893	missense	C	T	0.065	p.E84K
8206	*CDH11*	1797	16	65032542	missense	A	T	0.088	p.V149D
8206	*CDH11*	1797	16	65032569	missense	T	C	0.064	p.E140G
8206	*CDH11*	1797	16	65032741	missense	A	C	0.092	p.S83A
8206	*AANAT*	1166579	17	74464883	missense	G	C	0.229	p.G64R
8206	*C18orf65*	1272093	18	74208262	missense	C	T	0.175	p.L36F
8206	*ZNF700*	144566	19	12060844	missense	A	G	0.111	p.K669E
8206	*TPD52L2*	199363	20	62514136	missense	G	A	0.077	p.V147M
8206	*PISD*	14338	22	32017089	missense	C	T	0.062	p.R212Q
8206	*BEND2*	153346	X	18195820	missense	G	A	0.078	p.A500V
8206	*FAM104B*	138362	X	55172686	missense	C	T	0.088	p.S60N
8206	*TAF1*	138923	X	70609439	missense	C	T	0.077	p.A901V
8206	*HTATSF1*	14500	X	135593705	missense	G	A	0.154	p.E601K
8206	*LDOC1*	12317	X	140270981	missense	G	A	0.129	p.L76F
8207	*ZNF717*	1290209	3	75787467	stopgain	G	T	0.112	p.S386X
8207	*PLK2*	6622	5	57750471	stopgain	G	T	0.070	p.S652X
8207	*TNNI3K*	15978	1	74737334	missense	A	C	0.211	p.Q243P
8207	*IFI44L*	6820	1	79093748	missense	A	C	0.129	p.T50P
8207	*LYSMD1*	1136543	1	151138225	missense	T	C	0.247	p.K12E
8207	*RC3H1*	172071	1	173907924	missense	G	A	0.066	p.P1113S
8207	*RASAL2*	170692	1	178427372	missense	G	T	0.185	p.S982I
8207	*NLRP3*	183395	1	247587452	missense	C	T	0.117	p.A236V
8207	*ODC1*	2539	2	10581765	missense	C	T	0.057	p.D371N
8207	*AFF3*	2285	2	100170827	missense	G	C	0.310	p.L1169V
8207	*HOXD11*	21192	2	176972268	missense	C	T	0.268	p.A62V
8207	*UNC80*	182587	2	210843307	missense	G	A	0.170	p.R2927H
8207	*OBSL1*	15311	2	220416504	missense	C	G	0.265	p.M1810I
8207	*ZNF717*	1290209	3	75788230	missense	C	T	0.135	p.V132I
8207	*NAALADL2*	207015	3	175520828	missense	G	A	0.233	p.R742Q
8207	*LIMCH1*	14988	4	41648659	missense	A	G	0.155	p.T472A
8207	*AFP*	1134	4	74306391	missense	A	G	0.152	p.S115G
8207	*DSPP*	14208	4	88537574	missense	G	A	0.088	p.D1254N
8207	*CYP2U1*	183075	4	108866213	missense	T	C	0.136	p.L193P
8207	*MYO10*	12334	5	16701752	missense	T	C	0.146	p.K918E
8207	*GPR98*	32119	5	89990255	missense	G	C	0.202	p.G2561A
8207	*CCDC112*	152549	5	114611076	missense	T	C	0.307	p.D169G
8207	*FAM8A1*	16255	6	17601035	missense	A	G	0.061	p.H132R
8207	*FAM8A1*	16255	6	17601040	missense	G	A	0.054	p.G134S
8207	*FAM8A1*	16255	6	17601041	missense	G	T	0.051	p.G134V
8207	*FAM8A1*	16255	6	17601044	missense	T	C	0.052	p.L135P
8207	*FAM8A1*	16255	6	17601058	missense	G	A	0.055	p.A140T

8207	*FAM8A1*	16255	6	17601086	missense	A	G	0.055	p.Q149R
8207	*MUC22*	1198815	6	30996168	missense	C	T	0.136	p.A987V
8207	*CCND3*	1760	6	41903706	missense	G	T	0.134	p.P284H
8207	*COL12A1*	80645	6	75801047	missense	A	G	0.100	p.L2915S
8207	*ADGB*	24694	6	146977930	missense	A	C	0.106	p.E142D
8207	*ARID1B*	20732	6	157100043	missense	G	C	0.184	p.G327A
8207	*RELN*	173054	7	103276809	missense	A	C	0.086	p.F726V
8207	*RELN*	173054	7	103338450	missense	T	G	0.061	p.E331D
8207	*DOCK4*	14705	7	111386456	missense	T	G	0.059	p.E1528D
8207	*PURG*	1015508	8	30854119	missense	A	G	0.182	p.V320A
8207	*CNTLN*	17738	9	17273833	missense	A	T	0.238	p.I318F
8207	*OR13D1*	1004484	9	107456802	missense	G	C	0.244	p.E34Q
8207	*LMX1B*	2316	9	129458722	missense	G	A	0.185	p.A394T
8207	*MCM10*	182751	10	13213012	missense	C	T	0.055	p.T33M
8207	*LIPJ*	1010939	10	90351174	missense	T	C	0.175	p.Y17H
8207	*ROBO3*	22370	11	124748620	missense	C	A	0.054	p.A1154D
8207	*CTDSP2*	5730	12	58217698	missense	G	T	0.122	p.P227T
8207	*CTDSP2*	5730	12	58217770	missense	G	C	0.148	p.L203V
8207	*CTDSP2*	5730	12	58220811	missense	G	T	0.094	p.L108I
8207	*CTDSP2*	5730	12	58220831	missense	C	G	0.108	p.R101T
8207	*TBX3*	16569	12	115115388	missense	T	G	0.156	p.K313T
8207	*DACH1*	80759	13	72147103	missense	G	A	0.194	p.P392S
8207	*FGF14*	175929	13	102375189	missense	T	C	0.187	p.T251A
8207	*ADCK1*	20421	14	78285388	missense	T	C	0.058	p.F23L
8207	*HERC2*	4667	15	28501295	missense	C	T	0.286	p.V896M
8207	*TNFRSF12A*	16639	16	3071797	missense	G	C	0.261	p.E120D
8207	*NF1*	1128147	17	29509580	missense	G	A	0.060	p.R262H
8207	*PSMB3*	2795	17	36912204	missense	C	T	0.138	p.T86I
8207	*FBXL12*	17703	19	9922306	missense	C	T	0.176	p.G83S
8207	*TPRX1*	198479	19	48305528	missense	T	A	0.216	p.N247I
8207	*TPRX1*	198479	19	48305531	missense	G	A	0.195	p.P246L
8207	*ZSCAN5B*	1080456	19	56704307	missense	T	C	0.056	p.N39D
8207	*CHGB*	1819	20	5904133	missense	T	C	0.122	p.V448A
8207	*TPX2*	12112	20	30386234	missense	G	A	0.188	p.R671Q
8207	*MC3R*	19888	20	54824087	missense	T	C	0.266	p.V63A
8207	*EIF4ENIF1*	19843	22	31846304	missense	C	T	0.224	p.G519S
8212	*AASS*	5763	7	121756978	stopgain	C	A	0.222	p.E241X
8212	*FASTK*	33015	7	150776013	stopgain	G	A	0.147	p.R201X
8212	*WDR89*	80666	14	64066363	stopgain	G	A	0.078	p.R100X
8212	*KCTD1*	198991	18	24056619	stopgain	C	A	0.061	p.G57X
8212	*SLC5A7*	21815	2	108609473	missense	C	G	0.105	p.T113S
8212	*ITPR1*	2222	3	4702710	missense	C	G	0.088	p.T397S
8212	*GADL1*	207359	3	30880550	missense	A	G	0.073	p.F281S
8212	*ZNF717*	1290209	3	75788230	missense	C	T	0.117	p.V132I
8212	*COL6A5*	153264	3	130110487	missense	T	G	0.075	p.L961R
8212	*CCNL1*	20307	3	156867308	missense	C	G	0.125	p.D367H
8212	*MASP1*	1879	3	186938043	missense	T	A	0.146	p.K639M
8212	*ARHGAP24*	31305	4	86916156	missense	A	T	0.135	p.Q450L
8212	*MUC22*	1198815	6	30996831	missense	T	C	0.094	p.V1208A
8212	*MUC22*	1198815	6	30996836	missense	G	A	0.097	p.A1210T
8212	*MUC22*	1198815	6	30996837	missense	C	T	0.097	p.A1210V
8212	*MUC22*	1198815	6	30996839	missense	G	A	0.103	p.E1211K
8212	*DST*	1723	6	56480682	missense	C	T	0.054	p.G2528D
8212	*ZNF804B*	181646	7	88964702	missense	T	G	0.164	p.C802W
8212	*DNAJB9*	12328	7	108213520	missense	G	A	0.143	p.G132E
8212	*MGAM*	4668	7	141730463	missense	C	G	0.076	p.S459C
8212	*KBTBD11*	14867	8	1950194	missense	C	A	0.222	p.A279E
8212	*STMN4*	30795	8	27098753	missense	C	A	0.098	p.V73F
8212	*NRG1*	13964	8	32621466	missense	A	C	0.136	p.K495T
8212	*ZFHX4*	24721	8	77616655	missense	A	C	0.079	p.D111A
8212	*PKHD1L1*	177531	8	110497348	missense	C	A	0.130	p.L3218M
8212	*ADRB1*	684	10	115804898	missense	G	T	0.087	p.C336F
8212	*ENO4*	1242699	10	118616118	missense	G	T	0.085	p.W137L
8212	*CTBP2*	22802	10	126692019	missense	G	A	0.128	p.P34L
8212	*GRM5*	1143831	11	88242028	missense	G	A	0.085	p.T1124M
8212	*FAIM2*	12306	12	50283305	missense	G	T	0.118	p.L204I
8212	*KRT3*	57088	12	53189427	missense	C	G	0.099	p.A134P
8212	*CTDSP2*	5730	12	58217698	missense	G	T	0.092	p.P227T
8212	*CTDSP2*	5730	12	58220801	missense	G	T	0.054	p.T111N
8212	*CTDSP2*	5730	12	58220811	missense	G	T	0.105	p.L108I
8212	*CTDSP2*	5730	12	58220831	missense	C	G	0.083	p.R101T
8212	*GOLGA3*	5895	12	133350780	missense	G	A	0.073	p.P1424S
8212	*AKAP6*	4274	14	33292392	missense	G	T	0.108	p.L1791F
8212	*WDR89*	80666	14	64066352	missense	T	A	0.069	p.R103S
8212	*WDR89*	80666	14	64066367	missense	A	T	0.094	p.D98E
8212	*WDR89*	80666	14	64066395	missense	C	T	0.135	p.C89Y
8212	*WDR89*	80666	14	64066398	missense	G	C	0.133	p.A88G
8212	*WDR89*	80666	14	64066402	missense	A	G	0.133	p.S87P
8212	*SERPINA11*	1080451	14	94912938	missense	T	C	0.085	p.K216R
8212	*UMOD*	3361	16	20359982	missense	G	A	0.063	p.A247V
8212	*ALOXE3*	21628	17	8015509	missense	A	G	0.092	p.L229S

8212	*DNAH9*	1372	17	11671879	missense	A	C	0.146	p.K2427T
8212	*TIMM44*	6351	19	7992149	missense	C	T	0.139	p.D428N
8212	*ZNF414*	32370	19	8576597	missense	C	T	0.161	p.A260T
8212	*ZNF805*	1145078	19	57764976	missense	G	C	0.115	p.E263D
8213	*WDR89*	80666	14	64066363	stopgain	G	A	0.058	p.R100X
8213	*C17orf75*	22344	17	30660465	stopgain	G	A	0.083	p.R316X
8213	*ATP8B3*	138813	19	1789001	stopgain	C	T	0.167	p.W988X
8213	*CASQ1*	1231	1	160160548	missense	G	A	0.272	p.A3T
8213	*LRP1B*	18557	2	141092049	missense	C	A	0.319	p.V4066L
8213	*ERBB4*	5235	2	212570054	missense	C	A	0.213	p.R396I
8213	*DOCK10*	14689	2	225717680	missense	T	C	0.291	p.K683R
8213	*2-Sep*	1282973	2	242255956	missense	G	T	0.269	p.G25V
8213	*MAGI1*	15520	3	65376930	missense	G	T	0.173	p.P768H
8213	*C1QTNF7*	31911	4	15437392	missense	A	C	0.264	p.S9R
8213	*WDFY3*	14991	4	85658399	missense	T	C	0.152	p.D2232G
8213	*WDFY3*	14991	4	85708717	missense	T	G	0.244	p.E1273D
8213	*DSPP*	14208	4	88537243	missense	C	A	0.203	p.D1143E
8213	*F11*	128	4	187206946	missense	G	A	0.244	p.E487K
8213	*ITGA1*	181501	5	52218646	missense	G	A	0.281	p.D778N
8213	*ERAP1*	16442	5	96126318	missense	T	C	0.286	p.E450G
8213	*PCDHGA1*	31993	5	140710805	missense	G	C	0.264	p.G185A
8213	*PCDHGC5*	32407	5	140871416	missense	G	A	0.276	p.R870H
8213	*JAKMIP2*	14790	5	147008336	missense	G	A	0.185	p.T658I
8213	*TIGD6*	30953	5	149375542	missense	G	T	0.305	p.L124M
8213	*MUC22*	1198815	6	30996831	missense	T	C	0.152	p.V1208A
8213	*MUC22*	1198815	6	30996836	missense	G	A	0.151	p.A1210T
8213	*MUC22*	1198815	6	30996837	missense	C	T	0.149	p.A1210V
8213	*MUC22*	1198815	6	30996839	missense	G	A	0.165	p.E1211K
8213	*HLA-DQA2*	20056	6	32713076	missense	A	C	0.070	p.S75R
8213	*HLA-DQA2*	20056	6	32713080	missense	A	G	0.072	p.K76R
8213	*HLA-DQA2*	20056	6	32713090	missense	T	A	0.059	p.S79R
8213	*PIK3CG*	2649	7	106509642	missense	G	A	0.056	p.E546K
8213	*POT1*	15450	7	124511069	missense	C	A	0.256	p.D51Y
8213	*CNBD1*	173538	8	87917344	missense	C	T	0.222	p.T65I
8213	*C9orf72*	18325	9	27548356	missense	C	T	0.231	p.E442K
8213	*RASEF*	152573	9	85677517	missense	G	C	0.243	p.A89G
8213	*PAPPA*	2581	9	118997474	missense	T	C	0.211	p.S764P
8213	*BRD3*	7371	9	136918434	missense	C	A	0.233	p.A56S
8213	*SLC16A9*	194298	10	61413610	missense	C	A	0.146	p.A392S
8213	*PPAPDC1A*	1030059	10	122348953	missense	G	A	0.292	p.R252K
8213	*SIRT3*	12239	11	233376	missense	C	T	0.057	p.G147D
8213	*GRAMD1B*	20716	11	123480962	missense	C	G	0.212	p.T476S
8213	*ABCC9*	20297	12	22025644	missense	C	A	0.073	p.G705C
8213	*CCDC91*	18318	12	28603103	missense	C	A	0.056	p.L258I
8213	*CTDSP2*	5730	12	58217763	missense	C	T	0.244	p.R205H
8213	*CTDSP2*	5730	12	58217770	missense	G	C	0.076	p.L203V
8213	*CTDSP2*	5730	12	58220819	missense	A	G	0.078	p.V105A
8213	*CTDSP2*	5730	12	58220831	missense	C	A	0.087	p.R101M
8213	*GZMB*	4131	14	25102248	missense	C	T	0.236	p.E26K
8213	*WDR89*	80666	14	64066352	missense	T	A	0.052	p.R103S
8213	*ADAM20*	3814	14	70990018	missense	G	A	0.324	p.P536L
8213	*IFI27*	5532	14	94582140	missense	C	T	0.258	p.H4Y
8213	*CLMN*	24734	14	95670689	missense	C	T	0.239	p.E333K
8213	*AHNAK2*	138420	14	105406702	missense	G	A	0.250	p.P5029L
8213	*MYO5A*	1142495	15	52667653	missense	C	T	0.257	p.A809T
8213	*FAM154B*	1008226	15	82564095	missense	G	C	0.286	p.E69Q
8213	*BNC1*	1717	15	83933269	missense	G	C	0.257	p.P245R
8213	*MESP2*	1039958	15	90320144	missense	C	G	0.133	p.Q186E
8213	*CEMP1*	1048212	16	2580867	missense	G	A	0.277	p.H70Y
8213	*C16orf72*	14117	16	9186055	missense	C	T	0.194	p.P52S
8213	*PPP4C*	2720	16	30094070	missense	G	A	0.250	p.G69S
8213	*NFATC3*	173165	16	68200849	missense	C	T	0.226	p.R569C
8213	*ARHGAP27*	199282	17	43473870	missense	C	T	0.107	p.R378H
8213	*ABCA8*	7168	17	66937024	missense	A	G	0.231	p.L59P
8213	*CERS4*	24552	19	8322764	missense	C	A	0.299	p.A248D
8213	*DDX3X*	1193417	X	41205655	missense	G	A	0.500	p.A481T
8215	*KISS1*	2256	1	204159612	stoploss	T	C	0.118	p.X139W
8215	*VWF*	552	12	6058310	stopgain	G	T	0.237	p.C2771X
8215	*WDR89*	80666	14	64066363	stopgain	G	A	0.102	p.R100X
8215	*ASB17*	80868	1	76397823	missense	C	T	0.060	p.A52T
8215	*WDR63*	145172	1	85547057	missense	G	T	0.083	p.A82S
8215	*NT5C1B*	33253	2	18767530	missense	G	T	0.185	p.T143N
8215	*AAK1*	14911	2	69708048	missense	G	A	0.299	p.P838L
8215	*TANC1*	33394	2	160087420	missense	A	G	0.302	p.Q1828R
8215	*TTN*	133379	2	179614353	missense	T	A	0.148	p.Q4258H
8215	*MYO1B*	12223	2	192256834	missense	G	T	0.200	p.R661S
8215	*ERBB4*	5235	2	212989569	missense	C	T	0.132	p.A48T
8215	*MOGAT1*	58165	2	223554125	missense	T	C	0.182	p.Y139H
8215	*ZNF717*	1290209	3	75788230	missense	C	T	0.104	p.V132I
8215	*1-Mar*	1166373	4	164775193	missense	C	T	0.157	p.A31T
8215	*HLA-DQA2*	20056	6	32713076	missense	A	C	0.090	p.S75R

8215	*HLA-DQA2*	20056	6	32713090	missense	T	A	0.079	p.S79R
8215	*EYS*	198283	6	66044928	missense	G	T	0.159	p.Q571K
8215	*PLEKHG1*	1029884	6	151121977	missense	G	A	0.080	p.R251Q
8215	*PLEKHA8*	32639	7	30101601	missense	A	G	0.211	p.Q396R
8215	*PCLO*	33026	7	82785041	missense	T	G	0.126	p.I306L
8215	*FZD3*	145866	8	28385280	missense	G	T	0.126	p.A335S
8215	*KAT6A*	6766	8	41805346	missense	C	T	0.100	p.D609N
8215	*EBAG9*	198120	8	110563084	missense	T	G	0.056	p.F11V
8215	*COL13A1*	80805	10	71662606	missense	C	A	0.258	p.P295T
8215	*GLRX3*	6541	10	131943522	missense	C	T	0.087	p.A47V
8215	*OR52N4*	1005175	11	5776643	missense	C	T	0.217	p.R225W
8215	*SOX6*	33326	11	16117552	missense	T	C	0.067	p.K364R
8215	*SOX6*	33326	11	16117585	missense	A	G	0.151	p.F353S
8215	*P2RX3*	2559	11	57117368	missense	G	A	0.238	p.R234H
8215	*KDM2A*	1256405	11	67007826	missense	G	C	0.216	p.D50H
8215	*CAPN5*	4055	11	76796018	missense	A	C	0.056	p.D29A
8215	*CNTN5*	175566	11	100211334	missense	G	T	0.125	p.G957V
8215	*TECTA*	5422	11	120996567	missense	C	A	0.262	p.T587N
8215	*ATN1*	1940	12	7045894	missense	G	C	0.075	p.Q488H
8215	*KRT3*	57088	12	53189427	missense	C	G	0.111	p.A134P
8215	*SP1*	138473	12	53776733	missense	C	G	0.175	p.S334R
8215	*CTDSP2*	5730	12	58217698	missense	G	T	0.082	p.P227T
8215	*CTDSP2*	5730	12	58217763	missense	C	T	0.229	p.R205H
8215	*CTDSP2*	5730	12	58220811	missense	G	T	0.074	p.L108I
8215	*CTDSP2*	5730	12	58220819	missense	A	G	0.118	p.V105A
8215	*LRRIQ1*	1079910	12	85450256	missense	A	G	0.182	p.E562G
8215	*NR1H4*	1206993	12	100897169	missense	G	A	0.101	p.V2I
8215	*HSP90B1*	3299	12	104341133	missense	C	G	0.122	p.D769E
8215	*RIMBP2*	15347	12	130926679	missense	G	T	0.226	p.D389E
8215	*OR4K1*	1004063	14	20404535	missense	T	C	0.206	p.L237P
8215	*RHOJ*	20663	14	63749868	missense	G	T	0.172	p.L144F
8215	*WDR89*	80666	14	64066352	missense	T	A	0.100	p.R103S
8215	*WDR89*	80666	14	64066367	missense	A	T	0.112	p.D98E
8215	*WDR89*	80666	14	64066395	missense	C	T	0.101	p.C89Y
8215	*WDR89*	80666	14	64066398	missense	G	C	0.107	p.A88G
8215	*WDR89*	80666	14	64066402	missense	A	G	0.111	p.S87P
8215	*ASB2*	16150	14	94405980	missense	C	T	0.070	p.R364H
8215	*SLC25A29*	152333	14	100758733	missense	C	T	0.200	p.A201T
8215	*HSP90AA1*	5348	14	102551278	missense	T	C	0.241	p.K363E
8215	*SLC28A2*	4212	15	45561672	missense	A	G	0.233	p.Y502C
8215	*VPS13C*	20821	15	62202489	missense	G	A	0.078	p.P2911S
8215	*BAIAP3*	3933	16	1388651	missense	C	T	0.176	p.A69V
8215	*CRAMP1L*	20825	16	1718005	missense	C	G	0.224	p.L1049V
8215	*SNX29*	32167	16	12571597	missense	G	A	0.227	p.D687N
8215	*CDH11*	1797	16	65025686	missense	G	C	0.086	p.P266A
8215	*CDH11*	1797	16	65025724	missense	T	C	0.091	p.K253R
8215	*WDR81*	152348	17	1639381	missense	C	G	0.204	p.L741V
8215	*RAI1*	30665	17	17697096	missense	G	C	0.109	p.Q278H
8215	*NF1*	1128147	17	29483089	missense	A	G	0.140	p.K50R
8215	*SEC14L1*	3003	17	75196689	missense	G	A	0.167	p.E315K
8215	*MYOM1*	19856	18	3086135	missense	C	A	0.225	p.K1384N
8215	*TTC28*	1145418	22	28394914	missense	G	A	0.218	p.T1578M
8215	*DDX3X*	1193417	X	41206189	stopgain	C	T	0.633	p.Q549X
8216	*CCDC171*	173550	9	15784629	stopgain	T	G	0.341	p.Y1068X
8216	*DNAH3*	17539	16	20975551	stopgain	C	A	0.246	p.E3219X
8216	*PTPRU*	133178	1	29618493	missense	C	T	0.265	p.R821W
8216	*PPP2R5A*	6243	1	212502608	missense	G	A	0.073	p.V105I
8216	*PGBD5*	1258311	1	230493029	missense	C	T	0.301	p.D124N
8216	*DCDC2C*	1287444	2	3789631	missense	G	A	0.217	p.V180I
8216	*SAP130*	24545	2	128747352	missense	C	A	0.143	p.M548I
8216	*SCN9A*	2977	2	167162416	missense	C	A	0.207	p.G161V
8216	*FSIP2*	173651	2	186655481	missense	C	A	0.063	p.D1295E
8216	*ZNF717*	1290209	3	75788230	missense	C	T	0.089	p.V132I
8216	*PARP9*	1146106	3	122254966	missense	C	T	0.266	p.S710N
8216	*ZNF518B*	53042	4	10446244	missense	C	T	0.256	p.R570K
8216	*CCSER1*	207491	4	91230298	missense	G	T	0.115	p.G288V
8216	*NPNT*	1184693	4	106861733	missense	C	T	0.315	p.R265C
8216	*ANKRD50*	20337	4	125590810	missense	T	C	0.328	p.S1208G
8216	*MTMR12*	1294344	5	32248930	missense	G	A	0.091	p.P282S
8216	*PCDH1*	32420	5	141248622	missense	A	G	0.280	p.S139P
8216	*DDC*	1242890	7	50560650	missense	G	A	0.250	p.A333V
8216	*SSC4D*	80744	7	76033683	missense	C	T	0.255	p.G25E
8216	*CSMD1*	33225	8	2876015	missense	G	T	0.300	p.S2671R
8216	*OR13D1*	1004484	9	107457091	missense	C	T	0.275	p.A130V
8216	*NPY4R*	5972	10	47087874	missense	G	A	0.101	p.G364E
8216	*CTBP2*	22802	10	126686680	missense	A	C	0.182	p.C140G
8216	*KRT3*	57088	12	53189427	missense	C	G	0.222	p.A134P
8216	*CTDSP2*	5730	12	58217698	missense	G	T	0.114	p.P227T
8216	*CTDSP2*	5730	12	58217763	missense	C	T	0.259	p.R205H
8216	*CTDSP2*	5730	12	58217770	missense	G	C	0.110	p.L203V
8216	*CTDSP2*	5730	12	58220801	missense	G	T	0.118	p.T111N

8216	*HSP90B1*	3299	12	104341190	missense	A	T	0.050	p.E788D
8216	*ACAD10*	25247	12	112186176	missense	T	G	0.063	p.H878Q
8216	*ACAD10*	25247	12	112186201	missense	G	A	0.063	p.V887M
8216	*PIWIL1*	4764	12	130841552	missense	G	T	0.207	p.L498F
8216	*LTBP2*	428	14	75017904	missense	C	A	0.294	p.A517S
8216	*ATF7IP2*	24997	16	10575819	missense	A	G	0.093	p.R588G
8216	*CDH11*	1797	16	65032741	missense	A	C	0.069	p.S83A
8216	*TTC39C*	153211	18	21660861	missense	T	C	0.062	p.M197T
8216	*SETBP1*	15559	18	42532808	missense	A	G	0.301	p.D1168G
8216	*GP6*	1083899	19	55526103	missense	G	A	0.076	p.P404S
8218	*CCDC30*	1080850	1	43108234	stopgain	C	T	0.143	p.Q577X
8218	*WDR89*	80666	14	64066363	stopgain	G	A	0.088	p.R100X
8218	*BAI2*	1294336	1	32207068	missense	C	T	0.131	p.R567H
8218	*CELSR2*	1408	1	109811330	missense	C	T	0.180	p.A2149V
8218	*KIRREL*	18240	1	158045975	missense	G	C	0.195	p.C42S
8218	*COLGALT2*	15101	1	183942836	missense	G	T	0.078	p.L181M
8218	*PLXNA2*	25179	1	208206803	missense	G	A	0.081	p.P1639L
8218	*PLD5*	152666	1	242687422	missense	C	T	0.157	p.V53I
8218	*ATAD2B*	17552	2	23977054	missense	C	G	0.176	p.V1444L
8218	*MAPRE3*	12326	2	27246239	missense	G	A	0.056	p.G54D
8218	*PRR30*	178553	2	27360132	missense	G	T	0.199	p.Q356K
8218	*MEIS1*	2398	2	66670065	missense	G	C	0.186	p.R172P
8218	*DPP10*	20868	2	116599812	missense	T	C	0.130	p.V711A
8218	*FSIP2*	173651	2	186668723	missense	G	T	0.212	p.S4986I
8218	*STK36*	15690	2	219544724	missense	C	A	0.143	p.L353I
8218	*MOGAT1*	58165	2	223554125	missense	T	C	0.121	p.Y139H
8218	*FLNB*	1457	3	58134543	missense	G	A	0.063	p.E2050K
8218	*ZNF717*	1290209	3	75788230	missense	C	T	0.072	p.V132I
8218	*MYLK*	53028	3	123376048	missense	G	A	0.131	p.R1405C
8218	*PRSS12*	3619	4	119259392	missense	C	G	0.120	p.V194L
8218	*CAMK4*	1744	5	110819845	missense	A	T	0.167	p.D368V
8218	*PCDHA3*	31497	5	140181269	missense	G	A	0.108	p.G163R
8218	*CPEB4*	30627	5	173316986	missense	C	A	0.068	p.Q84K
8218	*HNRNPAB*	31266	5	177637589	missense	C	T	0.134	p.R322C
8218	*GFPT2*	5110	5	179729433	missense	C	T	0.068	p.R665Q
8218	*FAM8A1*	16255	6	17601035	missense	A	G	0.063	p.H132R
8218	*FAM8A1*	16255	6	17601040	missense	G	A	0.056	p.G134S
8218	*FAM8A1*	16255	6	17601041	missense	G	T	0.056	p.G134V
8218	*FAM8A1*	16255	6	17601044	missense	T	C	0.058	p.L135P
8218	*FAM8A1*	16255	6	17601058	missense	G	A	0.060	p.A140T
8218	*FAM8A1*	16255	6	17601086	missense	A	G	0.066	p.Q149R
8218	*HLA-DRB1*	2124	6	32551942	missense	T	C	0.172	p.D105G
8218	*SLC22A7*	153320	6	43266424	missense	A	G	0.097	p.T110A
8218	*AK9*	1145128	6	109940409	missense	G	A	0.098	p.R429C
8218	*NPM2*	182795	8	21882966	missense	G	C	0.211	p.E26D
8218	*BAI1*	1702	8	143623718	missense	A	C	0.053	p.T1375P
8218	*APBA1*	1163	9	72131099	missense	C	T	0.125	p.R343H
8218	*NTRK2*	6180	9	87475984	missense	A	C	0.192	p.K476Q
8218	*ABCA1*	5502	9	107583695	missense	T	C	0.122	p.N974S
8218	*SKIDA1*	207371	10	21804506	missense	T	G	0.128	p.N749T
8218	*SORCS1*	52918	10	108437074	missense	A	T	0.260	p.L610H
8218	*TACC2*	206862	10	123845410	missense	C	A	0.156	p.T1132N
8218	*ADAMTS15*	139055	11	130341273	missense	G	T	0.094	p.K691N
8218	*KLRC1*	213658	12	10603136	missense	C	A	0.143	p.G77V
8218	*DUSP16*	30640	12	12630494	missense	T	G	0.162	p.E424A
8218	*KRT3*	57088	12	53189427	missense	C	G	0.088	p.A134P
8218	*PDE1B*	1288769	12	54967153	missense	T	C	0.170	p.I147T
8218	*CTDSP2*	5730	12	58217763	missense	C	T	0.136	p.R205H
8218	*CTDSP2*	5730	12	58217770	missense	G	C	0.079	p.L203V
8218	*CTDSP2*	5730	12	58220801	missense	G	T	0.078	p.T111N
8218	*CTDSP2*	5730	12	58220811	missense	G	T	0.053	p.L108I
8218	*CTDSP2*	5730	12	58240188	missense	G	A	0.111	p.R11W
8218	*PTPRQ*	1145026	12	80943510	missense	A	G	0.121	p.T1256A
8218	*MPHOSPH8*	17520	13	20221166	missense	T	G	0.192	p.M318R
8218	*N4BP2L2*	33111	13	33016577	missense	T	G	0.131	p.Q699H
8218	*WDR89*	80666	14	64066352	missense	T	A	0.090	p.R103S
8218	*WDR89*	80666	14	64066367	missense	A	T	0.085	p.D98E
8218	*WDR89*	80666	14	64066395	missense	C	T	0.087	p.C89Y
8218	*WDR89*	80666	14	64066398	missense	G	C	0.087	p.A88G
8218	*WDR89*	80666	14	64066402	missense	A	G	0.085	p.S87P
8218	*YLPM1*	19589	14	75264567	missense	C	T	0.128	p.A856V
8218	*ATXN3*	30660	14	92537397	missense	T	C	0.122	p.T130A
8218	*DLK1*	3836	14	101195368	missense	G	C	0.143	p.C76S
8218	*RTL1*	1134888	14	101348485	missense	C	T	0.169	p.V881I
8218	*TRPM1*	2420	15	31362323	missense	A	T	0.167	p.W64R
8218	*MYH11*	22844	16	15835617	missense	C	G	0.169	p.Q891H
8218	*UMOD*	3361	16	20355416	missense	T	G	0.242	p.I454L
8218	*ZFHX3*	6885	16	72828452	missense	T	C	0.204	p.H2710R
8218	*WDR81*	152348	17	1637462	missense	C	T	0.114	p.H660Y
8218	*KRT9*	226	17	39723842	missense	T	C	0.099	p.S519G
8218	*CDC27*	1293091	17	45229234	missense	A	C	0.190	p.S336R

8218	*SCN4A*	334	17	62020395	missense	A	G	0.170	p.M1360T
8218	*PTPRM*	2845	18	7906519	missense	T	C	0.202	p.I162T
8218	*DSC2*	24422	18	28649110	missense	G	C	0.128	p.A753G
8218	*ST8SIA3*	15879	18	55027376	missense	T	A	0.099	p.H337Q
8218	*JSRP1*	144616	19	2252561	missense	T	C	0.218	p.R255G
8218	*PPFIA3*	3660	19	49639990	missense	C	T	0.138	p.R559W
8218	*CDH22*	21248	20	44828185	missense	G	A	0.184	p.R434C
8218	*KIAA1671*	1145206	22	25437455	missense	C	T	0.098	p.P1453L
8218	*GYG2*	3918	X	2761334	missense	A	G	0.211	p.R61G
8218	*CDKL5*	3159	X	18627604	missense	C	T	0.194	p.P689L
8218	*CACNA1F*	5183	X	49087000	missense	C	T	0.151	p.G198E
8218	*FAM104B*	1166703	X	55172521	missense	G	T	0.087	p.T114K
8218	*FAM104B*	138362	X	55172686	missense	C	T	0.140	p.S60N
8218	*FAM104B*	138362	X	55172689	missense	G	T	0.098	p.A59E
8218	*RPS6KA6*	14496	X	83319320	missense	G	A	0.143	p.R735W
8218	*BEX5*	1159560	X	101409105	missense	C	A	0.167	p.G45C
8218	*SLITRK4*	173078	X	142718677	missense	G	A	0.171	p.S83L
8222	*PLCH1*	14996	3	155200106	stopgain	C	A	0.178	p.G1245X
8222	*GNAT3*	1102386	7	80141196	stopgain	G	T	0.066	p.S16X
8222	*PCLO*	33026	7	82784338	stopgain	G	T	0.165	p.S540X
8222	*NCOR1*	6311	17	15984008	stopgain	G	A	0.108	p.Q1071X
8222	*TJP3*	1267561	19	3728698	stopgain	G	T	0.152	p.E58X
8222	*UBXN11*	183008	1	26608849	missense	T	C	0.266	p.S502G
8222	*ARID1A*	139135	1	27087363	missense	T	C	0.056	p.I646T
8222	*LCE2D*	178430	1	152636820	missense	T	C	0.212	p.L80P
8222	*ADAMTS9*	182920	3	64587644	missense	C	G	0.218	p.Q1331H
8222	*ZNF717*	1290209	3	75787351	missense	C	A	0.082	p.G425W
8222	*CBLB*	170662	3	105586351	missense	C	T	0.170	p.G24D
8222	*KIAA0226*	14687	3	197401980	missense	G	C	0.054	p.A898G
8222	*PI4K2B*	18323	4	25265395	missense	A	T	0.143	p.E334D
8222	*FREM3*	1168235	4	144620631	missense	G	A	0.190	p.R400C
8222	*NADK2*	1287341	5	36208759	missense	A	T	0.051	p.N135K
8222	*MAP3K1*	5921	5	56177851	missense	A	T	0.109	p.T942S
8222	*SH3TC2*	24577	5	148389854	missense	A	T	0.130	p.H1102Q
8222	*FAT2*	1447	5	150900870	missense	G	A	0.145	p.R3762W
8222	*KCNQ5*	19842	6	73904473	missense	A	G	0.185	p.Q731R
8222	*DMRT3*	21240	9	990110	missense	C	G	0.233	p.T175S
8222	*SLC24A2*	20344	9	19785978	missense	C	T	0.149	p.R296H
8222	*FBP2*	3837	9	97329590	missense	C	T	0.068	p.A223T
8222	*SVIL*	21738	10	29822202	missense	C	A	0.119	p.R365L
8222	*ANK3*	20987	10	61828941	missense	C	A	0.154	p.A3900S
8222	*CTBP2*	22802	10	126686649	missense	G	C	0.093	p.T150R
8222	*CTBP2*	22802	10	126686656	missense	T	C	0.086	p.R148G
8222	*CTBP2*	22802	10	126686659	missense	G	A	0.083	p.R147W
8222	*OR52E4*	1005165	11	5905718	missense	G	C	0.180	p.A66P
8222	*TRPT1*	31472	11	63991428	missense	A	G	0.168	p.S228P
8222	*DCP1B*	152640	12	2062353	missense	G	C	0.052	p.H251Q
8222	*OVCH1*	183378	12	29631779	missense	C	A	0.058	p.G353V
8222	*CTDSP2*	5730	12	58217698	missense	G	T	0.077	p.P227T
8222	*CTDSP2*	5730	12	58217763	missense	C	T	0.228	p.R205H
8222	*CTDSP2*	5730	12	58217770	missense	G	C	0.076	p.L203V
8222	*CTDSP2*	5730	12	58220801	missense	G	T	0.110	p.T111N
8222	*CTDSP2*	5730	12	58220811	missense	G	T	0.086	p.L108I
8222	*CTDSP2*	5730	12	58220831	missense	C	A	0.078	p.R101M
8222	*CTDSP2*	5730	12	58240188	missense	G	A	0.072	p.R11W
8222	*PABPC3*	30979	13	25670691	missense	G	T	0.055	p.V119F
8222	*PABPC3*	30979	13	25670703	missense	G	T	0.053	p.G123C
8222	*PABPC3*	30979	13	25670712	missense	C	G	0.050	p.L126V
8222	*FAM155A*	1080396	13	108518601	missense	G	A	0.118	p.P115L
8222	*FAM174B*	207446	15	93198688	missense	A	C	0.105	p.S68A
8222	*APOBR*	18690	16	28507445	missense	G	C	0.157	p.E361D
8222	*APOBR*	18690	16	28507452	missense	G	T	0.119	p.G364W
8222	*APOBR*	18690	16	28507458	missense	G	A	0.057	p.A366T
8222	*BCAR1*	14567	16	75269671	missense	G	A	0.146	p.P228S
8222	*FBXO39*	153230	17	6683372	missense	C	G	0.121	p.T62S
8222	*ANKLE1*	1278444	19	17397499	missense	G	T	0.178	p.C644F
8222	*SUN5*	80675	20	31572942	missense	A	G	0.166	p.F316S
8222	*DDX3X*	1193417	X	41204575	stopgain	C	T	0.253	p.Q374X
8223	*SP4*	3112	7	21469033	stopgain	C	T	0.204	p.Q84X
8223	*WDR89*	80666	14	64066363	stopgain	G	A	0.074	p.R100X
8223	*CHST9*	31422	18	24496522	stopgain	C	A	0.230	p.G345X
8223	*EPG5*	20964	18	43532369	stopgain	G	A	0.264	p.R417X
8223	*CHEK2*	145862	22	29121334	stopgain	C	T	0.245	p.W157X
8223	*SRRM1*	5839	1	24995954	missense	T	A	0.293	p.S694T
8223	*CDCP2*	201546	1	54605318	missense	T	G	0.200	p.M409L
8223	*POGZ*	207171	1	151378326	missense	T	C	0.181	p.E1062G
8223	*INTS3*	23015	1	153713205	missense	G	C	0.255	p.A77P
8223	*MARK1*	18650	1	220835416	missense	T	C	0.220	p.S766P
8223	*PXDN*	12293	2	1670184	missense	C	T	0.130	p.A365T
8223	*PREB*	13388	2	27356499	missense	C	T	0.185	p.A76T
8223	*VWA3B*	144992	2	98709683	missense	A	G	0.184	p.H43R

8223	*UXS1*	25076	2	106742130	missense	C	T	0.054	p.A190T
8223	*WDR33*	18383	2	128477291	missense	G	A	0.125	p.P770S
8223	*CCNT2*	58241	2	135694492	missense	C	A	0.254	p.H108N
8223	*LRP2*	4525	2	170177287	missense	C	G	0.130	p.A63P
8223	*MYO3B*	138995	2	171259408	missense	A	G	0.208	p.E727G
8223	*TTN*	133437	2	179460528	missense	C	A	0.256	p.G19185C
8223	*MOGAT1*	58165	2	223554125	missense	T	C	0.159	p.Y139H
8223	*UGT1A1*	463	2	234669630	missense	C	A	0.167	p.L233I
8223	*MAPK10*	138982	4	87023076	missense	G	A	0.233	p.H179Y
8223	*NEK1*	12224	4	170322917	missense	C	T	0.198	p.E1157K
8223	*SHROOM1*	133456	5	132159415	missense	C	T	0.153	p.R618Q
8223	*SPINK5*	6846	5	147480950	missense	A	C	0.238	p.E418A
8223	*FAM8A1*	16255	6	17601035	missense	A	G	0.075	p.H132R
8223	*FAM8A1*	16255	6	17601040	missense	G	A	0.073	p.G134S
8223	*FAM8A1*	16255	6	17601041	missense	G	T	0.077	p.G134V
8223	*FAM8A1*	16255	6	17601044	missense	T	C	0.077	p.L135P
8223	*FAM8A1*	16255	6	17601058	missense	G	A	0.073	p.A140T
8223	*FAM8A1*	16255	6	17601086	missense	A	G	0.066	p.Q149R
8223	*PHF3*	15153	6	64395368	missense	A	C	0.157	p.D494A
8223	*EIF2AK1*	14413	7	6080713	missense	T	C	0.055	p.N310S
8223	*ABCA13*	152701	7	48494696	missense	G	A	0.156	p.A4210T
8223	*CD36*	1289911	7	80301327	missense	G	A	0.145	p.E366K
8223	*PCLO*	33026	7	82581488	missense	T	A	0.167	p.E2927D
8223	*RP1L1*	178857	8	10467580	missense	T	A	0.054	p.E1343V
8223	*RXRA*	2957	9	137293550	missense	A	T	0.146	p.H34L
8223	*FFAR4*	181745	10	95347335	missense	A	C	0.229	p.K368T
8223	*ZNF143*	3442	11	9530285	missense	G	C	0.128	p.G423R
8223	*USH1C*	153676	11	17530985	missense	A	G	0.176	p.V644A
8223	*DYNC2H1*	1377	11	103152909	missense	A	G	0.196	p.E3595G
8223	*VWF*	552	12	6125377	missense	A	G	0.194	p.V1778A
8223	*ACRBP*	32489	12	6749357	missense	C	A	0.197	p.D432Y
8223	*ADAMTS20*	25003	12	43826430	missense	T	C	0.216	p.R969G
8223	*HDAC7*	15401	12	48189070	missense	C	T	0.241	p.R394Q
8223	*KMT2D*	3482	12	49445374	missense	T	C	0.168	p.T698A
8223	*KRT3*	57088	12	53189427	missense	C	G	0.230	p.A134P
8223	*MYO1A*	5379	12	57430165	missense	C	A	0.129	p.A759S
8223	*CTDSP2*	5730	12	58217396	missense	G	A	0.098	p.R269W
8223	*CTDSP2*	5730	12	58217398	missense	A	T	0.091	p.L268Q
8223	*CTDSP2*	5730	12	58217698	missense	G	T	0.078	p.P227T
8223	*CTDSP2*	5730	12	58217763	missense	C	T	0.258	p.R205H
8223	*CTDSP2*	5730	12	58220811	missense	G	T	0.063	p.L108I
8223	*CTDSP2*	5730	12	58220831	missense	C	G	0.054	p.R101T
8223	*CTDSP2*	5730	12	58240188	missense	G	A	0.136	p.R11W
8223	*OR4N2*	1004723	14	20296506	missense	A	G	0.186	p.K300R
8223	*WDR89*	80666	14	64066326	missense	C	T	0.053	p.G112D
8223	*WDR89*	80666	14	64066352	missense	T	A	0.070	p.R103S
8223	*WDR89*	80666	14	64066367	missense	A	T	0.075	p.D98E
8223	*WDR89*	80666	14	64066395	missense	C	T	0.073	p.C89Y
8223	*WDR89*	80666	14	64066398	missense	G	C	0.075	p.A88G
8223	*WDR89*	80666	14	64066402	missense	A	G	0.075	p.S87P
8223	*TJP1*	175610	15	30024889	missense	C	T	0.053	p.A627T
8223	*FAN1*	14967	15	31200400	missense	G	T	0.180	p.E438D
8223	*PRTG*	173814	15	55974582	missense	G	A	0.162	p.A219V
8223	*MESP2*	1039958	15	90320142	missense	G	C	0.135	p.G185A
8223	*MESP2*	1039958	15	90320144	missense	C	G	0.184	p.Q186E
8223	*LRRK1*	24652	15	101566189	missense	T	C	0.154	p.V751A
8223	*ZNF423*	15069	16	49670566	missense	A	G	0.111	p.F833L
8223	*CNEP1R1*	153261	16	50063692	missense	G	C	0.053	p.D69H
8223	*CNTROB*	53051	17	7838410	missense	C	T	0.147	p.P181S
8223	*ADORA2B*	676	17	15878112	missense	A	C	0.074	p.N152T
8223	*EPX*	502	17	56270883	missense	C	G	0.167	p.R108G
8223	*PRR12*	20719	19	50100531	missense	C	T	0.160	p.P980L
8223	*EPS8L1*	133180	19	55591145	missense	G	A	0.119	p.G69S
8223	*ZNF628*	33113	19	55994860	missense	C	T	0.213	p.A767V
8223	*NINL*	25176	20	25493497	missense	C	A	0.234	p.W141C
8223	*TOX2*	32883	20	42694398	missense	C	T	0.138	p.P294L
8223	*DOK5*	177959	20	53260028	missense	C	T	0.117	p.S148L
8223	*ZBTB21*	20727	21	43412789	missense	A	T	0.064	p.D472E
8223	*INPP5J*	1284286	22	31520984	missense	G	T	0.185	p.A20S
8223	*FAM104B*	138362	X	55172686	missense	C	T	0.186	p.S60N
8223	*FAM104B*	138362	X	55172689	missense	G	T	0.151	p.A59E
8223	*TCEAL5*	1012979	X	102529317	missense	C	T	0.166	p.E59K
8224	*KIF4B*	1099293	5	154394617	stopgain	G	T	0.093	p.E400X
8224	*PARS2*	152268	1	55223862	missense	A	C	0.064	p.F325V
8224	*DNAJC6*	14787	1	65855111	missense	C	G	0.054	p.Q386E
8224	*ANP32E*	30920	1	150199057	missense	C	T	0.053	p.R133K
8224	*IGFN1*	1164586	1	201184240	missense	G	T	0.133	p.V2985L
8224	*MARK1*	18650	1	220826523	missense	G	A	0.078	p.R606Q
8224	*GREB1*	14668	2	11767223	missense	G	A	0.051	p.R1481H
8224	*CRYGD*	6891	2	208986580	missense	G	C	0.072	p.D114E
8224	*PRKCD*	212539	3	53223262	missense	G	T	0.051	p.K581N

8224	*ZNF717*	1290209	3	75788230	missense	C	T	0.118	p.V132I
8224	*AGTR1*	32049	3	148459037	missense	T	A	0.066	p.L107Q
8224	*MME*	7289	3	154898198	missense	T	C	0.056	p.C735R
8224	*APOD*	1647	3	195298170	missense	C	A	0.053	p.K104N
8224	*FGFRL1*	21923	4	1016167	missense	G	A	0.140	p.V86M
8224	*KLB*	175737	4	39449999	missense	G	A	0.102	p.G943E
8224	*DSPP*	14208	4	88537243	missense	C	A	0.124	p.D1143E
8224	*CARTPT*	4291	5	71015710	missense	G	A	0.070	p.E55K
8224	*FAM8A1*	16255	6	17601035	missense	A	G	0.051	p.H132R
8224	*HLA-DQA2*	20056	6	32713061	missense	C	A	0.120	p.Q70K
8224	*HLA-DQB2*	1300790	6	32725636	missense	C	T	0.070	p.S224N
8224	*ZNF451*	15555	6	56993554	missense	G	T	0.068	p.G114W
8224	*INTS1*	1080453	7	1520473	missense	C	T	0.063	p.R1316Q
8224	*RP1L1*	178857	8	10464982	missense	A	G	0.056	p.L2209S
8224	*RP1L1*	178857	8	10465078	missense	A	G	0.156	p.L2177S
8224	*RP1*	6269	8	55541231	missense	G	C	0.072	p.D1597H
8224	*SLC7A13*	138817	8	87226851	missense	T	C	0.086	p.T402A
8224	*ZNF251*	138367	8	145947211	missense	G	T	0.061	p.H612N
8224	*ZNF16*	6958	8	146156611	missense	T	A	0.085	p.E521V
8224	*PIP5K1B*	3558	9	71532569	missense	G	A	0.097	p.D293N
8224	*AGTPBP1*	15239	9	88207536	missense	G	A	0.066	p.P796L
8224	*ZNF462*	21224	9	109690694	missense	G	C	0.058	p.A1501P
8224	*AK8*	152572	9	135601256	missense	T	C	0.141	p.Q420R
8224	*MYO3A*	17433	10	26463103	missense	G	C	0.069	p.E1304Q
8224	*SPOCK2*	14767	10	73823984	missense	C	T	0.089	p.A315T
8224	*ATE1*	7041	10	123673314	missense	T	G	0.108	p.S110R
8224	*PAOX*	207128	10	135202506	missense	T	A	0.053	p.F390I
8224	*SAAL1*	138421	11	18127530	missense	A	T	0.133	p.V20E
8224	*MYBPC3*	256	11	47359008	missense	C	T	0.107	p.V846I
8224	*MYBPC3*	256	11	47363576	missense	G	A	0.073	p.P586S
8224	*CTTN*	138565	11	70275265	missense	A	G	0.085	p.K379R
8224	*ME3*	6680	11	86158130	missense	C	T	0.075	p.E453K
8224	*KCNH3*	12284	12	49948302	missense	A	T	0.052	p.S701C
8224	*KRT3*	57088	12	53189427	missense	C	G	0.080	p.A134P
8224	*CTDSP2*	5730	12	58217763	missense	C	T	0.159	p.R205H
8224	*CTDSP2*	5730	12	58220801	missense	G	T	0.052	p.T111N
8224	*CTDSP2*	5730	12	58220811	missense	G	T	0.069	p.L108I
8224	*CTDSP2*	5730	12	58220819	missense	A	G	0.061	p.V105A
8224	*CTDSP2*	5730	12	58220831	missense	C	A	0.092	p.R101M
8224	*CTDSP2*	5730	12	58240188	missense	G	A	0.060	p.R11W
8224	*PABPC3*	30979	13	25670691	missense	G	T	0.055	p.V119F
8224	*PABPC3*	30979	13	25670703	missense	G	T	0.051	p.G123C
8224	*PABPC3*	30979	13	25670712	missense	C	G	0.051	p.L126V
8224	*WDR89*	80666	14	64066395	missense	C	T	0.050	p.C89Y
8224	*WDR89*	80666	14	64066398	missense	G	C	0.051	p.A88G
8224	*WDR89*	80666	14	64066402	missense	A	G	0.052	p.S87P
8224	*RPAP1*	15540	15	41813991	missense	C	T	0.110	p.E995K
8224	*GNB5*	16194	15	52476780	missense	G	T	0.050	p.Q32K
8224	*MESP2*	1039958	15	90320144	missense	C	G	0.121	p.Q186E
8224	*CHTF18*	22092	16	838769	missense	C	A	0.122	p.L29M
8224	*PRRT2*	145239	16	29824913	missense	G	A	0.126	p.E180K
8224	*COX6A2*	5205	16	31439159	missense	C	T	0.058	p.G77D
8224	*RAI1*	30665	17	17697096	missense	G	C	0.078	p.Q278H
8224	*STAT3*	213662	17	40474420	missense	C	A	0.080	p.D661Y
8224	*LRRC30*	1105581	18	7231350	missense	G	A	0.078	p.E72K
8224	*CACTIN*	21231	19	3626711	missense	C	T	0.126	p.R17Q
8224	*ADAMTS10*	30957	19	8654809	missense	G	A	0.065	p.R622C
8225	*DHX29*	19030	5	54581642	stopgain	G	A	0.235	p.R372X
8225	*UHRF1BP1L*	15054	12	100466506	stopgain	G	C	0.180	p.S498X
8225	*WDR89*	80666	14	64066363	stopgain	G	A	0.056	p.R100X
8225	*KIR2DL4*	2255	19	55320307	stopgain	G	A	0.121	p.W225X
8225	*ANP32E*	30920	1	150199057	missense	C	T	0.067	p.R133K
8225	*AGPS*	3659	2	178372759	missense	G	T	0.059	p.R536M
8225	*SYNPO2*	133477	4	119947964	missense	G	A	0.133	p.R147K
8225	*PCDH18*	19035	4	138451620	missense	G	C	0.267	p.I541M
8225	*FREM3*	1168235	4	144621404	missense	T	A	0.056	p.Q142L
8225	*ITGA1*	181501	5	52145270	missense	G	T	0.075	p.D45Y
8225	*FAM8A1*	16255	6	17601035	missense	A	G	0.056	p.H132R
8225	*FAM8A1*	16255	6	17601040	missense	G	A	0.052	p.G134S
8225	*FAM8A1*	16255	6	17601041	missense	G	T	0.052	p.G134V
8225	*FAM8A1*	16255	6	17601044	missense	T	C	0.050	p.L135P
8225	*FAM8A1*	16255	6	17601058	missense	G	A	0.059	p.A140T
8225	*FAM8A1*	16255	6	17601086	missense	A	G	0.063	p.Q149R
8225	*IBTK*	15525	6	82921225	missense	C	T	0.056	p.V786I
8225	*RP1*	6269	8	55534680	missense	C	T	0.250	p.P207S
8225	*GDF2*	16204	10	48414067	missense	C	A	0.258	p.E267D
8225	*CTBP2*	22802	10	126692019	missense	G	A	0.093	p.P34L
8225	*ARNTL2*	20183	12	27553559	missense	A	G	0.238	p.K338E
8225	*CTDSP2*	5730	12	58217698	missense	G	T	0.094	p.P227T
8225	*CTDSP2*	5730	12	58217763	missense	C	T	0.267	p.R205H
8225	*CTDSP2*	5730	12	58220801	missense	G	T	0.079	p.T111N

8225	*CTDSP2*	5730	12	58220811	missense	G	T	0.070	p.L108I
8225	*CTDSP2*	5730	12	58220819	missense	A	G	0.055	p.V105A
8225	*CTDSP2*	5730	12	58220831	missense	C	G	0.084	p.R101T
8225	*CTDSP2*	5730	12	58240188	missense	G	A	0.093	p.R11W
8225	*HSP90B1*	3299	12	104341190	missense	A	T	0.064	p.E788D
8225	*TBC1D4*	14832	13	75936391	missense	G	T	0.268	p.A284D
8225	*WDR89*	80666	14	64066367	missense	A	T	0.061	p.D98E
8225	*WDR89*	80666	14	64066395	missense	C	T	0.068	p.C89Y
8225	*WDR89*	80666	14	64066398	missense	G	C	0.069	p.A88G
8225	*WDR89*	80666	14	64066402	missense	A	G	0.075	p.S87P
8225	*TEX9*	198524	15	56704590	missense	G	A	0.129	p.E307K
8225	*BAIAP3*	3933	16	1397317	missense	C	A	0.077	p.H959N
8225	*APOBR*	18690	16	28507445	missense	G	C	0.111	p.E361D
8225	*APOBR*	18690	16	28507452	missense	G	T	0.075	p.G364W
8225	*KCTD1*	1142730	18	24126882	missense	A	G	0.057	p.V540A
8225	*KANK3*	198471	19	8400400	missense	C	T	0.066	p.G104D
8225	*SHANK1*	16148	19	51165712	missense	G	C	0.237	p.P1999R
8225	*KIR2DL4*	2255	19	55320306	missense	G	C	0.133	p.W225S
8225	*KIR2DL4*	2255	19	55320308	missense	C	T	0.125	p.P226S
8225	*KIR3DL2*	6737	19	55377265	missense	T	C	0.133	p.C336R
8225	*KIR3DL2*	6737	19	55377280	missense	G	A	0.140	p.V341I
8229	*KDM5B*	6618	1	202702651	stopgain	G	A	0.290	p.Q1263X
8229	*ACSBG1*	15162	15	78466801	stopgain	C	A	0.182	p.E590X
8229	*LMTK3*	1080434	19	49001508	stopgain	C	A	0.098	p.E969X
8229	*SLC45A1*	1080397	1	8385982	missense	G	A	0.154	p.G233S
8229	*HOXD10*	2148	2	176982245	missense	G	C	0.211	p.E228D
8229	*KIAA1919*	153369	6	111587637	missense	G	T	0.174	p.C291F
8229	*NPTX2*	2523	7	98254284	missense	C	T	0.118	p.R232C
8229	*CALU*	1219	7	128394410	missense	T	C	0.160	p.Y114H
8229	*UBN2*	173569	7	138967999	missense	G	T	0.138	p.G783V
8229	*UBN2*	173569	7	138968004	missense	C	T	0.138	p.P785S
8229	*NEFL*	6158	8	24813998	missense	G	A	0.174	p.S11L
8229	*CSMD3*	198124	8	113988302	missense	G	T	0.211	p.A329D
8229	*CSMD3*	198124	8	113988318	missense	T	C	0.235	p.T324A
8229	*FAM120A*	198841	9	96214599	missense	A	C	0.182	p.F131L
8229	*FAM120A*	198841	9	96214626	missense	C	T	0.222	p.M122I
8229	*GRIN3A*	133445	9	104433036	missense	G	A	0.222	p.T553I
8229	*AKAP2*	147150	9	112900432	missense	G	A	0.182	p.V870I
8229	*OLFM1*	14279	9	137998639	missense	A	G	0.261	p.T223A
8229	*HNRNPF*	4966	10	43882621	missense	T	C	0.222	p.T238A
8229	*HNRNPF*	4966	10	43882646	missense	T	A	0.211	p.E229D
8229	*LZTS2*	32429	10	102763609	missense	T	C	0.125	p.S252P
8229	*OR51E1*	152430	11	4674060	missense	C	G	0.182	p.L102V
8229	*OR5P3*	153445	11	7846768	missense	A	T	0.188	p.F251Y
8229	*OR5P3*	153445	11	7846826	missense	T	C	0.250	p.K232E
8229	*HIPK3*	5734	11	33374842	missense	A	T	0.148	p.T1126S
8229	*MUS81*	25128	11	65632742	missense	G	C	0.231	p.V485L
8229	*MUS81*	25128	11	65632766	missense	C	G	0.233	p.L493V
8229	*CTDSP2*	5730	12	58217698	missense	G	T	0.222	p.P227T
8229	*CTDSP2*	5730	12	58217770	missense	G	C	0.175	p.L203V
8229	*HSPA2*	21979	14	65008111	missense	A	C	0.196	p.I182L
8229	*PEAK1*	24776	15	77425525	missense	T	C	0.200	p.K1300R
8229	*PEAK1*	24776	15	77425556	missense	T	G	0.200	p.I1290L
8229	*MYLK3*	182493	16	46764595	missense	G	A	0.222	p.P493L
8229	*IRX6*	24335	16	55362984	missense	G	C	0.200	p.G365A
8229	*CDH11*	1797	16	65032548	missense	A	T	0.200	p.V147D
8229	*FAM222B*	18182	17	27085327	missense	C	A	0.133	p.E422D
8229	*MRPS23*	16070	17	55917265	missense	G	T	0.211	p.S151Y
8229	*NDUFS7*	24407	19	1390927	missense	A	G	0.261	p.M96V
8229	*ZNF575*	174945	19	44039702	missense	C	T	0.276	p.P201S
8229	*KLK2*	5551	19	51378030	missense	C	A	0.211	p.H34N
8229	*ZBED4*	14838	22	50279289	missense	G	A	0.167	p.S660N
8229	*ZBED4*	14838	22	50279333	missense	T	C	0.235	p.F675L
8234	*PARP6*	20214	15	72552908	stopgain	C	A	0.105	p.E223X
8234	*AP3B2*	4644	15	83346477	stopgain	G	A	0.148	p.R442X
8234	*TNRC6B*	15088	22	40704647	stopgain	A	T	0.068	p.K614X
8234	*SZT2*	15284	1	43892466	missense	G	A	0.052	p.A1042T
8234	*FLG*	2016	1	152278762	missense	A	C	0.079	p.I2867S
8234	*FLG*	2016	1	152278772	missense	G	A	0.067	p.H2864Y
8234	*KDM5B*	6618	1	202722082	missense	T	C	0.155	p.Q551R
8234	*TRANK1*	14831	3	36900272	missense	T	C	0.121	p.I493V
8234	*CELSR3*	1407	3	48694259	missense	G	A	0.068	p.P1424L
8234	*ZNF717*	1290209	3	75788230	missense	C	T	0.100	p.V132I
8234	*KIAA0226*	14687	3	197401980	missense	G	C	0.071	p.A898G
8234	*MFAP3L*	21647	4	170912543	missense	C	T	0.188	p.E406K
8234	*BMP6*	1718	6	7727516	missense	C	G	0.054	p.Q110E
8234	*VARS*	6295	6	31748301	missense	G	T	0.057	p.H948N
8234	*VARS*	6295	6	31749302	missense	C	T	0.070	p.V834I
8234	*PPP2R5D*	180977	6	42978300	missense	A	G	0.070	p.K460E
8234	*ZNF318*	14345	6	43306014	missense	G	T	0.103	p.P1908T
8234	*TFAP2B*	3221	6	50791525	missense	G	T	0.060	p.G163C

8234	*NT5E*	2526	6	86203600	missense	A	G	0.133	p.M535V
8234	*EPHX2*	1979	8	27382958	missense	G	T	0.050	p.V380L
8234	*OPTN*	21980	10	13151198	missense	C	G	0.075	p.H26D
8234	*SKIDA1*	207371	10	21805466	missense	C	T	0.143	p.G429E
8234	*OGDHL*	18245	10	50953473	missense	G	T	0.067	p.Q516K
8234	*MTG1*	138384	10	135207827	missense	A	C	0.076	p.M35L
8234	*CD5*	14207	11	60893246	missense	T	C	0.061	p.S475P
8234	*NANOGNB*	1145465	12	7917933	missense	G	A	0.070	p.E18K
8234	*TSFM*	5726	12	58176630	missense	G	A	0.078	p.G16R
8234	*CTDSP2*	5730	12	58217698	missense	G	T	0.071	p.P227T
8234	*CTDSP2*	5730	12	58217763	missense	C	T	0.203	p.R205H
8234	*CTDSP2*	5730	12	58220801	missense	G	T	0.094	p.T111N
8234	*CTDSP2*	5730	12	58220811	missense	G	T	0.167	p.L108I
8234	*CTDSP2*	5730	12	58220831	missense	C	A	0.114	p.R101M
8234	*OAS1*	16816	12	113346378	missense	G	T	0.107	p.R73L
8234	*PTPN21*	7039	14	88935980	missense	C	T	0.069	p.R1033H
8234	*MESP2*	1039958	15	90320144	missense	C	G	0.135	p.Q186E
8234	*IDH2*	2168	15	90630400	missense	A	T	0.159	p.F304Y
8234	*ITPRIPL2*	1034841	16	19126778	missense	C	T	0.085	p.A332V
8234	*PRPF8*	6445	17	1579819	missense	G	A	0.051	p.R790W
8234	*PELP1*	14389	17	4579124	missense	A	C	0.054	p.C199G
8234	*KIAA0100*	14680	17	26960681	missense	C	A	0.074	p.K1117N
8234	*MED1*	4774	17	37565055	missense	G	C	0.100	p.S1140C
8234	*NDUFS7*	24407	19	1390996	missense	G	C	0.141	p.V119L
8234	*NDUFS7*	24407	19	1393289	missense	G	C	0.064	p.R168S
8234	*KDM4B*	15015	19	5071042	missense	C	G	0.100	p.H216Q
8234	*USHBP1*	31941	19	17367384	missense	G	A	0.054	p.P456S
8234	*GPI*	1289790	19	34890626	missense	A	C	0.052	p.M532L
8234	*GPI*	1289790	19	34890628	missense	G	C	0.167	p.M532I
8234	*TPRX1*	198479	19	48305624	missense	A	T	0.120	p.I215N
8234	*VSIG10L*	1163922	19	51842384	missense	C	A	0.118	p.G496V
8234	*PYGB*	2862	20	25273104	missense	G	A	0.110	p.G678S
8742	*PRMT6*	18137	1	107599501	stopgain	C	A	0.100	p.S55X
8742	*LBX2*	1282430	2	74726463	stopgain	C	A	0.154	p.E68X
8742	*DCHS2*	17639	4	155156181	stopgain	G	T	0.138	p.S2753X
8742	*FBN2*	1999	5	127710336	stopgain	C	A	0.138	p.G694X
8742	*TGFBI*	358	5	135382088	stopgain	C	A	0.174	p.Y121X
8742	*PCSK5*	6200	9	78506128	stopgain	G	T	0.174	p.G11X
8742	*FAM196A*	1039762	10	128973889	stopgain	G	T	0.105	p.Y257X
8742	*ANAPC5*	16237	12	121790117	stopgain	G	T	0.154	p.Y9X
8742	*HHIPL1*	32425	14	100118650	stopgain	C	A	0.167	p.C115X
8742	*PABPN1L*	1294328	16	88931980	stopgain	G	T	0.129	p.Y150X
8742	*CELSR1*	14246	22	46790048	stopgain	G	T	0.118	p.C1985X
8742	*GPR153*	207370	1	6314791	missense	C	T	0.118	p.V59M
8742	*CELA2A*	33440	1	15793931	missense	G	T	0.111	p.Q230H
8742	*LCK*	5356	1	32742270	missense	G	T	0.148	p.D283Y
8742	*BEST4*	153274	1	45253257	missense	C	A	0.133	p.A41S
8742	*UBAP2L*	14847	1	154207713	missense	G	A	0.167	p.G164R
8742	*PRRC2C*	15172	1	171501914	missense	C	A	0.167	p.Q561K
8742	*FBXO41*	1080410	2	73487950	missense	C	A	0.143	p.Q736H
8742	*CNNM4*	20184	2	97427326	missense	G	A	0.111	p.G197D
8742	*TGFBRAP1*	4257	2	105924428	missense	G	T	0.167	p.P111T
8742	*TRPM8*	24080	2	234873293	missense	G	A	0.143	p.A591T
8742	*BRPF1*	4634	3	9784849	missense	G	T	0.118	p.Q735H
8742	*CLDN11*	5602	3	170141049	missense	G	A	0.200	p.G109S
8742	*FGFRL1*	21923	4	1019049	missense	G	C	0.143	p.D477H
8742	*SH3TC1*	18986	4	8229780	missense	C	A	0.118	p.P787T
8742	*ANK2*	1148	4	114275589	missense	G	A	0.182	p.V1939I
8742	*TRIO*	7118	5	14461146	missense	G	T	0.133	p.S1741I
8742	*FBXL7*	12304	5	15937252	missense	G	T	0.138	p.C478F
8742	*PCDHGA2*	32009	5	140718811	missense	C	A	0.167	p.D91E
8742	*SLC6A7*	14228	5	149585083	missense	C	A	0.182	p.P533H
8742	*LMAN2*	6816	5	176778484	missense	C	A	0.118	p.K55N
8742	*HIST1H3H*	3536	6	27777904	missense	G	A	0.125	p.R18H
8742	*BRPF3*	15695	6	36168330	missense	C	A	0.111	p.N77K
8742	*MDFI*	5586	6	41621211	missense	C	A	0.073	p.D213E
8742	*DOPEY1*	15018	6	83877616	missense	G	T	0.167	p.L2387F
8742	*TSPYL4*	21648	6	116574504	missense	C	A	0.167	p.R223M
8742	*ARID1B*	20732	6	157099497	missense	G	A	0.108	p.G145D
8742	*FNDC1*	32532	6	159660756	missense	C	A	0.125	p.T1463N
8742	*ZNF282*	3575	7	148921353	missense	C	T	0.125	p.R544W
8742	*ERICH1*	207332	8	623664	missense	T	C	0.105	p.R230G
8742	*SGK223*	1080826	8	8234037	missense	C	A	0.121	p.A628S
8742	*NKX3-1*	6167	8	23538975	missense	G	T	0.138	p.A80D
8742	*MROH6*	1100878	8	144652416	missense	G	C	0.143	p.A347G
8742	*SLC52A2*	24531	8	145584487	missense	G	A	0.100	p.G384S
8742	*CDK9*	1261	9	130551745	missense	C	A	0.154	p.Q348K
8742	*WDR34*	52844	9	131396150	missense	G	T	0.118	p.T495N
8742	*SURF4*	33161	9	136242815	missense	G	A	0.154	p.T9M
8742	*ARHGAP22*	21226	10	49667783	missense	G	T	0.138	p.N201K
8742	*NODAL*	18055	10	72195308	missense	C	A	0.111	p.G209W

8742	*CDH23*	52836	10	73326586	missense	C	A	0.148	p.P173T
8742	*DNAJC9*	15190	10	75006880	missense	C	A	0.182	p.R23L
8742	*CALHM1*	1001412	10	105215453	missense	G	T	0.105	p.R203S
8742	*SH3PXD2A*	14631	10	105362265	missense	G	T	0.154	p.P876T
8742	*COL17A1*	494	10	105801078	missense	C	A	0.129	p.G877V
8742	*PHRF1*	20901	11	607951	missense	C	A	0.100	p.P831Q
8742	*TMEM132A*	178031	11	60701078	missense	G	A	0.091	p.R474Q
8742	*FADS2*	4265	11	61631233	missense	C	G	0.143	p.H378D
8742	*TIGD3*	145719	11	65124393	missense	C	A	0.095	p.P372T
8742	*SNX32*	152760	11	65618581	missense	G	T	0.111	p.R220L
8742	*CARNS1*	20811	11	67187268	missense	G	T	0.093	p.V358L
8742	*NDUFS8*	2496	11	67803789	missense	A	G	0.100	p.T148A
8742	*NDUFS8*	2496	11	67803790	missense	C	T	0.102	p.T148I
8742	*NDUFS8*	2496	11	67803810	missense	T	C	0.116	p.F155L
8742	*NDUFS8*	2496	11	67803812	missense	C	G	0.114	p.F155L
8742	*PHLDB1*	15157	11	118514555	missense	C	A	0.148	p.P972H
8742	*SORL1*	3105	11	121460062	missense	G	T	0.167	p.M1347I
8742	*SORL1*	3105	11	121474927	missense	G	T	0.129	p.M1515I
8742	*NINJ2*	16533	12	772597	missense	G	T	0.174	p.A23E
8742	*GPR162*	19858	12	6933440	missense	C	G	0.105	p.R126G
8742	*C12orf57*	138425	12	7053335	missense	G	T	0.160	p.K17N
8742	*LPCAT3*	5768	12	7125700	missense	C	T	0.121	p.G10E
8742	*FOXJ2*	18416	12	8202111	missense	G	T	0.125	p.R494L
8742	*NCKAP5L*	1037806	12	50188843	missense	G	T	0.129	p.R934S
8742	*KRT1*	6121	12	53069161	missense	C	A	0.125	p.S584I
8742	*NFE2*	6163	12	54686517	missense	C	A	0.174	p.A255S
8742	*NFE2*	6163	12	54686606	missense	C	A	0.138	p.R225L
8742	*CDK2*	52827	12	56362706	missense	G	T	0.182	p.V154F
8742	*ATP5B*	1686	12	57039057	missense	C	T	0.154	p.V70M
8742	*DTX3*	178502	12	58002436	missense	G	A	0.148	p.G295D
8742	*CTDSP2*	5730	12	58217770	missense	G	T	0.163	p.L203I
8742	*CTDSP2*	5730	12	58220801	missense	G	T	0.200	p.T111N
8742	*KCNMB4*	14505	12	70760817	missense	C	A	0.108	p.H101Q
8742	*TMEM119*	181724	12	108985450	missense	G	A	0.154	p.S237L
8742	*KSR2*	173598	12	118105410	missense	C	A	0.108	p.S318I
8742	*UBAC2*	177967	13	99853763	missense	G	T	0.125	p.G33V
8742	*IL25*	172314	14	23844968	missense	C	A	0.125	p.T122N
8742	*ZFHX2*	33400	14	23992091	missense	C	A	0.182	p.V2267L
8742	*PLEKHG3*	15549	14	65209783	missense	G	A	0.121	p.G952R
8742	*SIPA1L1*	15556	14	72200404	missense	G	T	0.133	p.S1649I
8742	*VRTN*	18228	14	74823993	missense	C	A	0.095	p.S169R
8742	*LTBP2*	428	14	74973927	missense	C	A	0.103	p.G1288C
8742	*C14orf178*	174943	14	78235848	missense	G	A	0.154	p.A66T
8742	*RTL1*	1134888	14	101347478	missense	C	A	0.174	p.Q1216H
8742	*RPAP1*	15540	15	41813182	missense	C	A	0.108	p.A1068S
8742	*RGMA*	20211	15	93595629	missense	C	T	0.129	p.R80H
8742	*VASN*	138440	16	4431744	missense	G	A	0.118	p.R289H
8742	*PRRT2*	145239	16	29824547	missense	C	A	0.121	p.P58T
8742	*HSD3B7*	25193	16	30999412	missense	C	A	0.167	p.R340S
8742	*MYLK3*	182493	16	46764569	missense	C	A	0.138	p.V502L
8742	*SLC12A3*	1126108	16	56902284	missense	G	T	0.105	p.V169F
8742	*GPR97*	170776	16	57717969	missense	C	A	0.160	p.A336D
8742	*ENKD1*	32140	16	67698989	missense	G	T	0.108	p.S121R
8742	*HYDIN*	17558	16	71103286	missense	C	A	0.125	p.V620L
8742	*WDR81*	1163809	17	1630596	missense	G	T	0.167	p.M781I
8742	*USP43*	153210	17	9604699	missense	C	T	0.160	p.H565Y
8742	*GPR179*	1004334	17	36491566	missense	G	T	0.125	p.F438L
8742	*MARCH10*	152598	17	60814539	missense	C	A	0.121	p.E229D
8742	*UNK*	1080419	17	73808261	missense	G	T	0.154	p.G128V
8742	*CBX4*	3655	17	77808206	missense	C	A	0.148	p.R412L
8742	*TBC1D16*	19020	17	77984248	missense	G	T	0.129	p.L164M
8742	*S1PR4*	3775	19	3179353	missense	G	T	0.111	p.C188F
8742	*ANKRD24*	133475	19	4222722	missense	C	T	0.160	p.P1076L
8742	*CLPP*	6012	19	6361895	missense	G	T	0.206	p.A72S
8742	*PNPLA6*	6702	19	7616308	missense	C	A	0.108	p.Q710K
8742	*PNPLA6*	6702	19	7623993	missense	G	C	0.129	p.D1181H
8742	*FDX1L*	1031734	19	10426568	missense	C	A	0.118	p.E38D
8742	*ZNF653*	138783	19	11597646	missense	C	T	0.143	p.E465K
8742	*STX10*	3765	19	13256119	missense	G	T	0.143	p.Q152K
8742	*MAP1S*	18174	19	17844157	missense	G	T	0.148	p.D982Y
8742	*B3GNT3*	14256	19	17922782	missense	G	T	0.114	p.A324S
8742	*KIAA1683*	25249	19	18368069	missense	C	A	0.133	p.C1155F
8742	*KLHL26*	18316	19	18779582	missense	G	T	0.143	p.G459W
8742	*LRP3*	2333	19	33697105	missense	C	A	0.129	p.Q477K
8742	*PSMC4*	153001	19	40485845	missense	C	A	0.105	p.D234E
8742	*B3GNT8*	198540	19	41931979	missense	G	T	0.105	p.H235Q
8742	*PNMAL2*	20709	19	46998050	missense	C	A	0.100	p.A225S
8742	*STRN4*	13403	19	47224011	missense	G	T	0.160	p.R704S
8742	*LAIR1*	21706	19	54872712	missense	G	A	0.148	p.R59C
8742	*PPP6R1*	14931	19	55758453	missense	G	T	0.093	p.L7M
8742	*RNF225*	1195135	19	58907676	missense	G	T	0.108	p.V74L

8742	*ZNF324*	14347	19	58983484	missense	C	T	0.105	p.S542L
8742	*ZBTB45*	32792	19	59028943	missense	C	A	0.103	p.C33F
8742	*DDRGK1*	23935	20	3171355	missense	C	A	0.160	p.A297S
8742	*TNNC2*	3279	20	44452694	missense	G	T	0.174	p.D129E
8742	*CDH4*	1794	20	60318753	missense	G	T	0.167	p.V102L
8742	*KRTAP10-10*	181688	21	46057385	missense	G	T	0.125	p.W17C
8742	*KIAA1671*	1145206	22	25424032	missense	G	T	0.129	p.M22I
8742	*CCDC157*	1017437	22	30762010	missense	C	A	0.154	p.S7R
8742	*TMEM184B*	12264	22	38617518	missense	G	T	0.154	p.D328E
8742	*TBC1D22A*	14346	22	47189494	missense	G	T	0.133	p.W42C
8742	*FAM104B*	138362	X	55172689	missense	G	T	0.227	p.A59E
8743	*ABHD10*	18394	3	111710274	stopgain	C	A	0.060	p.Y209X
8743	*COL4A3BP*	31361	5	74677854	stopgain	G	A	0.082	p.R641X
8743	*NSMAF*	3580	8	59496673	stopgain	G	A	0.087	p.Q916X
8743	*WDR89*	80666	14	64066363	stopgain	G	A	0.120	p.R100X
8743	*CFHR5*	30787	1	196977695	missense	C	A	0.059	p.A531E
8743	*SLC30A3*	3459	2	27481707	missense	C	A	0.051	p.G64V
8743	*COBLL1*	14900	2	165551552	missense	C	T	0.053	p.A889T
8743	*PAX3*	181461	2	223163322	missense	C	A	0.055	p.A5S
8743	*LRRC2*	24512	3	46586717	missense	G	A	0.063	p.A51V
8743	*PTPRG*	2841	3	62267282	missense	G	A	0.179	p.M1270I
8743	*ZNF717*	1290209	3	75788230	missense	C	T	0.070	p.V132I
8743	*NKX6-1*	6168	4	85419342	missense	C	T	0.059	p.A14T
8743	*ARHGAP26*	15071	5	142264883	missense	C	A	0.070	p.D135E
8743	*FAM8A1*	16255	6	17601035	missense	A	G	0.081	p.H132R
8743	*FAM8A1*	16255	6	17601040	missense	G	A	0.077	p.G134S
8743	*FAM8A1*	16255	6	17601041	missense	G	T	0.078	p.G134V
8743	*FAM8A1*	16255	6	17601044	missense	T	C	0.078	p.L135P
8743	*FAM8A1*	16255	6	17601058	missense	G	A	0.085	p.A140T
8743	*FAM8A1*	16255	6	17601086	missense	A	G	0.070	p.Q149R
8743	*FAM8A1*	16255	6	17601098	missense	C	T	0.070	p.S153L
8743	*VARS*	6295	6	31749302	missense	C	T	0.076	p.V834I
8743	*HLA-DQB1*	2123	6	32632769	missense	T	C	0.140	p.Y62C
8743	*HLA-DQA2*	20056	6	32713061	missense	C	A	0.061	p.Q70K
8743	*NDUFAF6*	152416	8	96060712	missense	C	T	0.052	p.R248W
8743	*NFIB*	5596	9	14120495	missense	C	A	0.098	p.D397Y
8743	*CACNA1B*	1243812	9	140904506	missense	G	A	0.063	p.A713T
8743	*ANKRD30A*	52997	10	37414926	missense	G	A	0.095	p.A15T
8743	*CTDSP2*	5730	12	58217698	missense	G	T	0.060	p.P227T
8743	*CTDSP2*	5730	12	58217763	missense	C	T	0.197	p.R205H
8743	*CTDSP2*	5730	12	58217770	missense	G	T	0.091	p.L203I
8743	*CTDSP2*	5730	12	58220801	missense	G	T	0.066	p.T111N
8743	*CTDSP2*	5730	12	58220819	missense	A	G	0.062	p.V105A
8743	*CTDSP2*	5730	12	58240188	missense	G	A	0.108	p.R11W
8743	*DDHD1*	30637	14	53619495	missense	T	C	0.053	p.S108G
8743	*WDR89*	80666	14	64066352	missense	T	A	0.084	p.R103S
8743	*WDR89*	80666	14	64066367	missense	A	T	0.118	p.D98E
8743	*WDR89*	80666	14	64066395	missense	C	T	0.176	p.C89Y
8743	*WDR89*	80666	14	64066398	missense	G	C	0.176	p.A88G
8743	*WDR89*	80666	14	64066402	missense	A	G	0.180	p.S87P
8743	*C14orf180*	1286399	14	105055138	missense	A	G	0.108	p.T153A
8743	*MEF2A*	5587	15	100252744	missense	C	A	0.106	p.P421Q
8743	*SEC14L5*	14692	16	5058630	missense	G	A	0.058	p.R594Q
8743	*HYDIN*	1270974	16	71015346	missense	G	C	0.182	p.S1486R
8743	*NDUFS7*	24407	19	1391020	missense	A	G	0.085	p.T127A
8743	*NDUFS7*	24407	19	1391021	missense	C	T	0.085	p.T127I
8743	*NDUFS7*	24407	19	1393289	missense	G	C	0.110	p.R168S
8743	*TPRX1*	198479	19	48305690	missense	C	A	0.154	p.G193V
8743	*TPRX1*	198479	19	48305696	missense	A	T	0.101	p.I191N
8743	*NAPSA*	4851	19	50864975	missense	G	A	0.056	p.A138V
8743	*ZNF577*	32679	19	52383622	missense	G	A	0.065	p.T5M
8743	*MCM8*	1281521	20	5953323	missense	G	C	0.105	p.C442S
8743	*SEPT3*	145733	22	42383224	missense	G	A	0.056	p.G116S
8743	*MID1*	33290	X	10463718	missense	C	T	0.051	p.R257H
8743	*FAM104B*	1166703	X	55172521	missense	G	T	0.207	p.T114K
8743	*FAM104B*	138362	X	55172670	missense	G	C	0.070	p.N65K
8743	*FAM104B*	138362	X	55172686	missense	C	T	0.146	p.S60N
9612	*KCNJ3*	2239	2	155711794	stopgain	T	G	0.138	p.L492X
9612	*WDR89*	80666	14	64066363	stopgain	G	A	0.095	p.R100X
9612	*ABCA3*	1089	16	2350068	stopgain	C	A	0.179	p.E517X
9612	*PADI2*	7365	1	17445817	missense	G	A	0.103	p.A17V
9612	*POGZ*	207171	1	151403237	missense	T	A	0.188	p.T122S
9612	*LCE2A*	178428	1	152671555	missense	T	C	0.158	p.C60R
9612	*IVL*	5547	1	152883178	missense	A	C	0.196	p.Q302P
9612	*LTBP1*	206943	2	33572476	missense	C	G	0.131	p.A1300G
9612	*LTBP1*	206943	2	33572490	missense	G	T	0.103	p.V1305L
9612	*EML6*	1039753	2	55086815	missense	G	A	0.053	p.G601D
9612	*INPP4A*	4027	2	99181226	missense	G	A	0.135	p.G684R
9612	*LRP2*	4525	2	170025060	missense	A	C	0.197	p.L3875R
9612	*HIBCH*	198047	2	191116997	missense	G	A	0.074	p.P185L
9612	*AGAP1*	1244888	2	236761414	missense	C	G	0.092	p.P379A

9612	*ZNF717*	1290209	3	75787351	missense	C	A	0.156	p.G425W
9612	*ZNF717*	1290209	3	75788230	missense	C	T	0.117	p.V132I
9612	*IGSF11*	152538	3	118647457	missense	T	A	0.224	p.N107I
9612	*KBTBD12*	207335	3	127702942	missense	C	T	0.172	p.R565C
9612	*GPR125*	145290	4	22390385	missense	A	T	0.277	p.I970K
9612	*LRRC66*	1024611	4	52861083	missense	T	G	0.213	p.D702A
9612	*DSPP*	14208	4	88537243	missense	C	A	0.210	p.D1143E
9612	*TET2*	1127208	4	106180868	missense	A	C	0.139	p.K1299T
9612	*FREM3*	1168235	4	144619831	missense	T	G	0.156	p.Q666H
9612	*ASIC5*	17419	4	156757897	missense	A	T	0.172	p.S393R
9612	*C6*	1115131	5	41143055	missense	T	G	0.238	p.N893H
9612	*ARAP3*	22481	5	141059217	missense	A	G	0.202	p.I198T
9612	*ZDHHC2*	16353	8	17055093	missense	A	G	0.200	p.I126V
9612	*FOCAD*	17794	9	20944649	missense	G	C	0.286	p.G1144A
9612	*KLF9*	1206	9	73027931	missense	G	A	0.210	p.P117S
9612	*NTRK2*	6180	9	87549127	missense	G	A	0.147	p.E562K
9612	*PANX3*	52959	11	124481460	missense	T	G	0.216	p.L3R
9612	*PTPRO*	30667	12	15637067	missense	C	T	0.062	p.P79S
9612	*CTDSP2*	5730	12	58217763	missense	C	T	0.243	p.R205H
9612	*CTDSP2*	5730	12	58220811	missense	G	T	0.088	p.L108I
9612	*CTDSP2*	5730	12	58220819	missense	A	G	0.096	p.V105A
9612	*MEDAG*	32849	13	31480705	missense	C	T	0.217	p.S18L
9612	*PCDH9*	203487	13	67205347	missense	T	C	0.317	p.N1112S
9612	*EDNRB*	3991	13	78492693	missense	T	G	0.218	p.S96R
9612	*OR4M1*	1005500	14	20248876	missense	A	G	0.218	p.Y132C
9612	*TMEM55B*	144568	14	20929400	missense	C	T	0.316	p.G29R
9612	*NYNRIN*	25081	14	24886260	missense	A	G	0.168	p.T1769A
9612	*WDR89*	80666	14	64066326	missense	C	T	0.069	p.G112D
9612	*WDR89*	80666	14	64066352	missense	T	A	0.074	p.R103S
9612	*WDR89*	80666	14	64066367	missense	A	T	0.100	p.D98E
9612	*MED6*	5466	14	71059660	missense	A	G	0.263	p.S135P
9612	*CATSPERB*	24764	14	92136213	missense	G	T	0.070	p.P411H
9612	*ADAM10*	1110	15	58889762	missense	C	T	0.231	p.G744E
9612	*PGPEP1L*	1102612	15	99514279	missense	C	T	0.217	p.D44N
9612	*NKD1*	33119	16	50659487	missense	G	A	0.100	p.R153Q
9612	*HYDIN*	1270974	16	70902609	missense	C	G	0.160	p.R3725P
9612	*KLHDC4*	17566	16	87799495	missense	A	T	0.075	p.M1K
9612	*ALOX12B*	1139	17	7989458	missense	G	T	0.192	p.F76L
9612	*RAI1*	30665	17	17697096	missense	G	C	0.085	p.Q278H
9612	*FNDC8*	17559	17	33448770	missense	G	C	0.239	p.E20Q
9612	*STAT3*	213662	17	40475070	missense	T	G	0.158	p.S614R
9612	*PIEZO2*	22068	18	10762939	missense	A	G	0.240	p.F1010S
9612	*NDUFA7*	5001	19	8381467	missense	C	T	0.224	p.C55Y
9612	*CYP2F1*	774	19	41622481	missense	G	A	0.222	p.R98H
9612	*PEG3*	6210	19	57326252	missense	T	G	0.159	p.E1186D
9612	*KIF16B*	24704	20	16493499	missense	C	A	0.207	p.D140Y
9612	*CFAP61*	15585	20	20209013	missense	G	T	0.130	p.V685L
9612	*COL6A2*	58175	21	47532060	missense	C	T	0.198	p.R95C
9613	*RLF*	12421	1	40702415	missense	G	A	0.089	p.V681M
9613	*SLC5A9*	1135181	1	48697295	missense	T	C	0.111	p.L255P
9613	*HMCN1*	31935	1	186047301	missense	G	C	0.114	p.A2850P
9613	*RND3*	5168	2	151326571	missense	C	A	0.078	p.S222I
9613	*ZNF717*	1290209	3	75787351	missense	C	A	0.106	p.G425W
9613	*DSPP*	14208	4	88537243	missense	C	A	0.250	p.D1143E
9613	*ATOH1*	5172	4	94751107	missense	C	T	0.086	p.H344Y
9613	*FREM3*	1168235	4	144621404	missense	T	A	0.058	p.Q142L
9613	*ARRDC3*	20801	5	90678707	missense	T	A	0.060	p.N68I
9613	*PCDHA11*	31861	5	140249929	missense	G	C	0.063	p.R414P
9613	*BCKDHB*	183050	6	80878682	missense	G	A	0.114	p.G190S
9613	*OR2F1*	12369	7	143657490	missense	A	G	0.067	p.R143G
9613	*AGPAT5*	18361	8	6612599	missense	T	C	0.103	p.I258T
9613	*SLCO1C1*	17435	12	20868184	missense	A	T	0.085	p.D214V
9613	*CTDSP2*	5730	12	58217763	missense	C	T	0.307	p.R205H
9613	*CTDSP2*	5730	12	58217770	missense	G	C	0.065	p.L203V
9613	*CTDSP2*	5730	12	58220811	missense	G	T	0.081	p.L108I
9613	*CTDSP2*	5730	12	58220831	missense	C	A	0.069	p.R101M
9613	*CTDSP2*	5730	12	58240184	missense	C	A	0.060	p.R12M
9613	*CTDSP2*	5730	12	58240188	missense	G	A	0.182	p.R11W
9613	*METTL21C*	1010977	13	103339328	missense	G	A	0.074	p.A121V
9613	*MAP1A*	2373	15	43817807	missense	T	A	0.083	p.V1379E
9613	*LDHAL6B*	33195	15	59500155	missense	G	A	0.077	p.S339N
9613	*CIITA*	1286402	16	11001949	missense	G	A	0.050	p.G868D
9613	*NAE1*	3905	16	66844662	missense	C	A	0.067	p.A344S
9613	*HPR*	20995	16	72110615	missense	C	G	0.052	p.Q228E
9613	*CDH13*	1257	16	83159087	missense	T	A	0.078	p.F202Y
9613	*ABR*	21962	17	959322	missense	A	G	0.103	p.F505S
9613	*MRC2*	6039	17	60757217	missense	G	T	0.105	p.R751L
9613	*CTDP1*	48368	18	77475372	missense	G	A	0.053	p.A638T
9613	*LILRA2*	6866	19	55087544	missense	T	A	0.148	p.L408H
9613	*LILRA2*	6866	19	55098686	missense	C	A	0.093	p.P425H
9614	*CYP4X1*	178033	1	47501792	missense	C	T	0.268	p.P242S

9614	*ABCB11*	3742	2	169783865	missense	T	A	0.124	p.D1140V
9614	*ZNF717*	1290209	3	75787351	missense	C	A	0.163	p.G425W
9614	*ZNF717*	1290209	3	75788230	missense	C	T	0.141	p.V132I
9614	*DSPP*	14208	4	88537243	missense	C	A	0.211	p.D1143E
9614	*GUCY1B3*	1291954	4	156696196	missense	G	A	0.083	p.V32I
9614	*GABRB2*	21911	5	160757996	missense	G	A	0.074	p.A324V
9614	*RIPPLY2*	1009994	6	84567052	missense	T	C	0.150	p.F111L
9614	*KIAA1147*	1080392	7	141362499	missense	C	T	0.052	p.G442D
9614	*ATAD2*	14109	8	124382170	missense	A	T	0.174	p.D274E
9614	*EPPK1*	31308	8	144942798	missense	T	C	0.259	p.K1542E
9614	*SORL1*	3105	11	121430309	missense	G	A	0.222	p.A998T
9614	*OR10G9*	1001953	11	123894452	missense	A	G	0.313	p.I245V
9614	*PLEKHG6*	18173	12	6436949	missense	C	T	0.067	p.R734W
9614	*CTDSP2*	5730	12	58217763	missense	C	T	0.191	p.R205H
9614	*CTDSP2*	5730	12	58217770	missense	G	C	0.055	p.L203V
9614	*CTDSP2*	5730	12	58220811	missense	G	T	0.118	p.L108I
9614	*CTDSP2*	5730	12	58220831	missense	C	G	0.129	p.R101T
9614	*CTDSP2*	5730	12	58240188	missense	G	A	0.091	p.R11W
9614	*DCLK1*	4734	13	36686082	missense	A	G	0.053	p.V216A
9614	*WDR89*	80666	14	64066398	missense	G	C	0.056	p.A88G
9614	*MARK3*	2376	14	103931953	missense	C	A	0.129	p.S200R
9614	*MAP1A*	2373	15	43814600	missense	C	T	0.119	p.P310L
9614	*PPM1E*	14906	17	56833485	missense	C	T	0.092	p.P43S
9614	*ACTG1*	1614	17	79478592	missense	G	A	0.077	p.L142F
9614	*CDH20*	31891	18	59221783	missense	G	A	0.068	p.G754D
9614	*C19orf71*	1135580	19	3539176	missense	A	G	0.200	p.M1V
9614	*LPAR2*	4720	19	19737507	missense	G	A	0.059	p.S196L
9614	*FTL*	146	19	49469580	missense	A	G	0.057	p.K98E
9614	*FTL*	146	19	49469589	missense	A	T	0.053	p.M101L
9614	*FTL*	146	19	49469595	missense	C	A	0.052	p.L103M
9614	*SEC23B*	32986	20	18507131	missense	G	T	0.063	p.D317Y
9614	*ZNF831*	178457	20	57766922	missense	C	T	0.053	p.A283V
9615	*KIF2C*	6845	1	45220458	stopgain	C	T	0.167	p.R234X
9615	*TMCC1*	1017395	3	129546966	stopgain	G	A	0.268	p.Q86X
9615	*WRN*	553	8	30989939	stopgain	C	T	0.174	p.Q962X
9615	*BAI1*	1702	8	143618420	stopgain	C	T	0.215	p.Q1215X
9615	*LAMA1*	5559	18	6986307	stopgain	G	T	0.193	p.Y1736X
9615	*CLCN6*	1286	1	11897075	missense	G	A	0.212	p.R667Q
9615	*ATP1A2*	702	1	160093100	missense	G	A	0.169	p.R92H
9615	*CD34*	1773	1	208062143	missense	A	G	0.154	p.Y286H
9615	*MTR*	1291940	1	236998982	missense	G	A	0.167	p.A442T
9615	*DNAH6*	1370	2	84816032	missense	T	A	0.210	p.L855H
9615	*IRS1*	5544	2	227661545	missense	G	A	0.216	p.P637L
9615	*CRBN*	16302	3	3195675	missense	C	T	0.103	p.R307Q
9615	*SEC13*	183352	3	10354412	missense	T	C	0.292	p.H59R
9615	*HLA-DQB2*	1300790	6	32725636	missense	C	T	0.130	p.S224N
9615	*PKHD1*	170724	6	51612720	missense	C	A	0.090	p.A3232S
9615	*COL19A1*	1858	6	70639488	missense	G	T	0.133	p.D188Y
9615	*SYNJ2*	3898	6	158492695	missense	G	A	0.150	p.V668I
9615	*PDE1C*	1191058	7	32338305	missense	G	A	0.167	p.R15W
9615	*PCLO*	33026	7	82545073	missense	C	T	0.253	p.D4077N
9615	*SLC4A2*	3040	7	150761383	missense	A	G	0.065	p.E49G
9615	*CSPP1*	24790	8	68044256	missense	G	A	0.258	p.D585N
9615	*CDH17*	4063	8	95174361	missense	T	C	0.250	p.N438D
9615	*ALAD*	31	9	116153185	missense	T	G	0.233	p.E97A
9615	*NOTCH1*	17617	9	139396812	missense	G	C	0.167	p.Q1766E
9615	*ITIH2*	2216	10	7765454	missense	A	G	0.252	p.N303S
9615	*FAM171A1*	1010924	10	15262957	missense	G	A	0.233	p.S286F
9615	*POLR3A*	7055	10	79769340	missense	T	C	0.241	p.K622E
9615	*AHNAK*	1620	11	62286593	missense	G	A	0.270	p.S5099L
9615	*PGR*	926	11	100999399	missense	C	T	0.196	p.E135K
9615	*BACE1*	138973	11	117160302	missense	C	T	0.158	p.D427N
9615	*FGF23*	20638	12	4479739	missense	G	A	0.214	p.R176W
9615	*CTDSP2*	5730	12	58217763	missense	C	T	0.191	p.R205H
9615	*CTDSP2*	5730	12	58217770	missense	G	C	0.083	p.L203V
9615	*CTDSP2*	5730	12	58220811	missense	G	T	0.112	p.L108I
9615	*CTDSP2*	5730	12	58220831	missense	C	G	0.077	p.R101T
9615	*KERA*	7035	12	91445263	missense	A	G	0.170	p.S307P
9615	*STAB2*	17564	12	104083731	missense	G	T	0.140	p.L1051F
9615	*SLC8B1*	24959	12	113758421	missense	G	A	0.143	p.P164L
9615	*FOXG1*	5249	14	29237904	missense	T	G	0.232	p.D473E
9615	*WDR89*	80666	14	64066395	missense	C	T	0.071	p.C89Y
9615	*WDR89*	80666	14	64066398	missense	G	C	0.068	p.A88G
9615	*WDR89*	80666	14	64066402	missense	A	G	0.067	p.S87P
9615	*STARD9*	20759	15	42976420	missense	T	A	0.180	p.S882T
9615	*RASGRF1*	2891	15	79320181	missense	C	T	0.172	p.S428N
9615	*RASGRF1*	2891	15	79382825	missense	G	A	0.118	p.R6W
9615	*ALPK3*	20778	15	85399839	missense	G	A	0.230	p.G826R
9615	*OR4F6*	1005326	15	102346311	missense	T	C	0.222	p.L130P
9615	*GPR139*	1002911	16	20043121	missense	T	C	0.197	p.H333R
9615	*PLCG2*	2661	16	81973601	missense	G	T	0.143	p.D1140Y

9615	*TRIM47*	33452	17	73874427	missense	C	T	0.063	p.R68H
9615	*LAMA1*	5559	18	6986306	missense	G	T	0.190	p.Q1737K
9615	*CDH7*	33646	18	63481721	missense	G	A	0.308	p.G169E
9615	*RFX1*	2918	19	14076560	missense	T	C	0.141	p.K664R
9615	*URI1*	3796	19	30496287	missense	T	A	0.182	p.D129E
9615	*ZNF71*	21216	19	57133590	missense	C	T	0.219	p.S312L
9615	*SLC17A9*	22082	20	61593978	missense	C	T	0.121	p.T167M
9615	*USP16*	6447	21	30419532	missense	A	G	0.191	p.Q634R
9615	*ETS2*	5239	21	40186798	missense	G	A	0.209	p.G273D
9618	*ANO7*	1001891	2	242142814	stopgain	C	T	0.286	p.Q318X
9618	*FIP1L1*	30917	4	54324899	stopgain	C	T	0.172	p.R527X
9618	*HIVEP1*	2114	6	12124241	stopgain	C	T	0.149	p.Q1405X
9618	*COL12A1*	4370	6	75893732	stopgain	G	A	0.093	p.R376X
9618	*MUC16*	24690	19	9085862	stopgain	G	A	0.096	p.Q1985X
9618	*ARHGAP30*	181720	1	161018818	missense	G	A	0.107	p.P665S
9618	*SNAP47*	53052	1	227947179	missense	G	T	0.078	p.W372C
9618	*GRB14*	4490	2	165378509	missense	G	T	0.190	p.S266Y
9618	*FSIP2*	173651	2	186654495	missense	G	A	0.172	p.D967N
9618	*HIBCH*	14362	2	191073636	missense	C	G	0.108	p.G339R
9618	*MOGAT1*	58165	2	223554125	missense	T	C	0.154	p.Y139H
9618	*IRS1*	5544	2	227660523	missense	G	A	0.114	p.P978S
9618	*GIGYF2*	15575	2	233712247	missense	C	A	0.172	p.P1217Q
9618	*IL17RD*	17563	3	57140150	missense	C	T	0.108	p.A195T
9618	*NLGN1*	14932	3	173998281	missense	C	A	0.123	p.Q554K
9618	*GPRIN1*	52899	5	176026168	missense	C	T	0.148	p.S223N
9618	*OR2V1*	1258283	5	180551620	missense	T	A	0.286	p.I229L
9618	*CD2AP*	12120	6	47512380	missense	A	G	0.220	p.I120V
9618	*DST*	1723	6	56482074	missense	T	A	0.167	p.K2064M
9618	*HGF*	1010932	7	81334821	missense	G	T	0.267	p.A627E
9618	*KEL*	420	7	142638479	missense	G	A	0.138	p.P687S
9618	*ZNF425*	1001661	7	148802431	missense	G	A	0.169	p.P178S
9618	*FOXB2*	1013735	9	79635099	missense	G	A	0.286	p.V177I
9618	*SKIDA1*	207371	10	21805466	missense	C	T	0.108	p.G429E
9618	*PSD*	2779	10	104165255	missense	G	A	0.059	p.A346V
9618	*NRAP*	198060	10	115383378	missense	G	T	0.077	p.S789R
9618	*SHANK2*	133266	11	70332367	missense	G	C	0.121	p.S756W
9618	*TEAD4*	201441	12	3120221	missense	G	A	0.167	p.R93H
9618	*SOAT2*	3578	12	53509227	missense	C	T	0.186	p.A166V
9618	*CTDSP2*	5730	12	58217763	missense	C	T	0.141	p.R205H
9618	*CTDSP2*	5730	12	58220801	missense	G	T	0.108	p.T111N
9618	*CTDSP2*	5730	12	58220811	missense	G	T	0.098	p.L108I
9618	*CTDSP2*	5730	12	58220831	missense	C	A	0.085	p.R101M
9618	*PABPC3*	30979	13	25670691	missense	G	T	0.053	p.V119F
9618	*WDR89*	80666	14	64066367	missense	A	T	0.056	p.D98E
9618	*WDR89*	80666	14	64066395	missense	C	T	0.062	p.C89Y
9618	*WDR89*	80666	14	64066398	missense	G	C	0.063	p.A88G
9618	*WDR89*	80666	14	64066402	missense	A	G	0.066	p.S87P
9618	*DENND4A*	5848	15	66023984	missense	G	C	0.175	p.L388V
9618	*LPCAT2*	17839	16	55616860	missense	A	C	0.098	p.E495D
9618	*COG8*	32382	16	69364935	missense	T	C	0.323	p.Q549R
9618	*COG8*	32382	16	69364936	missense	G	T	0.333	p.Q549K
9618	*TXNDC2*	32243	18	9887856	missense	G	C	0.345	p.E393D
9618	*ZNF516*	14643	18	74091345	missense	T	A	0.169	p.R909W
9618	*BTBD2*	17797	19	1993050	missense	C	G	0.250	p.R218P
9618	*CEACAM1*	1712	19	43025485	missense	T	G	0.054	p.T298P
9618	*SYT3*	32298	19	51132693	missense	C	T	0.200	p.R380H
9618	*FPR1*	2029	19	52249509	missense	C	T	0.163	p.A247T
9618	*LILRA2*	6866	19	55086368	missense	T	G	0.257	p.W175G
9619	*ZSCAN23*	1012455	6	28403264	stopgain	G	A	0.200	p.R177X
9619	*C8orf34*	52958	8	69243409	stopgain	T	A	0.231	p.C54X
9619	*ZNF471*	20813	19	57037019	stopgain	C	A	0.233	p.S528X
9619	*PTPRU*	133178	1	29602196	missense	G	C	0.279	p.V461L
9619	*SMAP2*	22733	1	40879856	missense	T	A	0.178	p.L172Q
9619	*WLS*	24911	1	68659655	missense	T	C	0.138	p.K121R
9619	*KIAA0040*	14656	1	175129933	missense	T	C	0.182	p.K73E
9619	*IRF2BP2*	182972	1	234744394	missense	T	C	0.207	p.S283G
9619	*OR2T10*	1004693	1	248756263	missense	C	A	0.263	p.E269D
9619	*ABCB11*	3742	2	169850282	missense	A	G	0.244	p.L241P
9619	*FSIP2*	173651	2	186654216	missense	G	A	0.235	p.D874N
9619	*KIF1A*	4321	2	241658487	missense	C	T	0.093	p.R1717Q
9619	*KIF1A*	4321	2	241682361	missense	G	A	0.051	p.P1209S
9619	*KLF15*	14079	3	126071527	missense	A	G	0.265	p.L80P
9619	*PIK3R4*	14602	3	130424431	missense	T	C	0.250	p.E969G
9619	*WDFY3*	14991	4	85645657	missense	C	T	0.167	p.A2455T
9619	*NR3C2*	1166104	4	149073762	missense	A	C	0.200	p.F790V
9619	*ELL2*	12081	5	95234393	missense	G	A	0.256	p.S359F
9619	*ZNF608*	20747	5	124080258	missense	C	T	0.288	p.R142H
9619	*GABRA1*	1127648	5	161324145	missense	A	C	0.143	p.K363T
9619	*CANX*	1746	5	179132839	missense	C	T	0.299	p.P53S
9619	*TBC1D7*	16495	6	13321292	missense	C	G	0.215	p.V77L
9619	*FAM8A1*	16255	6	17601040	missense	G	A	0.119	p.G134S

9619	*FAM8A1*	16255	6	17601041	missense	G	T	0.118	p.G134V
9619	*FAM8A1*	16255	6	17601044	missense	T	C	0.119	p.L135P
9619	*FAM8A1*	16255	6	17601058	missense	G	A	0.106	p.A140T
9619	*FAM8A1*	16255	6	17601086	missense	A	G	0.115	p.Q149R
9619	*FAM8A1*	16255	6	17601098	missense	C	T	0.066	p.S153L
9619	*HLA-DRB1*	2124	6	32551894	missense	T	C	0.196	p.Q121R
9619	*HLA-DQB1*	2123	6	32632769	missense	T	C	0.100	p.Y62C
9619	*HLA-DQA2*	20056	6	32713061	missense	C	A	0.064	p.Q70K
9619	*DSE*	13352	6	116752270	missense	T	C	0.259	p.L275P
9619	*GPRC6A*	148963	6	117128013	missense	A	C	0.061	p.D285E
9619	*OLIG3*	175747	6	137814785	missense	C	T	0.059	p.A175T
9619	*ELFN1*	1128636	7	1785134	missense	C	T	0.173	p.T301M
9619	*OTUD6B*	16023	8	92083488	missense	C	A	0.060	p.Q99K
9619	*ABCA2*	212533	9	139912294	missense	G	C	0.346	p.S749C
9619	*MLLT10*	4641	10	21903771	missense	C	T	0.063	p.A174V
9619	*HK1*	33500	10	71146097	missense	A	G	0.229	p.T608A
9619	*SLC17A6*	20346	11	22396364	missense	A	G	0.186	p.I369V
9619	*MUC15*	145650	11	26582672	missense	T	G	0.226	p.E342D
9619	*OR4A15*	1005275	11	55136345	missense	T	G	0.241	p.L329R
9619	*C2CD3*	15531	11	73789391	missense	C	T	0.052	p.V1458I
9619	*GDPD4*	182833	11	76969472	missense	G	C	0.156	p.L275V
9619	*KRT3*	57088	12	53189427	missense	C	G	0.222	p.A134P
9619	*CTDSP2*	5730	12	58217698	missense	G	T	0.068	p.P227T
9619	*CTDSP2*	5730	12	58217763	missense	C	T	0.252	p.R205H
9619	*CTDSP2*	5730	12	58220801	missense	G	T	0.074	p.T111N
9619	*CTDSP2*	5730	12	58220811	missense	G	T	0.108	p.L108I
9619	*CTDSP2*	5730	12	58220831	missense	C	G	0.074	p.R101T
9619	*TDG*	3211	12	104379429	missense	A	G	0.139	p.Y338C
9619	*CUX2*	15267	12	111731337	missense	A	C	0.265	p.E175A
9619	*ANAPC5*	16237	12	121790092	missense	C	T	0.227	p.V18I
9619	*FAM155A*	1080396	13	107823123	missense	T	C	0.185	p.T367A
9619	*HOMEZ*	20834	14	23746259	missense	G	C	0.225	p.Q60E
9619	*PPP4R4*	58237	14	94731783	missense	G	A	0.080	p.G753R
9619	*BMF*	33503	15	40396512	missense	C	T	0.060	p.A108T
9619	*FBXL16*	153350	16	744652	missense	G	A	0.075	p.R425C
9619	*SH2B1*	15503	16	28877657	missense	C	T	0.106	p.A81V
9619	*CYLD*	15247	16	50815165	missense	G	C	0.114	p.E509D
9619	*TEX14*	198393	17	56642297	missense	T	C	0.184	p.S1373G
9619	*FHOD3*	25135	18	34182640	missense	T	G	0.345	p.V241G
9619	*MED16*	5481	19	868464	missense	C	A	0.253	p.R812I
9619	*ZNF625*	145233	19	12256410	missense	G	A	0.277	p.P274L
9619	*LEUTX*	1143832	19	40276562	missense	C	A	0.242	p.S98R
9619	*VRK3*	16440	19	50496118	missense	T	A	0.267	p.Q321L
9619	*MACROD2*	80676	20	13982967	missense	G	A	0.282	p.R27H
9619	*TPTE*	199261	21	10921938	missense	G	A	0.108	p.A362V
9619	*DDX53*	182699	X	23019279	missense	A	G	0.200	p.S369G
9619	*FAM104B*	138362	X	55172686	missense	C	T	0.143	p.S60N
9619	*FAM104B*	138362	X	55172689	missense	G	T	0.150	p.A59E
9619	*TCEAL2*	80390	X	101382112	missense	G	A	0.174	p.G104R
9619	*TCEAL2*	80390	X	101382123	missense	C	G	0.239	p.D107E
9619	*TCEAL2*	80390	X	101382148	missense	G	A	0.128	p.G116R
9619	*RBMXL3*	1145346	X	114425181	missense	A	G	0.082	p.R393G
9619	*L1CAM*	24003	X	153135542	missense	A	C	0.175	p.S320R
9627	*CACHD1*	20925	1	65095158	missense	G	A	0.063	p.R162H
9627	*KCNN3*	2249	1	154842244	missense	A	T	0.067	p.L66H
9627	*INSRR*	14215	1	156821904	missense	C	A	0.066	p.Q239H
9627	*ZNF717*	1290209	3	75788230	missense	C	T	0.080	p.V132I
9627	*FAM8A1*	16255	6	17601058	missense	G	A	0.096	p.A140T
9627	*CD109*	133493	6	74472138	missense	G	A	0.182	p.V264I
9627	*SCAF11*	4719	12	46320488	missense	T	C	0.056	p.E999G
9627	*CTDSP2*	5730	12	58217763	missense	C	T	0.176	p.R205H
9627	*CTDSP2*	5730	12	58220801	missense	G	T	0.073	p.T111N
9627	*CTDSP2*	5730	12	58220811	missense	G	T	0.118	p.L108I
9627	*ZFYVE26*	15346	14	68264965	missense	T	C	0.058	p.T672A
9627	*APOBR*	18690	16	28507445	missense	G	C	0.175	p.E361D
9627	*APOBR*	18690	16	28507452	missense	G	T	0.171	p.G364W
9627	*BBS2*	31885	16	56533698	missense	C	T	0.063	p.A507T
9627	*HLF*	2126	17	53392602	missense	G	A	0.105	p.A156T
9627	*ST6GALNAC1*	18414	17	74622824	missense	C	A	0.089	p.R275L
9627	*DDX39A*	5804	19	14521876	missense	G	A	0.050	p.R180W
9627	*ZNF569*	152484	19	37904580	missense	G	T	0.057	p.A327E
9627	*ARHGEF1*	199002	19	42396797	missense	G	A	0.087	p.G179D
9627	*FAM104B*	1166703	X	55172521	missense	G	T	0.075	p.T114K
9627	*FAM104B*	138362	X	55172686	missense	C	T	0.116	p.S60N
9627	*FAM104B*	138362	X	55172689	missense	G	T	0.090	p.A59E
9628	*RELN*	173054	7	103557564	stopgain	G	A	0.075	p.Q99X
9628	*FOXD2*	4474	1	47905258	missense	C	T	0.089	p.A484V
9628	*GRHL1*	198182	2	10132228	missense	T	C	0.287	p.L472P
9628	*STRN*	3162	2	37129752	missense	C	T	0.069	p.E212K
9628	*LRP1B*	18557	2	141739826	missense	G	C	0.088	p.D930E
9628	*UPK1B*	6952	3	118905592	missense	G	A	0.052	p.A2T

9628	*SLIT2*	4787	4	20550747	missense	T	G	0.275	p.L829V
9628	*UNC5C*	3728	4	96163682	missense	G	A	0.202	p.R336C
9628	*SLIT3*	3062	5	168244436	missense	C	A	0.234	p.C221F
9628	*FAM8A1*	16255	6	17601098	missense	C	T	0.083	p.S153L
9628	*MUC22*	1198815	6	30996839	missense	G	A	0.082	p.E1211K
9628	*HLA-DQB1*	2123	6	32632769	missense	T	C	0.105	p.Y62C
9628	*HLA-DQA2*	20056	6	32713061	missense	C	A	0.107	p.Q70K
9628	*SDK1*	152744	7	4172007	missense	G	A	0.250	p.V1394M
9628	*ABCA13*	152701	7	48315342	missense	A	G	0.063	p.M2027V
9628	*GATAD1*	21167	7	92077048	missense	C	T	0.227	p.P2L
9628	*POT1*	15450	7	124465335	missense	T	C	0.244	p.D588G
9628	*RNF32*	30936	7	156437439	missense	C	A	0.129	p.P88T
9628	*CSMD1*	33225	8	3253897	missense	C	A	0.189	p.Q804H
9628	*PRUNE2*	15225	9	79325973	missense	G	A	0.256	p.P406L
9628	*TMEM38B*	18112	9	108467901	missense	A	T	0.216	p.N46Y
9628	*IFIT5*	12420	10	91178194	missense	G	A	0.286	p.R413H
9628	*CTBP2*	22802	10	126692019	missense	G	A	0.222	p.P34L
9628	*MRGPRE*	1039165	11	3250020	missense	G	A	0.161	p.P4S
9628	*CTR9*	14633	11	10778364	missense	C	T	0.063	p.R191C
9628	*OR5D16*	1005496	11	55606357	missense	A	G	0.224	p.N44D
9628	*FAT3*	1008781	11	92616485	missense	A	C	0.065	p.N4288T
9628	*LRP6*	2336	12	12311792	missense	G	A	0.250	p.S921F
9628	*KRT76*	15848	12	53170841	missense	C	T	0.056	p.G79R
9628	*CTDSP2*	5730	12	58217698	missense	G	T	0.069	p.P227T
9628	*CTDSP2*	5730	12	58217770	missense	G	C	0.056	p.L203V
9628	*CTDSP2*	5730	12	58220811	missense	G	T	0.112	p.L108I
9628	*CTDSP2*	5730	12	58220819	missense	A	G	0.121	p.V105A
9628	*CTDSP2*	5730	12	58220831	missense	C	G	0.072	p.R101T
9628	*CTDSP2*	5730	12	58240188	missense	G	A	0.104	p.R11W
9628	*TMEM132D*	133448	12	129559442	missense	T	C	0.263	p.T760A
9628	*DNAAF1*	178452	16	84182738	missense	G	A	0.192	p.R84Q
9628	*C17orf49*	174893	17	6919879	missense	A	G	0.228	p.E95G
9628	*GFAP*	2055	17	42989131	missense	G	A	0.258	p.A272V
9628	*BPTF*	182641	17	65908784	missense	T	C	0.254	p.V1721A
9628	*RPTOR*	20761	17	78933959	missense	G	T	0.291	p.D1187Y
9628	*CLPP*	6012	19	6361895	missense	G	T	0.083	p.A72S
9628	*COL6A1*	1848	21	47406559	missense	G	A	0.167	p.G183D
9628	*CPT1B*	152246	22	51015768	missense	C	T	0.278	p.R89K
9628	*HEPH*	138737	X	65480080	missense	C	A	0.180	p.L1113I
9628	*AR*	1011645	X	66941771	missense	C	A	0.182	p.F273L
9628	*GPR112*	153834	X	135431614	missense	G	T	0.229	p.G1917W
9628	*GPR112*	153834	X	135432373	missense	A	G	0.181	p.I2170V
9629	*PBRM1*	18313	3	52598142	stopgain	G	A	0.121	p.Q1242X
9629	*ZBTB20*	15642	3	114070039	stopgain	G	A	0.109	p.Q223X
9629	*NUP153*	5124	6	17661928	stopgain	G	A	0.051	p.R451X
9629	*ATG2B*	18036	14	96771988	stopgain	C	T	0.053	p.W1557X
9629	*RPS6KA1*	2953	1	26887329	missense	A	G	0.250	p.E443G
9629	*ACKR1*	2036	1	159175522	missense	T	A	0.111	p.V98D
9629	*ITLN1*	17625	1	160851089	missense	T	A	0.073	p.Y140F
9629	*C4BPA*	715	1	207287489	missense	G	A	0.075	p.D63N
9629	*USH2A*	206933	1	215901673	missense	G	A	0.211	p.A3922V
9629	*MAP10*	19090	1	232942128	missense	A	C	0.163	p.Q453H
9629	*CCDC142*	32779	2	74702216	missense	T	A	0.101	p.Q601L
9629	*LRP1B*	18557	2	141777603	missense	G	A	0.125	p.H620Y
9629	*FMNL2*	52905	2	153484907	missense	C	T	0.091	p.R754W
9629	*GIGYF2*	15575	2	233704620	missense	C	T	0.087	p.T943M
9629	*LHFPL4*	198560	3	9594093	missense	C	T	0.119	p.A91T
9629	*NCKIPSD*	184231	3	48716855	missense	C	T	0.064	p.A502T
9629	*ZNF717*	1290209	3	75788230	missense	C	T	0.202	p.V132I
9629	*OR5K4*	1005517	3	98073598	missense	A	T	0.167	p.I301F
9629	*TIMMDC1*	16589	3	119219609	missense	G	A	0.062	p.G88S
9629	*ENAM*	31889	4	71497439	missense	A	G	0.098	p.N36S
9629	*TET2*	1127208	4	106180899	missense	T	A	0.139	p.F1309L
9629	*ARHGEF28*	1244364	5	73207259	missense	G	C	0.147	p.V1603L
9629	*VWA7*	25258	6	31743196	missense	C	T	0.118	p.S175N
9629	*HLA-DQA2*	20056	6	32713061	missense	C	A	0.149	p.Q70K
9629	*ZNF292*	15021	6	87969625	missense	A	C	0.118	p.K2093T
9629	*NDUFA4*	2489	7	10979669	missense	T	G	0.082	p.I6L
9629	*MPLKIP*	138701	7	40173925	missense	G	A	0.074	p.P81L
9629	*PLOD3*	1084	7	100853439	missense	A	G	0.065	p.W540R
9629	*CHPF2*	19015	7	150932286	missense	G	T	0.156	p.G131V
9629	*C8orf22*	1256598	8	49986834	missense	T	G	0.085	p.F59V
9629	*KANK1*	153186	9	744533	missense	A	G	0.072	p.K1314E
9629	*SPATA6L*	1039395	9	4661921	missense	T	C	0.200	p.Q52R
9629	*DOCK1*	1380	10	128840973	missense	G	T	0.110	p.A678S
9629	*OR51B2*	33180	11	5344887	missense	A	T	0.127	p.F214Y
9629	*CTR9*	14633	11	10772991	missense	G	A	0.118	p.R11Q
9629	*TPCN2*	139075	11	68848872	missense	C	T	0.078	p.P532S
9629	*USP35*	20798	11	77920646	missense	G	A	0.051	p.C582Y
9629	*TENM4*	1098816	11	78380720	missense	G	A	0.255	p.H2224Y
9629	*TSPAN9*	6675	12	3389554	missense	G	A	0.143	p.E113K

9629	*PDZRN4*	13377	12	41900447	missense	G	A	0.149	p.A345T
9629	*CTDSP2*	5730	12	58217698	missense	G	T	0.059	p.P227T
9629	*CTDSP2*	5730	12	58220811	missense	G	T	0.082	p.L108I
9629	*CTDSP2*	5730	12	58220819	missense	A	G	0.146	p.V105A
9629	*CTDSP2*	5730	12	58220831	missense	C	G	0.087	p.R101T
9629	*CTDSP2*	5730	12	58240188	missense	G	A	0.160	p.R11W
9629	*MYH6*	2471	14	23857002	missense	A	T	0.174	p.L1497Q
9629	*WDR89*	80666	14	64066352	missense	T	A	0.083	p.R103S
9629	*WDR89*	80666	14	64066367	missense	A	T	0.100	p.D98E
9629	*WDR89*	80666	14	64066395	missense	C	T	0.139	p.C89Y
9629	*WDR89*	80666	14	64066398	missense	G	C	0.153	p.A88G
9629	*WDR89*	80666	14	64066402	missense	A	G	0.137	p.S87P
9629	*IRF2BPL*	24496	14	77493537	missense	G	A	0.183	p.P200L
9629	*C16orf45*	33201	16	15677089	missense	C	A	0.089	p.P166T
9629	*ADAMTS18*	199355	16	77356283	missense	T	C	0.107	p.T705A
9629	*ZNF469*	1127464	16	88503683	missense	G	A	0.225	p.E3241K
9629	*DNAH2*	20877	17	7682634	missense	T	C	0.093	p.I1872T
9629	*KRTAP1-4*	1257305	17	39186320	missense	C	G	0.122	p.C4S
9629	*TMEM104*	17728	17	72791154	missense	G	A	0.202	p.G144E
9629	*TMC8*	152468	17	76137024	missense	C	T	0.207	p.P671L
9629	*SLC7A9*	14270	19	33359437	missense	C	T	0.202	p.G2R
9629	*ZSCAN5B*	1080456	19	56704307	missense	T	C	0.052	p.N39D
9629	*RALGAPA2*	20343	20	20475761	missense	C	A	0.066	p.E1789D
9629	*NPEPL1*	24663	20	57276087	missense	G	A	0.055	p.G184D
9629	*NCAM2*	4540	21	22710835	missense	C	G	0.163	p.T342R
9631	*LIPT1*	145199	2	99779506	stopgain	G	A	0.227	p.W362X
9631	*COG3*	31431	13	46054378	stopgain	A	T	0.069	p.K168X
9631	*KCNJ12*	21012	17	21319092	stopgain	C	A	0.257	p.Y146X
9631	*RNF207*	207396	1	6266719	missense	C	A	0.053	p.H42N
9631	*KIAA1324*	20775	1	109742751	missense	C	G	0.263	p.T900S
9631	*FMO3*	6894	1	171086491	missense	G	A	0.272	p.G503E
9631	*COBLL1*	14900	2	165548810	missense	T	C	0.224	p.Q1179R
9631	*ROBO1*	133631	3	78680389	missense	G	T	0.281	p.A1183E
9631	*GUCY1A3*	1256449	4	156634624	missense	C	G	0.209	p.I487M
9631	*HLA-DQB2*	1300790	6	32725636	missense	C	T	0.145	p.S224N
9631	*FAM20C*	20223	7	298646	missense	C	A	0.089	p.L494M
9631	*MUC17*	1040105	7	100680057	missense	C	G	0.052	p.T1787S
9631	*KMT2C*	170606	7	152027726	missense	C	T	0.082	p.A117T
9631	*KIAA1429*	183009	8	95531549	missense	T	C	0.056	p.E726G
9631	*TRAPPC9*	31466	8	141460890	missense	T	C	0.080	p.R293G
9631	*SWI5*	1040011	9	131038437	missense	G	A	0.231	p.G5S
9631	*ANK3*	20987	10	61894096	missense	A	G	0.071	p.L926S
9631	*HPSE2*	21828	10	100503687	missense	T	G	0.250	p.K246T
9631	*CTBP2*	22802	10	126686646	missense	C	G	0.135	p.W151S
9631	*CTBP2*	22802	10	126686649	missense	G	C	0.128	p.T150R
9631	*CTBP2*	22802	10	126686656	missense	T	C	0.132	p.R148G
9631	*CTBP2*	22802	10	126686680	missense	A	C	0.116	p.C140G
9631	*SOX5*	178010	12	23687439	missense	G	T	0.281	p.P656H
9631	*CTDSP2*	5730	12	58217763	missense	C	T	0.197	p.R205H
9631	*CTDSP2*	5730	12	58217770	missense	G	C	0.059	p.L203V
9631	*CTDSP2*	5730	12	58220801	missense	G	T	0.057	p.T111N
9631	*CTDSP2*	5730	12	58220819	missense	A	G	0.070	p.V105A
9631	*CTDSP2*	5730	12	58240184	missense	C	A	0.071	p.R12M
9631	*CTDSP2*	5730	12	58240188	missense	G	A	0.123	p.R11W
9631	*NAV3*	14903	12	78444893	missense	G	C	0.267	p.G828R
9631	*HSP90B1*	3299	12	104341190	missense	A	T	0.077	p.E788D
9631	*MAB21L1*	5584	13	36050061	missense	G	C	0.248	p.T72S
9631	*UGGT2*	20121	13	96547442	missense	G	T	0.182	p.N917K
9631	*FBN1*	138	15	48762896	missense	G	T	0.277	p.P1465H
9631	*FAM174B*	207446	15	93198688	missense	A	C	0.128	p.S68A
9631	*TUBB3*	6086	16	90001641	missense	C	T	0.080	p.P261L
9631	*RAI1*	30665	17	17697096	missense	G	C	0.054	p.Q278H
9631	*CDC27*	1293091	17	45229221	missense	C	T	0.200	p.E341K
9631	*CDC27*	1293091	17	45229228	missense	A	C	0.172	p.N338K
9631	*CDC27*	1293091	17	45229234	missense	A	C	0.147	p.S336R
9631	*FAM83E*	17708	19	49107038	missense	G	T	0.074	p.Q297K
9631	*ZNF577*	32679	19	52376946	missense	C	A	0.179	p.Q99H
9631	*PEG3*	6210	19	57327001	missense	C	A	0.292	p.A937S
9631	*MYL9*	181526	20	35177615	missense	G	A	0.051	p.R161H
9631	*TAF4*	3185	20	60573244	missense	C	A	0.062	p.R973L
9631	GNAQ	2072	9	80537112	missense	T	A	0.227	p.T96S
9632	*CCDC88A*	18084	2	55573427	stopgain	G	A	0.051	p.R309X
9632	*CCDC168*	1146197	13	103395635	stopgain	A	T	0.223	p.L2471X
9632	*DHX34*	14681	19	47885359	stopgain	C	T	0.215	p.Q1141X
9632	*LRRC7*	20794	1	70504563	missense	C	T	0.275	p.P981L
9632	*DDX20*	7204	1	112309408	missense	T	G	0.164	p.Y788D
9632	*POU2F1*	2697	1	167353121	missense	C	T	0.050	p.T249M
9632	*PAPPA2*	21936	1	176564431	missense	G	T	0.146	p.S564I
9632	*HMCN1*	31935	1	186135419	missense	T	A	0.163	p.D5141E
9632	*BRINP3*	199051	1	190067855	missense	G	A	0.163	p.R532W
9632	*2-Mar*	17898	1	220935037	missense	A	G	0.125	p.N162D

9632	*RBMS1*	16836	2	161141342	missense	T	A	0.247	p.M282L
9632	*SCN1A*	6920	2	166848587	missense	A	C	0.246	p.I1733S
9632	*ANKMY1*	16552	2	241451318	missense	G	T	0.269	p.T749K
9632	*SUMF1*	182760	3	4459758	missense	C	T	0.179	p.A221T
9632	*ROBO2*	2942	3	77147256	missense	C	A	0.195	p.N51K
9632	*EPHA3*	5233	3	89478256	missense	G	A	0.087	p.S692N
9632	*ZNF718*	182524	4	59387	missense	C	G	0.127	p.P23R
9632	*DSPP*	14208	4	88537243	missense	C	A	0.163	p.D1143E
9632	*MAB21L2*	6439	4	151504662	missense	C	T	0.209	p.R161C
9632	*FAM149A*	15398	4	187075716	missense	C	T	0.053	p.R103C
9632	*CCNO*	21147	5	54527321	missense	G	A	0.239	p.A312V
9632	*MAST4*	15183	5	66462811	missense	G	T	0.274	p.D2602Y
9632	*IL5*	879	5	131879143	missense	G	C	0.208	p.L10V
9632	*HMGXB3*	14983	5	149431670	missense	C	T	0.211	p.T1265I
9632	*FAM8A1*	16255	6	17601058	missense	G	A	0.089	p.A140T
9632	*MUC22*	1198815	6	30995478	missense	C	A	0.109	p.T757K
9632	*HLA-DQA2*	20056	6	32713076	missense	A	C	0.092	p.S75R
9632	*HLA-DQA2*	20056	6	32713080	missense	A	G	0.094	p.K76R
9632	*HLA-DQA2*	20056	6	32713090	missense	T	A	0.088	p.S79R
9632	*ITPR3*	2224	6	33651107	missense	G	A	0.050	p.R1574Q
9632	*RIMS1*	14989	6	73100335	missense	A	G	0.151	p.R1468G
9632	*ZNF804B*	181646	7	88965990	missense	A	G	0.235	p.I1232V
9632	*LOC93432*	1293626	7	141920342	missense	G	A	0.073	p.A2011T
9632	*LOC93432*	1293626	7	141920414	missense	G	A	0.173	p.G2035S
9632	*ESYT2*	20728	7	158552795	missense	C	A	0.059	p.R474I
9632	*DLC1*	182643	8	12957705	missense	A	G	0.145	p.V714A
9632	*MFSD3*	138431	8	145735371	missense	G	A	0.063	p.A219T
9632	*SLC46A2*	33051	9	115652523	missense	C	T	0.127	p.G147S
9632	*TTC16*	144965	9	130489280	missense	G	A	0.074	p.G486D
9632	*ABCA2*	212533	9	139907001	missense	G	A	0.258	p.P1738L
9632	*OR13A1*	1004297	10	45799171	missense	C	T	0.262	p.A234T
9632	*SH3PXD2A*	14631	10	105362982	missense	A	T	0.191	p.S637T
9632	*FOLH1*	4476	11	49170265	missense	C	G	0.250	p.M648I
9632	*TBX10*	5995	11	67400493	missense	C	A	0.188	p.A211S
9632	*USP35*	20798	11	77921717	missense	G	T	0.088	p.R939I
9632	*SCNN1A*	1159576	12	6471352	missense	G	A	0.057	p.A270V
9632	*ATF7IP*	181352	12	14578026	missense	G	T	0.100	p.D401Y
9632	*KANSL2*	17822	12	49072823	missense	G	C	0.263	p.L181V
9632	*HSD17B6*	3725	12	57180993	missense	C	T	0.100	p.S274L
9632	*CTDSP2*	5730	12	58217698	missense	G	T	0.067	p.P227T
9632	*CTDSP2*	5730	12	58217763	missense	C	T	0.228	p.R205H
9632	*CTDSP2*	5730	12	58220801	missense	G	T	0.111	p.T111N
9632	*CTDSP2*	5730	12	58240188	missense	G	A	0.103	p.R11W
9632	*NAV3*	14903	12	78593302	missense	G	A	0.267	p.V2236I
9632	*PRDM4*	12406	12	108145611	missense	G	C	0.198	p.S236C
9632	*ANKLE2*	15114	12	133306731	missense	T	C	0.108	p.R673G
9632	*SLC39A2*	14579	14	21469691	missense	G	T	0.069	p.V295L
9632	*MYH7*	257	14	23898542	missense	C	A	0.136	p.A385S
9632	*WDR89*	80666	14	64066395	missense	C	T	0.082	p.C89Y
9632	*WDR89*	80666	14	64066398	missense	G	C	0.085	p.A88G
9632	*WDR89*	80666	14	64066402	missense	A	G	0.091	p.S87P
9632	*PAPLN*	173462	14	73732130	missense	C	T	0.192	p.R1033W
9632	*NUTM1*	175741	15	34640318	missense	C	A	0.163	p.F55L
9632	*MGA*	1164273	15	42021452	missense	G	T	0.164	p.D1250Y
9632	*STARD9*	20759	15	42978986	missense	C	T	0.241	p.S1737F
9632	*LDHAL6B*	33195	15	59500155	missense	G	A	0.102	p.S339N
9632	*ZFHX3*	6885	16	72829881	missense	G	T	0.174	p.P2234T
9632	*OSGIN1*	182981	16	83999068	missense	C	T	0.218	p.A297V
9632	*KIF1C*	6612	17	4925427	missense	C	G	0.127	p.S684C
9632	*RAI1*	30665	17	17697096	missense	G	C	0.085	p.Q278H
9632	*SLFN5*	144975	17	33585731	missense	G	A	0.237	p.D8N
9632	*MPP2*	5374	17	41960309	missense	G	T	0.160	p.R139S
9632	*CDC27*	1293091	17	45229221	missense	C	T	0.129	p.E341K
9632	*CDC27*	1293091	17	45229228	missense	A	C	0.114	p.N338K
9632	*CDC27*	1293091	17	45229234	missense	A	C	0.105	p.S336R
9633	*DLC1*	182643	8	12943321	stoploss	C	A	0.206	p.X1529L
9633	*NDST4*	22569	4	115767059	stopgain	G	A	0.213	p.R679X
9633	*SVIL*	21738	10	29840013	stopgain	G	A	0.267	p.R114X
9633	*WDR89*	80666	14	64066363	stopgain	G	A	0.055	p.R100X
9633	*DUOX2*	14080	15	45387752	stopgain	C	T	0.067	p.W1374X
9633	*BEND5*	24603	1	49224707	missense	G	T	0.163	p.L204M
9633	*VWA3B*	144992	2	98736192	missense	C	A	0.212	p.Q170K
9633	*FSIP2*	173651	2	186696577	missense	G	T	0.131	p.G6941V
9633	*NR1D2*	5126	3	24003867	missense	G	A	0.182	p.S306N
9633	*ADAMTS9*	182920	3	64554177	missense	C	T	0.172	p.R1464Q
9633	*MBNL1*	207297	3	152132763	missense	C	G	0.055	p.H70D
9633	*DSPP*	14208	4	88537574	missense	G	A	0.092	p.D1254N
9633	*ZSWIM6*	20928	5	60831400	missense	C	T	0.145	p.P779S
9633	*HDAC3*	3883	5	141005272	missense	C	T	0.225	p.E347K
9633	*BTNL3*	197975	5	180419906	missense	C	T	0.145	p.A48V
9633	*FAM8A1*	16255	6	17601035	missense	A	G	0.165	p.H132R

9633	*FAM8A1*	16255	6	17601040	missense	G	A	0.157	p.G134S
9633	*FAM8A1*	16255	6	17601041	missense	G	T	0.154	p.G134V
9633	*FAM8A1*	16255	6	17601044	missense	T	C	0.165	p.L135P
9633	*FAM8A1*	16255	6	17601058	missense	G	A	0.175	p.A140T
9633	*FAM8A1*	16255	6	17601086	missense	A	G	0.131	p.Q149R
9633	*FAM8A1*	16255	6	17601098	missense	C	T	0.118	p.S153L
9633	*PDGFRL*	6207	8	17447007	missense	G	A	0.138	p.R29H
9633	*LOXL2*	2318	8	23186032	missense	C	T	0.208	p.R338H
9633	*FOXB2*	1013735	9	79635495	missense	G	C	0.159	p.V309L
9633	*HABP4*	14282	9	99227751	missense	A	C	0.163	p.E215D
9633	*CNTRL*	7018	9	123919842	missense	C	T	0.123	p.T1487I
9633	*STXBP1*	3165	9	130444690	missense	G	A	0.174	p.R518H
9633	*BBOX1*	3986	11	27141323	missense	T	C	0.053	p.F256S
9633	*CTDSP2*	5730	12	58217698	missense	G	T	0.103	p.P227T
9633	*CTDSP2*	5730	12	58217770	missense	G	C	0.076	p.L203V
9633	*CTDSP2*	5730	12	58220811	missense	G	T	0.067	p.L108I
9633	*SNRNP35*	180699	12	123950115	missense	G	A	0.204	p.E15K
9633	*ATP11A*	32189	13	113459275	missense	C	A	0.140	p.T56K
9633	*WDR89*	80666	14	64066352	missense	T	A	0.052	p.R103S
9633	*WDR89*	80666	14	64066367	missense	A	T	0.057	p.D98E
9633	*SETD3*	32233	14	99870629	missense	C	G	0.262	p.L370F
9633	*ALPK3*	20778	15	85383493	missense	G	A	0.231	p.R530Q
9633	*MESP2*	1039958	15	90319965	missense	G	A	0.141	p.R126H
9633	*LRRK1*	24652	15	101549076	missense	C	T	0.178	p.P266L
9633	*PIGQ*	148920	16	633184	missense	G	C	0.163	p.L611F
9633	*CORO7*	24535	16	4457570	missense	C	T	0.098	p.G140D
9633	*STAT3*	213662	17	40474420	missense	C	A	0.239	p.D661Y
9633	*KIR2DL4*	2255	19	55317489	missense	C	A	0.052	p.Q149K
9633	*KIR2DL4*	2255	19	55317490	missense	A	G	0.060	p.Q149R
9633	*KIR2DL4*	2255	19	55317529	missense	A	G	0.072	p.E162G
9633	*KIR3DL1*	13289	19	55329789	missense	C	G	0.077	p.F30L
9633	*KIR3DL2*	6737	19	55377280	missense	G	A	0.226	p.V341I
9633	*ZNF584*	173548	19	58929123	missense	G	A	0.053	p.R413K
9633	*DEFB132*	207469	20	239803	missense	G	A	0.245	p.M48I
9633	*MPST*	21126	22	37425325	missense	C	A	0.204	p.L222M
9633	*FAM104B*	1166703	X	55172521	missense	G	T	0.082	p.T114K
9633	*FAM104B*	138362	X	55172686	missense	C	T	0.077	p.S60N
9635	*WDR89*	80666	14	64066363	stopgain	G	A	0.111	p.R100X
9635	*FLG*	2016	1	152282092	missense	T	C	0.133	p.E1757G
9635	*C1QTNF7*	31911	4	15444308	missense	C	A	0.104	p.T252K
9635	*CCDC125*	176816	5	68616126	missense	C	A	0.105	p.S81I
9635	*FAM8A1*	16255	6	17601058	missense	G	A	0.093	p.A140T
9635	*FAM8A1*	16255	6	17601086	missense	A	G	0.090	p.Q149R
9635	*FAM8A1*	16255	6	17601098	missense	C	T	0.088	p.S153L
9635	*MUC22*	1198815	6	30997575	missense	C	A	0.107	p.T1456N
9635	*HLA-DQA2*	20056	6	32713076	missense	A	C	0.099	p.S75R
9635	*HLA-DQA2*	20056	6	32713080	missense	A	G	0.097	p.K76R
9635	*HLA-DQA2*	20056	6	32713090	missense	T	A	0.093	p.S79R
9635	*PEG10*	15068	7	94293635	missense	C	T	0.278	p.A290V
9635	*PUF60*	78480	8	144900573	missense	C	T	0.316	p.M160I
9635	*FRMD4A*	18027	10	13698923	missense	T	C	0.190	p.D889G
9635	*GOLGA7B*	1010917	10	99619251	missense	C	A	0.060	p.L17M
9635	*LRRC27*	30626	10	134165176	missense	G	C	0.141	p.E363Q
9635	*CTDSP2*	5730	12	58217698	missense	G	T	0.062	p.P227T
9635	*CTDSP2*	5730	12	58217763	missense	C	T	0.227	p.R205H
9635	*CTDSP2*	5730	12	58217770	missense	G	C	0.062	p.L203V
9635	*CTDSP2*	5730	12	58220811	missense	G	T	0.151	p.L108I
9635	*CTDSP2*	5730	12	58220819	missense	A	G	0.064	p.V105A
9635	*CTDSP2*	5730	12	58220831	missense	C	G	0.119	p.R101T
9635	*CTDSP2*	5730	12	58240188	missense	G	A	0.154	p.R11W
9635	*TMEM260*	17799	14	57051736	missense	G	A	0.073	p.A60T
9635	*WDR89*	80666	14	64066352	missense	T	A	0.080	p.R103S
9635	*WDR89*	80666	14	64066367	missense	A	T	0.120	p.D98E
9635	*WDR89*	80666	14	64066395	missense	C	T	0.092	p.C89Y
9635	*WDR89*	80666	14	64066398	missense	G	C	0.092	p.A88G
9635	*WDR89*	80666	14	64066402	missense	A	G	0.094	p.S87P
9635	*GALNT16*	20692	14	69806253	missense	G	C	0.262	p.K368N
9635	*LDHAL6B*	33195	15	59500155	missense	G	A	0.070	p.S339N
9635	*CDK12*	16507	17	37649029	missense	A	G	0.053	p.R712G
9635	*CDC27*	1293091	17	45229221	missense	C	T	0.182	p.E341K
9635	*CDC27*	1293091	17	45229228	missense	A	C	0.148	p.N338K
9635	*CDC27*	1293091	17	45229234	missense	A	C	0.138	p.S336R
9635	*ST6GALNAC1*	18414	17	74622824	missense	C	A	0.057	p.R275L
9635	*JPH2*	20433	20	42788604	missense	C	T	0.164	p.E275K

*Based on NCBI human reference genome GRC Build 37 (hg19).

Supplementary Table 9. Identification of proteins associated with wide type or mutated Gαq protein by co-immunoprecipitation and spectra count based label free proteomics.

NO	Bait	Prey	PreyGene	Spec	SpecSum	AvgSpec	Number of Replicates	ctrlCounts	SaintScore*	FoldChange	FDR
1	WT	A8K5J7	BAG5	27708|24152|45292	31616	10538.67	3	0|0|0	1	105386.67	0
1	T96S	A8K5J7	BAG5	23088|58096|6872	22520	7506.67	3	0|0|0	1	75066.67	0
2	WT	O60884	DNAJA2	60760|12616|20408	28248	9416	3	0|0|0	1	94160	0
2	T96S	O60884	DNAJA2	22480|18008|53736	28688	9562.67	3	0|0|0	1	95626.67	0
3	WT	O95816	BAG2	63744|31072|56136	19880	6626.67	3	0|0|0	1	66266.67	0
3	T96S	O95816	BAG2	31072|56784|36928	59248	19749.33	3	0|0|0	1	197493.33	0
4	WT	P0DMV9	HSPA1B	27192|38208|10728	10592	3530.67	3	0|0|0	1	35306.67	0
4	T96S	P0DMV9	HSPA1B	45200|41136|56160	11424	3808	3	0|0|0	1	38080	0
5	WT	P31689	DNAJA1	5760|39736|57712	37672	12557.33	3	0|0|0	1	125573.33	0
5	T96S	P31689	DNAJA1	6680|48056|30320	19520	6506.67	3	0|0|0	1	65066.67	0
6	WT	P31943	HNRNPH1	21784|40136|50640	47024	15674.67	3	31984|55664|46736	1	14.2	0
6	T96S	P31943	HNRNPH1	59032|29240|12208	34944	11648	3	31984|55664|46736	1	10.55	0
7	WT	P54652	HSPA2	13768|23936|16088	53792	17930.67	3	0|0|0	1	179306.67	0
7	T96S	P54652	HSPA2	54976|48200|54728	26832	8944	3	0|0|0	1	89440	0
8	WT	P60866	RPS20	25808|44672|20240	25184	8394.67	3	0|26872|42288	1	6.95	0
8	T96S	P60866	RPS20	8264|15712|63944	22384	7461.33	3	0|26872|42288	1	6.18	0
9	WT	P62873	GNB1	12072|5336|50568	2440	813.33	3	0|0|0	1	8133.33	0
9	T96S	P62873	GNB1	27112|35624|29856	27056	9018.67	3	0|0|0	1	90186.67	0
10	WT	P62879	GNB2	31400|39432|36352	41648	13882.67	3	0|0|0	1	138826.67	0
10	T96S	P62879	GNB2	33168|27856|43904	39392	13130.67	3	0|0|0	1	131306.67	0
11	WT	Q9BQA1	WDR77	35776|27456|31888	29584	9861.33	3	62480|63776|6832	1	14.67	0
11	T96S	Q9BQA1	WDR77	22440|3168|12928	38536	12845.33	3	62480|63776|6832	0.99	19.12	0.01
12	WT	H0YMM1	ANXA2	23808|25620|10572	60000	20000	3	0|58880|29940	0*	2.58	1
12	T96S	H0YMM1	ANXA2	55316|64400|59088	47732	15910.67	3	0|58880|29940	1	2.05	0
13	WT	P49411	TUFM	31128|3096|21336	55560	18520	3	0|0|7916	0.67*	7.02	0.33
13	T96S	P49411	TUFM	42752|60568|53416	25664	8554.67	3	0|0|7916	1	3.24	0
14	WT	Q53FC7	HSP70B	52176|52000|15552	54192	18064	3	0|0|0	1	180640	0
14	T96S	Q53FC7	HSP70B	0|26928|48512	9904	3301.33	3	0|0|0	0.67*	33013.33	0.33
15	WT	Q6IPH7	RPL14	35224|31380|55752	56820	18940	3	21048|5976|40408	1	29.97	0
15	T96S	Q6IPH7	RPL14	0|62716|22352	19532	6510.67	3	21048|5976|40408	0.67*	10.3	0.33
16	WT	Q6IPT9	EEF1A1	24400|64640|24656	48160	16053.33	3	25136|54144|58304	1	7.4	0
16	T96S	Q6IPT9	EEF1A1	49928|8064|62336	54792	18264	3	25136|54144|58304	0.67*	8.41	0.33
17	WT	Q9P219	CCDC88C	50448|44696|24724	54332	18110.67	3	0|0|0	1	181106.67	0
17	T96S	Q9P219	CCDC88C	50448|44696|24724	54332	18110.67	3	0|0|0	1	181106.67	0

*Significance Analysis of INTeractome (SAINT) expression was utilized to calculate the probability of protein-protein interaction from background, nonspecific interactions with a SAINT probability threshold of ⩾ 0.8. The five proteins (ANXA2, TUFM, HSP70B, RPL14 and EEF1A1) showed SAINT probability scores of < 0.8 for one of the baits, suggesting that their binding is influenced by the presence/absence of the T96S mutation.

Supplementary Table 10. Significant pathways identified by gene set enrichment analysis

NAME	SIZE	ES	NES	NOM p-val	FDR q-val
KEGG_ETHER_LIPID_METABOLISM	26	0.61113083	1.7556682	0	0.14206062
KEGG_INTESTINAL_IMMUNE_NETWORK_FOR_IGA_PRODUCTION	41	0.50478137	1.5370177	0	0.21443069
KEGG_TERPENOID_BACKBONE_BIOSYNTHESIS	15	0.63365686	1.5663483	0	0.21745014
**KEGG_T_CELL_RECEPTOR_SIGNALING_PATHWAY**	101	0.33099383	1.5453835	0	0.21775618
KEGG_ALLOGRAFT_REJECTION	28	0.54870063	1.5702128	0	0.23435001
KEGG_TYPE_I_DIABETES_MELLITUS	33	0.4488917	1.4510427	0	0.24647677
KEGG_FOCAL_ADHESION	194	0.38443148	1.4522494	0	0.2570027
KEGG_AXON_GUIDANCE	124	0.48318574	1.5887975	0	0.261466
KEGG_COMPLEMENT_AND_COAGULATION_CASCADES	58	0.56535196	1.4586229	0	0.26914763
KEGG_HEMATOPOIETIC_CELL_LINEAGE	73	0.49186575	1.4634719	0	0.27680755
KEGG_NEUROTROPHIN_SIGNALING_PATHWAY	120	0.2868243	1.4270812	0	0.28059024
KEGG_GLYCEROPHOSPHOLIPID_METABOLISM	67	0.3541833	1.3977689	0	0.2811309
KEGG_GLYCOSAMINOGLYCAN_DEGRADATION	20	0.55429554	1.4066336	0	0.2854676
KEGG_ASTHMA	21	0.60966164	1.4809217	0	0.28819868
KEGG_AUTOIMMUNE_THYROID_DISEASE	32	0.5157162	1.4140673	0	0.28938106
**KEGG_MAPK_SIGNALING_PATHWAY**	244	0.3336603	1.4852967	0	0.30279276
KEGG_ALPHA_LINOLENIC_ACID_METABOLISM	15	0.6568755	1.5890207	0	0.31483272
KEGG_ECM_RECEPTOR_INTERACTION	81	0.4195791	1.3275493	0	0.33419177
KEGG_ARRHYTHMOGENIC_RIGHT_VENTRICULAR_CARDIOMYOPATHY_ARVC	69	0.43407646	1.3303643	0	0.34663492
KEGG_HYPERTROPHIC_CARDIOMYOPATHY_HCM	75	0.4339285	1.3510684	0	0.34878737
KEGG_DILATED_CARDIOMYOPATHY	80	0.43336964	1.3574308	0	0.35530043
KEGG_GRAFT_VERSUS_HOST_DISEASE	31	0.4115281	1.2700267	0	0.39485765
KEGG_CELL_ADHESION_MOLECULES_CAMS	123	0.3470763	1.2585121	0	0.39760044
KEGG_ENDOCYTOSIS	173	0.30305654	1.5913094	0	0.40377685
KEGG_CALCIUM_SIGNALING_PATHWAY	157	0.3212866	1.2704787	0	0.40604636
KEGG_LINOLEIC_ACID_METABOLISM	23	0.5034449	1.2907393	0	0.40924773

Supplementary Table 11. Associations between *GNAQ* T96S mutations and clinical characteristics.

Characteristics	Total (n=99)	Wild type (n=90)	T96S (n=9)	P
Age (years)				0.336
≤60	86	79	7	
>60	13	11	2	
Gender				0.719
Male	70	64	6	
Female	29	26	3	
Ann Arbor stage				
I/II	68	65	3	0.025
III/IV	31	25	6	
Serum LDH				0.719
Normal	69	62	7	
Increased	30	28	2	
B symptoms				0.149
Absence	60	57	3	
Presence	39	33	6	
Primary site				0.592
Nasal	89	80	9	
Non-nasal	10	10	0	
ECOG PS				0.154
0-1	91	84	7	
≥2	8	6	2	
Ki67				0.035
≤50%	46	45	1	
>50%	53	45	8	
IPI score				0.622
0-2	84	77	7	
3-5	15	13	2	
TCR expression				1.000
NK cell origin (βF1/ TCRγ -)	95	86	9	
T cell origin (βF1/ TCRγ +)	4	4	0	
EBER				N/A
Positive	99	90	9	
Negative	0	0	0	
CD3ε				N/A
Positive	99	90	9	
Negative	0	0	0	
CD2				1.000
Positive	96	87	9	
Negative	3	3	0	

CD4				N/A
Positive	0	0	0	
Negative	99	90	9	
CD8				1.000
Positive	1	1	0	
Negative	98	89	9	
TCR rearrangement				0.547
No	91	83	8	
Yes	8	7	1	
Therapy				0.495
Chemotherapy alone	50	44	6	
Radiotherapy alone	7	7	0	
Chemotherapy + Radiotherapy	42	39	3	

Abbreviations: Serum LDH, Serum lactate dehydrogenase; ECOG PS, Eastern Cooperative Oncology Group performance status; IPI, International Prognostic Index; EBER, Epstein-Barr virus (EBV)-encoded small RNAs; N/A, not applicable.

Supplementary Table 12. Univariate and multivariate analysis for clinical and biological characteristics of overall survival and progression-free survival in NKTCL.

Univariate analysis

	OS	PFS
	P (FDR*)	*P* (FDR*)
Age＞60	0.856	0.661
Gender (male)	0.692	0.165
B symptoms	0.010	0.018
Primary site (non-nasal)	0.013	0.003
IPI score＞2	< 0.001	< 0.001
Ki67>50%	0.018	0.034
Therapy (CT vs RT vs CMT)	0.084	0.164
DDX3X mutation	0.001 (0.033)	0.008 (0.059)
TP53 mutation	0.003 (0.043)	< 0.001 (0.013)
GNAQ T96S mutation	0.006 (0.048)	0.005 (0.059)

*Derived *P* values were adjusted for FDR using the Benjamini-Hochberg method.

Multivariate analysis

	OS		PFS	
	HR (95%CI)	P	HR (95%CI)	P
B symptoms	1.673 (0.843–3.319)	0.141	1.540 (0.816–2.906)	0.183
Primary site (non-nasal)	3.328 (1.169–9.472)	0.024	3.311 (1.269–8.633)	0.014
IPI score＞2	3.996 (1.760–9.071)	0.001	3.783 (1.727–8.283)	0.001
Ki67>50%	1.791 (0.875–3.668)	0.111	1.709 (0.896–3.260)	0.104
GNAQ T96S mutation	3.314 (1.333–8.242)	0.010	2.847 (1.273–6.368)	0.011

Abbreviations: IPI, International Prognostic Index; CT, Chemotherapy alone; RT, Radiotherapy alone; CMT, Chemotherapy + Radiotherapy; HR, hazard ratio. CI, confidence interval.

Supplementary Table 13. Primers for genotyping knockout mice.

	Primers	Sequence	Product size
Flanking *LoxP* 1 (P1)	F1	GCATTACGGTATGTCAGATACAAC	*floxed:* 244bp
	R1	TCGGTCCAATCCATCTTCCAT	wild-type: 210bp
Ncr1-Cre (P2)	*Ncr1-cre*-F	GACCATGATGCTGGGTTTGGCCCAGATG	*Ncr1-Cre:* 500bp
	Ncr1-cre-R	ATGCGGTGGGCTCTATGGCTTCTG	

Supplementary Table 14.Somatic copy-number alterations of the 28 exome-sequencing cases.

Cytoband	Wide Peak Boundaries*	P_value	Q_value	8221	8191	8184	9638	8208	9626	9624	3329	9616	3326	9622	3325	8198	3324	8219	9623	3327	3336	151	8186	3337	8210	9634	8194	8751	8188	8199	149
10q22.1	chr10:70998001-73827499	0.00053207	0.0023316	5.7	4	4.7	2	2	4	3.3	2.7	2	2	2	2	2	2	5.3	2	1.3	4	2	3	5.7	4	2	2	5.7	5.7	2	1.3
10q22.2	chr10:75522251-75758249	5.11E-10	3.18E-09	5.7	5.7	5.3	2	2	5	4	4	2	4	2	4	2	2	4.7	4	1.3	5	2	2	5	4	2	4	5.7	5.7	4	3.3
10q24.31	chr10:102045251-102295999	0.00010976	0.0003657	5.7	4	4	2	2	2	4	2.7	2	4	2	2	2	3	4	4	1.3	4	2	2	5	3	2	5	5.7	4	4	1.3
10q26.3	chr10:133758001-135534747	0.0013974	0.004859	5.7	3	5.7	3	2	2	4	2.7	2	4	2	2	1	2	5.7	2	1.3	5	2	2	5.7	2	2	2	4	5.7	2	1.3
11p11.2	chr11:47598501-47603999	0.013851	0.060619	5.7	5	4	2	2	3	2.7	2	2	2	2	2	2	2	5.3	2	1.3	4	2	2	5.7	5.7	2	2	5.7	5.7	2	1.3
11p15.1	chr11:17526501-17757749	0.15131	0.18901	5.7	5	4.7	2	2	3	3.3	2.7	2	2	2	2	2	2	5.3	2	1.3	4	2	2	5.7	2	2	2	4	4	2	1.3
11p15.4	chr11:5988751-6226749	1.82E-09	1.15E-08	5.7	5.7	5.7	2	2	4	4	2.7	2	4	2	4	3	3	5.7	5.7	2.7	5.7	4	2	2	4	2	4	5.7	5.7	5	2.7
11p15.5	chr11:1-2966999	0.00053207	0.0030999	5.7	4	5.7	3	2	4	4	2.7	2	4	2	2	2	2	5.7	2	1.3	5.7	2	2	5	4	2	2	5.7	5	2	1.3
11q12.1	chr11:56948501-57144249	0.0032655	0.022154	5.7	4	4.7	2	2	4	4	3.3	2	2	2	2	2	2	3.3	4	1.3	5.7	2	2	5.7	4	2	2	5.7	4	3	1.3
11q12.3	chr11:62341501-62648749	6.91E-06	0.00010453	5.7	5.7	4.7	2	2	3	4	3.3	2	5	2	3	3	2	4	4	1.3	4	3	4	5	2	2	3	5.7	5.7	4	1.3
11q13.1	chr11:65265251-65273499	3.45E-10	8.58E-09	4	4	5.7	1	2	4	4	2.7	2	4	2	4	5.7	5	5.7	2	4.7	5.7	3	4	5.7	3	2	4	5.7	5.7	3	2.7
11q23.3	chr11:118483751-120201249	0.0077282	0.0077282	5.7	4	5.3	2	2	4	2.7	2.7	2	2	2	2	2	2	4.7	2	1.3	4	3	2	5	4	2	3	5.7	5.7	2	1.3
12p13.31	chr12:6976001-7075249	6.91E-06	6.91E-06	5.7	4	4.7	2	2	4	4	3.3	2	5.7	2	3	2	2	4	2	1.3	4	2	3	5.7	2	2	2	5.7	5.7	4	1.3
12q13.12	chr12:49980501-50136249	1.95E-06	2.40E-05	5.7	4	4.7	2	2	4	4	2.7	2	3	2	3	2	2	5.3	3	1.3	4	2	2	5	4	2	4	5.7	5.7	4	1.3
12q13.2	chr12:56029751-58009249	9.73E-07	1.27E-05	5.7	5	4.7	2	2	4	4	2.7	2	4	2	3	2	2	4	3	1.3	4	3	2	5	2	2	5	5.7	5.7	4	1.3
12q24.12	chr12:111741251-111893999	0.047739	0.15856	4	5.7	5.7	2	2	2	4	1.3	2	2	2	2	2	2	5.7	2	1.3	2	2	2	4	2	2	2	4	5.7	2	1.3
12q24.31	chr12:123334001-123485499	1.48E-05	8.22E-05	5.7	5	5.7	3	2	2	4	3.3	2	4	2	2	2	2	5.7	2	1.3	5.7	4	2	5.7	2	2	2	4	5.7	2	1.3
12q24.31	chr12:124371001-133446499	0.0032655	0.0062965	5.7	4	4	2	2	2	2.7	3.3	2	5	2	2	2	2	5.7	2	1.3	4	2	2	5.7	2	2	2	4	5.7	2	1.3
13q34	chr13:114497751-114806749	0.0077282	0.0077282	5.7	4	4.7	4	2	2	2.7	2.7	2	2	2	2	2	2	5.7	2	1.3	4	3	2	4	2	2	2	4	5.7	2	1.3
14q11.2	chr14:22591001-22965749	2.96E-08	0.00027281	4	5.7	2.7	3	2	4	2.7	2.7	2	5.7	2	4	2	5.7	2.7	4	4	4	4	5.7	2	4	4	4	5	4	4	2.7
14q12	chr14:24600501-24777249	1.42E-10	7.93E-07	5.7	5.7	5.7	2	2	4	4	4	2	4	2	4	4	2	4.7	4	1.3	5.7	3	3	5.7	4	2	4	5.7	5.7	4	2.7
14q23.3	chr14:65193751-66028499	0.01135	0.028511	4	5.7	5.3	2	2	2	3.3	2	2	2	2	2	2	4	4.7	2	2.7	4	2	2	4	3	2	2	5.7	5	2	1.3
14q24.3	chr14:74705501-75052999	0.033529	0.076371	5.7	4	2.7	2	2	2	2.7	4	2	2	2	2	2	2	5.7	2	1.3	5	2	2	4	3	2	2	5	5.7	2	1.3
14q32.33	chr14:104638751-107349540	0.00011734	0.00017239	5.7	4	5.7	4	2	2	4	1.3	2	5	2	2	1	2	5.7	2	1.3	4	3	2	5.7	2	2	2	5.7	5.7	2	1.3
15q15.1	chr15:42058501-42305999	0.00011734	0.00053099	5.7	4	5.7	2	2	2	4	3.3	2	2	2	3	2	2	5.7	2	1.3	5.7	3	2	5.7	2	2	2	5.7	5.7	2	2
15q15.1	chr15:41031501-41247999	0.011787	0.051365	5	4	5.7	2	2	2	4	2.7	2	2	2	2	2	2	5.7	2	1.3	5	2	2	2	4	2	2	5.7	5.7	2	1.3
15q15.1	chr15:40544251-40866499	0.013851	0.061644	5	4	5.7	2	2	2	3.3	2.7	2	4	2	2	2	2	5.7	2	1.3	4	2	2	2	4	2	2	5.7	5.7	2	1.3
15q21.1	chr15:45364751-45545749	0.024164	0.11594	5.7	5	3.3	2	2	3	2.7	1.3	2	2	2	2	2	4	4.7	2	1.3	5	3	2	2	2	2	2	5.7	4	2	2
15q24.1	chr15:73614001-75660999	0.0032655	0.035828	5.7	4	5.3	2	2	2	2.7	2.7	2	2	2	2	2	2	4.7	2	1.3	4	4	2	5	2	2	3	5	5.7	2	1.3
15q26.1	chr15:91448251-91503749	0.00014941	0.00078684	5.7	5.7	5.7	3	2	2	4	2.7	2	2	2	2	2	2	5.7	2	1.3	5	2	2	5	3	2	2	5.7	5.7	2	1.3
15q26.1	chr15:79215001-90905749	0.0059741	0.16545	5	4	4.7	2	2	2	3.3	2.7	2	2	2	3	2	2	4.7	2	1.3	4	2	2	5	3	2	2	5.7	5.7	2	1.3
16p11.2	chr16:28497001-31336499	0.0059741	0.0059741	5.7	4	5.7	2	2	3	3.3	2.7	2	5	2	4	2	2	5.7	2	1.3	5	2	4	5.7	3	2	3	5.7	5.7	4	1.3
16p13.3	chr16:1-105499	0.0027896	0.0027896	5.7	4	5.7	2	2	4	4	3.3	2	4	2	2	2	2	5.3	2	1.3	5.7	3	2	5.7	2	2	5	5.7	5.7	5.7	1.3
16q22.1	chr16:67182251-67264749	0.034747	0.034747	5.7	5	5.7	2	2	2	4	3.3	2	5.7	2	3	2	2	5.7	2	1.3	5.7	3	2	5.7	2	2	2	5.7	5.7	2	1.3
17p11.2	chr17:16843001-19459249	0.020377	0.038272	5.7	4	4.7	2	2	3	3.3	2.7	2	2	2	3	2	2	5.7	3	1.3	4	2	2	5	2	2	2	5.7	5.7	2	1.3
17p13.1	chr17:7720501-8198249	4.57E-09	7.33E-09	5.7	5.7	5.7	2	2	4	4.7	2.7	2	5	2	4	2	2	5.7	4	1.3	5.7	3	4	5.7	3	2	4	5.7	5.7	4	1.3
17p13.2	chr17:4794751-4851999	0.0065873	0.0065873	5.7	4	4.7	2	2	2	4	3.3	2	5.7	2	2	2	2	4.7	2	1.3	5	2	2	5.7	2	2	2	5.7	5.7	2	1.3
17q11.2	chr17:26655251-27022749	6.45E-05	0.00017586	5.7	5	4.7	2	2	4	3.3	2.7	2	3	2	2	2	2	5.7	4	1.3	4	4	2	5.7	4	2	4	5.7	5	3	1.3
17q11.2	chr17:27888751-27959499	0.0018981	0.0036057	5.7	5.7	5.7	2	2	2	4	2.7	2	2	2	2	2	3	5.7	2	2	5.7	2	2	5.7	2	2	2	5.7	5.7	2	1.3
17q21.2	chr17:39323251-39657249	4.92E-05	0.00014941	5.7	5	5.3	2	2	4	3.3	2.7	2	2	4	2	2	2	5.3	2	2	4	2	4	5.7	4	2	2	5.7	5.7	4	2
17q21.31	chr17:42750751-44110999	0.00066624	0.018813	5.7	4	4.7	2	2	3	4	2.7	2	2	2	3	2	2	5.7	2	1.3	5	2	2	5.7	4	2	2	5.7	5.7	4	1.3
17q21.33	chr17:47920501-48761499	0.00053207	0.029018	5.7	4	5.7	2	2	3	3.3	2.7	2	2	2	2	2	2	5.7	3	1.3	5	2	2	5	4	2	2	5.7	5.7	4	1.3
17q22	chr17:54587001-56621749	0.00092532	0.0082362	5	5	4.7	2	2	4	3.3	2.7	2	2	2	2	2	2	4	4	1.3	5	2	2	5	4	2	2	5.7	5	4	1.3

17q23.3	chr17:61895001-61907999	1.47E-05	5.96E-05	5.7	4	5.3	2	2	5	3.3	2.7	2	2	2	2	2	2	4.7	4	1.3	5	2	3	5.7	5	2	2	5.7	5	5	2.7
17q23.3	chr17:60748251-61788499	0.0288	0.08947	5.7	4	4	2	2	4	3.3	2.7	2	2	2	2	2	2	4	2	1.3	5	2	3	5.7	2	2	2	5.7	5	2	1.3
17q25.1	chr17:73227501-73916499	0.00017915	0.00017915	5.7	4	5.7	2	2	2	3.3	2.7	2	4	3	2	2	2	5.7	2	1.3	5.7	3	2	5.7	2	2	3	5.7	5.7	4	1.3
18p11.22	chr18:9234751-9256749	0.01135	0.01135	4	4	2.7	2	2	2	1.3	1.3	2	5.7	2	5.7	5	4	2.7	2	3.3	2	5	2	2	2	2	2	2	4	2	3.3
18q21.1	chr18:47805251-47806499	0.00013499	0.0013974	5.7	5.7	5.7	2	2	4	2.7	4.7	2	5	2.9	2	2	2	3.3	2	1.3	5.7	2	2	2	2	2	4	4	5.7	2	1.3
18q21.1	chr18:43795251-44301499	0.0095186	0.14075	5.7	5.7	3.3	2	2	5	4.7	2	2	2	2	2	2	2	3.3	4	1.3	5	2	2	2	2	2	2	4	4	3	1.3
18q23	chr18:72672251-78077248	0.040468	0.040468	4	4	4.7	4	2	2	2.7	2	2	4	2	2	2	2	4.7	2	1.3	4	2	2	2	3	2	2	4	5	3	1.3
19p13.11	chr19:15052501-19790499	0.0032655	0.030586	5.7	4	4.7	2	2	2	2.7	2.7	2	2	2	2	2	2	5.7	2	1.3	4	2	2	5.7	4	2	3	5.7	5.7	4	1.3
19p13.12	chr19:12815251-14717749	0.00041796	0.0037631	5.7	4	5.7	2	2	2	4	2.7	2	4	2	3	2	2	5.7	2	1.3	4	2	5	5.7	2	2	2	5.7	5.7	2	1.3
19p13.2	chr19:9045251-9091749	6.88E-07	1.16E-05	5.7	5.7	4	2	2	5.7	4	2.7	2	2	2	4	2	2	4	4	1.3	5.7	2	2	5.7	5	2	4	5.7	4	4	2.7
19p13.2	chr19:9921001-11660749	0.0040378	0.0040378	5.7	4	5.3	2	3.3	2	3.3	2.7	2	4	2	2	2	2	5.7	2	1.3	4	2	2	5.7	2	2	2	5.7	5.7	2	1.3
19p13.3	chr19:1-2813249	0.00053207	0.0069643	5.7	4	5.7	3	2	2	2.7	2.7	2	4	2	2	2	2	5.7	2	1.3	5	2	4	5.7	3	2	2	5.7	5.7	2	1.3
19p13.3	chr19:2938751-6831999	0.0059741	0.070781	5.7	4	5.7	2	2	2	2.7	2.7	2	4	2	2	2	2	5.7	2	1.3	5	2	2	5.7	2	2	2	5.7	5.7	2	1.3
19q13.12	chr19:35448251-36637249	1.18E-05	1.74E-05	5.7	4	5.7	2	2	3	4	2.7	2	4	2	5	2	2	5.7	2	1.3	5.7	2	2	5.7	3	2	2	5.7	5.7	4	1.3
19q13.2	chr19:39875751-40487249	4.92E-05	0.00014927	5.7	5.7	5.7	2	2	2	4	2.7	2	3	2	4	4	2	4.7	2	1.3	5	2	3	5.7	2	2	2	5.7	5	4	1.3
19q13.31	chr19:40719251-44306749	0.00022291	0.00054954	5.7	4	5.7	2	2	2	4	2.7	2	4	2	5	2	2	5.7	2	1.3	4	2	2	5.7	2	2	2	5.7	5.7	4	1.3
19q13.33	chr19:48789251-52327499	0.00010976	0.00032197	5.7	4	5.7	2	2	2	4	2.7	2	4	2	5	2	2	5.7	2	1.3	5	2	4	5.7	2	2	2	5.7	5.7	2	1.3
19q13.42	chr19:55856751-56041749	0.00010976	0.00097242	5.7	4	5.7	3	2	2	4	2.7	2	5	2	5	2	2	5.7	2	1.3	5	2	2	5.7	2	2	2	5.7	5.7	2	1.3
19q13.43	chr19:58981751-59128983	1.48E-05	8.66E-05	5.7	5	5.7	2	2	4	3.3	2.7	2	3	2	5	2	2	5.7	2	1.3	5	2	3	5.7	2	2	4	5.7	5.7	2	2
1p13.3	chr1:109642001-110884999	0.0011277	0.019067	5.7	4	4.7	2	2	4	2.7	2.7	2	2	2	2	2	2	4.7	3	1.3	4	2	2	5.7	4	2	4	5.7	4	2	1.3
1p32.3	chr1:55066001-55537749	0.040468	0.313	5.7	4	4.7	2	2	2	2.7	1.3	2	2	2	2	2	2	4	2	1.3	4	2	2	5	2	2	3	5.7	5	2	1.3
1p34.1	chr1:39733501-46087499	0.0013974	0.22173	5.7	5.7	4.7	2	2	4	4	2.7	2	2	2	2	2	2	4	2	1.3	5	2	2	5	4	2	2	5.7	4	2	1.3
1p36.13	chr1:12515501-35449499	0.0032655	0.095809	5.7	4	4.7	2	2	2	2.7	2.7	2	2	2	2	2	2	4.7	4	1.3	4	2	2	5	4	2	4	5.7	4	2	1.3
1p36.33	chr1:1-6579499	0.00015895	0.0036776	5.7	4	5.7	3	2	2	2.7	2.7	2	5	2	3	2	2	5.7	2	1.3	5.7	2	2	5.7	2	2	2	5.7	5.7	2	1.3
1q21.1	chr1:114353501-153974249	0.00010976	0.20241	5	5	3.3	2	2	4	3.3	2.7	2	5.7	2	2	5	2	1.3	3	2	4	2	2	2	4	2	4	5.7	5	4	2.7
1q21.3	chr1:154232001-155308249	8.49E-08	0.00069826	5.7	5.7	5.7	2	2	4	3.3	2.7	2	4	2	3	2	2	5.3	3	1.3	5.7	2	2	5	4	2	4	5.7	5	4	2.7
1q23.3	chr1:155899001-165620499	2.65E-05	0.035463	4	4	4	2	2	4	3.3	2.7	2	5	2	3	3	2	2.7	4	1.3	4	2	3	5	4	2	4	5.7	4	4	2.7
1q32.1	chr1:200841501-207287749	0.00017586	0.043267	5.7	4	5.3	2	2	4	3.3	3.3	2	2	2	2	2	2	4	4	2.7	4	2	2	5	4	2	3	5.7	4	3	2.7
1q32.2	chr1:207974251-209964249	6.45E-05	0.010484	5	5.7	5.3	2	2	4	3.3	2.7	2	2	2	2	2	2	4	4	2.7	5	2	2	5	4	2	3	5.7	4	3	2.7
1q42.13	chr1:227919751-228871999	0.004859	0.01883	5.7	4	5.7	2	2	2	4.7	2.7	2	2	2	2	2	2	5.7	2	2.7	4	2	2	5.7	2	2	2	4	5.7	2	1.3
1q44	chr1:247485251-249250621	0.02849	0.06218	4	4	3.3	2	2	4	3.3	1.3	2	2	3	2	2	2	2.7	4	2.7	5.7	2	2	2	4	2	4	3	4	2	2.7
20p13	chr20:1-2842249	0.00022291	0.00032368	5.7	5	4.7	2	2	4	3.3	2.7	2	2	2	2	2	2	4.7	4	1.3	4	2	2	5	4	2	4	5.7	5.7	3	1.3
20p13	chr20:3626501-4765249	0.0095186	0.012932	5.7	4	5.7	2	2	2	4	2.7	2	2	2	2	2	2	5.7	2	1.3	5	2	2	5	2	2	2	5.7	5.7	2	1.3
20q11.21	chr20:21491751-34295999	0.016502	0.025607	5.7	5.7	4.7	2	2	2	4.7	2	2	2	2	3	2	2	4	3	1.3	4	2	2	4	3	2	2	5.7	5	3	1.3
20q13.12	chr20:44438751-45012249	0.00041796	0.0011596	5.7	5.7	5.7	2	2	4	4	2.7	2	2	2	3	2	2	5.3	2	1.3	5	2	2	4	4	2	4	5.7	5.7	2	1.3
20q13.33	chr20:60881751-60887999	2.10E-05	4.80E-05	5.7	4	5.3	3	2	2	3.3	2.7	2	5	2	5	1	2	5.7	2	1.3	5.7	3	2	5.7	2	2	2	5.7	5.7	3	1.3
21q22.11	chr21:31743501-32490749	0.016166	0.016166	4	4	3.3	2	3	4	3.3	2.7	2	2	2	2	2	4	2.7	4	2.7	4	2	2	3	4	2	2	4	4	4	2.7
21q22.3	chr21:45501751-47614749	0.0003657	0.0003657	5.7	4	5.3	2	2	2	2.7	2.7	2	2	2	5.7	1	1	5.7	2	1.3	5.7	2	2	5.7	2	2	2	5.7	5.7	3	1.3
22q12.2	chr22:30642251-31743249	0.057733	0.083824	5.7	4	5.7	2	2	4	4	2.7	2	2	2	3	2	2	5.3	2	1.3	5	2	2	5	3	2	2	5.7	5.7	2	1.3
22q12.3	chr22:36592751-38884249	0.047739	0.095018	5.7	5	5.3	2	2	4	2.7	2.7	2	2	2	2	2	2	5.7	4	1.3	4	2	2	5	3	2	2	5.7	5.7	2	2.7
22q13.1	chr22:39066251-39120499	0.0062965	0.0084111	5.7	5	4.7	2	2	2	2.7	2.7	2	2	2	2	2	2	4.7	2	1.3	4	2	2	5.7	5	2	5	5.7	5.7	5	2
22q13.33	chr22:50925751-51304566	2.76E-06	2.76E-06	5.7	5	5.7	2	2	4	4	2.7	2	4	2	4	2	2	5.7	2	1.3	5.7	4	2	5.7	5	2	4	5.7	5.7	4	1.3
2p11.2	chr2:85042251-128479249	0.01962	0.1588	5.7	4	4.7	2	2	2	3.3	2.7	2	2	2	2	2	2	2.7	3	1.3	5	2	2	4	2	2	2	5.7	5.7	2	1.3
2p13.1	chr2:74681751-74687499	8.64E-14	5.67E-13	5.7	5.7	5.7	2	2	4	4.7	4	3	5.7	2	4	2	2	5.7	4	1.3	5.7	4	4	4	4	2	4	5.7	5.7	4	1.3
2p23.3	chr2:26851501-27682249	1.95E-06	1.95E-06	5.7	5	5.3	3	2	4	3.3	3.3	2	2	2	3	2	2	4.7	4	1.3	4	2	2	5	4	2	3	5.7	5.7	3	1.3
2q21.1	chr2:130938251-133541999	0.00041796	0.00089881	5.7	5	5.7	2	2	2	4	2.7	2	2	2	2	2	2	5.7	2	1.3	5.7	2	2	2	2	2	4	5	5.7	4	1.3

2q31.1	chr2:176946001-177054499	0.016502	0.051106	5.7	2	5.7	2	2	2	1.3	2	2	4	2	2	2	4	5.7	2	1.3	5.7	2	3	0.7	2	2	2	2	5.7	2	3.3
2q32.1	chr2:179643501-197854249	0.033529	0.093625	2	2	1.3	1	2	2	1.3	4	2	3	2	4	4	5	1.3	4	3.3	5	5.7	2	0.7	2	1	4	2	2	2	3.3
2q35	chr2:220045001-220115749	8.64E-14	7.07E-13	5.7	5.7	5.7	2	2	4	4	2.7	2	4	2	4	4	3	5.3	4	2	5.7	4	5.7	5	4	2	5	5.7	5.7	3	1.3
2q35	chr2:217524751-219301999	0.01135	0.034747	5.7	4	4.7	2	2	2	3.3	2.7	2	3	2	2	2	3	4.7	2	1.3	5	2	2	1	2	2	2	5.7	5.7	2	1.3
2q37.3	chr2:238977251-243199373	0.00078684	0.0052508	5.7	4	5.7	2	2	2	2.7	2.7	2	2	2	2	2	2	5.7	2	1.3	5.7	2	2	5.7	3	2	2	5.7	5.7	2	1.3
3p21.1	chr3:52810751-52867499	0.0026245	0.05014	5.7	4	5.7	2	2	2	3.3	2.7	2	2	2	2	2	2	5.3	2	1.3	5	3	2	5.7	2	2	2	4	5.7	2	1.3
3p21.31	chr3:50152751-50431499	8.66E-05	0.0031631	5.7	4	5.7	2	2	2	4	2.7	2	5	2	2	2	2	5.3	2	1.3	5	2	2	5.7	3	2	2	5.7	5.7	2	1.3
3p22.2	chr3:38016501-38739999	0.00064955	0.010339	5.7	5	3.3	2	2	4	2.7	2.7	2	2	2	3	2	2	4.7	4	1.3	4	2	2	5	3	2	3	5.7	4	2	2.7
3p25.3	chr3:4941751-9991999	0.00010976	0.00098397	5.7	4	4	2	2	4	2.7	2.7	2	2	2	4	2	2	4	2	1.3	5.7	2	2	5	3	2	3	5.7	5.7	4	1.3
3q21.3	chr3:125724751-129512999	0.002206	0.002206	5.7	4	4.7	2	2	2	2.7	2.7	2	2	2	2	4	2	4.7	2	1.3	4	2	2	5.7	2	2	2	5.7	5.7	3	1.3
3q27.1	chr3:183894501-184081499	3.69E-09	3.69E-09	5.7	5.7	5.3	2	2	4	4	2.7	2	4	2	4	2	2	5.3	4	1.3	5.7	2	2	5.7	4	2	3	5.7	5.7	4	2
4p16.1	chr4:3213251-10117999	0.0095186	0.22057	5.7	4	4	5.7	2	2	2.7	2.7	2	2	2	2	2	2	5.3	2	1.3	4	2	2	4	2	2	2	4	5.7	2	2.7
4p16.3	chr4:676251-1245249	1.48E-05	0.00037239	5.7	4	5.7	4	2	2	4	2.7	2	5	2	2	2	2	5.7	2	1.3	5.7	2	2	5.7	2	2	2	5.7	5.7	2	1.3
4q21.1	chr4:77659251-77941999	0.01962	0.01962	5.7	4	4.7	2	2	2	4	1.3	2	2	2	2	2	2	4	2	2.7	5	2	2	1	4	2	3	4	4	4	2.7
5p15.33	chr5:1-3601249	0.0021115	0.0021115	5.7	4	4	2	2	3	3.3	2	2	3	2	3	1	2	4	4	1.3	5	3	2	1	4	2	2	5.7	5.7	3	1.3
5q31.1	chr5:131704751-131823999	4.99E-06	5.34E-05	5.7	5.7	4.7	2	2	4	4	2.7	2	4	2	2	2	3	4	4	1.3	4	2	4	4	4	2	2	5.7	5.7	3	2.7
5q31.3	chr5:139926501-140044749	4.57E-09	6.70E-08	5.7	5.7	5.7	2	2	4	4	4	2	5	2	4	2	2	4	3	1.3	5	3	2	4	5	2	4	5.7	5.7	4	2.7
5q33.1	chr5:148044251-157158249	1.40E-06	1.28E-05	5.7	5	5.7	2	2	4	4	2.7	2	2	3	3	2	2	4	5	1.3	4	2	2	4	4	2	4	5.7	5.7	4	2.7
5q35.3	chr5:180582001-180915260	3.82E-07	7.63E-05	5.7	5.7	5.7	2	2	4	2.7	2.7	2	5	2	4	2	2	5.7	2	1.3	4	2	2	5	4	2	4	5.7	5.7	4	1.3
5q35.3	chr5:176792001-177020999	3.15E-07	8.66E-05	5.7	5.7	5.7	2	2	3	4	4	2	4	2	4	2	2	5.7	3	1.3	5.7	3	2	5	4	2	2	5.7	5.7	2	1.3
6p12.2	chr6:52008251-52147249	0.047739	0.10564	2	2	1.3	1	2	4	1.3	2	2	2	2	5.7	2	3	1.3	5.7	2.7	2	2	2	1	5.7	2	5.7	5.7	2	2	2.7
6p21.1	chr6:42857501-43492749	1.48E-05	0.001189	5.7	5.7	4.7	2	2	4	4	2.7	2	3	2	2	2	2	5.3	2	1.3	5	2	2	5.7	4	2	4	5.7	5.7	4	1.3
6p21.1	chr6:39023501-42233249	0.00035298	0.0042079	5.7	5	5.3	2	2	4	3.3	2.7	2	2	2	2	2	2	4	2	1.3	4	2	2	5.7	4	2	4	5.7	5.7	4	2
6p21.1	chr6:43590751-44311499	0.00073731	0.020845	5.7	4	5.3	2	2	4	3.3	2.7	2	4	2	2	2	2	4.7	2	1.3	5	2	2	1	2	2	4	5.7	5.7	2	2
6p21.32	chr6:32797001-32943249	8.64E-14	3.57E-09	5.7	5.7	5.3	2	2	4	4	4	2	5.7	2	4	3	3	5.3	4	2	5	4	3	5	5.7	2	5.7	5.7	5.7	4	2.7
6p21.33	chr6:31087501-32190999	1.59E-08	0.019265	5.7	5.7	4.7	2	2	4	4	3.3	2	4	2	4	2	2	4.7	4	1.3	5.7	4	2	5.7	4	2	3	5.7	5.7	4	2.7
6p22.1	chr6:29363751-30916499	8.49E-08	0.0086509	5.7	5.7	4.7	2	2	4	4	3.3	2	2	2	4	2	2	4	5	2.7	5	2	2	5.7	5.7	2	4	5.7	5	4	2.7
6p22.2	chr6:25983001-27115749	4.57E-09	3.40E-06	4	5.7	4.7	2	2	4	4.7	2.7	2	5.7	2	2	5	2	4.7	4	2.7	4	2	4	5.7	4	2	5	5.7	5	4	2.7
6q21	chr6:109786001-109787499	0.0032655	0.0032655	4	5.7	5.7	1	2	2	4	2.7	2	3	2	2	2	3	4.7	2	1.3	5.7	2	2	1	2	2	2	5.7	5.7	2	2
7p13	chr7:41728501-47481749	0.00014941	0.0045717	5.7	5	4.7	2	2	3	4	2.7	2	3	2	4	3	2	5.3	2	1.3	4	2	2	2	3	2	2	5.7	5.7	4	1.3
7p15.2	chr7:27146751-27570999	0.00053207	0.017357	4	4	3.3	3	2	3	2.7	2.7	2	4	2	4	2	2	5.7	2	2	4	2	2	1	4	2	3	5	5.7	4	2.7
7p22.3	chr7:1-5663749	0.0003657	0.0003657	5.7	4	5.7	4	2	2	2.7	2.7	2	4	2	2	2	2	5.7	2	1.3	4	2	2	5.7	2	2	2	5.7	5.7	2	1.3
7q22.1	chr7:99685751-100006499	2.24E-08	2.24E-08	5.7	5	4.7	2	2	4	4	2.7	2	4	2	4	2	2	5.7	4	1.3	5.7	2	2	5.7	2	2	4	5.7	5.7	4	1.3
7q32.1	chr7:128469751-128586749	0.00011734	0.0061524	5.7	4	4.7	2	2	3	3.3	2	2	2	2	4	2	2	5.7	2	1.3	5.7	2	2	5.7	2	2	2	5.7	5.7	4	1.3
7q34	chr7:142205751-142498749	8.05E-13	8.50E-06	5.7	5.7	4.7	2	2	4	4	3.3	2	2	2	5	2	2	4	4	1.3	5.7	3	5	5.7	5.7	3	5	5.7	5.7	4	2.7
7q36.1	chr7:150163001-150492249	1.48E-11	7.70E-05	5.7	4	5.7	2	2	4	4	3.3	2	4	2	4	4	2	5.7	2	1.3	5.7	3	3	5.7	4	2	5	5.7	5.7	4	2.7
8p11.21	chr8:41475501-41791999	0.040468	0.068293	5.7	5	4.7	2	2	4	3.3	2	2	2	2	2	2	2	4.7	2	2	2	2	3	1	2	2	2	5	5.7	2	1.3
8p21.2	chr8:26362001-27528749	0.13026	0.21685	5.7	4	4.7	2	2	2	4	2	2	2	2	2	2	2	4	2	1.3	4	2	2	1	2	2	2	5	5	2	1.3
8p21.3	chr8:22431251-22460999	0.0013974	0.0020625	5.7	4	5.7	2	2	2	4	4	2	2	2	5	2	2	5.3	2	1.3	4	2	2	5.7	2	2	2	5.7	5.7	2	1.3
8p23.1	chr8:10386251-11710999	0.0288	0.0288	5.7	3	4.7	2	2	2	4	2	2	2	2	2	2	2	4	2	1.3	5.7	2	2	5.7	2	2	2	5.7	5	4	1.3
8q24.22	chr8:133898751-134251249	0.0017757	0.010353	4	5.7	4.7	2	2	4	2.7	3.3	2	2	2	4	2	2	4	2	1.3	4	4	2	1	4	2	2	5.7	4	2	2.7
8q24.3	chr8:144641251-144650499	2.10E-05	0.00016769	5.7	4	5.7	2	2	2	4	4	1	5	2	5	2	2	5.7	2	1.3	5.7	2	2	5.7	2	2	2	5.7	5.7	3	1.3
9p13.3	chr9:35605001-35846749	1.42E-10	1.42E-10	5.7	5	5.7	2	2	4	4	3.3	2	5	2	4	2	2	4.7	4	1.3	5.7	4	3	5	5	2	4	5.7	5.7	4	2.7
9q22.31	chr9:95781251-96847249	0.024164	0.024164	4	4	4.7	2	2	3	2.7	3.3	2	2	2	2	2	2	5.7	2	1.3	4	5	2	4	2	2	2	5	5.7	2	1.3
9q32	chr9:116190751-117379749	5.19E-07	1.52E-05	5.7	4	4	2	2	4	4	4.7	2	2	2	4	2	2	5.7	4	1.3	5	2	2	5.7	4	2	4	5.7	5.7	4	2
9q34.11	chr9:130473251-130890749	0.0003657	0.033118	5.7	5	4.7	2	2	2	3.3	2.7	2	4	2	5	2	2	5.3	2	1.3	5.7	2	2	5.7	2	2	2	5.7	5.7	2	1.3

9q34.13	chr9:134348001-134404749	4.92E-05	0.0095862	5.7	5.7	5.3	3	2	4	4	2.7	2	3	2	3	2	2	4.7	2	1.3	4	3	2	5.7	3	2	3	5.7	5.7	2	1.3
9q34.3	chr9:139701251-140387499	1.48E-05	0.00064955	5.7	4	5.7	4	2	2	4	2.7	2	5	2	2	2	2	5.7	2	1.3	5.7	4	2	5.7	2	2	2	5.7	5.7	3	1.3
19p12	chr19:19843751-21987999	1.17E-07	1.17E-07	2	1	1.3	2	2	1	1.3	1.3	2	2	2	1	2	2	1.3	1	1.3	1	2	2	5.7	1	2	2	1	2	2	1.3
1p32.3	chr1:55530501-55681499	0.00041873	0.00041873	2	2	1.3	1	2	2	2	1.3	2	2	2	2	2	4	1.3	2	2.7	2	2	2	1	2	2	3	2	2	2	2.7
19p13.3	chr19:1-362249	0.042472	0.042472	5.7	3	5.3	3	2	2	3.3	1.3	2	3	2	2	1	1	5.7	2	0.7	2	2	2	5.7	3	3	2	4	5.7	2	1.3
13q34	chr13:109860251-110801499	0.077311	0.077311	2	4	3.3	2	2	2	2.7	2.7	1	2	2	2	2	2	4	2	1.3	4	3	2	1	2	2	2	4	2	2	2.7
17q25.3	chr17:79304251-79476999	0.0791	0.077311	5.7	4	5.7	3	2	2	3.3	2.7	2	5	2	2	2	2	5.7	2	1.3	5.7	2	1	5.7	2	2	2	5.7	5.7	2	0.7
20q13.33	chr20:58422751-58508999	0.13627	0.13627	4	4	2.7	0.7	1	3	2.7	2.7	2	2	2	5	2	3	3.3	2	2.7	4	5	2	0.7	2	1	2	5	4	2	4.7
2q32.1	chr2:172341501-191513999	0.17316	0.17316	2	2	1.3	1	2	2	1.3	1.3	2	3	2	2	2	5	1.3	2	3.3	3	4	2	0.7	2	1	4	2	2	2	3.3
10q26.3	chr10:134790001-135093249	0.17316	0.17316	5.7	3	5.7	3	2	2	3.3	2.7	2	4	2	2	1	2	5.7	2	1.3	5	2	1	5.7	2	2	1	4	5.7	2	1.3
16q12.2	chr16:52119001-54317249	0.17316	0.17316	2	2	1.3	1	2	2	1.3	2	2	2	2	2	2	4	1.3	2	2.7	2	2	2	5.7	2	2	2	2	3	2	1.3
18q21.31	chr18:54305751-54814499	0.18144	0.17316	2	2	1.3	2	2	2	1.3	1.3	2	2	2	2	2	3	1.3	2	2.7	2	2	2	2	2	2	2	2	2	2	1.3
12q24.33	chr12:133783501-133851895	0.18144	0.18144	2	2	1.3	1	2	2	1.3	1.3	2	2	2	2	2	2	1.3	2	1.3	3	2	2	5.7	2	1	2	4	3	2	0.7
15q11.2	chr15:25415751-25496249	0.18144	0.18144	4	2	2.7	4	2	2	1.3	1.3	2	2	2	1	2	2	2.7	2	1.3	2	2	2	2	2	2	2	2	2	2	0.7
19p13.2	chr19:9405501-58992999	0.18144	0.18144	2	2	1.3	1	2	2	1.3	1.3	2	2	2	2	2	2	1.3	2	1.3	2	2	2	5.7	2	2	2	2	2	2	0.7
21q22.3	chr21:46874751-46934749	0.18144	0.18144	5.7	4	5.3	2	2	2	2.7	2.7	2	2	2	2	1	1	5.7	2	1.3	5.7	2	2	5.7	2	2	2	5.7	5.7	3	1.3
22q13.1	chr22:39928751-40139249	0.18144	0.18144	5.7	5	4.7	2	2	2	2.7	2.7	2	2	2	2	1	2	4.7	2	1.3	4	2	2	5.7	2	2	3	5.7	5.7	3	1.3
16q12.2	chr16:1-90354753	0.17316	1	2	2	1.3	1	2	2	1.3	2	2	2	2	2	2	4	1.3	2	2.7	2	2	2	5.7	2	2	2	2	3	2	1.3
16q22.1	chr16:1-90354753	0.18144	1	4	2	1.3	1	2	2	2.7	2	2	2	2	2	2	2	2.7	2	1.3	4	2	2	5.7	2	1	2	2	4	2	1.3

Copy number: >2, amplification; <2, deletion.

*Based on NCBI human reference genome GRC Build 37 (hg19).

Supplementary Table 15. Loss of heterozygosity analysis of the 28 cases of NKTCL.

Patient ID	Chromsome	Hugo_Symbol	Start_position*	End_Position*	Nucleotide change	Protein Change	Copy number	Genotype
149	X	MAGEB6	26213093	26213093	c.C1130T	p.A377V	3	AAA
149	X	GPC4	132437337	132437337	c.C1325T	p.A442V	3	AAA
149	15	USP8	50785055	50785055	c.G2074C	p.A692P	3	AAA
149	X	MAGEC1	140996239	140996239	c.G3049T	p.E1017X	3	AAA
149	11	OR5R1	56185159	56185159	c.T550C	p.F184L	3	AAA
149	X	SCML1	17771387	17771387	c.G502A	p.G168R	3	AAA
149	X	MAGEB16	35820795	35820795	c.A482G	p.H161R	3	AAA
149	15	ATP8B4	50226313	50226313	c.C1354A	p.H452N	3	AAA
149	X	KIAA1210	118222360	118222360	c.A2833T	p.I945F	3	AAA
149	19	LOC100379224	44612231	44612231	c.A1918G	p.K640E	2	AA
149	X	SPANXC	140785696	140785696	c.A220G	p.K74E	3	AAA
149	3	ULK4	41756986	41756986	c.T2530A	p.L844M	3	AAA
149	19	LOC100379224	44610665	44610665	c.A352G	p.M118V	2	AA
149	6	OR14J1	29274486	29274486	c.T20C	p.M7T	3	AAA
149	15	USP8	50784955	50784955	c.C1974A	p.N658K	3	AAA
149	2	TTN	179458591	179458591	c.G31241A	p.R10414H	5	AAAAA
149	16	MC1R	89986154	89986154	c.G488A	p.R163Q	2	AA
149	15	USP8	50784950	50784950	c.C1969T	p.R657W	3	AAA
149	17	FOXN1	26851602	26851602	c.C205T	p.R69C	2	AA
149	17	KRTAP29-1	39458893	39458893	c.C211T	p.R71C	3	AAA
149	17	KRTAP16-1	39464736	39464736	c.G770C	p.S257T	3	AAA
149	X	CXorf67	51150032	51150032	c.C164T	p.S55L	3	AAA
149	17	C17orf77	72588326	72588326	c.T16G	p.S6A	2	AA
149	3	TOP2B	25646268	25646268	c.C4457T	p.T1486M	4	AAAA
149	6	NKAPL	28227604	28227604	c.C455A	p.T152N	3	AAA
149	16	CENPBD1	90037828	90037828	c.C503T	p.T168I	2	AA
149	6	OR2J2	29142064	29142064	c.A652G	p.T218A	3	AAA
149	6	NKAPL	28227436	28227436	c.A287G	p.Y96C	3	AAA
151	3	ZNF197	44683542	44683542	c.C920T	p.A307V	3	AAA
151	X	DMD	32503194	32503194	c.A2621G	p.D874G	2	AA
151	2	TTN	179398509	179398509	c.G75638T	p.G25213V	3	AAA
151	12	OR10A7	55615094	55615094	c.G286A	p.G96S	3	AAA
151	12	FAM186A	50727706	50727706	c.C6684G	p.H2228Q	2	AA
151	X	RP11-87M18.2	36403036	36403036	c.A1817G	p.H606R	2	AA
151	2	TTN	179404628	179404628	c.A70969T	p.I23657F	3	AAA
151	22	PI4KA	21161673	21161673	c.G1145A	p.R382H	2	AA
151	X	DMD	32380996	32380996	c.G1211A	p.R404H	2	AA
151	12	ANKRD52	56639301	56639301	c.G2264C	p.R755P	3	AAA
151	5	PCDHA5	140249527	140249527	c.C839T	p.S280F	3	AAA
151	3	GNL3	52727257	52727257	c.G1099A	p.V367M	3	AAA
3324	21	KRTAP11-1	32253513	32253513	c.T331A	p.C111S	3	AAA
3324	4	FAM47E-STBD1	77192868	77192868	c.G817A	p.E273K	3	AAA
3324	X	NHS	17744510	17744510	c.T1690G	p.F564V	3	AAA
3324	X	DDX3X	41204711	41204711	c.G1177A	p.G393S	3	AAA

3324	3	KBTBD12	127641968	127641968	c.C64A	p.Q22K	2	AA
3325	20	ZNF831	57768743	57768743	c.C2669T	p.A890V	3	AAA
3326	X	HDHD1	6975782	6975782	c.G593C	p.C198S	2	AA
3326	19	DMKN	36004171	36004171	c.G207T	p.E69D	2	AA
3326	17	PYY	42030531	42030531	c.C215G	p.T72R	3	AAA
3327	X	FAM47C	37026830	37026830	c.C347T	p.A116V	3	AAA
3327	X	REPS2	17086525	17086525	c.A1207G	p.K403E	3	AAA
3327	X	ATRX	76931736	76931736	c.A3680G	p.N1227S	3	AAA
3327	10	WNT8B	102240866	102240866	c.G353T	p.R118L	2	AA
3327	19	HMHA1	1068738	1068738	c.G464A	p.R155H	2	AA
3327	2	DES	220283420	220283420	c.C236T	p.T79M	3	AAA
3327	X	RGAG1	109693960	109693960	c.G115A	p.V39I	3	AAA
3329	X	MAGEC1	140993875	140993875	c.G685C	p.A229P	3	AAA
3329	X	BEND2	18230715	18230715	c.T462G	p.F154L	2	AA
3329	19	MUC16	9058907	9058907	c.C28539G	p.H9513Q	4	AAAA
3329	22	TRIOBP	38121152	38121152	c.C2589A	p.N863K	3	AAA
3329	X	ZMAT1	101139254	101139254	c.C1145A	p.P382Q	3	AAA
3329	19	MUC16	9076083	9076083	c.C11363T	p.T3788I	4	AAAA
3336	9	NOTCH1	139409115	139409115	c.A2054C	p.N685T	5	AAAAA
3336	6	SYNJ2	158497754	158497754	c.A1678C	p.T560P	2	AA
3337	5	LHFPL2	77784941	77784941	c.G466T	p.A156S	1	A
3337	X	NKRF	118723880	118723880	c.C1508T	p.A503V	1	A
3337	2	KCNH7	163280034	163280034	c.G1945T	p.A649S	1	A
3337	7	RSBN1L	77407690	77407690	c.G1829T	p.C610F	1	A
3337	4	FAT4	126370253	126370253	c.C8088A	p.D2696E	1	A
3337	4	BOD1L1	13588088	13588088	c.G8365T	p.D2789Y	1	A
3337	2	PUS10	61180157	61180157	c.A1283G	p.D428G	1	A
3337	4	TIGD2	90034606	90034606	c.G481T	p.E161X	1	A
3337	5	CMYA5	79025711	79025711	c.G1123A	p.E375K	1	A
3337	6	HTR1B	78172216	78172216	c.A905T	p.K302M	1	A
3337	7	CTTNBP2	117450848	117450848	c.C385A	p.L129M	1	A
3337	2	NRXN1	50847258	50847258	c.C1222A	p.L408M	1	A
3337	5	PDE4D	59189315	59189315	c.G135T	p.Q45H	1	A
3337	4	BMP2K	79793795	79793795	c.C1636A	p.Q546K	1	A
3337	8	DPYS	105456535	105456535	c.A734G	p.Y245C	1	A
8184	X	HDHD1	6975782	6975782	c.G593C	p.C198S	4	AAAA
8184	2	RTP5	242814009	242814009	c.G302A	p.G101E	9	AAAAAAAAA
8184	X	RBMXL3	114425400	114425400	c.G1396A	p.G466R	4	AAAA
8184	17	KRTAP9-1	39346518	39346518	c.T380C	p.I127T	8	AAAAAAAA
8184	17	KRTAP4-7	39240819	39240819	c.C361G	p.L121V	8	AAAAAAAA
8184	2	UNC80	210824272	210824272	c.G7448A	p.R2483Q	3	AAA
8184	8	CYP11B1	143961102	143961102	c.G128A	p.R43Q	9	AAAAAAAAA
8186	15	MAP1A	43818052	43818052	c.G4381A	p.D1461N	2	AA
8186	X	ARMCX4	100749127	100749127	c.A5551G	p.I1851V	2	AA
8186	6	TREML4	41196605	41196605	c.T217C	p.W73R	2	AA
8188	19	ZNF568	37487873	37487873	c.C1088A	p.A363E	2	AA
8188	6	MUC21	30955179	30955179	c.G1227C	p.E409D	2	AA
8188	5	SH3RF2	145442203	145442203	c.G2129C	p.G710A	3	AAA

8188	2	TTN	179421694	179421694	c.T60992C	p.I20331T	2	AA
8188	2	ANKZF1	220097027	220097027	c.C307T	p.R103C	6	AAAAAA
8188	2	TTN	179397561	179397561	c.G76586A	p.R25529H	2	AA
8188	5	SH3RF2	145393364	145393364	c.C799T	p.R267C	3	AAA
8188	2	TTN	179623758	179623758	c.G10118A	p.S3373N	2	AA
8188	19	ZNF568	37487866	37487866	c.A1081T	p.S361C	2	AA
8188	2	TTN	179634421	179634421	c.A8749C	p.T2917P	2	AA
8188	11	NUP160	47857253	47857253	c.A1051G	p.T351A	2	AA
8188	6	MUC22	30995786	30995786	c.A2578G	p.T860A	2	AA
8188	2	TTN	179629461	179629461	c.G9643A	p.V3215M	2	AA
8188	3	CSRNP1	39184959	39184959	c.G1357A	p.V453I	6	AAAAAA
8188	8	TRMT12	125463250	125463250	c.T82C	p.W28R	3	AAA
8191	17	HDAC5	42164885	42164885	c.C1782G	p.D594E	5	AAAAA
8191	12	RAD9B	110960196	110960196	c.G898A	p.G300R	2	AA
8191	15	ZNF106	42758294	42758294	c.C73T	p.R25W	4	AAAA
8191	X	GPC3	132833929	132833929	c.G998A	p.R333Q	3	AAA
8191	X	DMD	32632562	32632562	c.G1316A	p.R439K	3	AAA
8191	5	NSD1	176637576	176637576	c.T2176C	p.S726P	2	AA
8191	X	TENM1	123630915	123630915	c.G3646T	p.V1216L	3	AAA
8191	1	AIM1L	26671248	26671248	c.T1901C	p.V634A	5	AAAAA
8194	19	ZNF419	58004346	58004346	c.G286C	p.E96Q	3	AAA
8194	10	KIF20B	91497912	91497912	c.A3314G	p.K1105R	3	AAA
8194	4	ZGRF1	113540718	113540718	c.G480C	p.L160F	3	AAA
8194	4	FREM3	144617606	144617606	c.A4223T	p.N1408I	3	AAA
8194	X	P2RY4	69478942	69478942	c.A533C	p.N178T	3	AAA
8194	16	GRIN2A	9857958	9857958	c.C3443T	p.P1148L	3	AAA
8194	4	TET2	106156909	106156909	c.C1810T	p.Q604X	3	AAA
8194	6	HLA-A	29910693	29910693	c.A233G	p.Q78R	4	AAAA
8194	4	DKK2	107845794	107845794	c.G437A	p.R146Q	3	AAA
8194	19	ZNF264	57723007	57723007	c.G542C	p.R181T	3	AAA
8194	19	ZNF264	57723013	57723013	c.G548A	p.R183H	3	AAA
8194	19	VN1R1	57967133	57967133	c.C722T	p.S241F	3	AAA
8194	18	MEP1B	29793211	29793211	c.C1268T	p.S423L	3	AAA
8194	10	COL17A1	105824333	105824333	c.C629T	p.T210M	3	AAA
8198	X	MXRA5	3240343	3240343	c.C3383T	p.A1128V	2	AA
8198	11	CHID1	870446	870446	c.C920T	p.A307V	1	A
8198	7	OR2AE1	99474427	99474427	c.T230C	p.I77T	2	AA
8198	4	LCORL	17885365	17885365	c.A1787G	p.K596R	2	AA
8198	15	AKAP13	86122654	86122654	c.T1355C	p.M452T	4	AAAA
8198	3	MYH15	108159977	108159977	c.C2846T	p.T949I	3	AAA
8198	15	AKAP13	86123988	86123988	c.G2689A	p.V897M	4	AAAA
8199	16	HS3ST6	1961674	1961674	c.C946G	p.L316V	3	AAA
8199	X	REPS2	17153504	17153504	c.A1780G	p.M594V	2	AA
8199	X	ATRX	76953110	76953110	c.C203T	p.S68L	3	AAA
8208	1	BTBD19	45275892	45275892	c.T94C	p.C32R	2	AA
8208	20	MYT1	62839425	62839425	c.G876T	p.E292D	2	AA
8208	X	DCAF8L1	27998800	27998800	c.T652G	p.L218V	3	AAA
8208	X	BEND2	18220008	18220008	c.T960G	p.N320K	3	AAA

8208	14	ADAM21	70924335	70924335	c.C119T	p.P40L	2	AA
8208	10	PDCD11	105184755	105184755	c.C2778A	p.S926R	2	AA
8210	X	MXRA5	3228144	3228144	c.G8100C	p.E2700D	3	AAA
8210	X	RPS6KA6	83442896	83442896	c.C12A	p.F4L	4	AAAA
8210	3	FAM198A	43074074	43074074	c.T319A	p.S107T	3	AAA
8219	1	NBPF12	146400170	146400170	c.G646A	p.G216S	4	AAAA
8219	2	STK25	242438696	242438696	c.C561G	p.I187M	5	AAAAA
8219	12	GYS2	21733432	21733432	c.G147C	p.Q49H	2	AA
8221	X	TMEM47	34675139	34675139	c.C8T	p.S3L	3	AAA
8751	3	CELSR3	48699499	48699499	c.A569T	p.N190I	7	AAAAAAA
8751	X	BCYRN1	70444065	70444065	c.G508T	p.V170F	7	AAAAAAA
9616	9	FAM166A	140139794	140139794	c.G487A	p.A163T	2	AA
9616	1	NBPF20	145075705	145075705	c.G158A	p.R53H	2	AA
9622	2	KHK	27317406	27317406	c.A271C	p.T91P	2	AA
9623	6	SYNE1	152763329	152763329	c.G3910A	p.A1304T	2	AA
9624	9	PIP5KL1	130692077	130692077	c.C118T	p.R40C	5	AAAAA
9624	7	KPNA7	98790650	98790650	c.A628C	p.T210P	4	AAAA
9634	X	MAGED1	51638851	51638851	c.C748T	p.R250C	2	AA
9634	4	MUC7	71347185	71347185	c.T724C	p.S242P	1	A
9638	6	MYO6	76566846	76566846	c.C1256T	p.A419V	1	A
9638	7	FZD1	90895963	90895963	c.G1768A	p.A590T	1	A
9638	7	PUS7	105108822	105108822	c.G1487T	p.G496V	1	A
9638	9	TRPM6	77377716	77377716	c.C3856A	p.H1286N	1	A
9638	10	ZNF33B	43089179	43089179	c.C1219A	p.H407N	1	A
9638	7	SLC26A4	107335127	107335127	c.T1403C	p.I468T	1	A
9638	4	NKX6-1	85414591	85414591	c.A955G	p.N319D	1	A
9638	2	ORC4	148716428	148716428	c.A11G	p.N4S	1	A
9638	9	LINGO2	27949105	27949105	c.A1565G	p.N522S	1	A
9638	17	MYHAS	10346774	10346774	c.G5738A	p.R1913Q	1	A
9638	8	GEM	95262720	95262720	c.C709T	p.R237X	1	A
9638	8	KLHL38	124665039	124665039	c.G128T	p.R43L	1	A
9638	10	ITGA8	15590522	15590522	c.C2767T	p.R923X	1	A
9638	4	ENAM	71510366	71510366	c.A3223T	p.S1075C	1	A
9638	13	GPC6	94482467	94482467	c.C380A	p.T127N	1	A
9638	18	ESCO1	19154143	19154143	c.C662T	p.T221M	1	A
9638	4	FAM184B	17710691	17710691	c.G718A	p.V240M	1	A

*Based on NCBI human reference genome GRC Build 37 (hg19).
